# Developmental Control of *NRAMP1* (*SLC11A1*) Expression in Professional Phagocytes

**DOI:** 10.3390/biology6020028

**Published:** 2017-05-03

**Authors:** Mathieu F. M. Cellier

**Affiliations:** Inrs-Institut Armand-Frappier, 531, Bd des prairies, Laval, QC H7V 1B7, Canada; mathieu.cellier@iaf.inrs.ca; Tel.: +1-450-687-5010 (ext. 4681)

**Keywords:** natural resistance-associated macrophage protein (NRAMP), solute carrier family 11 (SLC11), hematopoiesis, professional phagocytes, gene expression, transcription factor, epigenetics, single nucleotide polymorphism, expression quantitative trait locus (eQTL)

## Abstract

NRAMP1 (SLC11A1) is a professional phagocyte membrane importer of divalent metals that contributes to iron recycling at homeostasis and to nutritional immunity against infection. Analyses of data generated by several consortia and additional studies were integrated to hypothesize mechanisms restricting *NRAMP1* expression to mature phagocytes. Results from various epigenetic and transcriptomic approaches were collected for mesodermal and hematopoietic cell types and compiled for combined analysis with results of genetic studies associating single nucleotide polymorphisms (SNPs) with variations in *NRAMP1* expression (eQTLs). Analyses establish that *NRAMP1* is part of an autonomous topologically associated domain delimited by ubiquitous CCCTC-binding factor (CTCF) sites. *NRAMP1* locus contains five regulatory regions: a predicted super-enhancer (S-E) key to phagocyte-specific expression; the proximal promoter; two intronic areas, including 3′ inhibitory elements that restrict expression during development; and a block of upstream sites possibly extending the S-E domain. Also the downstream region adjacent to the 3′ CTCF locus boundary may regulate expression during hematopoiesis. Mobilization of the locus 14 predicted transcriptional regulatory elements occurs in three steps, beginning with hematopoiesis; at the onset of myelopoiesis and through myelo-monocytic differentiation. Basal expression level in mature phagocytes is further influenced by genetic variation, tissue environment, and in response to infections that induce various epigenetic memories depending on microorganism nature. Constitutively associated transcription factors (TFs) include CCAAT enhancer binding protein beta (C/EBPb), purine rich DNA binding protein (PU.1), early growth response 2 (EGR2) and signal transducer and activator of transcription 1 (STAT1) while hypoxia-inducible factors (HIFs) and interferon regulatory factor 1 (IRF1) may stimulate iron acquisition in pro-inflammatory conditions. Mouse orthologous locus is generally conserved; chromatin patterns typify a de novo myelo-monocytic gene whose expression is tightly controlled by TFs Pu.1, C/ebps and Irf8; Irf3 and nuclear factor NF-kappa-B p 65 subunit (RelA) regulate expression in inflammatory conditions. Functional differences in the determinants identified at these orthologous loci imply that species-specific mechanisms control gene expression.

## 1. Introduction

*NRAMP1* gene encodes a phagocytosis-related function that is specifically expressed in mature myelo-monocytic cells. It was discovered as a host factor mediating resistance to intracellular pathogens that replicate inside phago-lysosomes in macrophages (MFs) [1]. NRAMP1 is an integral membrane protein catalyzing proton-dependent transport of divalent metal ions, such as iron and manganese, out of phago-lysosomes into the cytoplasm; it is also known as SLC11A1 (SLC11 family member 1 [2]). NRAMP1/SLC11A1 activity is thus key to host nutritional immunity by depriving ingested microbes from vital micro-nutrients [3]. In addition, NRAMP1 contributes to MF recycling of divalent metals, notably iron, post-ingestion of apoptotic cells and aged erythrocytes [4,5]. Hence *NRAMP1* gene expression is associated with both pro- and anti-inflammatory activities of professional phagocytes. This dual role in nutrition and immunity has ancient origin since proton-dependent metal depletion of phagosomes mediated by NRAMP1 is conserved in the social amoeba *Dictyostelium discoideum* [6,7,8].

Analysis of high throughput datasets (mostly from ENCODE consortium [9,10]) depicting DNAse footprinting (DNase 1 hypersentitive sites, DHSs), chromatin immuno-precipitations coupled to deep sequencing (ChIP-seq) and targeting specific histone modifications or RNA polymerase II (RNA Pol II), CCCTC-binding factor (CTCF) and various transcription factors (TFs) interacting with *NRAMP1* locus, in both acute myeloid leukemia (AML) cell lines and primary monocytes, allowed us to delineate a ~40 kb regulatory domain insulated by CTCF sites [11]. This postulated regulatory domain comprises several hypothetical determinants, located upstream of or within *NRAMP1* gene, which may regulate transcription either positively or negatively depending on the TF involved, the developmental stage of myeloid precursors as well as the immune context and tissue-specific environment later encountered by mature cells. Data analysis confirmed in particular the crucial role previously established for the TF CCAAT enhancer binding protein beta (C/EBPb) at *NRAMP1* proximal promoter [12], and further suggested plausible contributions of PU.1 and EGR2 [13].

Integrating data obtained with several cell types that represent various stages of myelopoiesis suggested that sequential mobilization of regulatory elements during the developmental maturation of monocytic and granulocytic cells dictates the specificity of *NRAMP1* expression [11]. The purpose of the present analysis is to formulate refined hypotheses that can be tested experimentally to decipher the molecular control of *NRAMP1* expression. For this, the most recent high throughput datasets (from NIH Roadmap [14]; EU Blueprint epigenome [15], and RIKEN FANTOM5 [16,17]) obtained using several AMLs and blood cell types were analyzed to further test postulated cell-type specific determinants of *NRAMP1* expression and to interpret their possible role in controlling gene transcription, notably in the context of recent advances regarding enhancer function.

Gene expression controlling enhancers may be predicted without knowing the TFs involved by integrating complementary bodies of epigenetic data produced by high throughput experiments. Cell type-specific enhancers are formed by the juxtaposition of several binding sites specific for various TFs (separated by ~20–100 bp). They can act independently of their distance from, and orientation relative to, promoter elements through (inter)chromosomal looping or facilitated tracking [18]. Enhancers may also interact with different transcriptional start sites to elicit alternative gene expression. While inactive enhancers are buried in compact chromatin (heterochromatin), active enhancers lie in regions of lightly packed chromatin (euchromatin) which allows transcription of enhancer RNA (eRNA) [19].

Activation of mammalian enhancers begins with the binding of both lineage-specific TFs and collaborating TFs at neighbor sites still embedded in nucleosomes [20]. Instrumental to this process pioneer TFs (pTFs) can target silent genes locked in chromatin by binding partial or degenerate motifs that are exposed along one face of the DNA while the other face remains interacting with nucleosomal histone octamer. Binding of the pTF does not interfere with constraints imposed on DNA bound to nucleosomes while secondary events involving chromatin remodelling factors lead to subsequent changes in local DNA structure and accessibility [21]. pTFs may directly act as or recruit additional factors acting as master regulators indispensable to specify cell lineage [18,22,23].

Enhancer activation may be initiated in progenitor cells, for example hematopoietic stem cell (HSC) precursors or myeloid progenitors, and proceeds with recruitment of co-regulators such as the Mediator complex, ATP-dependent chromatin remodelling factors and histone acetyltransferases (HAT) which allow nucleosome remodelling to accommodate RNA Pol II binding. HATs such as p300 and CBP (cAMP-response element-binding protein (CREB)-binding protein) may acetylate lysine residues in the tail of some histones (e.g., histone 3 acetylation at lysine 27: H3K27ac or K27ac), while KAT8/MYST1 may acetylate lysine residue 16 of histone 4 (H4K16ac). Acetylation marks are recognized and bound by bromodomain-containing proteins and positive transcription elongation factor (P-TEFb) complex, which allow the recruiting of general TFs forming the pre-initiation complex as well as RNA Pol II and associated kinases and mixed lineage leukemia proteins (MLL1/3/4) that deposit mono- and di-methylation marks at lysine 4 of histone 3 (H3K4me1/2 [18,22]). Enhancer activation may also include regulation of DNA CpG dinucleotide methylation state, as CpG are bound by various factors either in methylated or unmethylated state, and cytosine methylation can be erased through active and/or passive oxidative steps [24].

The enhancer activation process may be stalled after binding of pioneer TFs, in the absence of further recruitment of additional TF, letting the enhancer in a primed state (showing low activity), or ‘poised’ in case histone deacetylases (HDAC) are recruited, limiting histone acetylation, together with additional histone methylase (e.g., EZH2) that introduce marks which inhibit RNA transcription (e.g., H3K27me3). Then, a novel signal-dependent TF may be recruited to primed or ‘poised’ enhancer and allow the resumption of the activation process: chromatin remodelling will spread to neighboring nucleosomes and ultimately allow transcription of eRNAs and open reading frame (ORF) mRNA. Alternatively, ‘de novo’ enhancers are activated in a single step as both lineage- and signal-dependent TFs are recruited together to trigger RNA Pol II activity [22,25].

One major advance in recent years has been the discovery of eRNAs, indicating that RNA Pol II transcribes not only ORFs and genes encoding regulatory RNAs but also short segments of chromosomal regulatory elements such as enhancers [26,27,28,29,30,31]. Though the potential function of eRNAs is still not clear, RNA Pol II transcriptional activity at enhancer loci contributes to their functional priming [32] and may precede their labelling with histone methylation marks such as H3K4me1 and H3K4me2 [33,34]. Possible roles of eRNAs include acting as decoy molecules to release the negative elongation factor from paused RNA Pol II [18] or as low affinity binding sites tethering certain TFs at gene regulatory sites to favor stable gene expression (e.g., YY1 and CTCF) [35].

In general, enhancers display tissue-specific transcription and eRNA transcripts prominently contribute to enhancer-mediated activation of tissue-specific gene expression programs [36]. In particular, knock-down of *IL1b* eRNAs in human monocytic cells attenuates both lipopolysaccharide (LPS)-induced production of *IL1b* and *CXCL8* mRNAs and subsequent release of the corresponding mediators, indicating that expression and release of proinflammatory cytokines is indeed regulated by nuclear-located non-coding transcripts [37]. Given the plasticity of mononuclear phagocyte developmental programs and the polarizability of mature cell phenotypes it is anticipated that characterizing the enhancer repertoire of MF populations will help defining the molecular basis of their phenotypic diversity [38].

A subset of remarkable enhancers also has recently emerged that were denominated super-enhancers (S-Es) because they apparently control mammalian cell identity and disease [39,40]. S-Es were defined by demonstrating that the master TFs which control embryonic stem cells (ESC) pluripotent state do so by activating unusually large regulatory domains at loci implicated in maintenance of the pluripotent state. Similarly, in differentiated cells, S-Es were evidenced at genes that control cell functional identity by showing S-Es bind multiple cell-type specific master TFs. In contrast, S-Es were not found at house-keeping genes [39]. S-Es constitute relatively dense TF-binding platforms formed by clustered enhancers that bind specific pioneer regulators; by sensing pTFs abundance S-Es may conduct dynamic chromatin modelling and integrate developmental regulation of cell-type-specific loci [41].

Several properties distinguish S-Es from common enhancers by an order of magnitude: S-Es are larger (mean size ~8.7 kb vs. ~700 bp); ChIP-seq analyses indicated S-Es display higher levels of histone marks of activation such as H3K4me1 and H3K27ac; also, they bind larger amount of the Mediator complex (Med1 component), which couples physically active enhancers and promoters, and knockdown of Med12 component preferentially affected the expression of genes governed by S-Es [39]. It was also observed that binding of PU.1 and Med at enhancers are highly correlated in pro-B cells, and in MFs, C/EBPa binds large domains comprising clusters of enhancers [39]. Lastly, S-Es act as regulatory hubs (RHs) that coordinate eRNA and target gene transcription; S-Es are highly responsive to external signals because they bind TFs that are terminal effectors of signalling pathways (e.g., SMAD3 for TGFb pathway and NFKB p65 for TLR4 signalling [42,43]).

S-E functional properties may be relevant to *NRAMP1* transcriptional control because several candidate enhancers bind both PU.1 and C/EBPb, and some of them form a large domain upstream of *NRAMP1* [11,13]. In addition, MF S-Es were associated with genes encoding activities which are critical to cell-type specific functions such as wound response, membrane organization and invagination, endocytosis, immune and inflammatory responses [39]. Given that NRAMP1 exerts a cardinal function of professional phagocytes (depletion of the phagosome lumen from vital transition metals such as Fe^2+^, Mn^2+^ and Co^2+^) and since its expression is developmentally controlled during myelopoiesis, and further regulated by infectious and inflammatory signals, it seems possible that an S-E domain controls *NRAMP1* transcription.

Because active enhancers (including S-Es) overlap with RNA Pol II bound regions and display (bidirectional) elongation of eRNAs, evaluating the transcriptional status of potential regulatory elements previously delineated at *NRAMP1* locus should inform on regulation of gene expression. Cap analysis of gene expression (CAGE) was thus examined along *NRAMP1* locus to seek functional evidence that would support postulated enhancer determinants, and to study their activity level in human blood cell types.

In addition, single cell analyses revealed that H3K27ac can regulate downstream transcription kinetics by favoring TF recruitment and accelerating RNA Pol II promoter escape, i.e., transition from initiation toward elongation of transcription [44]. In contrast, inhibiting transcript elongation blocks ligand-induced H3K9ac but not H3K27ac [45]. Deposition of these histone marks at *NRAMP1* locus was thus re-examined together with that of H3K4me3, another transcription-dependent mark and one of the most frequently associated with a combinatorial ‘histone code’ for enhancer transcriptional activity [46], to determine how they may correlate with RNA transcription (CAGE) data.

To further document potential activity of the candidate enhancers studied, local distribution of selected histone marks was compared with the location of DHSs and transcriptionally active sites [47] in way to distinguish between primed or active states; the status of CpG dinucleotide methylation and the distribution of predicted TFs binding sites (TFBS) were also monitored as complementary properties. Altogether, available data regarding *NRAMP1* locus in various cell-types were integrated using the UCSC genome browser [48] to consolidate functional hypotheses regarding the developmental control of *NRAMP1* transcription.

Four possible classes of regulatory elements [49] were used as a guide: (i) active transcriptional regulatory elements, which correspond to DHS and are flanked by active histone marks (e.g., H3K27ac and H3K9ac) while the central area is largely nucleosome-free and may bind high density of TFs; (ii) transcriptionally inactive ‘open’ regulatory elements (DHS with active histone marks, e.g., H3K27ac and H3K4me1), where only the central nucleosome is displaced and few TFs bind; (iii) marked potential regulatory elements showing limited histone marking (e.g., H3K4me1) and (iv) insulator and regulatory sites, bound by CTCF, RAD21 and/or SMC3 and corresponding to DHS.

Because Fe metabolism is key to MF function, the transcriptional/regulatory status of other genes known to participate in MF Fe metabolism was also examined. A summary is provided to describe loci carrying genes that display expression in blood cells relatively similar to *NRAMP1*. This set includes genes that exhibit tissue-specific expression profile similar to *NRAMP1*, as indicated by GTEx datasets [50] i.e., high expression levels in vivo in blood and/or lung, spleen, and which encode functions that contribute to iron (including heme) acquisition, iron storage and lysosomal recycling. A fraction of these exhibit candidate S-Es that may enable coordinated regulation of MF iron genes.

Lastly, to evaluate whether *NRAMP1* epigenetic regulation is conserved, the expression of the mouse orthologous gene *Nramp1* was examined during embryogenesis and organognesis; activation of *Nramp1* locus was studied during hematopoiesis, both primitive and definitive, and compared between cells either explanted or cultured in vitro. Also an inventory was made for the TFs interacting with *Nramp1* candidate regulatory elements in various cell types. These analyses revealed that both the myelo-monocytic specificity and timing of expression during myeloid differentiation are common to *Nramp1* and *NRAMP1* orthologs, as well as involvement of master TFs such as Pu.1 and C/ebps. However, the candidate regulatory elements identified and their pattern of mobilization appeared divergent between mammalian species.

## 2. Results and Discussion

### 2.1. Functional Delineation of NRAMP1 Locus by CTCF Sites

**Figure 1:**
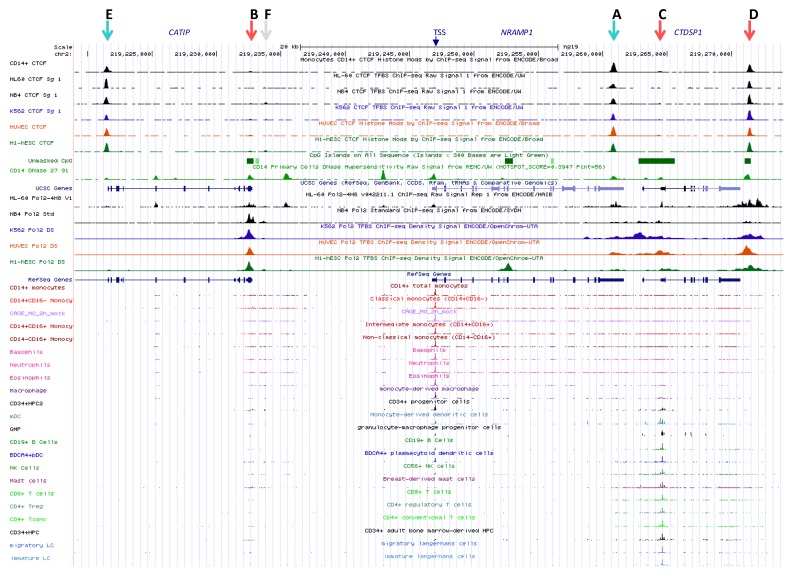
CAGE at *NRAMP1* locus in blood cell types. From top to bottom: CTCF sites are shown by arrows (red, forward orientation; blue, reverse orientation; grey, undetermined), including *NRAMP1* locus boundaries (sites A and E), and *NRAMP1* TSS as well as surrounding genes are indicated; chromosome 2 scale; CTCF ChIP-seq data for CD14^+^ monocytes (MNs), acute promyelocytic leukemia HL-60 and NB4, erythroleukemia K562, human umbilical vein epithelial cells (HUVEC) and embryonic stem cells (ESC; data from ENCODE consortium [9,10]); CpG islands; DNAse1 footprints: hypersensitive sites (DHS) in CD14^+^ MNs (ENCODE) [9,10]; UCSC browser genes; RNA Pol II ChIP-seq data for HL-60, NB4, K562, HUVEC and ESC (ENCODE); RefSeq genes; CAGE data [16,17] for 22 blood cell types.

It has been proposed that *NRAMP1* locus spans a chromosome 2 segment of ~40 kb, delimited by two strong CTCF binding sites [11] that were previously revealed by ChIP-seq analyses in several cell types [9,10]. One CTCF site would mark the locus 5′ boundary upstream of *CATIP* gene while the other lies at *NRAMP1* 3′ UTR (sites E and A, respectively, Figure 1), implying these sites may act as insulator elements. Yet, widening UCSC genome browser window [48] shows that *NRAMP1* locus represents the 3′ end of a larger ensemble (chr2:219,075,000-219,280,000). This extended domain (205 kb) is characterized by similar patterns of DHS that indicate myeloid gene expression; blocks of co-localizing H3K27ac/H3K4me1 marks, distribution of PU.1 binding sites, as well as relatively similar decoration with histone marks of transcriptional activity (Figure S1A).

Could this 205 kb region constitute a larger regulatory domain that controls gene expression or alternatively, do *NRAMP1* flanking CTCF sites function as bona fide insulating boundaries?

Three lines of evidence argue that *NRAMP1* ~40 kb locus likely represents an autonomous regulatory domain: (i) transcription data reveal *NRAMP1* is the sole gene that shows strictly myelo-monocytic specific expression (Figure S1B); (ii) ENCODE data for chromatin interaction analysis by paired-end tag sequencing (ChIA-PET) [51,52], obtained for both K562 megakaryocytic cells and MCF-7 epithelial cells [53], indicate similar chromatin loops that suggest insulation of *NRAMP1* locus by its flanking CTCF sites (Figure S1B,C); and (iii) single nucleotide polymorphisms (SNPs) that were identified at genome-wide significance as potential quantitative trait loci affecting *NRAMP1* expression in *cis* (cis eQTLs) reveal a possible regulatory influence of the areas carrying CTCF sites E and A (Figure S1D) [54].

Genome organization in topological domains relies on CTCF binding to sites that influence transcription either by facilitating spatial clustering of distal DNA elements, such as enhancer-promoter contacts, or by insulating distal elements away from non-target genes to prevent unwanted contacts [55,56]. A loop extrusion model has been proposed to explain the orientation bias seen in CTCF-mediated looping [57,58,59,60]: CTCF binding bends DNA, favoring loop formation in one direction so that parallel interactions between an upstream forward (F) and a downstream reverse (R) motif are generally observed. CTCF-mediated looping may then be expanded by the action of cohesin and/or transcriptional machinery allowing additional interactions with additional CTCF binding sites and/or other DNA elements.

*NRAMP1* locus shows six CTCF sites (A–F) which include, based on binding strength, three major (A, D, E) and three minor sites (B, C, F; Figure 1). Based on published CTCF consensus sequences [61,62,63] sites A and E are in reverse orientation while sites B-D are oriented forwardly, and site F is undetermined (Figure 1 and Figure S1C). Current model of asymmetric loop formation [57,58,60] predicts that CTCF sites displaying “R” orientation (sites E and A, Figure 1) may anchor loops that extend to upstream “F” CTCF sites. Such loops may either allow interactions between functional determinants that control *NRAMP1* expression (e.g., interaction of sites A and B) or insulate *NRAMP1* locus from upstream determinants that control other genes (e.g., loop(s) involving site E). In addition, *NRAMP1* CTCF sites in “F” orientation (e.g., C or D) may be part of loops directed by downstream CTCF sites, which would contribute to insulate *NRAMP1* as well.

To investigate *NRAMP1* CTCF looping pattern ChIA-PET data derived from K562 and MCF-7 cells were examined. The results appeared consistent between both cell types. Hence, site D, which is the most downstream “F” site, was found in loops involving further downstream areas, each comprising a CTCF site in “R” orientation (chr2:219,330,316-479 (K562)/219,330,530-1,339 (MCF7) and 219,512,425-946 (K562); indicated as sites 331 and 513, respectively, Figure S1B,C). Regarding the “R” sites A, located within *NRAMP1* 3′ UTR, and E, forming the locus 5′ boundary, both are part of loops connecting further upstream areas that carry an “F” site (219,156,895-7,776 (K562); 219,136,806-8,047 (K562)/219,137,148-8,025 (MCF7); 219,165,729-6,647 (K562)/219,165,757-6,700 (MCF7) and 219,165,724-6,789 (K562)/219,165,749-6,710 (MCF7); 219,137,780-8,521 (K562)/219,136,040-8,028 (MCF7)), respectively (indicated as sites 137, 157, 166, Figure S1). Because similar loops were observed both in K562 and MCF-7 cells, it seems plausible similar domains are also formed in myelo-monocytic cells to insulate *NRAMP1* 40 kb locus.

ChIA-PET data in myeloid K562 cells showed that CTCF site E preferentially interacts with upstream site 166 (score 800) whereas CTCF site A favors looping with site 157 (score 500). Both sites A and E may also interact with alternate sites, respectively 157 and 166, showing reduced scores (400 and 200). Accordingly, it is possible to envision formation of one large loop (site 157-CTCF site A), which would include a smaller one (site 166-CTCF site E) insulating the upstream gene *PNKD* (Figure S1A,B). The chromosome segment separating sites 157 and 166 contains no gene or transcriptionally active area. Accordingly, the ~40 kb interval delineated by CTCF sites E and A may comprise all the regulatory elements required to control *NRAMP1* expression.

The potential role of CTCF sites A and E in the regulation of *NRAMP1* expression is further underscored by candidate eQTLs found in both areas in whole genome association studies (Figure S1D). Hence, genetic evidence in line with epigenetic data imply CTCF sites E and A insulate *NRAMP1* locus, and suggest an important regulatory role of its 3′ boundary (Figure S1B–D).

### 2.2. Regulatory Determinants Identified by Cap Analysis of NRAMP1 Expression

CAGE reveals transcription patterns based on 5′ end-derived sequence tags obtained by adding a linker to the 5′ end of cDNAs followed by Nextgen sequencing [64]. As human gene promoters may display intrinsic directional bias [17,65,66,67] enhancers show non-overlapping bidirectional transcription, and CAGE enables to identify both types of regulatory elements [16,68]. In addition, using transcriptional activation to detect regulatory elements allowed to identify the majority of sites that control gene expression [49].

#### 2.2.1. BloodCAGE (TrackHub CAGE of Haematopoietic Cell Types)

CAGE of human blood cell types allows us to compare *NRAMP1* locus transcriptional activity between divergent lineages whose development controls gene expression. Data compiled for the 39.5 kb interval insulated by CTCF sites A and E indicate different transcriptional signatures based on the relative intensity of *NRAMP1* (*SLC11A1*) transcriptional start sites (TSS) compared to the downstream gene *CTDSP1* TSS, while *C2Orf62*/*CATIP* gene, situated upstream of *NRAMP1*, is not expressed in the cell types tested (Figure 1 and Figure S2A).

*CTDSP1* encodes a class 2C phosphatase whose preferred substrate is RNA Pol II phosphorylated by TFIIH; CTDSP1 activity is required to initiate transcription and it is ubiquitously expressed [69,70,71]. Accordingly, BloodCAGE shows *CTDSP1* transcription level is relatively constant, varying about 2.5 fold among the cell types tested.

In contrast, *NRAMP1* transcription varies over two orders of magnitude and consequently, *CTDSP1* transcription appears negligible in cells expressing *NRAMP1* at high level. Three transcriptional signatures were observed: *NRAMP1* TSS is the most intense signal indicated by CAGE tag clustering; *CTDSP1* TSS appears sole hugely marked by CAGE tags; both *NRAMP1* and *CTDSP1* TSSs display similar CAGE tag clusters (Figure 1 and Figure S2A).

##### 2.2.1.1. *NRAMP1* Expressing Blood Cells

Herein, CAGE tags cover both *NRAMP1* ORF and the following gene (*CTDSP1*); they also mark additional DNA segments upstream of *NRAMP1*, some of which lie within *C2Orf62*/*CATIP* and were previously identified as potential enhancing elements based on specific chromatin marks (K9ac/27ac, K4me1/2/3), DHSs and TF binding (e.g., RNA Pol II, PU.1, C/EBPb, EGR2) [11]. In fact, large scale genomic data previously revealed that a majority (58%) of DHSs reside within transcription units (intronic DHSs), and some of them have been shown to regulate nearby genes in hematopoietic lineages [72]. *C2Orf62*/*CATIP* is strongly expressed in testis [73]; it is thus predicted that candidate myelo-monocytic enhancers embedded in *C2Orf62*/*CATIP* constitute bona fide determinants of *NRAMP1* expression.

*NRAMP1* expressing cell-types are professional phagocytes; ranked by decreasing level of transcription, they include: CD14^+^ CD16^−^ monocytes (MNs), CD14^+^ CD16^+^ MNs, CD14^−^ CD16^+^ MNs, neutrophils, basophils, eosinophils, and MN-derived MFs (MDMs; Figure 1) [68]. These data are consistent with previous analyses that indicated blood neutrophils, MNs and alveolar MFs as the cell types showing high level of *NRAMP1* mRNA [74].

##### 2.2.1.2. Blood Cells Not Expressing *NRAMP1*

In these cell types, capped transcription tags span an interval beginning at the 3′ end of *NRAMP1* and covering *CTDSP1* downstream ORF. CAGE tags also mark a small area situated at the end of *CATIP* (Figure 1 and Figure S2A). *NRAMP1* negative blood cell types include mast cells (MCs), some CD34^+^ hematopoietic progenitors, CD8^+^ T cells, CD4^+^ regulator T cells, conventional CD4 T cells, immature and migratory Langerhans cells (LCs).

##### 2.2.1.3. Blood Cells Expressing *NRAMP1* at Intermediate Levels

In this case, *CTDSP1* TSS is marked more intensely than *NRAMP1* TSS, except for some (other samples of) CD34^+^ hematopoietic progenitors. Transcription tags appear scattered along both genes and at some predicted upstream enhancer elements (Figure 1, Figure S2A). These cell types include MN-derived dendritic cells (MDDCs), granulocyte-MF progenitor (GMP) cells, CD19^+^ B cells, BDCA4^+^ plasmacytoid dendritic cells and CD56^+^ natural killer cells.

In MDDCs, CD34^+^ progenitors, MCs and in a pool of AMLs samples another cluster of CAGE tags maps at the 3′ end of *NRAMP1*, within intron 12 and immediately preceding exon XIII (Figure S2A,B). This area also binds RNA Pol II in model megakaryocytic-erythrocytic progenitors (MEP) (K-562, erythroleukemia; CMK megakaryoblast); in precursors of phagocytes such as NB4 cells, and to lesser extent in HL-60 cells. It also corresponds to a DNAse footprint (F10) in myeloid cell types apparently not expressing *NRAMP1* (either at appreciable level: G-CSF mobilized CD34^+^ progenitors (mCD34) or not at all: MEP and (acute promyelocytic leukemia, APL) NB4 cells; Figure S2).

Co-detection of bound RNA Pol II and CAGE tags at the 3′ end of *NRAMP1* ORF in cells expressing little or no mRNA thus indicate an active element that may impact gene expression negatively. Because intragenic enhancers can act as alternative TSS, producing low protein-coding potential RNAs [75], and since *NRAMP1* intron 12 CAGE tags map on the forward strand (Figure S2A), F10 element may thus represent an alternative 3′ TSS antagonizing full-length gene expression in some myeloid cell types (e.g., erythrocytic, DC and MC lineages).

BloodCAGE data [68] analysis indicates high level *NRAMP1* transcriptional activity specifically in microbicidal phagocytes, in accordance with phagocytosis-related roles of NRAMP1 [76,77]. BloodCAGE data also show *NRAMP1* locus carries both positive and negative determinants that may be mobilized sequentially or alternatively depending on myeloid fates.

#### 2.2.2. *NRAMP1* CAGE in AML Cells

Detailing *NRAMP1* transcription status in cell lines representing various stages of myelo-monocytic progenitors confirms the presence of a negative determinant at the gene 3′ end.

##### 2.2.2.1. An Alternative *NRAMP1* TSS in AMLs?

The main cluster of CAGE tags detected at *NRAMP1* locus in a pool of 21 cell lines maps on the forward strand at the putative “3′ alternate TSS”/negative determinant that precedes exon XIII (Figure S2). One cell line accounts for most tags per million (tpm) detected at this site (EEB, 90 tpm) but more than three tpm were also reported in six other cell lines (F-36P, 17.6 tpm; THP-1, 12.5 tpm; P31/FUJ, 7.1 tpm; MKPL-1, 4.8 tpm; F-36E, 4.6 tpm; M-MOK, 3.6 tpm) [68].

Comparing tpm counts at *NRAMP1* 5′ and “3′ alternative” TSS and at *CTDSP1* TSS in CD14^+^CD16^−^MNs (61.7/0.05/3.1 tpm, respectively), CD34^+^ progenitors (4.9/0.3/2.9 tpm), MDDCs (1.4/1.9/3.1 tpm), AMLs (0.2/0.5/2.4 tpm) and MCs (0.03/0.2/4.8 tpm) shows some inverse relationship between *NRAMP1* 5′ and “3′ alternative” TSSs. One possible interpretation suggests antagonism between these “alternative” TSSs due to negative activity of intron 12 DHS F10.

Interestingly, the 3′ portion of the CAGE tag cluster mapping to *NRAMP1* intron 12 is adjacent to the poly-pyrimidine stretch of the intron 3′ junction; it shows strong conservation among simians (e.g., 100% identity with various species of Old World monkeys). This conserved area also overlaps a potential binding site for TF JunD/AP1 (located in the middle of a JunD ChIP-seq segment obtained in K562 cells, Figure S2B) [9,10].

Other ChIP-seq data from K562 cells indicate several possible binding sites for C/EBPs downstream of F10 putative regulatory element, which span the end of intron 12 and beginning of exon XIII [78]. Additional binding sites predicted from K562 ChIP-seq data lie more upstream, at the beginning of the CAGE tag cluster: one is specific for a MYC-associated zinc finger (MAZ) and another for MYC-MAX (MYC-associated factor X) complex (found in both K562 and NB4 cells).

Two other sites preceding the most upstream CAGE tags may bind EGR1 and ELF1 (Figure S2B). Each site constitutes a central motif in DNAse footprints obtained from CMP (mCD34), MEPs (CMK and K562 cells) or myelo-monocytic cells (CD14^+^ MNs, HL-60 and NB4). Both EGR1 and ELF1 can exert positive or negative regulatory effects on target gene expression in various myeloid lineages [79,80,81,82,83,84,85,86,87,88,89,90,91] so that either factor might inhibit full-length transcription of *NRAMP1* in non-expressing cells (see Section 2.3.1.6). Lastly, transcriptional activity at DHS F10 was independently reported in K562 cells [49] thus supporting a regulatory role of this element in myeloid cells.

##### 2.2.2.2. Correlating CAGE Signals and Other Marks of *NRAMP1* Expression in Myeloid Leukemias

Detailed examination of the elements depicted in Figure S2B is provided as Supplementary Text, [92,93,94]. In summary, CAGE tag clustering, histone acetylation marks and gene expression levels in K562 erythrocytic and THP1 monocyte-like cells support the presence of a negative determinant in *NRAMP1* intron 12. Activation of this element in progenitors of professional phagocytes (e.g., mCD34 cells/CMPs, MEPs and the APL NB4), and in blood cells derived from them by either undergoing divergent developmental pathway (e.g., MCs) or following subsequent differentiation (e.g., MDDCs), may restrict expression of full-length *NRAMP1*.

#### 2.2.3. Sites Showing Bidirectional CAGE Signals in *NRAMP1* Expressing Cells

As occurrence of low abundance, non-overlapping, bidirectional and divergent tag clusters on opposite DNA strands identifies enhancers in human cells [31,68], detecting such features in professional phagocytes can validate potential enhancer elements previously predicted in myelo-monocytic progenitors and MNs based on specific DNAse footprints, functional histone modifications and interactions with various TFs [11].

Myeloidome CAGE data thus support several determinants of *NRAMP1* expression previously suggested by ENCODE epigenetic data for various cell types corresponding to distinct stages of myelopoiesis, including (i) G-CSF mobilized CD34^+^ hematopoietic stem progenitor cells (HSPC) that correspond to common myeloid progenitors (CMP; mCD34); (ii) cell lines regarded as proxies to various downstream myeloid progenitors, such as CMK (megakaryoblast), K562 (erythroleukemia), HL-60 (promyelocytic acute myeloid leukemia, AML) and NB4 APL and iii) CD14^+^ MNs [11].

CAGE may also suggest candidate determinants not previously described, and additional cell types were considered in the present study to examine the specificity of potential regulatory determinants, including (i) non-hematopoietic cell types such as ESC (capable of multi-lineage differentiation) and human umbilical vein endothelial cells (HUVEC, a mesodermal cell type) and ii) various hematopoietic cell types, including: CD133^+^ HSC, CD34^+^ HSPCs, MDMs and CD15^+^ polymorphonuclear neutrophils (PMN).

Inspection of bidirectional CAGE clusters (center ±250 bp) [68] at *NRAMP1* locus indicates potential enhancers with different structures: three of them, located upstream of the gene, form part of an ensemble that resembles a super-enhancer (S-E) while the fourth cluster, closer to *NRAMP1* TSS, may rather represent a classical enhancer.

##### 2.2.3.1. Upstream Ubiquitous Regulatory Hub (DHS F12)

This CAGE cluster, found in all blood cell types albeit with variable intensity, is situated in the untranslated part of *CATIP* last exon [68]; it binds massive amounts of RNA Pol II even in non-hematopoietic cells (e.g., ESC and HUVEC; Figure 1, Figure S3). This element also coincides with a CpG island, a remarkable property since enhancers are usually CpG poor [18]. DHS F12 also corresponds to CTCF site B (Figure 1).

Modest peaks of K27ac and K4me3 marks decorate DHS F12 in different tissues in absence of *NRAMP1* expression [14] (Figures S3 and S12). Accordingly, this element might act as a RH influencing gene expression. Mobilization of DHS F12 in various lineages (together with a relatively similar intronic element, DHS F9, cf. Section 2.3.1.5), may explain at least partly why *NRAMP1* locus generally appears devoid of inhibitory histone marks, such as K9me3 and K27me3 (Figure S8).

**Figure 2:**
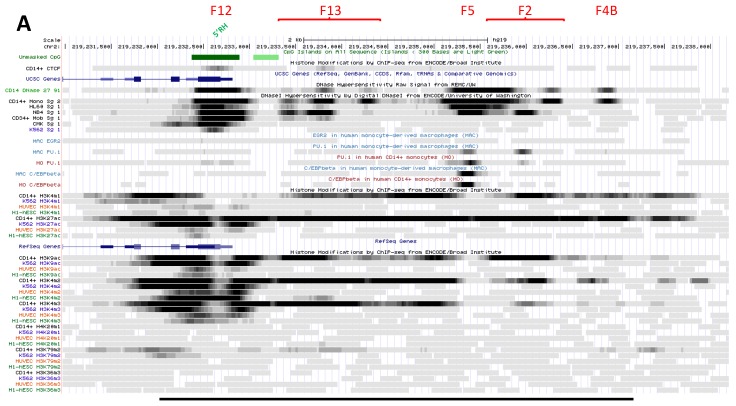

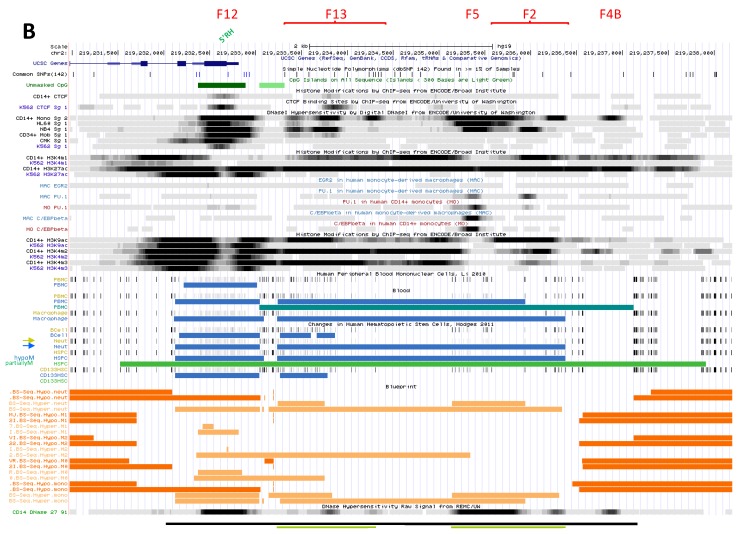
Epigenetic modifications at *NRAMP1* locus region ii, encompassing Dnase1 footprints F12/RH, F13, F5 and F2, and F4B (described in text sections: Section 2.2.3.1, Section 2.2.3.2, Section 2.2.3.3 and Section 2.3.2.2, respectively) part of a candidate super-enhancer domain (data from ENCODE consortium [9,10,78] or stated otherwise). (**A**) *From top to bottom*: chromosome 2 scale; CpG islands; CTCF sites in CD14^+^ MNs; UCSC gene descriptions; DNAse1 footprints in CD14^+^ MNs, HL-60, NB4, mCD34, CMK and K562 cells; EGR2, PU.1 and C/EBPb ChIP-seq data from MNs (MO) and MFs (MAC) [13]; H3K4me1 and H3K27ac ChIP-seq data from CD14^+^ MNs, K562 cells, HUVEC and ESC; RefSeq genes; H3K9ac, H3K4me2, H3K4me3, H4K20me1, H3K79me2 and H3K36me3 ChIP-seq data from CD14^+^ MNs, K562 cells, HUVEC and ESC; Black/grey symbols indicate location and intensity of CAGE signals detected in myeloid cells (cf. Figure 1, [68]). (**B**) From top to bottom: chromosome 2 scale; UCSC genes; common SNPs; CpG islands; CTCF ChIP-seq data for CD14^+^ MNs and K562 cells; DNAse1 footprints in CD14^+^ MNs, HL-60, NB4, mCD34, CMK and K562 cells; H3K4me1 and H3K27ac ChIP-seq data from CD14^+^ MNs, K562 cells; EGR2, PU.1 and C/EBPb ChIP-seq data from MO and MAC; H3K9ac, H3K4me2, H3K4me3 ChIP-seq data from CD14^+^ MNs and K562 cells; CpG hypomethylation data from PBMCs, MFs, B cells, Neutrophils, CD34^+^ HSPCs and CD133^+^ HSCs, as well as select Blueprint data (Neutrophils; M1, M2 and M0 MFs; monocytes) [14,15]; DNAse1 footprints in CD14^+^ MNs; Black/grey symbols indicate location and intensity of CAGE signals (cf. Figure 1); Green symbols highlight hypomethylated areas in MFs, neutrophils and/or CD34^+^ HSPCs.

F12 site is marked with similar histone 3 modifications in both CD14^+^ MNs and K562 cells [14,15], notably K9ac, K4me2 and K4me3; it is also flanked in 5′ with K79me2 (Figure 2A). Both ESC and HUVEC also display significant K4me2/3 and K9ac marks thus implying broad activity of this site, demonstrated by transcriptional activity in non-hematopoietic cells [49]. Most histone decorations surround F12 DHS, suggesting it may accommodate a combination of TFs; predicted TF binding sites (TFBS) include some for ETS factors, CREB, NF-KB, SP1, IRF and a potential pTF, FOXO1.

DNAse footprints show higher accessibility in myelo-monocytic precursors (mCD34 cells; HL-60 and NB4 cells), terminally differentiated cells (CD14^+^ MNs) and megakaryotcytic progenitors (CMK cells) compared to K562 erythrocytic precursors (Figure 2A). In contrast, K562 cells display predominant CTCF binding (Figure 2B) and association with various TFs as well as chromatin remodelling factors [78] that exert methyltransferase, demethylase, HAT, or chromatin modifying or reading activities, and with members of the pre-initiation complex (Figure S9).

The area spanning F12 site is globally unmethylated (Figure 2B) [14,15,95,96,97], hence compatible with CTCF binding [98] in all cell types studied, including HSC (Figure 2B). Thus F12 site is broadly accessible and it supports gene expression in hematopoietic cells.

Lastly in MDM (using M-CSF), F12 element appeared constitutively bound by STAT1, an association that was little affected by IFN-g priming and/or further stimulation with LPS [99] (Figure S10). Potential interaction with IRF1 was also noted. F12 element thus seems to principally regulate *NRAMP1* expression in basal conditions.

##### 2.2.3.2. Intermediate Cluster of Myeloid-Specific TF Binding Sites (DHS F13)

CAGE data at DHS F13 show transcription mostly restricted to myeloid cell types, including MNs, granulocytes (GNs) and some CD34^+^ HSPCs; RNA Pol II activity was also detected in MC though these cells do not express *NRAMP1* [68] (Figure S3).

DNAse footprinting shows two major accessible sites in CD14^+^ MNs and two bording them but less apparent. The two first sites are more accessible in NB4 cells than in HL-60 and mCD34 cells, implying myelo-monocytic regulation (Figure 2A). These sites bind RNA Pol II strongly in NB4 cells, and more weakly in HL-60 and K562 cells as well as ESC (Figure S3).

In K562, NB4 and ESC the F13 area interacts with CTCF (Figure S3; corresponds to site F, Figure 1) as well as some cohesin subunits (in HepG2 cells); it binds few TFs (GABP, PU.1) in HSPCs or MYC-MAX in NB4 cells [78] (Figure S9). Interactions with both CTCF and hematopoietic TFs thus imply lineage-specific regulatory roles for this cluster of DHS.

Histone marking at F13 is specific of myelo-monocytic cells and absent in K562 erythrocytic precursors (Figure 2A). Both K9ac and K27ac strongly mark the area in CD14^+^ MNs, as well as K4me1/2/3 (vs. traces of K79me2; Figure 2A). Nucleosome density suggests focal TF binding in CD14^+^ MNs while predicted BS include C/EBP, FOX, SP and IRF factors.

DNA hypomethylation at the two first sites shows differences between MFs and peripheral blood cell types (PMNs, CD34^+^ HSPCs) vs. CD133^+^ HSCs from umbilical cord blood (Figure 2B) [14,15]. Data thus suggest myeloid regulation of F13 area may be initiated around the HSPC stage and maintained through the GMP stage and in mature myelo-monocytic cell types (MNs and GNs) [100].

F13 forms thus a complex DHS whose properties differ from upstream neighbor site F12. Their combined activation may confer strong transcriptional activity (F12) and narrow cell-type specificity (F13), which are both required to switch on *NRAMP1* expression specifically in precursors of PMNs, MNs and MFs; in addition, F13 may confer responsiveness to immuno-modulatory signals.

##### 2.2.3.3. Complex Cluster of Myelomocytic-Specific Binding Sites (DHS F5-F2)

Contiguous CAGE clusters comprise several DNAse 1 footprints spanning adjacent areas previously described (F5, F2) [11] and close to a third, downstream DHS (F4B) not matched by significant CAGE signal (cf. Section 2.3.2.2; Figure S3). Up to six DNAse footprints suggested by uniform peaks may be juxtaposed along a 1.8 kb segment thus delineating another complex cluster of TFBS (F5-F2 area).

The first site is part of a strong CAGE tag cluster found in MNs and PMNs (Figure S3); in both CD14^+^ MNs and MDMs the area binds TFs key to the myelo-monocytic fate: PU.1 and C/EBPb (Figure 2A) [13,25]. The corresponding DNAse footprint is present in both HL-60 and NB4 promyelocytic cells: it represents a major binding site for RNA Pol II in the former (Figure S3) while in the latter, it binds TF MYC [11].

In CD14^+^ MNs both K9ac and K27ac surround this site as well as K4me1/2/3 (Figure 2A), suggesting full activation in mature mononuclear phagocytes and consistent with binding of several TFs (including STAT3 for instance) and associated activities (such as cohesin subunit, Figure S9). TFBS predicted in this area include YY1, FOX, IRF, STAT, C/EBP, NF-IL3, NRs, SMAD and PU.1.

In MDMs, F5 area demonstrated little constitutive association with STAT1 [99]; binding increased with IFN-g priming and/or activation with LPS, as well as IRF1 recruitment (Figure S10). The data suggest a key role of element F5 to regulate *NRAMP1* expression in response to pro-inflammatory signals, whereas C/EBPb and PU.1 may control F5 basal activity.

Next, there are two to three footprints part of F2 area, which shows moderate CAGE tag clustering in both MNs and GNs (Figure S3). These footprints are found in AMLs (the most 5′ in HL-60 cells, and the others in NB4); the first is present in CD14^+^ MNs as well, weakly decorated by K9ac/27ac and K4me1/2/3 marks (apparently flanking the upstream footprint F5; Figure 2A).

The second footprint corresponds to a candidate PU.1 binding site (detected in HSPCs, MDMs and more weakly in MNs) and it is intensely marked with modified histone (Figure 2A). This site may form the 3′ boundary of a cluster of BS for myelo-monocytic specific TFs (such as C/EBPb and MAFK) and allow the recruitment of RNA Pol II and HDAC activities (e.g., HDAC2; Figure S9).

The last footprint in the F2 area is present in CD14^+^ MNs only; it sits at the 5′ boundary of an area flanked on both sides by histone acetylation and methylation marks; it may thus bind several TFs and co-factors in these cells (Figure 2A) as suggested by predicted TFBS (e.g., FOX, C/EBP, ETS, POU and PPARg).

The chromosomal segment encompassing F5 and F2 areas shows extensive hypomethylation in MFs, PMNs and HSPCs (Figure 2B), an observation consistent with prevalent TF binding and active histone marking in professional phagocytes, both in basal and inflammatory conditions (Figure 2A).

##### 2.2.3.4. Candidate Super-Enhancer Domain

The cluster of footprints F12, F13, F5 and F2 covers a region that is highly transcribed in professional phagocytes (Figure S3) and abundantly marked in CD14^+^ MNs with histone modifications that support transcriptional activity (Figure 2A). Accordingly, this region matches an S-E domain predicted by profiling K27ac ChIP-seq signals in the interactive database DBsuper [101] (Figure S11). Integrating ChIP-seq signals for additional trans-factors (such as Mediator complex, BRD4 and RNA Pol II) and active histone marks (e.g., K4me2/3) may strengthen such S-E predictions [102].

The proposition that *NRAMP1* upstream area spanning F12-F2 DHSs, perhaps including also F4B (cf. Section 2.3.2.2), forms an S-E in CD14^+^ MNs is supported by comparing chromatin marking intensity of this domain with a predicted “classic” enhancer (see Section 2.2.3.5): the candidate S-E shows, on a broader scale, stronger deposition of K27ac and K4me1, PU.1 TF binding and RNA Pol II activity (Figure S11). Collectively, high level transcriptional activity and chromatin reorganization ability imply this 5 kb area may form a major regulatory domain whose properties seem compatible with the functional definition of an S-E [20,39,40,41,102].

The 5′ element F12 is the most intensely transcribed in phagocytes; it corresponds to a CpG island marked by K27ac, K9ac and K4me3 in various tissues (Figure S12) and may act as a RH (F12/5′ RH, Section 2.2.3.1). In contrast, 3′ elements are decorated with histone modifications only in cells that express *NRAMP1* at high level; among these, F5 element binds STAT1 and IRF1 in response to infection stimuli (Figure S10). Data thus distinguish two functional areas in the proposed S-E domain whose combined activation may induce *NRAMP1* expression specifically in mature phagocytes and allow immuno-modulation.

##### 2.2.3.5. Simple Myelo-Monocytic Proximal Enhancer (DHS F7)

**Figure 3:**
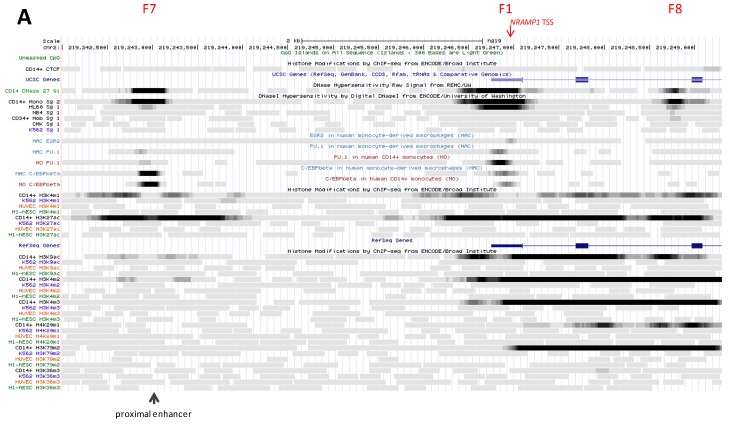

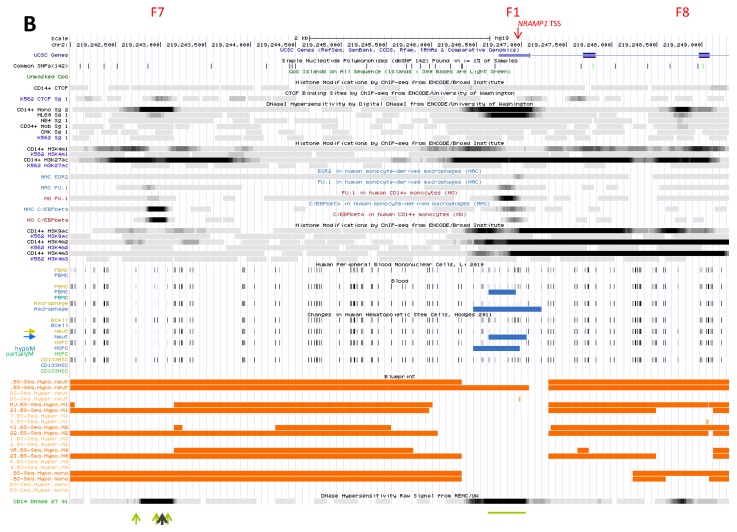
Epigenetic modifications at *NRAMP1* locus region iii, encompassing Dnase1 footprints F7, F1 and F8 (described in text Section 2.2.3.5, Section 2.3.1.3 and Section 2.2.3.6, respectively). (**A**), (**B**) same as Figure 2.

CAGE tag clustering at element F7 indicates moderate activity compared to the proposed S-E (Figure S11) but similar specificity, being detected essentially in mature phagocytes (Figure S4). DHS F7 typifies *NRAMP1* expressing cells, such as CD14^+^ MNs (Figure 3A) and HL-60 cells wherein RNA.

Pol II binds this site (Figure S4). F7 interacts with C/EBPb in mononuclear phagocytes (Figure 3A) while it seems associated with CTCF insulator in erythrocytic precursors (K562; Figure S4).

Regarding histone decoration, few marks are observed in CD14^+^ MNs and none in K562 cells. Histone marks in MNs indicate low level of transcriptional activity, with predominant deposition of K27ac and by decreasing abundance K4me1, K4me2 and traces only of K9ac. Histone marks border the DHS, suggesting several TFs may bind F7 element in MNs; predicted TFBS include USF, MAF and SMAD. The area also shows DNA hypomethylation in MFs, PMNs and HSPCs compared to HSCs (Figure 3B), thus further supporting regulatory activity in professional phagocytes.

In MDMs exposed to hypoxia (for 4–8 h) DHS F7 represents the strongest ChIP-seq signal specific for HIF1a and HIF2a [103] (Figure S10). F7 association with STAT1 was also noted after stimulation with LPS, as well as possible binding of IRF1 in response to IFN-g priming and further activation with LPS [99] (Figure S10). Hence, in addition to constitutive association with C/EBPb, F7 element may bind TFs that regulate *NRAMP1* expression in response to various infection stimuli.

Current data suggest DHS F7 is mobilized specifically during myelo-monocytic differentiation. Transcriptional activation occurs in mature cells and it appears possible that full activation requires further cooperation with signal-dependent TFs (e.g., Fos/AP1, HIFs, STAT1 and IRF1).

##### 2.2.3.6. DNase Footprint in *NRAMP1* Intron 3 (DHS F8)

CAGE signal is moderate at site F8 in MNs and MDMs (Figure S4), including LPS-treated and influenza virus infected MDMs [16,17]. The corresponding DHS was revealed in CD14^+^ MNs and HL-60 cells, and to lesser extent in NB4 and mCD34 cells (Figure 3A). Little if any bound RNA Pol II was detected in HL-60 only (Figure S4) while AP1-type or MYC TF, and chromatin modifier such as BRG1 may associate with this site (Figure S9).

The area is decorated with various histone 3 acetylation and methylation marks, K27ac and K4me2/3 for the most intense (Figure 3A). No histone marks or CTCF binding was detected in K562 cells. The area shows limited DNA methylation and no evidence of hypomethylation (Figure 3B). The paucity of TF binding this element, e.g., weak association with AP-1 (Figure S9) and STAT1 (Figure S10), may suggest myelo-monocytic specificity and/or stimulus-dependency; NRs, SMAD and NRF2 represent candidates based on predicted TFBS.

The potential regulatory determinants presented in Section 2.2.3., which show bidirectional CAGE tag clustering, were not recognized as such initially [16,17] presumably because *NRAMP1* transcriptional signals are relatively weak on a genome scale. Yet, all these elements are decorated with K9ac in CD14^+^ MNs, which supports enhancer-type transcriptional activity [44,45]. This interpretation is supported by prevalent deposition of K4me3 mark in CD14^+^ MNs and binding of RNA Pol II in committed myelo-monocytic cells (HL-60). Accordingly, these sites are expected to support *NRAMP1* expression in MNs although some appear little active, such as DHSs F7 (5′ of TSS) and F8 (in intron 3). These two sites may require either lineage- or signal-dependent collaborating TF(s) to become fully activated (such as HIFas, STAT1, IRF1 or AP-1).

### 2.3. Other Candidate NRAMP1 Regulatory Elements

Combining CAGE results with epigenetic marks suggesting functional activity allowed the identification of strong candidate regulatory elements in phagocytes (Section 2.2.3.). Additional CAGE signals showed clusters of tags that reflected either forward or reverse transcription only. Such eRNA data indicate elements with low transcriptional activity that may reflect either open chromatin or bookmarking for later activation [49]. In these cases, seeking additional marks of activity is crucial to predict regulatory potential.

#### 2.3.1. Determinants Partially CAGE Positive

Additional candidate regulatory elements were tentatively delineated by aggregating results from DNAse footprints, ChIP-seq data for specific histone marks and/or TFs, including RNA Pol II and CTCF, and DNA methylation status (from consortia: ENCODE [9,10,78]; NIH Roadmap [14]; EU Blueprint epigenome [15]), in addition to evidence of forward or reverse CAGE data (RIKEN FANTOM5 [16,17]).

##### 2.3.1.1. Upstream Footprint That Overlaps *CATIP* Exon V (DHS F6)

**Figure 4:**
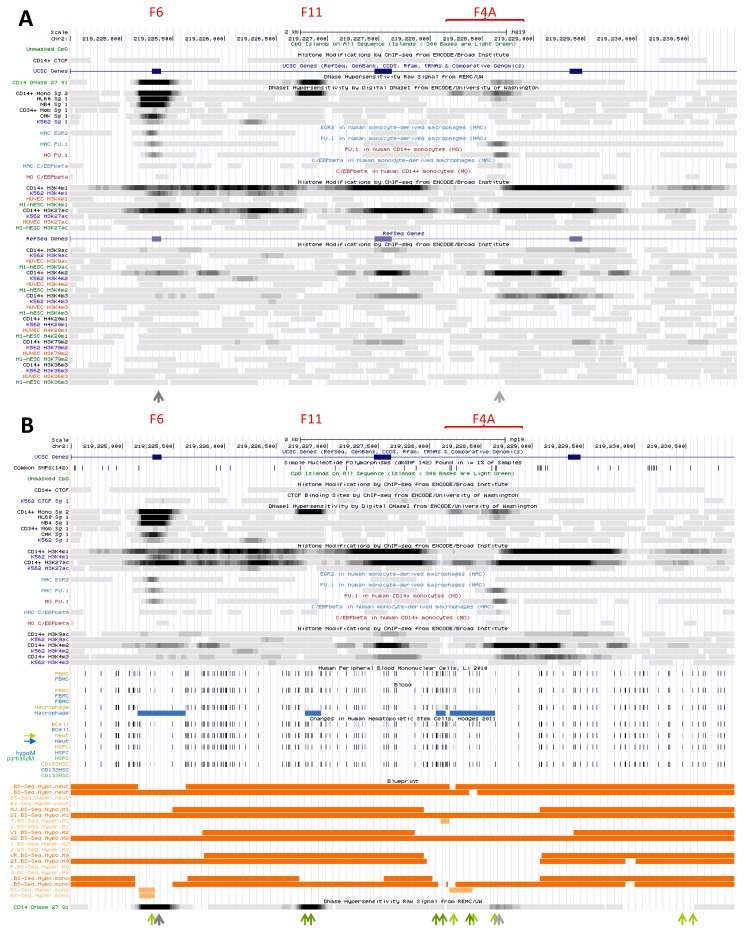
Epigenetic modifications at *NRAMP1* locus region i, encompassing Dnase1 footprints F6, F11 and F4A (described in text Section 2.3.1.1, Section 2.3.2.1 and Section 2.3.1.2, respectively). (**A**), (**B**) same as Figure 2.

This most upstream forward CAGE signal, detected in MNs and PMNs (Figure S5), matches a strong DHS (F6) in myelo-monocytic cells (Figure 4A); transcription was also observed in LPS-treated and Influenza virus-infected MDMs [16,17]. F6 element was detected in CD14^+^ MNs, in HL-60 cells as well as in NB4 APL (Figure 4A), and RNA Pol II strongly binds this site in undifferentiated HL-60 cells (Figure S5). Lighter DNAse footprinting was revealed in mCD34 cells (weak) and precursors of the megakaryocytic (CMK) and erythrocytic (K562) pathways (Figure 4A). Accordingly, this site is expected to be mobilized early during myelopoiesis.

Several TFs bound element F6 in K562 cells, such as EGR1, ELF1, MAZ (Figure S9), which are known to regulate gene expression in myelo-monocytic cells. These same TFs also bind the 3′ regulatory site in *NRAMP1* intron 12 (F10, cf. Section 2.3.1.6) in K562 cells. Predicted TFBS at F6 include NRs, RUNX, EGR, ETS and NRF2.

F6 and F10 sites are distant of ~33 kb but within CTCF boundaries of *NRAMP1* regulatory domain. Both associate with RCOR1 in K562 (Figure S9), which suggests a potential mechanism for co-repression of *NRAMP1*, as well as with EGR2 in MDMs. In addition, F6 site binds PU.1 in MNs and MDMs (Figure 4A).

Histone marks deposited at site F6 support little transcriptional activity in CD14^+^ MNs. Traces of K9ac or K4me3 decoration were detected together with K27ac and K4me1 marks as well as low level of K4me2 (Figure 4A). In K562 cells, only little K4me1 was found, implying reduced priming in erythrocytic progenitors. Yet, since TFs important for myelo-monopoiesis can bind F6 site in K562 cells (Figure S9), wherein CTCF associates with sites F12, F13 and F7 (cf. Section 2.2.3.1, Section 2.2.3.2 and Section 2.2.3.5), data therefore imply specific mobilization of F6 in K562 as well.

Regarding DNA methylation, few CpGs are present in the area but reduced methylation at F6 site was detected in MFs, PMNs and HSPCs, supporting activation of this element during myelo-monocytic differentiation (Figure 4B).

Modest interaction with STAT1 was also detected and seemingly constitutive (Figure S10) [99]. This observation correlates with low level PU.1 binding in MNs and MDMs, as well as EGR2 binding in MDMs, suggesting several factors may cooperate at F6 element to control *NRAMP1* basal expression level.

The signals detected imply F6 site binds TFs important for myelo-monocytic fate. F6 is mobilized early during myelopoiesis and its functional status differs between myelo-monocytic and erythrocytic lineages. However, this regulatory element is not fully active in mature cells. It is possible that full transcriptional activation of this element depends on recruitment of lineage- and/or signal-dependent TF(s).

##### 2.3.1.2. Pair of DNase1 Footprints in *CATIP* Intron 6 (DHS F4A)

CAGE revealed only forward signal in this area, with low abundance transcript tags observed in MNs and PMNs essentially (Figure S5). The area comprises two DHSs of medium intensity (previously reported as F4A); the upstream footprint was detected only in CD14^+^ MNs while that in 3′ is shared by HL-60 cells (Figure 4A) wherein it demonstrates little RNA Pol II binding (Figure S5). The 3′ footprint matches a PU.1 binding site evidenced in both MNs and MDMs (Figure 4A), implying that weak eRNA production at F4A does not merely indicate tracking of upstream enhancer-bound transcriptional complex moving towards *NRAMP1* TSS, for instance [18]. The area also contains few CpGs, some of which are hypomethylated in mature phagocytes vs. HSCs, with differences between MDMs and PMNs (Figure 4B).

Marked histone decoration is myelo-monocytic specific (Figure 4A). Marks essentially flank in 3′ or overlap the downstream footprint, including both acetylated (predominantly K27ac) and methylated (K4me1/2) histones. Lighter deposition of K4me3 and K9ac is consistent with low level transcriptional activity. Still, histone decoration pattern may suggest broad nucleosomal displacement and several TFBS were predicted in the area (e.g., C/EBP, NR, POU, FOX, ETS, GFI, NF-IL3).

These data point a regulatory area that is bound by PU.1. F4A dual determinant is specific of the myelo-monocytic lineage, apparently activated late in the differentiation program and demonstrating little transcriptional activity in blood phagocytes. The nature of the TF(s) collaborating with PU.1 and of the potential signals required to fully activate this element remain to be investigated, using microbial compounds for instance.

##### 2.3.1.3. *NRAMP1* TSS Area (DHS F1)

This site is the strongest CAGE tag cluster observed in blood cells, predominantly in phagocytes, including MN subpopulations (peak value at TSS 272 ± 73), MDMs (49.5), pDCs (12), and MDDCs (5.5) as well as GNs (neutrophils, 395; eosinophils, 263; basophils, 134) and other cell types such as HSPCs (29), GMP (8.5) and some lymphocytes (B and NK, both 11; Figure 1 and Figure S4). These data demonstrate *NRAMP1* TSS is transcribed at high level in microbicidal phagocytes. CAGE tag clusters map within 60 nucleotides downstream of the TSSs previously identified [76,104], suggesting they may indicate stalled RNA Pol II [18]. F1 constitutes a major DHS in both CD14^+^ MNs and HL-60 cells (Figure 3A).

F1 footprint overlaps binding sites for the TFs C/EBPb and PU.1, detected in both MNs and MDMs, and EGR2 (MDMs; Figure 3A). RNA Pol II associates strongly with this area in HL-60 cells (Figure S4). In CD14^+^ MNs, F1 site is heavily decorated with K27ac and surrounded by K9ac marks as well; K4me2 is the dominant H3 methylation mark detected together with K4me3 while K4me1 is less abundant, consistent with a pattern generally observed at TSSs. In contrast, the area lacks histone marks in K562 cells. Apparent nucleosomal displacement from the central area in MNs is compatible with binding of several TFs (e.g., TFBS predicted for IRF, FOX, RUNX, CREB, NF-KB, PU.1, ETS, SP1, GFI, GKLF, NR, C/EBP, NF-Y, NF-AT and NF-IL3 within F1 area).

F1 also corresponds to moderate HIFa ChIP-seq signals that overlap the polymorphic TG repeat known to contain two sites for the HIF-1α/ARNT heterodimer, whose binding stimulated *NRAMP1* expression in THP-1 MFs activated by pathogen or proinflammatory signals [105]. ChIP-seq data in response to hypoxia suggested both HIFas may bind *NRAMP1* promoter, while IL-10 pre-treatment seemed to reduce HIFas binding [103] (Figure S10). It will be interesting to detail the interplay of hypoxia, infection and HIF-associated factors in regulating *NRAMP1* expression.

F1 element interacts, similarly to F12, with STAT1 in MFs both constitutively and in response to IFN-g priming and/or activation with LPS; possible association with IRF1 was also noted in IFN-g primed MFs further stimulated with LPS [99] (Figure S10). The data suggest that both factors may cooperate with other TFs bound to F1 area (e.g., C/EBPs, PU.1, EGR2, HIFas) to regulate *NRAMP1* expression in inflammatory conditions.

Lastly, the region comprises several CpGs, the majority of which are hypomethylated in MFs and select blood cells (HSPCs and PMNs) compared to HSCs and lymphocytes (Figure 3B). Together the data demonstrate specific activation of *NRAMP1* TSS in the maturation pathways leading to professional phagocytes.

##### 2.3.1.4. Putative Element in *NRAMP1* Intron 5 (DHS F14)

**Figure 5:**
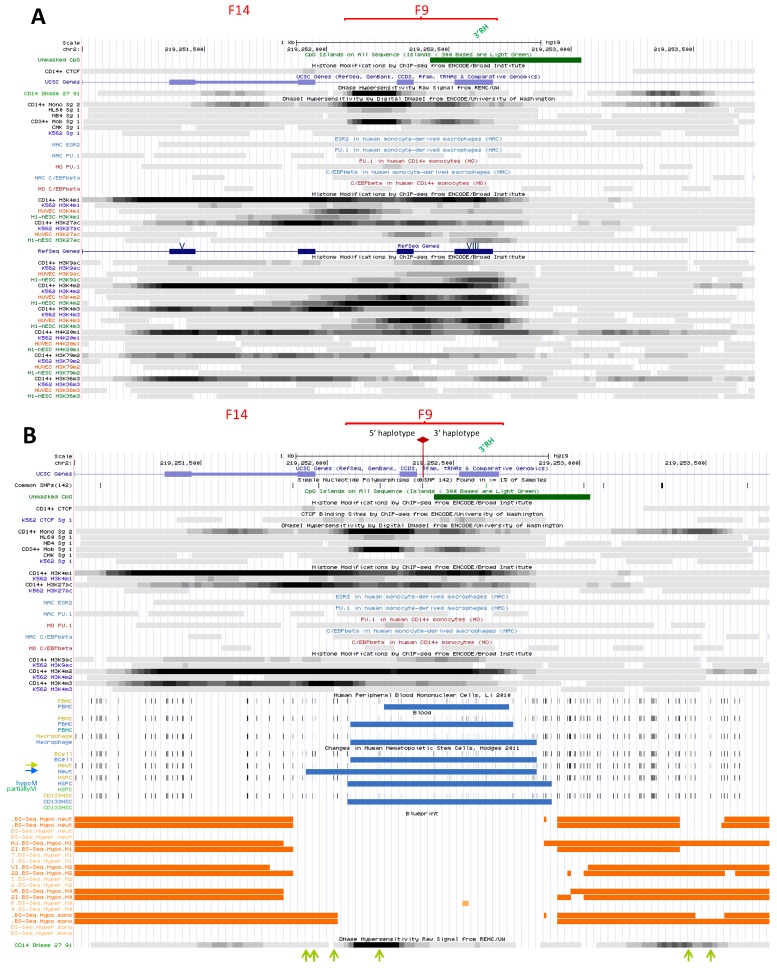
Epigenetic modifications at *NRAMP1* locus region iv, encompassing Dnase1 footprints F14 and F9 (described in text sections: Section 2.3.1.4 and Section 2.3.1.5, respectively). (**A**) same as Figure 2. (**B**) same as Figure 2; the boundary of *NRAMP1* 5′ and 3′ haplotypes is indicated (cf Section 2.3.5).

This site, located between exons V and VI (Figure 5A), yielded both forward and reverse CAGE signals shared by most myelo-monocytic cell types, but relatively weak and convergent (Figure S6). The area matches a DNAse footprint not previously described (F14) that is found only in CD14^+^ MNs, and which may bind TFs of the AP1 family (among others, Figure S9). Histone marking includes predominantly K4me1/2 and weaker decoration by K4me3, K27ac as well as traces of K9ac; it also indicates ORF transcription (H4K20me1, H3K79me2 and H3K36me3; Figure 5A).

F14 area shows few CpGs but no sign of hypomethylation in the cell populations studied (Figure 5B). A possible contribution of this site to regulate *NRAMP1* expression may be to confer myelo-monocytic specificity to the downstream candidate RH (see below, Section 2.3.1.5).

##### 2.3.1.5. Candidate Regulatory Hub within *NRAMP1* ORF (DHS F9)

A faint CAGE tag cluster preceding exon VII was detected in myelo-monocytic cells (Figure S6). It corresponds to a DNAse footprint previously described (F9), showing high accessibility in CD14^+^ MNs and mCD34 cells, and to lesser extent in HL-60 (Figure 5A). Another weaker, monocytic footprint locates just upstream of exon VIII, and a CpG island overlaps both the second footprint and exon VIII.

A number of TFs have been found interacting with element F9, albeit not in myeloid cells. These include members of AP1, FOX and HNF families as well as general factors such as YY1, SP1 and RXRa (Figure S9); TFBS for GFI, SOX, LMO, PU.1, NRs, SP3 and AP2 were also predicted. F9 area was also found associated with both members of the transcription preinitiation complex and some methyl-transferase and HAT activities (Figure S9), suggesting element F9 may act as an intronic RH with chromatin remodeling activity.

Regarding histone decorations, F9 area is mainly marked in MNs, but not in K562 erythrocytic progenitors, with K4me2 and K4me1 and to a lesser extent with K27ac and K4me3. Traces of K9ac co-localize with the downstream footprint (Figure 5A). Notably, the area is also labelled with several histone marks in both ESC and HUVEC (Figure 5A).

In fact, the area spanning exons VI-VIII is marked by K4me1 in most cell types and tissues, a subset of which also exhibits dual decoration with K4me3 and K27ac [14] (Figure S12). Data suggest that, similarly to the CpG island/RH localized in 5′ (F12, Section 2.2.3.1), F9 area might serve as a RH independent of *NRAMP1* expression. Supporting this proposition, strong binding of RNA Pol II was detected at F9/3′ RH CpG island in ESC (Figure S6).

Nonetheless both candidate RHs F12 and F9 also display negative marks such as K27me3 and K9me3, in ESC and HUVEC for instance, with K9me3 dominating at 5′ RH/F12 and K27me3 more abundant at F9/3′ RH (Figure S12). Accordingly, a balance of positive/negative marks may allow dynamic regulation at these sites.

F9 area and the overlapping CpG island demonstrate generalized hypomethylation in both mature phagocytes and HSCs; F9 site is preceded by few CpGs specifically hypomethylated in MFs, PMNs and HSPCs (Figure 5B).

It thus seems conceivable that the F14-F9 region constitutes a RH with chromatin remodeling activity that may regulate myelo-monocytic gene expression. Low level histone acetylation in mononuclear phagocytes suggests F14-F9 elements may require further signaling (either lineage-specific or in response to external stimulus) to be fully activated. Further analyses will be necessary to clarify the putative role of these candidate regulatory sites.

##### 2.3.1.6. Negative Regulatory Element in *NRAMP1* Intron 12 (DHS F10)

**Figure 6:**
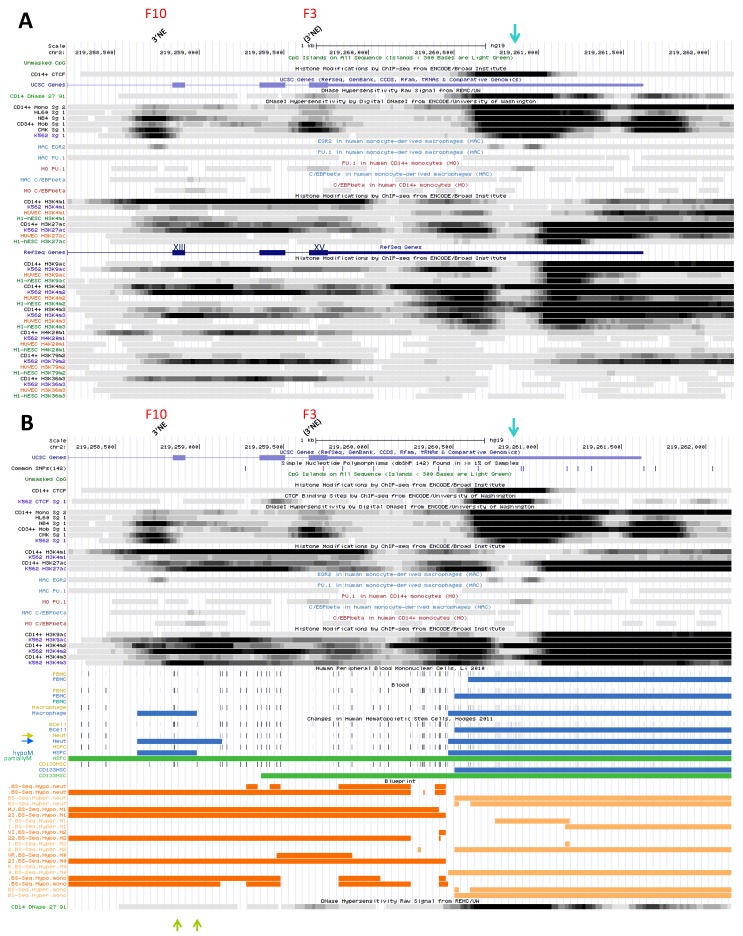
Epigenetic modifications at *NRAMP1* locus region v, encompassing Dnase1 footprints F10 and F3 (described in text Section 2.3.1.6 and Section 2.3.1.7). (**A**), (**B**) same as Figure 2.

CAGE data at this site suggest an alternative, 3′ TSS in cell types expressing little or no full-length *NRAMP1* (cf. Section 2.2.2.1). The corresponding footprint F10 was found in various myeloid cells but interestingly, predominantly in precursors of the megakaryocytic/erythrocytic lineages (CMK, K562) as well as some GMP proxy (NB4) i.e., cells not expressing *NRAMP1* (Figure 6A). F10 area is comparatively less sensitive to DNAse digestion in cells predisposed to or that express *NRAMP1*, i.e., CD14^+^ MNs, HL-60 and mCD34 cells.

Remarkably, RNA Pol II binds to element F10 primarily in cells that do not express *NRAMP1* (K562, NB4) while HL-60 cells display scattered interactions (Figure S7). F10 area is also decorated in K562 cells with histone marks of transcriptional activity (K9ac, K4me2/me3 and notably, K79me2, mostly on the 3′ side; Figure 6A). In contrast, CD14^+^ MNs exhibit K4me2 mark, low amount of K4me3 but no evidence of K9ac deposition, implying lower activity level, despite abundant K4me1 mark, moderate K27ac decoration as well as evidence of full-length ORF transcription (H4K20me1, H3K36me3).

A variety of TFs bind F10 element in K562 (cf. Section 2.2.2.1) and other cells (Figure S9), including those found associated with the 5′ element F6 (cf. Section 2.3.1.1), as well as regulatory factors such as the HAT p300. Predicted TFBS include NRs, RUNX, GKLF, SP1, PU.1, NF-Y and NF-KB. In MDM, EGR2 binding to F10 supports a possible role in regulating *NRAMP1* expression. Yet, low level hypomethylation at this site, in mature phagocytes and HSPCs (Figure 6B), implies such a role may be modest.

The 3′ element F10 stands apart from other *NRAMP1* candidate regulatory sites because it is transcribed in absence of gene expression. This indicates element F10 may exert a negative role that limits *NRAMP1* expression in some myeloid lineages. It remains possible F10 element modulates *NRAMP1* expression in the myelo-monocytic lineage, perhaps by recruiting stimuli-responsive TFs.

##### 2.3.1.7. Potential Negative Element Overlapping *NRAMP1* Exon XV (DHS F3)

This partial CAGE signal shows preferential forward transcription in *NRAMP1* expressing blood cells (PMNs, CD14^+^ MNs) while reverse transcription seems favored in non-expressing cells such as AMLs [68].

The bidirectionally transcribed area overlaps the previously described DHS F3, which spans *NRAMP1* exon XV and was observed in models of early stages of myelopoiesis, such as megakaryocytic precursors (CMK) and CMP (mCD34 cells). Lower accessibility of this area is observed in other myelo-monocytic cell types (CD14^+^ MNs, HL-60 and NB4) but not in the erythrocytic lineage (K562 cells; Figure 6A). Element F3 apparently binds the TF ELF1 in K562 cells, as observed for F6 (Section 2.3.1.1) and F10 (Section 2.3.1.6).

F3 decoration with modified histones is more extensive in K562 cells compared to CD14^+^ MNs, suggesting that, similarly to F10 site, F3 may regulate gene activity in the erythrocytic lineage (Figure 6A). Acetylation, K4me2/3 and K79me2 marks are more abundant in K562 cells vs. MNs whereas the contrary is observed for K4me1, implying that elements F10 and F3 may recruit lineage-specific factors (erythrocytic vs. myelo-monocytic).

Lastly, F3 area lacks CpG dinucleotides and provides no evidence of hypomethylation (Figure 6B). Together the data imply that element F3 may exert different regulatory roles on *NRAMP1* expression, depending on the TFs present in divergent myeloid lineages.

#### 2.3.2. CAGE Negative Determinants

Two additional elements in the 5′ part of *NRAMP1* locus that correspond to DHSs in MNs however display very low level CAGE signals in blood cells.

##### 2.3.2.1. Seemingly Primed 5′ Element (DHS F11)

The DNAse footprint F11 matches a potential FOSL1 BS evidenced in K562 erythrocytic cells (Figure S9). In MNs the area is little decorated with modified histone, being bordered by K4me1 and K27ac marks that suggest functional priming (Figure 4A). Since the DHS itself is not decorated, it is logical to assume this element recruited several lineage-dependent TFs and/or co-factors; a number of TFBS were predicted in this area (e.g., ELF, AP-1, NRs, NF-KB, C/EBP and GKLF). Transcriptional activation may depend on signal-dependent TFs, whose binding could stimulate further decoration of the area.

This suggestion is supported by observing that despite high prevalence of CpG dinucleotides the area shows sign of hypomethylation in MFs only and not in HSPCs or PMNs (Figure 4B). Interestingly, differences between MF and PMN status of CpG methylation were also observed at the neighbor site F4A (Section 2.3.1.2). Examination of the chromatin from professional phagocytes further stimulated by microbial and/or immunological signals may provide clues to the regulatory mechanisms controlling this element.

##### 2.3.2.2. Potential Element at the 3′ Boundary of *NRAMP1* Candidate S-E (DHS F4B)

MN footprint F4B is decorated by K27ac and K4me1 marks and labeled in 5′ by K4me2, but it lacks strong signal that would indicate robust transcriptional activity (Figure 2). Histone data thus appear consistent with virtual lack of CAGE tag cluster in this area (Figure S3). Traces of K9ac, K4me3 and K79me2 marks around footprint F4B may support the view that this site constitutes the 3′ boundary of the proposed S-E domain (Section 2.2.3.4). Though no TF was found associated with DHS F4B several potential TFBSs (HNF, SOX, PU.1, NF-AT, USF, NRs) were predicted in the area, which is devoid of CpGs (Figure 2B).

Further probing of myelo-monocytic TF-chromatin interactions in mature phagocytes should help identify trans-factors that recognize F4B site, perhaps considering environmental conditions that may enhance *NRAMP1* S-E mobilization.

Mining *NRAMP1* locus for potential transcriptional regulatory elements identified four new candidates (F11-F14). F12, F5, F7 and F1 represent together with F6 the most accessible sites among the 14 candidate transcriptional regulatory elements that together demonstrate functional variety which suggests they are targets for combinatory signals that relate to lineage-, tissue-, and stimulus-specificity; transcriptional activation, which maybe demonstrated, potential or simply primed for subsequent stimulation; and impact on gene expression, including positive or inhibitory effects.

#### 2.3.3. CTCF Sites at *NRAMP1* Locus

CTCF binding sites are frequently associated with cohesin complex and topologically associating domain (TAD) formation can exert major regulatory roles during hematopoiesis [60,106]. Each CTCF site of *NRAMP1* locus has a specific profile of epigenetic marks, depending on DHS intensity, abundance of histone marking and variety of TF bound, which suggest distinct contributions to regulate gene expression.

Of note, site B (i.e., F12/5′RH) was the sole that did not bind components of the cohesin complex (e.g., SMC3, RAD21, Figure S9). Besides, intensity of histone decoration of CTCF sites proximal to *NRAMP1* (A, B, C, F) differ among hematopoietic cells and HUVEC or ESC, as well as the level of *CTDSP1* mRNA, which may suggest some possible correlation (Figures S15 and S16). Additional epigenetic differences distinguish cells expressing *NRAMP1* from others hematopoietic cells.

Site A, located within *NRAMP1* 3′ UTR forms the locus downstream boundary and shows abundant CTCF binding in various cells (Figure 1). In blood cells this site corresponds to a strong DHS that is decorated with K27ac, K9ac and K4me3 (Figures S15A and S16A); it is also flanked on both sides by heavy K4me1 marks, while histone 3 modifications typical of transcriptional activation cover the downstream area (Figure S15). In HUVEC and ESC, CTCF_A displays reduced DNAse sensitivity and histone decorations (Figure S16), correlating with lower expression level of *CTDSP1* (K36me3 and RNA-seq data). Epigenetic status of CTCF_A thus varies with the level of expression of surrounding genes, implying it may influence local transcriptional activity.

CTCF_A demonstrated similar propensity to form upstream loops in both K562 and MCF-7 cells (cf. Section 2.1). Assuming such looping pattern is common to myelo-monocytic cells implies CTCF_A would contribute to insulate *NRAMP1* gene (Figures S1B and S17). ENCODE data show this site may bind a variety of TFs, including STAT1 (Figure S9), whose interaction appeared constitutive while IRF1 binding seemed induced by IFN-g [99] (Figure S10). These data together suggest a possible contribution of CTCF_A to regulate *NRAMP1* expression.

Genome-wide association of SNPs with *NRAMP1* expression level in M2 MFs in various conditions pointed at CTCF_A area, revealing a statistically significant eQTL. Homozygous variation at rs17229016 correlated with low level *NRAMP1* expression independent of infection [54]. In another study, this same eQTL was also associated with reduced *NRAMP1* mRNA level both in basal conditions and 6 h post-infection with Influenza A virus, and it was found in linkage disequilibrium with surrounding SNPs within a ~2 kb fragment [107]. Genetic variation thus links CTCF_A area with reduced *NRAMP1* expression, corroborating epigenetic data that suggest a possible regulatory role.

Site B is located upstream of *NRAMP1*, at the 3′ end of the *CATIP* gene. This site shows little CTCF binding (Figure 1) and matches a ubiquitous DHS, F12/5′ RH that is stronger in myelo-monocytic cells (Figures S15 and S16). Accordingly, histone decoration correlates with *NRAMP1* expression level (judged by K36me3 and RNA-seq), including K4me1, K27/9ac and K4me3 notably upstream of this site. CTCF_B thus likely contributes to control *NRAMP1* expression, consistent with the variety of TFs constitutively associated with this site (Figure S9), including STAT1 in MFs [99] (Figure S10).

Site C also displays moderate CTCF binding; it is located near *CTDSP1* major TSS and not part of the loop that delineates *NRAMP1* locus (Figure S17). CTCF binding is not ubiquitous (Figure 1) but this site represents a DHS found in non-hematopoietic cells as well (Figures S15 and S16). It is flanked on both sides by histone marks of transcriptional activity (K9ac, K4me3) though decorations are reduced in ESC. Data suggest CTCF_C might regulate *CTDSP1* expression in the hematopoietic lineage and ENCODE ChIP-seq results show it may bind numerous TFs as well (Figure S9).

Site D is located just downstream of *CTDSP1* and strongly associated with CTCF (Figure 1). It forms a strong, ubiquitous DHS (Figures S15 and S16) lightly decorated on both sides by histone marks that indicate little transcriptional activation and argue against a role in the regulation of *NRAMP1*. However, since a large collection of TFs was associated with this site (Figure S9), and because it may be part of downstream loops (Figures S1B and S17) it is possible that CTCF_D contributes to regulate downstream genes, including *VIL1* and possibly *USP37*, *RQCD1*, *PLCD4*.

Weak constitutive STAT1 ChIP-seq signal was detected at CTCF sites C and D in human MDMs (using M-CSF), apparently unchanged by IFN-g priming and/or LPS activation, as well as weak IRF1 binding in response to IFN-g [99] (Figure S10). Data thus suggest little if any contribution of CTCF sites C and D to *NRAMP1* expression.

CTCF_E is the most upstream site of *NRAMP1* locus and it is predicted to form an upstream loop insulating *PNKD* gene (Figure S17). This site binds CTCF strongly and ubiquitously (Figure 1), yet it corresponds to a weak DHS, with minimal histone decorations (Figures S15 and S16) that suggest no role in *NRAMP1* regulation. In fact, CTCF_E bound few TFs (Figure S9).

CTCF_F is located nearby and downstream of site B (F12/5′ RH); it corresponds to a weak DHS, F13, found in myelo-monocytic cells (Figures S15 and S16). CTCF_F has a degenerate sequence motif, representing perhaps functional divergence within a functional TAD [55], and it binds CTCF weakly (NB4 APL and ESC; Figure 1). CTCF_F lies amid histone marks found in *NRAMP1* expressing cells; it was associated with few nuclear factors among which members of the cohesin complex, i.e., SMC3 and RAD21, implying a possible regulatory and/or topological role (Figure S9).

CTCF sites around *NRAMP1* locus may thus exert different roles. CTCF_A, which seems critical to insulate *NRAMP1*, and CTCF_B and CTCF_F that are part of the predicted insulated loop (Figure S17), embedded in *NRAMP1* candidate S-E (Figure S11), together appear most likely to exert regulatory roles in myelo-monocytic cells. CTCF_C may also control local transcription in hematopoietic cells.

#### 2.3.4. Area Downstream of *NRAMP1* 3′ CTCF

CTCF looping pattern and distribution of regulatory elements F1-F14 imply the ~40 kb interval delimited by CTCF sites A and E may delineate *NRAMP1* locus. Yet transcription data suggest a possible regulatory role for the adjacent stretch that separates CTCF_A and CTCF_C, at least in blood cells. Could this 3′ CTCF stretch form a *cis* determinant whose activity influences expression of both *NRAMP1* and *CTDSP1*?

Activation of this 3′ CTCF stretch is not necessary for *CTDSP1* expression (shown by RNA-seq and CAGE data from ESC, HUVEC and K562; Figure S1B); yet bi-directional transcription of this area coincides with transcriptional activation of F12/5′ RH (CTCF_B) and upregulation of *CTDSP1* expression (CD34^+^ HSPCs, CD14^+^ MNs and PBMCs; Figure S1B). So, hematopoiesis may activate *NRAMP1* 3′ CTCF stretch and stimulate *CTDSP1* transcription.

NIH Roadmap data in primary blood cells shows three profiles of DHS and histone marks at *NRAMP1*: non-expressing cells, e.g., T cell populations and K562 cells; expressing cells, e.g., CD14^+^ MNs, CD15^+^ PMNs; and populations with intermediate status (reflecting either moderate expression by a small fraction of cells or generally low level expression), including CD34^+^ HSPCs, PBMCs, splenocytes as well as CD56^+^ NK and CD19^+^ LBs (Figure S15).

A consistent picture among blood cells shows substantial transcriptional activity of *NRAMP1* 3′ CTCF stretch: it is accessible to DNAse and displays strong histone marks such as K4me1, K27ac, K9ac and K4me3 (Figure S15A,B). Patterns of K36me3 decoration and RNA accumulation indicate substantial expression of *CTDSP1*, independent of *NRAMP1* status (Figure S15C). In contrast, non-hematopoietic cell types show prominent accumulation of negative histone marks along *NRAMP1* 3′ CTCF stretch and reduced expression of *CTDSP1* (Figure S16C).

Activation of *NRAMP1* 3′ CTCF stretch in hematopoietic cells is accompanied by low level transcriptional activity of upstream CTCF_B (F12/5′ RH; Figure S15C and S16C). Those CTCF determinants however are insufficient to support *NRAMP1* gene expression, which requires myelo-monocytic TFs. Blood cell data thus suggest activation of *NRAMP1* 3′ CTCF stretch stimulates *CTDSP1* expression only.

Yet may this 3′ CTCF stretch influence *NRAMP1* expression? Data from AMLs and CD34^+^ cells show that CTCF_B (F12/5′ RH) and *NRAMP1* 3′ CTCF stretch exhibit similar profiles of TF binding (cf. Section 2.4.4 and Figure S9). Also, RNA data suggest these determinants become activated at the same time (Figure S1B). One possible interpretation might be they interact functionally in cells poised to express *NRAMP1*.

Though CTCF_A (3′ UTR) and CTCF_B (F12/5′ RH) exhibit convergent orientations, respectively “R” and “F”, they may not interact directly as CTCF_A may rather be mobilized in upstream loops (Figure S1B and S17) while CTCF_B showed no association with cohesin complex components (Figure S9). Alternatively, CTCF_F and CTCF_C might interact directly through cohesin-mediated looping (‘handcuff model’) [108]. It may be hypothesized that such a loop, nested in *NRAMP1* TAD (CTCF_157 and CTCF_A; Figure S17), could restrict gene expression in early stages of myelopoiesis, while myelo-monocytic differentiation would provide TFs able to activate *NRAMP1* candidate S-E (e.g., F13 and F5 sites) and in turn disrupt interaction of CTCF_F and CTCF_C.

Interestingly, one SNP part of the ~2 kb fragment spanning CTCF site A (cf. Section 2.3.3), and which is 3′ adjacent to element F3, was identified as genome-wide significant *cis* reQTL in response to *Salmonella* infection [54]. Another cis reQTL involved in MF response to infection with *Listeria* is situated within element F13 [54]. (Epi)genetic data therefore suggest potential interaction between F12/5′ RH-F13 area and *NRAMP1* 3′ CTCF stretch, warranting further investigation of their possible regulatory role.

Myelo-monocytic events triggering reorganization of *NRAMP1* locus would thus include (Figures S15 and S16): (i) increased mobilization of F12/5′ RH, indicated by stronger DHS, (ii) specific activation of F9/3′ RH, resulting in DHS distinct from ESC, together with (iii) recruitment of (pioneer) TFs at sites such as F6; F13, F5 and F7, corresponding to novel DHS, and (iv) extensive K4me1 deposition, spreading bi-directionally from F12/5′ RH and F9/3′ RH sites to cover most of *NRAMP*1 locus, as well as (v) decoration of TF binding areas with histone 3 acetylation marks K27/9ac. Transition from non-expressing cells (e.g., ESC) to cells expressing or prone to express *NRAMP1*, is also indicated by a shift of K4me3 mark, from F9/3′ RH site toward F1/TSS and F12/5′ RH and downstream area, as well as by histone marks of ORF transcription (e.g., K36me3) decorating *NRAMP1*.

In sum, initiating hematopoiesis would erase histone 3 inhibitory marks (K9me3 and K27me3) from *NRAMP1* 3′ CTCF stretch, elevating *CTDSP1* transcription level but favoring some putative interaction between *NRAMP1* CTCF sites F (DHS F13) and C (3′ CTCF stretch) until myelo-monopoiesis is engaged. According to this hypothesis, activation of *NRAMP1* 3′ CTCF stretch would prevent expression of *NRAMP1* until specific myelo-monocytic TFs become available. Transcription of *NRAMP1* 3′ CTCF stretch thus suggests priming of *NRAMP1* locus in mobilized CD34^+^ cells (CMP stage).

#### 2.3.5. Autonomous Regulation of *NRAMP1* Locus

CTCF predicted looping and gene expression data both suggested regulation of *NRAMP1* by elements intrinsic to the locus delineated by CTCF_A and CTCF_E, while CTCF_C downstream of CTCF_A might exert an additional role through reversible interaction with CTCF_F (cf. Section 2.3.4).

Broader examination of chr2 extended domain that carries *NRAMP1* locus (cf. Section 2.1; 205 kb: 219,075,000-219,280,000) provides additional arguments supporting autonomous regulation of gene expression: the CTCF sites that segment this extended domain map at gene boundaries, suggesting they segregate distinct regulatory units (Figure S1A); K27ac marks generally correlate with CTCF sites and gene TSSs, implying each ORF may be regulated independently; *NRAMP1* locus is the sole wherein strong C/EBPb interactions were found in both MNs and MDMs [13]; other ChIP-seq studies showed binding of HIF factors (HIF1a and HIF2a) only at *NRAMP1* locus [103], wherein STAT1 DNA association was enriched compared to neighbor genes (four strong signals vs. two, *TMBIM1*; one, *AAM*; one, *ARPC2*); in addition, only *NRAMP1* locus exhibits myelo-monocytic specific DHS co-localizing with K27ac/K4me1 marks, and it displays a unique balance of histone marks of transcriptional activation (K9ac, K4me2 and K4me3) that distinguishes MN chromatin from those of K562 cells, HUVEC and ESC; lastly, it is the sole locus showing strictly myelo-monocytic specific transcription (Figure S1B).

*NRAMP1* epigenetic pattern differs from both neighbor genes, including those expressed in the myelo-monocytic lineage (e.g., *ARPC2*, *GPBAR1*, *AAMP*, *TMBIM1*; and *PNKD* that appeared insulated from surrounding genes; Figure S17), as well as more distant genes located upstream of chr2 205 kb extended domain which are also expressed in the myelo-monocytic lineage (*RUFY4*, *CXCR1* and *CXCR2*).

Altogether data suggest that, within this chr2 205 kb interval, CTCF boundaries delineate functional gene units, supporting that *NRAMP1* 40 kb locus comprises the regulatory determinants controlling myelo-monocytic expression. Prior genetic analyses indicated that *NRAMP1* locus is divided in 5′ and 3′ haplotypes [109,110,111] (Figure 5B). Most of TF interactions revealed so far take place within *NRAMP1* 5′ haplotype (e.g., PU.1, C/EBPb, HIFs, IRF1) while several negative interactions involved determinants from the 3′ haplotype (e.g., F10, F3 and the 3′ CTCF stretch). *NRAMP1* 5′ and 3′ haplotypes may therefore reflect distinct regulatory roles to control myelo-monocytic determinants within *NRAMP1* TAD.

### 2.4. Recapitulating the Process of NRAMP1 Gene Activation during Development

#### 2.4.1. Reappraisal of *NRAMP1* Tissue-Specificity Using Large Scale Transcriptomic Datasets

Multi-organ RNA-seq analyses [50] demonstrated *NRAMP1* gene expression is tissue-specific (principally found in blood, spleen and lung, ranked by decreasing transcript abundance). These data confirmed prior studies which showed high level expression in professional phagocytes [74,112], and revealed some molecular mechanisms controlling *NRAMP1* transcription specificity, using model cell lines for myelo-monocytic differentiation, such as granulocyte-MF progenitors (HL-60), [12,76,113,114,115] and monocyte-like cells (U-937 and THP1) [104,105,116,117].

Examining *NRAMP1* locus decoration with K36me3 in a variety of tissues (121 samples, NIH Roadmap) [14] confirms that ORF transcription is restricted to tissues highly enriched in MFs, such as spleen, or in MNs (e.g., PBMC), and explanted phagocytes such as CD14^+^ MNs and CD15^+^ PMNs. Collectively, these data indicate *NRAMP1* expression is strictly controlled during hematopoiesis, and specifically induced along the myelo-monocytic pathway. Integrating chromatin properties at *NRAMP1* locus in cellular proxies of discrete stages of this developmental pathway should thus suggest a plausible sequence of events controlling *NRAMP1* expression.

#### 2.4.2. Segmenting *NRAMP1* Locus in Five Regulatory Regions

Areas F1-F14 defined on the basis of DHS and CAGE data represent key candidate regulatory elements controlling *NRAMP1* gene expression during myelo-monocytic development; also, some of these and/or additional elements may modulate gene expression in response to environmental stimuli which include tissue context, i.e., development stage, location and physiological conditions such as homeostasis or inflammatory and/or infectious states [118,119,120].

Hence *NRAMP1* expression in MFs may be modulated to match metabolic (re)programming during phenotypic adaptation to tissular environment and in response to inflammatory context [121,122]. *NRAMP1* expression also has to be tightly controlled because the protein imports into the cytoplasm metal elements such as iron, manganese and cobalt, which are potentially noxious since they may perturb evolution of oxygen radicals either massively produced as antimicrobial defense or derived from respiratory activity and signaling pathways [123].

Several studies documented the regulation of mononuclear phagocyte genes by the microenvironment. For instance, MF-specific enhancers may be established in precursors, being occupied first by primary factors that poise chromatin locally and bookmark it for subsequent expression at a later stage of differentiation [72]. Also, areas controlling basal gene expression may become remodelled in response to inflammatory signals by transcription factors such as NF-KB [33,124].

The functional importance of inflammatory remodeling of chromatin was highlighted in studies showing that pharmacological inhibition of gene activity triggered by environmental transitions allows better control of various pathological conditions (e.g., arthritis and cancer) [125,126]. Likewise, genome regions targeted during fast transitions and/or pathological conditions frequently constitute S-E domains which are enriched for TFBSs not only within, but also outside of known DHS; and disease- or QTL-related SNPs also map frequently outside established DHSs within S-E domains [127].

Accordingly, regulatory determinants of *NRAMP1* expression are probably not limited to DHSs detected in CD14^+^ MNs and CD15^+^ PMNs, which likely control basal activity levels; rather, they may include additional elements part of the segments spanning them, which are decorated by K4me1 and K27ac and may carry binding sites for other TFs required to regulate expression in response to environmental variations (e.g., NF-KB and SMAD3).

**Figure 7:**
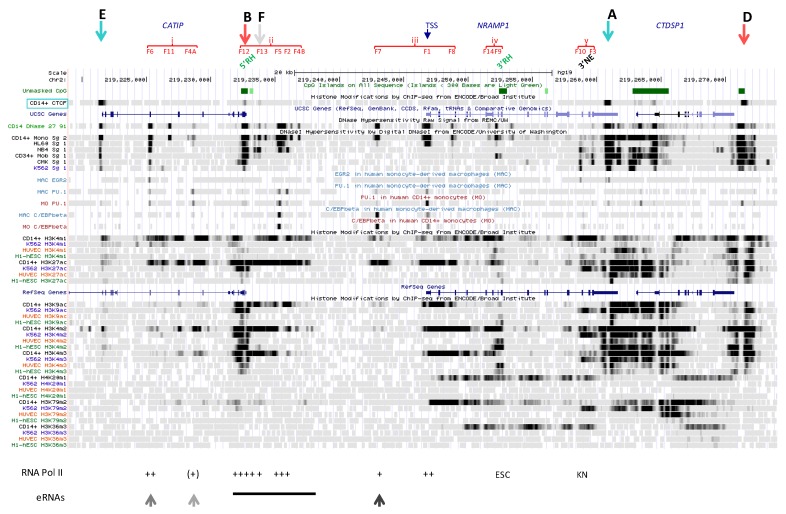
Epigenetic modifications and myeloid transcriptional regulatory elements at *NRAMP1* locus (data from ENCODE consortium [9,10,78] or stated otherwise). From top to bottom: Gene names, location of CTCF sites delineating *NRAMP1* locus (light blue arrows, A and E) and *CTDSP1* gene (red arrow, D) and position of *NRAMP1* TSS (blue arrow); chromosome segments comprising transcriptionally active regions; position of DHSs (F1-F14), RHs and negative element (NE); chromosome 2 scale; CpG islands; CTCF sites in CD14^+^ MNs; UCSC gene descriptions; DNAse1 footprints in CD14^+^ MNs, HL-60, NB4, mCD34, CMK and K562 cells; EGR2, PU.1 and C/EBPb ChIP-seq data from MO and MAC [13]; H3K4me1 and H3K27ac ChIP-seq data from CD14^+^ MNs, K562 cells, HUVEC and ESC; RefSeq genes; H3K9ac, H3K4me2, H3K4me3, H4K20me1, H3K79me2 and H3K36me3 ChIP-seq data from CD14^+^ MNs, K562 cells, HUVEC and ESC; areas bound by RNA Pol II in HL-60 cells, except ESC (Embryonic stem cells) and KN (K562 and NB4 cells); eRNAs: major CAGE signals in CD14^+^ MNs [68]. Details on the transcriptionally active regions i–v are presented in Figure 2, Figure 3, Figure 4, Figure 5 and Figure 6 and Figures S3–S7 and S14.

*NRAMP1* locus can then be subdivided in five regions (Figure 7) which may operate independently or in complementary ways to control gene expression as hematopoiesis progresses on the one hand, and to mediate environmental adaptation on the other hand:

-region (i) a set of three upstream elements (F6, F11, F4A, ~5 kb) embedded in *CATIP* gene and potentially extending the S-E domain in 5′ (region ii) to mediate signal-dependent regulation in phenotypically diverse phagocytes;

-region (ii) a large cluster of upstream regulatory determinants (F12, F13, F5, F2 and F4B, ~6.5 kb) likely behaving as a super-enhancer (S-E) in mature phagocytes, which contains a candidate 5′ RH/CpG island (F12) that is already mobilized in non-hematopoietic cells;

-region (iii) a ~7 kb block centered on *NRAMP1* TSS encompassing the proximal promoter, a typical upstream enhancer and a downstream, intronic, potential regulatory element (F1, F7, F8, respectively);

-region (iv) an intronic, ~2 kb segment comprising two elements (F14, F9), including another candidate 3′ RH/CpG island mobilized in non-expressing cells as well, but differentially activated in professional phagocytes, and

-region (v) an area at the 3′ end of the gene that contains negative elements showing specific activation in myeloid cells that do not express *NRAMP1* at high level (F10, F3).

Investigating epigenetic mechanisms of innate memory suggests distinct functional roles for regions i and ii of *NRAMP1* locus. Hence, MN stimulation for 24 h with fungal beta-glucan (BG) mobilizes these two regions, as well as region iii, suggesting upregulation of *NRAMP1* expression (Figure S18). Notably, five days after BG treatment *NRAMP1* locus appears as active as in freshly explanted MNs, showing very similar histone modification profiles for regions ii and iii, plus low level activation of region i (sites revealed by Tn5 transposase accessibility (ATAC-seq): F6 (and F4A), within K27ac marked area), and very low level activation of regions iv and v (increased transposase accessibility; Figure S18).

In contrast, 24 h treatment of MNs with bacterial LPS also increases *NRAMP1* locus accessibility, but compared to BG, it stimulates more regions ii and iii and less region i; then, five days post-treatment, both ATAC-seq and histone modification ChIP-seq data demonstrate gene activity level inferior to fresh MNs (Figure S18). Lastly, MN incubation in RPMI alone for 24 h reduces significantly *NRAMP1* activation, while culture for five more days restores moderate gene activity that is intermediate between freshly explanted MNs and LPS treated MNs (Figure S18).

These data appear consistent with BG inducing trained innate immunity [15] because *NRAMP1* activity level induced by 24 h BG treatment was maintained for five subsequent days in absence of stimulus. In addition, data suggest that balanced activation of *NRAMP1* regions i and ii may mediate BG-induced innate epigenetic memory by preserving long term gene expression level. Along similar lines, strong and preferential mobilization of region ii may mediate LPS tolerance, because *NRAMP1* appeared turned off five days after stimulation [128].

In sum, *NRAMP1* response appears to recapitulate both MN refractory functional state (immunotolerance), characterized by incapacity to produce proinflammatory mediators after the initial stimulation phase triggered by LPS, and BG-induced trained immunity, typified by an exclusive epigenetic signature that affects both the promoter and distal regulatory elements [15,128]. Physical delimitation of *NRAMP1* regulatory regions i and ii has thus functional significance.

#### 2.4.3. Regional Predictions of Potential TFBSs

Predicting TFBS in regions i–v using Mapper2 [129] indicates they are not restricted to DHS areas but distributed in between as well. Putative TFBS were examined for (i) homologs of mouse TFs shown to be activated differently either in vivo between large peritoneal MFs and microglia [119] or in vitro between classically vs. alternatively activated MFs [130]; (ii) human TFs regulated during either PMA-induced growth arrest of THP1 monocytic cells [131] or polarized activation of primary MFs [132]; and (iii) candidate pioneer TFs possibly involved in hematopoiesis [133,134,135,136].

Overall, the five regions exhibit similar frequency of putative regulatory elements (2.15 ± 0.2 per 100 nt) with slight enrichment or depletion in regions i and ii, respectively. Some regions display candidate TFBS evenly distributed among DHSs and intervals between them: region ii, and region iii (except areas around F7 and downstream of F8, relatively depleted) while regions i, iv and v show enrichment around specific DHSs (respectively, F6 to F11 and downstream area, F9 and downstream area, and F3 and downstream area). Conservation of predicted TFBS among primates or simians, as suggested by UCSC browser [48], displays inter-region variations as well, with peak levels (>85% TFBSs, region i, DHS F6; region ii, F12 and surrounding areas; region iii: F7, F8 and surrounding areas; region iv: F9 and surrounding areas; region v, F10-F3 stretch), and bottom levels (<45% TFBSs, region i upstream of F4A; region ii, F2-F4B linker; region iii, areas surrounding F7).

Regarding potential pioneer TFBS, enrichment (~25% of all predicted TFBS) was noted in some areas (region i: F11 and F4A; region ii: F5/F2 and upstream segment; region iii: F1 and downstream area, area downstream of F8; region iv: areas surrounding F14, F9; region v: F3 and downstream area). Conservation among primates or simians was high (>85%) in region i, except F4A; in region ii, except F5/F2 area and segments linking F12 and F13 as well as F2 and F4B; in region iv, except F14 and downstream of F9; in region v, except areas upstream of F10 and downstream of F3; in region iii, only F1 and downstream area and F8 downstream area showed high proportion of conserved sites.

Predicted BSs were quite abundant for some TFs (C/EBPs, NF-KB, SP1/3, IRFs, SOXs, NKXs, MZFs, ETS, HLHs, FOX). NF-KB sites were generally predicted in intervals separating DHSs as well as NKX sites (abundant in region i), while C/EBP, FOX, ETS, MZF, IRF and SP sites were found both within and between DHSs as well as SOX sites (underrepresented in region i), and HLH sites mostly predicted within DHSs.

Other BSs were scarcely found, such as AP-1 (both in DHSs and intervals, region i, iv and v), CTCF (DHSs in region iii), PU.1 (within DHSs, regions ii and iii, between DHSs, regions iii and iv), MAFG (DHS in region iii), EGR (DHS, regions iii and v), NRF2 (DHSs in regions i and iii), NF-AT (DHSs in region ii and iii, between DHSs, regions iii) and GKLF (both in DHSs, regions i, iii and v, and between DHSs, regions i and iii), whereas few TFBSs were predicted more frequently, such as CREB (DHSs only, not in region iv), USF, SMAD and STAT (predominantly between DHSs), RUNX and NFY (both absent from region ii).

Thus both known DHSs and contiguous areas decorated only with K4me1/K27ac may carry TFBSs that potentially modulate *NRAMP1* expression in response to environmental cues.

#### 2.4.4. *NRAMP1* Locus Activity in Early Developmental Stages of Hematopoiesis

ESC, multi-potent cells capable of self-renewal and multi-lineage differentiation, and mesodermal HUVEC provide outliers to the definitive hematopoietic lineage. However, given emergence of erythro-myeloid precursors (EMPs) during embryogenesis and before the production of HSC [137,138] it may not be surprising that *NRAMP1* locus shows opened chromatin in ESC. Nevertheless, *NRAMP1* remains silent in ESC and HUVEC whereas chromatin reorganization as hematopoiesis progresses leads to gene expression.

HUVEC and ESC both share *NRAMP1* locus CTCF boundaries (Figure 1 and Figure 7), activation marks such as DHS and decorations with modified histones that are restricted to CTCF_A-D and the candidate RHs in regions ii and iv (F12/5′ RH and F9/3′ RH; Figure S16A,B), which both bind RNA Pol II (Figures S3 and S6). Yet, minimal CAGE and RNA-seq signals (Figure S14, regions ii and iv) plus inhibitory marks such as K9me3 and K27me3 (Figures S13 and S16C) indicate limited activation, insufficient to trigger *NRAMP1* expression. CAGE suggests similar status for *NRAMP1* locus in CD133^+^ HSCs [68].

CD34^+^ HSPC is the next developmental step to consider because HSPCs give rise to all blood cell types. Their distinct DHS profile includes intense peaks at F12/5′ RH (CTCF_B), 3′ CTCF stretch (spanning sites A to C) and CTCF_D, as well as modest peaks at F9/3′ RH and the 5′ CTCF_E. Trace DHS at F6, F13, F5, F10-F3 and F7, F1 (Figures S10, S15A and S19) [139] indicate elements that become activated later, during myelo-monopoiesis. RNA-seq and K36me3 data (Figure S15B,C) plus histone 3 acetylation and K4me3 marks decorating regions ii and iii (Figure S15A,B) indicate marginal gene expression (Table 1). Activation of F12/5′ RH and 3′ CTCF stretch thus render CD34^+^ HSPCs more prone to express *NRAMP1* than CD133^+^ HSC.

AMLs arise from (epi)genetic abnormalities in HSC and evolve into aggressive blood cancers caused by the proliferation of immature myeloid cells [140]. *NRAMP1* epigenetic profile in different AML models [139,141,142,143,144] may therefore inform on TF spatiotemporal contribution to regulate *NRAMP1* expression.

Constitutive activity of the tyrosine kinase receptor FLT3 is due to secondary mutations (such as internal tandem duplications, ITD) frequently found in AMLs [139]. DHS profiles of FLT3-ITD AMLs are similar to CD34^+^ HSPCs with few differences: reduced footprints at F12/5′ RH and F9/3′ RH but increased signal at F6 (Table 1).

Similar to FLT3-ITD cells, two AMLs display stereotypical patterns of nuclear factor association with *NRAMP1* locus (Figures S19, S21 and S22): Kasumi (a model for t(8;21) producing AML1-ETO fusion protein; Figure S21) [143] and ME-1 cells (with inversion of chromosome 16, inv(16)(p13q22) yielding the chimeric protein CBFb–MYH11) [144]. In Kasumi cells both HAT (p300) and co-repressor activities (NCoR,[143]; HDAC1 and 2, Figure S21) were associated with F12/5′ RH and *NRAMP1* 3′ CTCF stretch, implying dynamic regulation that is corroborated in ME-1 cells, wherein RNA Pol II, TBP, p300 and HDAC also bind these elements. In addition, both F6 and F10-F3 bound p300 and HDAC in ME-1 and Kasumi cells, implying early mobilization of these elements downstream of CD34^+^ HSPC.

The association pattern of TF RUNX1/AML1 with *NRAMP1* in AMLs provides further information. RUNX1/AML1 is required for emergence of both EMPs and HSCs [145]; it associates with F12/5′ RH, F10-F3 and *NRAMP1* 3′ CTCF stretch in both CD34^+^ cells and FLT3-ITD AML but binds F6 in the latter only [139] (Figure S19). RUNX1 binding at loci encoding phagocyte effector functions (e.g., *CD14*, *CD15*, *S100A8/9*, *IL-12b*, *NF-KB*) also shows additional FLT3-ITD-specific peaks [139], supporting that FLT3-ITD AML are locked in an hematopoietic stage downstream of CD34^+^ HSPCs that induces mobilization of *NRAMP1* DHS F6.

RUNX1 binding profile in FLT3-ITD AML overlaps those reported in AML blasts and Kasumi cells, which show prominent binding to F12/5′ RH and 3′ CTCF stretch. Kasumi RUNX1(AML1)-ETO fusion can act as a dominant regulator by recruiting HDACs and/or NCoR or p300 HAT, and it is less specific than RUNX1 vis-a-vis its recognition site [142]; prominent association of AML1-ETO with *NRAMP1* F10 element in Kasumi may thus result from relaxed DNA binding specificity.

RUNX1 binding pattern further extends in TSU-1621-MT AML (expressing the FUS–ERG fusion protein due to translocation t(16;21)(p11;q22)) [141]. In these cells, RUNX1 also binds DHS F5, in addition to F6, F10-F3, F12/5′ RH and *NRAMP1* 3′ CTCF stretch (Figure S20), suggesting that mobilization of F5 elements follows that of F10-F3 and F6.

TSU-1621-MT cells also show binding of PU.1 (SPI1) to F5-F2 determinants in addition to other elements (F6, F10-F3, F12/5′ RH, 3′ CTCF stretch and 5′ CTCF site (E); Figure S20A). Mobilization of DHS F5 by both RUNX1 and PU.1 may thus imply a more advanced stage of differentiation than those of Kasumi and ME-1 cells. Accordingly, genes activated during neutrophil development (*MPO*, *LTF*, *LCN2*, *CD11b*, *LYZ*) appeared more prone to expression in TSU-1621-MT cells than in Kasumi and ME-1 cells [141,142,143,144]. Together, data suggest increased activation of *NRAMP1* locus in TSU-1621-MT cells due to mobilization of F5 element.

RUNX1 pattern of chromatin association in NB4 APL [146] further highlights the mobilization of F5 element during myelo-monopoiesis, in the transition toward GMP. In NB4, RUNX1 binds F13 and F5-F2 DHSs, in addition to F6 and F10, F12 and 3′ CTCF stretch (Figure S21). These sites also bind RNA Pol II and the HAT p300 (Figure S20B), indicating increased activation of *NRAMP1* in these cells. On the other hand, F6, F5 and F7 areas are resistant to the restriction enzyme *Hpa* II which is blocked by CpG methylation (Figure S20B). In contrast, blood cells demonstrate hypo-methylation of these areas (CD34^+^ HSPCs, PMNs and MFs; Figure 2B, Figure 3A,B, Figure 4A,B, respectively). Local demethylation thus contributes to activate *NRAMP1* expression yet this process is impaired in NB4 cells, which also retain K9me3 mark upstream of F12/5′ RH (Figure S20B).

RUNX1 binding data in TSU-1621-MT and NB4 AMLs point mobilization of F13 and F5-F2 elements as the next step to activate *NRAMP1* expression, which apparently depends on CpG de-methylation. Besides, treatment of both cell lines using *all-trans* retinoic acid (ATRA) does not stimulate *NRAMP1* expression, as previously reported for HL-60 promyelocytic AML [74]. Epigenetic data collected from various AMLs thus confirm the interaction of RUNX1/AML1 with *NRAMP1* locus observed in normal CD34^+^ cells.

AML data together indicate that *NRAMP1* F12/5′ RH and 3′ CTCF stretch are co-activated early in the hematopoietic lineage and subjected to dynamic regulation (indicated by bound p300/HDAC). These determinants may constitute major regulatory elements because they recruit several TFs, including PU.1, ERG, FLi-1 and RUNX1, as well as RNA Pol II and TBP in various AMLs (Figures S19–S22). These data strengthen the suggestion that F12/5′ RH may interact with *NRAMP1* 3′ CTCF stretch, through cohesin-mediated contact between CTCF sites C and F (cf. Section 2.3.3. and Section 2.3.4.; Figure S17). Alternatively, the observed stereotypical patterns of TF binding might indicate non-specific “phantom peaks” due to high level transcription of the corresponding regulatory elements [68,147]. Site-directed mutagenesis studies will be necessary to demonstrate the functional role of these elements.

Lastly, AML data suggest subsequent mobilization of F10-F3 and F6 determinants, subjected to dynamic regulation in the first steps of hematopoiesis (e.g., Kasumi and ME-1 cells), and preceding activation of elements F5 and F13 at the onset of myelopoiesis (e.g., TSU-1621-MT). Since PU.1 acts early in blood development, possibly as a pTF [148], CD34^+^ and AMLs thus provide early snapshots of the developmental program that activates *NRAMP1*. De-activation of DHS F10 element (cf. Section 2.2.2.1 and Section 2.2.2.2) may represent a further step as myelo-monocytic differentiation progresses.

#### 2.4.5. Activation of *NRAMP1* Regulatory Regions i–v during Myelo-Monopoiesis

Upon mobilization with G-CSF CD34^+^ HSPCs progress toward the stage of GMP while bifurcating away from MEPs. Such mCD34 cells, which represent CMPs, may thus be considered as the first stage of the myelo-monocytic lineage, while immortalized cell lines CMK, K562 and HL-60, NB4 represent proxies to downstream progenitors committed toward either megakaryocytic, erythrocytic or myelo-monocytic lineages, respectively, and CD15^+^ PMNs and CD14^+^ MNs constitute end points of the granulocytic and monocytic pathways. Comparing the transcriptomic and epigenomic status of *NRAMP1* regulatory regions i-v among these cell types should inform on the mechanisms of gene activation during myeloid development (Table 1).

##### 2.4.5.1. Region ii: 5′ Regulatory Hub and Candidate Super-Enhancer

mCD34 progenitors demonstrate moderate transcriptional activity of element F12/5′ RH (Figure S14). DNAse footprints map essentially to F12 and F5 (Figure 7 and Figure S15A) together with K4me1 and K9/27ac decorations while K4me3 marks F12/5′ RH element only (Figure S15A,B). Region ii activation in CMP thus appears restricted to F12/RH and F5 elements (Table 1).

Regarding MEPs, erythrocytic K562 cells showed minute amounts of RNA-seq tags around F12/5′ RH in the absence of CAGE clusters (Figure S14). DNAse1 accessibility is confined to this element while a larger area was delimited in megakaryocytic CMK cells (Figure 7). ChIP-seq assays in K562 revealed local decoration of F12/5′ RH with K9ac and K4me2/3 as well as K27ac, and limited marking with K4me1 and K79me2 (Figure 7 and Figure S15). These data show little residual activity of F12/5′ RH only as erythro-megakaryocytic differentiation progresses (Table 1).

In contrast, maturation toward GMP (HL-60 and NB4) extends DNAse footprints downstream of F12/5′ RH, including F13, F5 and F2 elements (Figure 7). Binding of RNA Pol II at F13 (NB4) and F5 (HL-60) also increases the range of interactions compared to K562 cells (Figure S3). Arguably, myelo-monocytic differentiation thus allows the recruitment of novel TFs and associated factors (e.g., DNA and histone demethylases), which stimulate transcription of region ii 3′ elements (Table 1). Such polarity among enhancer elements seems consistent with the directionality commonly observed for activating signals [18].

Lastly, in CD14^+^ MNs and CD15^+^ PMNs, massive transcription from F12/5′ RH to F2 elements indicates global activation of region ii (Figures S14 and S15). Strong DNAse1 footprints highlight F12/5′ RH and F5 sites, which form together with F13, F2 and F4B a large cluster of active elements (~5 kb wide; Figure 7 and Figure S15). Histone marks of enhancer priming (K4me1 and K27ac) cover the area, spanning ~7 kb, and those indicating transcription (K9ac, K4me2/3) decorate a shorter interval. Both sites F12 and F5 are depleted of nucleosomes, and F5 binds TFs key for myelo-monocytic fate, i.e., PU.1, C/EBPb, STAT1 and IRF1 (Figure 7, Figure S10) [13,25,99].

*NRAMP1* region ii extending from F12/RH to F4B constitutes a strong candidate CD14^+^ MN S-E (Figure S11) because maximal activation of this 5 kb region correlates with maximal expression of *NRAMP1* (Table 1, Figure 7), and depends on myelo-monocytic specific TFs. Region ii is also strongly and selectively activated as MNs respond to short term stimulation with LPS, a condition which results in posterior immunotolerance (cf. Section 2.4.2) [15,128].

##### 2.4.5.2. Region i: Most Upstream Elements

CMPs display modest CAGE cluster (Figure S14) and faint DNAse footprint at site F6, which is weakly decorated with K4me1 and traces of K27ac (Figure S15A), implying little mobilization at this stage (Table 1).

Progression toward MEP pathway reduces F6 activation (Table 1), as K562 cells display weak DHS and minimal decoration with K4me1 only (Figure 7, Figure S15A), in absence of eRNA (Figure S14). CMK cells also show moderate accessibility at F6 suggesting limited mobilization of region i in both erythrocytic and megakaryocytic lineages.

Around GMP stage on the other hand, F6 appears more accessible in both HL-60 and NB4 cells (Figure 7). HL-60 demonstrates significant binding of RNA Pol II (Figure S5), also observed at F4A, which suggests moderate activation (Table 1). Region i may thus be mobilized concomitantly to activation of region ii.

In MNs and PMNs, elements F6 and to lesser extent F4A, both show transcriptional activity (Figures S4 and S14). DNAse footprints identify another site in-between, F11, (Figure 7, Figure S15). DHSs show decreasing intensity from F6 to F4A, yet in MNs and MDMs F4A binds PU.1 more strongly than F6. Histone 3 decorations show relatively uniform priming (K4me1 and K27ac), and less intense indications of transcriptional activity, incrementing towards F4A (predominance of K4me2 over K4me3, and K9ac traces; Figures S10 and S15A,B), implying limited activation (Table 1).

Region i thus displays also asymmetric activation in mature phagocytes. This most upstream region may require stimuli-dependent TFs to reach full-activation and in turn, extend in 5′ the S-E domain delineated by region ii [20,119]. Indeed, region i is activated in MNs exposed to microbial stimuli and displays specific epigenetic memory in response to BG (Figure S18). Differences between MDMs and PMNs in CpG methylation at F11 and F4A sites (Figure 4B) suggest such stimuli might have cell-type specific effects.

A conserved haplotype spanning F6 and F4A sites comprises nine SNPs representing QTLs that explain an amount of the variance observed in mRNA levels (eQTLs). Within this short interval (chr2:219,225,957-230,213 bp) five SNPs, distributed in blocks of three and two polymorphisms separated by a ~1 kb segment, were associated with identical phenotypes in independent analyses: homozygous SNPs correlated with strong down-regulation of *NRAMP1* expression in both blood cells (GTEx post-mortem analysis of gene expression in tissues) [50] and M2-type MFs, independent of infection (i.e., in basal conditions and post-infection with either gram positive or gram negative bacteria) [54]. For each of these SNPs, heterozygosity yielded expression levels similar to WT in both types of analyses.

Genome-wide genetic analyses thus support the notion that region i is mobilized in certain environmental conditions, such as those prevailing in the general circulation in vivo or after MF polarization in vitro. Moreover, balanced activation of regions i and ii during MN response to BG seemed to mediate trained innate immunity (cf. Section 2.4.2) [15,128].

##### 2.4.5.3. Region iii: Around *NRAMP1* TSS

CMPs exhibit weak DNAse accessibility of the elements in this area (proximal enhancer F7, TSS (F1) and intronic site F8; Figure 7 and Figure S15A), a small CAGE cluster at *NRAMP1* TSS (Figure S14), and very low level of mRNA (Figure S15C). Consistent with limited activation of region ii sites F12 and F5 (Figure S14), data imply very low level ORF transcription at this stage (Table 1).

Erythrocytic cells (K562) lack DNAse footprints and show neither activation of the TSS area nor decoration with modified histones (Figure 7, Figure S15). Thus ORF transcription is shut down through bifurcation toward MEP as residual activity of region ii F12/5′ RH does not suffice to express *NRAMP1* (Table 1).

Data from the GMP stage substantiate this point: HL-60 cells exhibit RNA Pol II binding at sites F7, F1 and F8 (Figure 3B and Figure 7) contrary to NB4 cells (Figure 3). Defective activation and persistent methylation of region ii (Figure 2 and Figure S20) may explain the difference in mobilization between regions ii and iii in NB4 (Table 1).

CD14^+^ MN CAGE signals match DNAse footprints at all sites in region iii (Figure S14) and correlate with maximal *NRAMP1* gene expression (Figure 1 and Figure S15). These sites appear similarly primed (K4me1 and K27ac decorations) but differ in binding of TFs PU.1, C/EBPb, STAT1 and HIFs [13,99,103] and extent of transcriptional activity (K4me2, K4me3 and K9ac; Figure 7, Figure S10 and S15).

Priming of region iii DHSs may be coordinated during myelo-monopoiesis while their activation could proceed independently (Table 1). Indeed, PU.1 signals may arise at promoters, poising chromatin for myelo-monocytic fate specification by maintaining it open for later binding by promoter-associated TFs such as C/EBPb, a key regulator of MN/Mac lineage [72], and STAT1. This scenario fits DHS F1. In contrast, intronic element F8 is weakly associated with STAT1 only, while full activation of F7 upstream enhancer (i.e., decoration with K4me2/3 and K9ac marks) may occur later, through binding of stimulus-dependent TFs such as HIFs, STAT1 or IRF1 (Figure S10) and in response to microbial compounds (Figure S18).

##### 2.4.5.4. Region iv: Intragenic 3′ Regulatory Hub

CMPs display CAGE signal in intron 6 that matches a dual DNAse footprint (DHS F9; Figure S14); decoration of the area with K4me1 and to lesser extent with K9ac, K4me3 and K27ac (Figure S15), suggests this site may be transcriptionally active and contribute to prime *NRAMP1* (Table 1).

Erythrocytic K562 cells show little signal, regarding RNA transcription, DNAse footprint or modified histone marks (Figure 7, Figures S14 and S15), therefore implying some correlation between inactivity of region iv and lack of *NRAMP1* expression (Table 1).

This point is substantiated by comparing data from promyelocytic AMLs either prone to express *NRAMP1* (HL-60) or not (NB4): NB4 chromatin displays neither DNAse footprint nor RNA Pol II binding whereas HL-60 cells exhibit weak signals at F9/3′ RH site (Figure 7 and Figure S6; Table 1).

In CD14^+^ MNs and CD15^+^ PMNs, transcriptional signals from region iv appear relatively weak compared to those of flanking exons (Figures S6 and S14). Yet presence of DHSs F14 in intron 5 and F9/3′ RH in intron 6, and decoration of the corresponding areas with histone marks both suggest activation of these determinants (Figure 7, Figures S14 and S15). Transcriptional activation of region iv may thus contribute to stimulate *NRAMP1* expression in myelo-monocytic cells (Table 1).

##### 2.4.5.5. Region v: *NRAMP1* 3′ Negative Elements

CMPs display small but significant CAGE signal that matches a weak DNAse footprint at site F10 in intron 12 (Figure S14); their chromatin also shows other footprints, in intron 13 (similar intensity) and at intron 14/exon XV junction (stronger, F3; Figure 7). Evidence of transcription and decoration with K4me1 and K27ac spanning F10-F3 sites (Figure S15) suggest region v may be active in mCD34 cells. Low level *NRAMP1* expression and modest activation of regions ii-iv may seem consistent with an inhibitory role of region v (Table 1; cf. Section 2.2.2.1).

K562 erythrocytic cells show strong CAGE signal (Figure S14) and DNAse footprint at F10 site (Figure 7) as well as abundant RNA Pol II binding (Figure S7). Marked DHSs are also detected at both F10 and F3 sites in CMK megakaryocytic cells (Figure 7). K562 cells also display marks of transcriptional activation (K9ac, K79me2, K4me2 and K4me3) at F10-F3 sites, consistent with CAGE and GRO-seq results (Figure S14) [49], which together support a regulatory role of region v.

Transcriptional activity of F10 in K562 cells correlates with little activation at F12/5′ RH, inactive TSS and F9/3′ RH areas, while CMPs show lower level of F10 activation but detectable activity at F12/5′ RH, TSS and F9/3′ RH (Figure S14). Increased activity of site F10 concomitant to reduced activation of other sites in regions ii-iv may therefore extinguish *NRAMP1* expression outside of the myelo-monocytic lineage (Table 1). Mobilization of F3 determinant in both mCD34 and CMK cells suggests it may exert a negative role as well.

Both NB4 and HL-60 show little DNAse sensitivity of site F3. Yet NB4 chromatin displays strong footprint and significant RNA Pol II binding at site F10 compared to weaker signals in HL-60 (Figure 7 and Figure S7). Limited accessibility of F10 site may thus distinguish cell types prone to express *NRAMP1* (mCD34 and HL-60 cells) whereas increased sensitivity to DNAse 1 could typify cells that do not express the gene (K562 and NB4; Figure 7).

Both F10 and F3 are weakly mobilized in CD14^+^ MNs based on DNAse footprints, histone decorations (Figure 7, Figure S15) and CAGE tags (Figure S14), which suggest reduced activation in MNs (Table 1). CAGE signals detected at F10 in blood cell types showing no appreciable levels of *NRAMP1* mRNA (e.g., MDDC and MC; cf. Section 2.2.2.1) imply that activation of F10 element may antagonize full-length gene expression.

As transcriptional activation of region v seems to vary inversely from *NRAMP1* mRNA accumulation it is plausible that myelo-monocytic fate includes relieving F10 negative influence to achieve high level *NRAMP1* expression.

#### 2.4.6. Myelo-Monocytic Programming of *NRAMP1* Gene Expression

In the first steps of hematopoiesis (CD34^+^ HSPC) chromatin at *NRAMP1* locus status is already open, bearing few suppressive histone marks; yet, though F12/5′ RH and CTCF_A-C stretch both display evidence of dynamic histone regulation, additional signals to activate expression are lacking.

AML data indicated that, prior to CMP stage, both downstream and upstream elements, F10-F3 and F6 respectively, become subjected to dynamic mobilization of modified histones (Kasumi and ME-1 cells, Figure S21) while elements F5 and F13 elements are activated subsequently (TSU-1621-MT cells, Figure S19).

Myeloid fate (mCD34/CMP) then induces significant changes principally in three regions of the locus, with balancing effects: *NRAMP1* becomes primed for expression due to activation of regions ii (elements F12, F13, F5) and iii (F7 and F1) whereas weak mobilization of region v (F10) may limit gene expression; regions i (F6) and iv (F9) also appear slightly activated compared to HSPC.

Divergent progression toward the MEP lineage (K562 and CMK cells) maintains and/or increases activation of negative elements in region v (F10 and F3) while the intragenic 3′ RH/F9 in region iv and regions i–iii are de-activated; as a result, *NRAMP1* expression becomes extinguished.

Myelo-monocytic differentiation via GMP affects *NRAMP1* locus differently depending on the promyelocytic AML model: HL-60 cells behave as expected for GMP, with concomitant activation of regions i-iii (elements F6, F5, F1); in contrast, NB4 APL seems to lock gene activation in a state intermediate between HL-60 and K562, presumably due to defective mobilization of region ii (e.g., F5), extinction of the intragenic F9/5′ RH (region iv) and persistent stimulation of region v.

In blood phagocytes, transcriptional activity of *NRAMP1* locus further increases with stimulation of all described DHSs, except in region v (F10, F3). Both regions ii and iii are prominently activated, ensuring basal expression levels in mature phagocytes. Moderate activation of the upstream region (i) as well as elements F7 and F8 in region iii suggests dependence on additional environmental stimuli and/or (epi)genetic makeup.

Hence, basal levels of *NRAMP1* mRNA appeared slightly elevated in individuals of African ancestry in both MNs and M2 MFs [54,107]. MN stimulation with bacterial compounds up-regulates gene expression: agonists of TLR4 (ultrapure LPS) and TLR2 (Pam_3_CSK_4_) both elevated *NRAMP1* mRNA levels (more intensely in individuals of European ancestry). In contrast, MN stimulation with TLR7/8 agonist (R848) and infection with influenza A virus both reduced *NRAMP1* transcript levels, and more strongly for individuals of African ancestry (down to ~50%) [107]. Hence *NRAMP1* basal expression level in MNs and/or MFs is influenced by genetics and further modulated by microbial infections. Furthermore, *NRAMP1* regulation in vitro is subjected to MN epigenetic memory that either maintains or reduces locus activation depending on the microbial stimulus [15,128].

Lastly, CD14^+^ MNs represent classical MNs (aka CD14^+^ CD16^dim^) that highly express the chemokine receptor CCR2 and migrate to sites of injury and/or infection where they differentiate into pro-inflammatory (M1) MFs. They are distinguished from non-classical MNs (aka CD14^dim^ CD16^+^); these resting MNs display prominently the adhesion-related receptor CX3CR1, contribute to vascular homeostasis, and protect against tumor metastasis in the lung. Non-classical MNs were shown to arise from classical MNs [149,150,151]. Decoration of *NRAMP1* proximal enhancer (F7) with K4me1 and K27ac marks appeared reduced, and corresponding CAGE tags less abundant, in non-classical/resting MNs (Figure S10) [152], implying that maturation toward CD16^+^ MNs may reduce *NRAMP1* expression.

Overall, with more than 8 predicted regulatory DHSs *NRAMP1* provides an example of ‘high complexity’ gene [72] even though it is part of a relatively densely populated domain of chromosome 2 (Figure S1A). Three stages in *NRAMP1* activation may be distinguished based on successive mobilization of different elements: (i) F12, F9, F10-F3 and F6 in early steps of hematopoiesis; (ii) F13, F5, F7 and F1 at the onset of myelopoiesis; (iii) F11, F4A, F2, F4B, F14 and F8 through terminal myelo-monocytic differentiation. Experimental testing of the designated regulatory areas is required to validate this hypothetical model of developmental control of *NRAMP1* gene expression, which involves both positive and negative determinants.

### 2.5. Transcriptomic Analyses of NRAMP1 and Other Genes Contributing to MF Iron Trafficking

MFs are central to body iron metabolism and numerous gene products have been implicated in the trafficking of iron by this cell-type [153,154], including: (i) receptors that capture various forms of iron: CD163 (haptoglobin-hemoglobin), LRP1/CD91 (hemopexin), LRP2/24p3R (lipocalin-2, LCN2), TFRC/TFR1 and TFR2 (holo-transferrin, TF), GAPDH (holo-lactoferrin, LTF) [155]; (ii) endosomal and phagosomal ferrireductase activities: STEAP3 [156], CYBRD1 [157]; (iii) membrane transporters facilitating metal import into the cytoplasm either from the lumen of endosomal, lysosomal, phagosomal or autophagosomal vesicles: SLC11A1/NRAMP1 and SLC11A2/DMT1 (Fe^2+^, Mn^2+^, Co^2+^), MCOLN1/TRPML1 (Ca^2+^, Fe^2+^, Zn^2+^) [158,159], SLC48A1/HRG1 (heme) [160] or at the plasma membrane: DMT1, SLC39A14/ZIP14 (Fe^2+^, Mn^2+^, Co^2+^, Zn^2+^), SLC46A1/HCP-1 and SLC49A2/FLVCR2 (heme) [161,162]; (iv) cytoplasmic iron chaperones: PCBPs [163]; (v) heme oxidase activity HMOX1/HO-1; (vi) iron storage molecules: heavy and light chain ferritins (FTH1 and FTL, respectively) and (vii) co-effector of degradation of ferritin lattice for lysosomal iron recycling: NCOA4 [164,165] as well as (viii) membrane exporters for extracellular release of iron, in the form of heme: SLC49A1/FLVCR1 [166], or as elemental iron via SLC40A1/FPN-1 for uploading onto TF aided by (ix) extracellular ferroxidase activities such as ceruleoplasmin (CP) and hephestin (HEPH) and which is controlled by the (x) regulator hepcidin (HAMP), a circulating SLC40A1 ligand that restricts body iron circulation notably in response to infection; in addition, molecules acting as (xi) iron/metal chelator and/or circulating ion transporter that also contribute to host resistance to infection include TF, LTF, LCN2 and calprotectin (CP), an antimicrobial complex secreted by inflammatory neutrophils. CP is formed by heterodimers or heterotetramers of S100A8 and S100A9 proteins that in the presence of Ca^2+^ act as potent chelators of divalent metals (Zn, Mn and Fe) [167].

To seek genes that may share expression properties with *NRAMP1* the GTEx RNA expression database [50] and epigenetic datasets hosted at UCSC [48] were interrogated to visualize and compare transcription patterns of these genes; RNA-seq data of hematopoietic cell-types from NIH Roadmap epigenomics projects were also examined [14,72] as well as those of MDMs in baseline conditions (using GM-CSF or M-CSF) and activated with 28 stimulation conditions that depict various states comprised in the current M1 vs. M2 polarization spectrum [132].

A survey of *NRAMP1* specific expression properties indicates that none of the selected genes displays similar characteristics, i.e., tissue-specific expression with high level transcript accumulation in blood, both in MNs and PMNs, abundant mRNA in lung and spleen, most potent stimulation of expression in Mac induced by M1-polarizing stimuli, and at the chromosome level, no CpG island in the TSS area but myelo-monocytic specific epigenetic marks of gene activation distributed along the locus, including a potential S-E domain (Table S1).

However, a few genes display several properties similar to *NRAMP1*. These include the locus that carries *S100A8* and *S100A9* genes whose similar characteristics (Table S1) may relate to their roles in nutritional immunity; another example is *FTH1*: despite differences in gene organization and overall tissue expression profile (*FTH1* transcript is rather ubiquitously abundant, and the gene displays a CpG island in the TSS area) *FTH1* displays high level expression in blood, spleen and lung as well as in CD14^+^ MNs (Table S2), in which K27ac decoration pattern suggests the presence of a candidate S-E domain; *FTH1* is also expressed in PMNs and MDMs. Notably, *NRAMP1* and *FTH1* display similar expression profiles in response to 28 stimuli that generate a spectrum of MF phenotypes (Figure S23A) [132].

Another gene, *NCOA4*, which is required for intracellular ferritinophagy (Fe-FTN recycling through lysosomes) displays several similarities with *FTH1* (Table S1) although *NCOA4* expression is less affected by MF polarizing compounds (Figure S23A). Yet *NCOA4* mRNA levels display variations that correspond to similar deviations in *FTH1* profile, which may suggest functional relation. Indeed, neighbor parologs *S100A8* and *S100A9*, which encode calprotectin, display quasi-identical expression profiles (Figure S23B). Hence, co-regulation of *FTH1* and *NCOA4* by fatty acids (Figure S23A) [132] may have functional significance. Likewise, *FTH1* being sole among the genes tested to display an expression profile similar to *NRAMP1*, it is tempting to deduce some functional relation as well.

Maximal mRNA accumulation for all three genes *NRAMP1*, *FTH1* and *NCOA4* results from sLPS_IFN-g treatment. *NRAMP1* mRNA is, in general, more up-regulated by M1-polarizing stimuli (from sLPS_IFN-g, left, to sLPS_IC, middle right, albeit few M0/M2 activation conditions intercalate between TNFa and IFN-g, i.e., P3C_PGE2, LiA and IL-10) [132]; the remaining inducers, at right of sLPS_IC, yield either M0 or M2 phenotypes and induce lesser up-regulation of *NRAMP1* mRNA, while stimulation with IL-4 or IL-13 preserve about half of baseline transcript level. Similar profiles of *NRAMP1* and *FTH1* mRNA abundance, up-regulated by M1 polarizing signals whereas M0/M2 conditions reduce levels closer to baseline conditions (Figure S23A) [132] thus seems consistent with dual roles in intracellular nutritional immunity (M1) and iron recycling for catabolism (M2) [168], respectively.

In contrast, five genes with properties less similar to *NRAMP1* (Figure S23C; Table S1) display maximal expression level in response to glucocorticoids (GC) [132]: *CD163* and *HMOX1* have complementary functions, i.e., uptake of hemoglobin and haptoglobin and heme degradation, respectively; *MCOLN1* and *CYBRD1* may act in conjunction in the endo-lysosomal pathway to facilitate cytoplasmic import of iron, whereas *SLC40A1* encoded FPN-1 catalyzes cell Fe export. Limited similarity in the expression profiles of these genes (i.e., sustained expression in response to M0/M2 polarizing compounds, including IL10) may relate to their anti-inflammatory activities.

Altogether, similarities in expression properties of *NRAMP1*, *FTH1* and *NCOA4* in MFs imply plausible functional links, such as Fe acquisition and sequestration (intracellular nutritional immunity, *NRAMP1* and *FTH1*) as well as ferritinophagy (*FTH1* and *NCOA4*), which may involve shared mechanisms of gene regulation (such as S-E domains, for instance).

### 2.6. Expression Studies of the Mouse Ortholog Nramp1

#### 2.6.1. *Nramp1* Locus Organization

Mouse *Nramp1* locus is generally conserved with its human counterpart: the corresponding genes are present in the same order (*Pnkd*)-*Catip*-*Slc11a1*-*Ctdsp1*. There is also similar arrangement of major Ctcf binding sites which may correspond to human CTCF sites E, A, C and D, as observed in various mouse cell types (MEL (murine erythroleukemia) cells, CH12 cells (B-cell lymphoma, GM12878 analog), bone marrow derived MFs (BMDMs) [169,170] and pluripotent stem cells induced from MFs [171]).

Yet mouse locus displays differences as well: it is more compact, ~25 kb (Figures S24 and S25; Mm10 genome assembly); it apparently lacks internal/minor Ctcf sites, and it exhibits fewer predicted regulatory determinants (Figures S26–S28; Mm9 genome assembly). Also, none of the CpG islands found at *NRAMP1* F12/5′ RH and F9/3′ RH elements seems present. These observations suggest mouse *Nramp1* may be regulated by mechanisms different from those deduced by studying *NRAMP1*.

#### 2.6.2. Regulation of Expression

*Nramp1* expression is tightly controlled during myelo-monocytic development (Figures S24–S27). It is induced during primitive and definitive hematopoieses, as indicated by different studies of cellular differentiation from either (i) pluripotent ESC, through the stages of mesoderm cell (MES), hemangioblast (HB), hemogenic endothelium (HE), hematopoietic precursor (Cd41^+^, HP) and MFs (MAC; Cd11b^+^; Figure S25) produced in vitro [172] or (ii) HSC, such as bone marrow long-term and short-term hematopoietic SC (LT-HSC and ST-HSC) generating the sequential multipotent progenitor (MPP), CMP and GMP toward PMN, MN and MF in vivo (Figure S26A, vs. MEP, erythrocytic precursors A and B, EryA and EryB, common lymphocytic precursor, CLP, CD4 and CD8 T lymphocytes, B lymphocytes and NK cells; Figure S26B) [25,173], and fetal liver HSC differentiated in vitro toward CMP, MEP and GMP [174], as well as analysis of subtypes of CMP obtained from bone marrow (Figure S27) [175].

In addition, differences were observed in *Nramp1* regulation of expression between explanted cells, including tissue MFs (such as Kupffer cells, microglia, and MFs from long and small intestine, lung and peritoneal cavity) as well as blood and bone marrow MNs and PMNs [25,120], and cells generated in vitro such as BMDM, either naïve [169,170] or stimulated with lipopolysaccharide (LPS) [176] or interferon gamma (Ifn-g; Figures S26 and S27) [173,177].

Throughout embryogenesis and post-natal development, *Nramp1* activation revealed by ATAC-seq and K4me2 decoration appeared sustained through several early stages (Figure S27): first, in pre-MFs deriving from yolk sac (YS) progenitors, which originate in blood islands and colonize the embryo at day 7.5 post-conception (E7.5) -giving rise to primitive erythroblasts, megakaryocytes and pre-MFs (~E9.5)- in which *Nramp1* locus shows a distinct pattern of activation; second, in early microglia that derive from EMPs that arose from the YS hemogenic endothelium (around E8.0–E8.5) and migrated to the fetal liver to expand and differentiate into various myeloid cell types, including early microglia that transfer to the brain (day E10.5 to E14); third, in pre-microglia that proliferate locally in the brain and disseminate within the CNS (day E14 to postnatal day P9). *Nramp1* is also expressed in adult microglia (4 weeks and onward). These data showed that in vivo hematopoiesis leads to early *Nramp1* expression in the MF and microglia lineage [137,178].

Such epigenetic data are consistent with RNA-seq studies of the specification of tissue-resident MFs during embryogenesis and organogenesis [118]. These transcriptional analyses provide a broader window on *Nramp1* expression across different tissues (Figure S24A), showing very low levels (if any) of *Nramp1* mRNA in EMP (before E10.5), beginning of accumulation in pre-Mac (E9.5) followed by increase (E10.25) to reach maximal levels in tissue resident MFs (*F4/80*^+^). *Nramp1* expression is thus more precocious than *F4/80* (*Emr1*; Figure S24B) but subsequent to *Cx3cr1* (Figure S24C) [118]. *Nramp1* transcripts are detected in most MF populations of adult tissues at levels similar to embryo tissues, with notable exceptions: skin (P8, P21) and lung (P2, P8, P21; Figure S24A). Skin results seem reminiscent of absence of *NRAMP1* expression in LCs (cf. Section 2.2.1.2). Based on these RNA-seq data, developmental induction of *Nramp1* appears part of the MF core transcriptional program and is further influenced by environmental changes in some adult tissues [118].

In vitro modeling of embryonic blood cell development further details induction of *Nramp1* expression, based on detecting co-localizing signals such as DHS and histone 3 K27ac, K9ac and K4me3 marks [172]. *Nramp1* is induced late after triggering differentiation of ESC, i.e., during the transition from Cd41^+^ HP toward mature MFs, and under the control of myelo-monocytic master regulators such as Pu.1 (both in HP and MF/MAC) and C/ebpb, Tal1 and Runx1 (in MF; Figure S25). Neither TF Pou5F1, Elk4 nor Cebpb interacted with *Nramp1* locus at the developmental stage MES; also, neither Gata2, Lmo2, Tal1 nor Cebpb bound *Nramp1* locus at HB stage; none of Meis1, Fli1, Lmo2, Tal1 or Cebpb were associated with *Nramp1* locus in HE and in HP, all the TFs tested were also negative (Gfi1, Gfib, Gata1, Gata2, Fli1, Lmo2, Tal1, Runx1 and C/ebpb; [172]). These data illustrate the tight regulation of *Nramp1* expression during myelo-monocytic differentiation [172].

In vivo, examining steps of definitive hematopoiesis further confirms late induction of *Nramp1* expression: it is primed around the CMP stage, based on co-localizing marks K27ac, K4me2 and K4me3, and further activated through GMP leading to expression in mature phagocytes (PMN, MN, Mac; Figure S26A). In contrast, *Nramp1* remains silent through the MEP pathway as well as in lymphoid lineages (Figure S26B) [25]. Regarding distinct CMP populations (CD41^+^, CD41^−^, Flt3^+^, MHC II^+^ and triple negative) [175], only CMP_MHCII, precursor of DC, showed priming of *Nramp1* with K4m2 mark decorating both elements F12- and F1-like while CMP_Flt3^+^, precursor of MN/DC, and CMP Cd41^−^ displayed weaker signals.

In vitro probing of immature stages of the myeloid lineage produced from fetal liver HSC further supports absence of *Nramp1* expression early during definitive myelopoiesis as only GMP revealed K4me2 activation mark at *Nramp1* TSS/F1-like and F7-like (Figure S27) [174]. Of note, *Nramp1* chromatin status differed between progenitors, such as CMP and GMP, either derived in vitro (Figure S27) [174] or generated in vivo (Figure S26A) [25].

Therefore, *Nramp1* myelo-monocytic specificity and timing of expression during hematopoietic development appear consistent with both previous studies of mouse *Nramp1* gene expression [179] and human data showing *NRAMP1* becomes prone to expression in bi-potential progenitors of phagocytes (cf. Section 2.4.6), such as HL-60 model cells [11,74]. Mouse studies also point epigenetic differences between progenitors that were either explanted or produced in vitro, implying important regulatory role of environmental cues.

#### 2.6.3. Predicted Regulatory Elements

Available data suggest the predicted regulatory determinants and sequence of events that leads to activate *Nramp1* expression may differ from those proposed for the human ortholog: several regulatory areas sit in locations that may correspond physically between human and mouse genes yet they may exert different roles, based on TF recruitment and local epigenetic modifications. For instance, the three Ctcf sites downstream of *Nramp1* (Figures S26–S28) may correspond to human CTCF sites A, C and D (Figure 1A), but they do not appear regulated during hematopoiesis nor influencing gene expression as observed for human *NRAMP1* (e.g., accessible sites, K9/27ac and K4me3 marks in Figure S26 compared to Figure S15).

An F12-like element, apparently behaving as a distal enhancer, is located at the end of *Catip* (Figures S26–S28). As observed with the human locus, signs of activation (e.g., K4me2) precede induction of *Nramp1* expression during in vitro differentiation (Figures S25 and S26). F12-like is the predominantly accessible element in progenitors derived in vitro compared to explanted counterparts (e.g., CMP and GMP; Figures S26 and S27). Yet, in MF derived in vitro from either ESC (Figure S25) [172] or HSC (BMDM, Figure S27) [169,170,176,177] activation marks such as K9/27ac and K4me2/3 appear reduced at this site compared to the TSS/F1-like element whose K27ac and K4me3 marking levels [169,170,176,177] (Figure S27) imply MFs express *Nramp1* [180]. In contrast, in tissue-resident MFs (peritoneum, small intestine and microglia, both embryonic and adult) and blood MNs, both K27ac and K4me2 mark *Nramp1* TSS and the end of *Catip* gene to similar extent (Figure S27) while phagocytes explanted from bone marrow display low level K27ac at F12-like element. Accordingly, mouse F12-like upstream element may be properly activated by environmental signals present in peripheral tissues only. This suggestion is supported by differences in *Nramp1* activation status between BV2 microglial cell line and primary microglia: *Nramp1* pattern of histone marks of activation in BV2 cells [181] resembles more those of BMDM (or ESCDM) vs. tissue MFs, including microglia (Figure S27). It will be important to determine whether *NRAMP1* activation is similarly controlled by environmental stimuli and/or in vitro differentiation cues, as differences in DNA methylation observed between MDM and PMN in region i may suggest (Section 2.3.1.2 and Section 2.3.2.1).

*Nramp1* TSS/F1-like area appears highly active in mouse phagocytes, as K27/9ac and K4me2/3 decorations imply a dominant regulatory role, apparently able to sustain gene expression in conditions where F12-like element is weakly activated, e.g., in vitro derived MFs (Figures S25A and S26) or MN explanted from bone marrow (Figures S25B and S26A). As noted above, such pattern contrasts with co-activation of mouse F12- and F1-like elements in tissue MFs (Figure S27).

An intronic F9-like element is also present within *Nramp1* gene, albeit in intron 9 (Figure S28). It shows moderate activity compared to TSS/F1-like in ESCDM and bone marrow Ly6C^lo^ MNs (Figure S25) as well as microglial cells (Figure S27), yet it responds remarkably to pro-inflammatory stimuli, such as Tlr4-specific agonist lipid A in BMDM, wherein it shows time-dependent transposase accessibility and association with the TFs Irf3 and RelA (Figure S28) [182]. In contrast, *NRAMP1* region iv (F14, F9) appeared less mobilized in response to microbial compounds (Figure S18). Mouse F9-like element was also mobilized during embryogenesis, specifically in primitive MFs derived from YS progenitors (Figure S27) [178]. It thus seems possible that *Nramp1* F9-like element has a different role compared to *NRAMP1* F14 and F9/3′ RH elements.

*Nramp1* also comprises an F7-like element, which is lightly mobilized and decorated compared to TSS/F1-like element (DHS, K27ac, K4me2), both in mature phagocytes and GMP (Figures S25–S27) [25,174]. F7-like shows increased mobilization in GMP derived in vitro compared to in vivo counterparts (Figures S26 and S27). Both F1- and F7-like elements bind several TFs in RN2 AML (MLL-AF9/Nras^G12D^), which represents an early stage in hematopoiesis [183]. However, *Nramp1* is probably not expressed in RN2 AML (based on temporal profile of histone decoration during hematopoiesis, Figure S26A, and the distinct patterns of K27ac mark observed in RN2 cells and BMDM; Figure S28). In addition, though Pu.1 associates with F7-like element in mature cells (MFs and MNs, Figures S25 and S28, and PMN, Figure S27), this is not the case for C/ebp [184,185,186] (Figure S28). C/ebp binds at F7-like element either in immature cells, such as RN2 cells, or in cells expressing *Nramp1* at low level, such as BMDC (MoDC, using GM-CSF [180] based on little co-localization of K27ac and K4me3 marks at the TSS [180] and the ratio of *Nramp1*/*Ctdsp1* transcription measured in BMDC and BMDM (Figure S28). These observations contrast with *NRAMP1* F7, which binds C/EBPb in late stages of the myelo-monocytic lineage and other TFs in response to pro-inflammatory stimuli (e.g., Figure S10).

Lastly, an F5-like element appears involved in response to Irf8 (both in Tot2 cells [187] and BMDC (pDC, using Flt3 ligand) [188]; Figure S28). Tot2, is a bone marrow-derived *Irf8*^−/−^ cell line [189] whose monocytic differentiation potential is restored by expressing Irf8 [187]. Mouse F5-like element also seemed mobilized in various tissue environments (e.g., K4me2 and K27ac decorations detected in MFs from small intestine; Figure S27) [120].

Examination of *Nramp1* transcriptional activation thus reveals potential elements that resemble the major determinants controlling *NRAMP1* expression but nonetheless display distinct properties, implying that different mechanisms likely regulate the mouse and human counterparts.

#### 2.6.4. TF Binding at *Nramp1* Locus

Various TFs were found associated with several areas of *Nramp1* locus, including the TSS/F1-like area that may be bound at 5′ and/or 3′ proximal sites, and which interacted with factors playing important roles in professional phagocyte lineages, such as (i) Pu.1 (MF and MN, Figure S25; PMNs, Figure S27; BMDC, RN2 AML and Irf8^+^ Tot2 cells, Figure S28, as well as ESC-derived HP, [172]); (ii) C/ebps (BMDC, MF and GMP and RN2 AML; Figures S25A and S28); (iii) Runx1 and Tal1 (MF, Figure S25A); (iv) Irf8 (constitutively bound in BMDM [177]); weak and marginal association in Irf8^+^ Tot2 cells and in BMDC, respectively, except in *Batf3^−/−^* BMDC (Figure S28); (v) Irf3 (binding induced by lipid A in BMDM, Figure S28) and Irf1 (stimulated by Ifn-g in BMDM [177]), as well as (vi) additional factors such as Erg, Fli1 and Myb (RN2 AML; Figure S28).

*Nramp1* F7-like element, which resembles *NRAMP1* proximal enhancer but seems mobilized earlier during mouse myelo-monopoiesis, was found associated with (i) Pu.1 (MFs and MNs, Figure S25; PMN, Figure S27; BMDC, Irf8^+^ Tot2 cells and RN2 AML, Figure S28, as well as HP progenitors, not shown); (ii) C/ebps (BMDC and RN2 AML; low level in GMP, Figure S28); (iii) Erg, Fli1 and Myb (RN2 AML, Figure S28); (iv) Irf8 (Irf8^+^ Tot2 cells, low level, and BMDC, marginal association unless *Batf3^−/−^* BMDC, Figure S28).

*Nramp1* F5-like element, resembling a part of *NRAMP1* S-E domain (Figure S11), was bound by Irf8 in Irf8^+^ Tot2 cells, as well as in WT but not in *Irf8*^−/−^ BMDC (Figure S28). Interestingly, in *Batf3*^−/−^ BMDC, Irf8 association with chromatin shows a binding pattern similar to Pu.1 (as observed in Tot2 Irf8^+^ cells, Figure S28, and in PMN, Figure S27), including F7- and F1-like sites instead of F5-like element, which suggests that Irf8 association with F5-like site requires Batf3 in DC [188,190]. In BMDM, Irf8 essentially associated with *Nramp1* TSS [177] yet activation of F12- and F5-like elements seemed to depend on Irf8 (Figure S28). Further comparison of TF association with *Nramp1* locus among different cell types should thus be informative.

Lastly, F12-like element binds (i) Pu.1 in Ly6c^hi^ MNs, and to lower extent in Ly6c^lo^ MNs [191] (Figure S25B) as well as in RN2 cells (Figure S28) and (ii) C/ebps in RN2 cells; Pu.1 and C/ebps may also bind F12-like in BMDC (Figure S28). Weak interactions were also detected between F12-like and NF-KB p65 and RNA Pol II in thioglycolate-elicited MFs [191].

Pu.1 and C/ebps are orthologs of key factors involved in the regulation of human *NRAMP1* (PU.1 and C/EBPb), but their respective patterns of chromatin association at *Nramp1* loci differ between species (compare Figures S25 and S28 with Figure S10). Also, human homologs of Runx1, Tal1, Erg and Fli1 bound rather distal elements in immature myeloid progenitors (e.g., F12/5′ RH and 3′ CTCF stretch; Figures S19–S22) instead of binding the TSS/F1-like area of *Nramp1* (Figures S25 and S28). These data support the possibility of divergent mechanisms regulating mammalian *Nramp1* homologs, in accordance with rapid evolution of enhancers among mammals [192].

Preferential association of TFs Pu.1, C/ebps and Irfs at F1-, F5-, F7- and F9-like areas in mouse cell types expressing *Nramp1* implies a dominant regulatory role of these cis-elements. Binding of Pu.1 at both F1- and F7-like elements has probable functional role since it correlates with high level gene expression (e.g., MF and PMN, Figure S27). The significance of C/ebp binding at F7 (Figure S28) is less clear, being observed in cells that either express *Nramp1* at low level, such as BMDCs, or do not express *Nramp1*, such as RN2 AML (Figure S28). Since F7-like element was mobilized both in bone marrow progenitors (CMP, GMP; Figure S26A) and in mature phagocytes (Figures S25, S27 and S28) it seems possible that activation of F7-like element enhances mobilization of *Nramp1* TSS/F1-like element.

Of note, RN2 cells exposed to 10 µM of C646, an inhibitor of p300 that also inhibits HDAC at high dose (i.e., >7 µM), displayed elevated decoration with H3K27ac and H4K8ac [126,193] but to levels that remained inferior to those of *Nramp1* expressing cells, implying RN2 cells lack the TFs required to express *Nramp1*. Similar observations were made at *Cd14* and *FmlpR* loci in RN2 cells, implying their block at an immature stage prevents expression of phagocyte effector functions. The corollary is that, in RN2 AML, binding of both F1- and F7-like elements by TFs such as C/ebpa, C/ebpb, Pu.1, Erg, Fli1 and Myb, together with HAT p300, is not sufficient to induce *Nramp1* expression. This suggests that additional myelo-monocytic factors such as Irf8, which regulates the production of phagocyte subsets [190], and that associates preferentially with F5-like element in BMDC and with F1-like/TSS in MFs, may be necessary to activate *Nramp1* transcription during myelopoiesis. Reduced activation of F12-like in BXH2 BMDM seems interesting in that regard [177] (Figure S26). IRF8 thus represents a candidate TF that may regulate *NRAMP1* expression in human cells as well.

Mouse BMDM short term stimulation with lipid A induced *Nramp1* F9-like element interaction with both Irf3 and RelA, whereas their association with Ctcf_A-like site decreased time-dependently. Lipid A also increased DNAse accessibility of F9-like element, and to lesser extent of F1-like/TSS site (Figure S28) [182]. These data suggest F9- and Ctcf_A-like cis-elements may regulate *Nramp1* expression in pro-inflammatory BMDM, although the significance of Irf3/RelA binding is not established yet, as *Nramp1* was not identified as a lipid A primary response gene [182], and stimulation of BMDM with LPS suggested low level mobilization of F9-like area (Figure S27) [176]. Yet another study indicated F9-like element bound NF-KB p65 in thioglycolate-elicited MF, both constitutively and in response to a Tlr4-specific agonist while the area displayed moderate histone decoration suggesting transcriptional activation (K4me2, K27ac and H4K5ac; [191]). Current data thus implicate F9-like element in the regulation of *Nramp1* expression in inflammatory MFs.

Lastly, in mice as in humans, two subsets of MNs are functionally distinguished: classical/inflammatory monocytes (Ly6C^hi^ CD43^−^) and nonclassical/patrolling MNs (Ly6C^lo^ CD43^+^) [149,150]. *Nramp1* status differs between these subsets: both the patterns of TF Pu.1 association and K27ac decoration differ at several elements (F12-, F7-, F1- and F9-like; Figure S25B) [151]: classical MNs display a pattern resembling RN2 cells (Figure S28) whereas patrolling MNs seem more similar to ESCDM (Figure S25A), blood PMN (Figure S27), BMDC and Tot *Irf8*^+^ cells (Figure S28). Whether such differences influence *Nramp1* transcriptional activation, and to what extent such regulation might be conserved in human MNs (cf. Section 2.4.6.) will require further analyses.

The determinants predicted to regulate *Nramp1* or *NRAMP1* homologs thus maintain globally similar cell-type specificity and timing of expression in mature myelo-monocytic cells, suggesting that control of gene expression during myeloid development predated mammalian divergence. However, currently available data imply the regulatory elements involved may act through specific mechanisms that differ between human and mouse. Whether evolution of different regulatory mechanisms results from intrinsic species-specific variation or pathogen-driven pressure remains to be established. Yet the data presented mean the regulation of *NRAMP1* expression cannot be inferred from mouse data but will require direct studies to decipher the regulatory mechanisms involved in human professional phagocytes.

## 3. Conclusions

*NRAMP1* differs from many inflammatory loci locked in repressed chromatin configuration in mature phagocytes until primary stimulation and training; and given the importance of STAT1 activation for the induction of trained immunity [194], *NRAMP1* epigenetic status may owe in part to constitutive binding of STAT1 at several sites of the locus [99], as well as constitutive association with the master regulators PU.1 and C/EBPb [13,25,72]. Chromatin at *NRAMP1* locus appeared generally open, even outside of the hematopoietic lineage, and local transcriptional activation occurs downstream of CD34^+^ HSPC stage in serial steps as expected for a de novo gene. Myelo-monocytic specific TFs allow activation of a candidate S-E that is key to stimulate *NRAMP1* expression in mature phagocytes. Gene expression in professional phagocytes is further up-regulated by mediators polarizing MF towards M1 and M0 phenotypes, consistent with NRAMP1 dual role in nutritional immunity and iron recycling [3,195]. Also, microbial compounds from bacterial or fungal origin upregulate *NRAMP1* transcriptional activation, yet through distinct mechanisms that induce different epigenetic memories, i.e., either immunotolerance (LPS) or trained innate immunity (BG) [15,128].

Examining the expression of genes involved in MF iron metabolism indicates some relationships with *NRAMP1*. Hence, 12 MF iron gene loci that exhibited H3K27me3 negative histone mark in MN also appeared devoid of STAT1 binding (*SLC39A14*, *TF*, *STEAP3*, *LTF*, *LCN2*, *LRP2*, *SCARA5*, *SLC40A1*, *LRP1*, *HEPH*, *PCBP3*, *PCBP4*) [99]. Another set of genes shared with *SLC11A1* binding of HIFs in MDMs subjected to hypoxia: *TFRC*, *MCOLN1*, *PCBP1*, *FTH1*, *HMOX1*, *CP* and *HAMP* [103], suggesting roles in sequestering iron away from invading microbes and in intracellular storage within ferritin.

**Figure 8:**
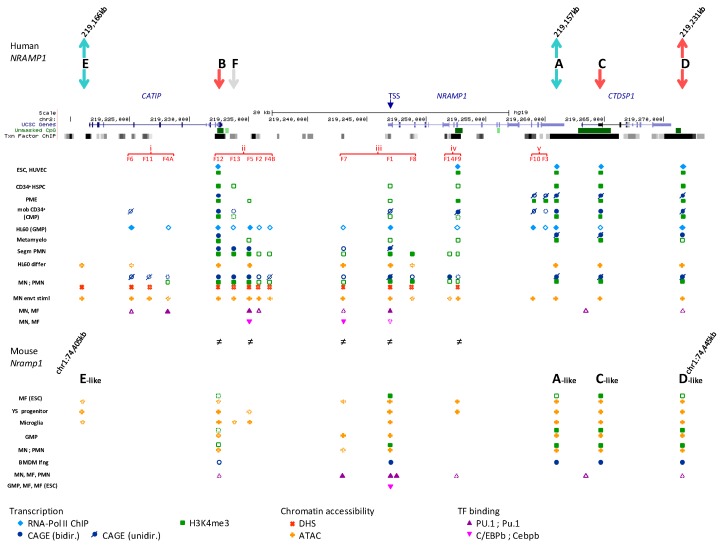
Recap of transcriptional activity, chromatin accessibility and TF binding at human and mouse *Nramp1* loci compiled for selected cell types. Human locus, from top to bottom: CTCF sites (F orientation, red; R orientation, blue; undetermined orientation, grey); coordinates above sites E, A and D indicate potential interaction sites creating regulatory loops; chr2 scale; UCSC genes (names indicated above); CpG islands; ENCODE transcription factor ChIP-seq data. Position of regulatory regions i–v, including DHS F1-F14; cell types: non hematopoietic: ESC and HUVEC; hematopoietic: CD34^+^ HSPC, mature polymorphonuclear eosinophil (PME), successive stages of myelo-monocytic differentiation, including mobilized CD34^+^ progenitors (CMP proxy), acute myeloid leukemia HL60 (GMP proxy), neutrophilic metamyelocyte (metamyelo), segmented polymorphonuclear neutrophil (segm PMN), differentiated HL60 cells (PMN, MN, MF), mature MN and PMN, and mature MN stimulated with environmental stimuli (MN envt stiml). ≠ symbols indicate that human and mouse regulatory elements are not functionally equivalent. Mouse locus, from top to bottom: Ctcf sites; hematopoietic cell types: in vitro ESC-derived MF; yolk sac (YS) MF progenitors; microglia; bone marrow GMP, MN and PMN; Ifn-gamma stimulated BMDM. Transcription related signals: CAGE tags, RNA Pol II, H3K4me3, PU.1 and C/EBPb ChIP-seq data and chromatin accessibility data: DNase 1 hypersensitive sites (DHS-seq), Tn5 transposase accessibility (ATAC-seq) are indicated with different symbols and color intensity reflects signal strength.

*NRAMP1* displays a complex pattern of transcriptional regulatory elements. DHS F12, F5, F7, F1, CTCF_A and F6 may play dominant roles, among other candidate regulatory determinants mobilized in successive steps, to regulate gene expression (recapitulated in Section 2.4.6, Table 1 and Figure S17). In contrast, distinct transcriptional regulatory determinants apparently control the mouse ortholog *Nramp1* (Figure 8). Nevertheless, *NRAMP1* and *Nramp1* share similar tissue-specific expression profiles that are restricted to mature phagocytes, and which both require the TFs PU.1 (Pu.1) and C/EBPb (C/ebpb) (Figure 8). *NRAMP1* complex regulation may thus owe in part to species-specific mechanisms.

*NRAMP1* contributes dual MF functions, either key to host defense, by withdrawing metal nutrients from microbial invaders, or to metal recycling within the host. This twofold activity, which reflects polarization of MF phenotypes along the spectrum of M1 to M0 to M2 states, may contribute to the complexity in *NRAMP1* activation, by analogy with different profiles of enhancer activation reported in Th1 vs. Th2 lymphocytes [20], even though mechanisms that differentiate Th cell subsets are not governed by combinations of lineage- and stimulus-dependent TFs such as those described in MFs [22,196]. In turn, complex regulation of *NRAMP1* expression may partly explain how SNPs may be associated with susceptibility to infections and/or autoimmune diseases in different human populations [111,197,198,199,200].

MF iron loci that apparently display similar regulatory complexity include those that may be controlled by S-Es as well as *LRP1* and *HMOX1*. About half of the human genes expressed at high level in blood, spleen and/or lung, and both in mononuclear and polymorphonuclear phagocytes, exhibit K27ac marks suggestive of potential S-E domains that may facilitate coordinated gene expression in MFs engaged in host defense and/or metal recycling. Most of these genes form with *NRAMP1* an immune regulatory network [201], and several show specifically high level expression in MNs (*NRAMP1*, *S100A9*, *FTL*, *FTH1* and *NCOA4*). Regulatory regions associated with candidate S-Es also share relatively recent origins, following gene duplications that occurred either in mammals (*FTL*, *FTH1*, *PCBP1*) or in vertebrates (*NRAMP1*, *MCOLN1*, *S100A9*, *GAPDH*) [202], which may suggest some parallel between genetic regulation and organism complexities.

Chromatin properties of candidate S-E domains predicted in CD14^+^ MNs or in spleen or lung correlate with hallmarks of *NRAMP1* locus: myelo-monocytic DNAse footprints (DHSs in HL-60 and CD14^+^ MNs), binding sites for RNA Pol II (HL-60 cells), PU.1 and/or C/EBPb (MNs and/or MDMs) as well as STAT1 in MFs, histone marks of activation and transcription (in CD14^+^ MNs and CD15^+^ PMNs), as well as highest levels of transcript accumulation in blood (*S100A9*, *FTL*) or spleen (*MCOLN1*, *NCOA4*). DHSs in CD14^+^ MNs appeared more frequently conserved in HL-60 vs. NB4 or mCD34 cells, both for genes associated with candidate S-Es and for others highly expressed in blood, spleen or lung or whose transcript accumulate both in PMNs and MNs. HL-60 cells thus represent a useful human GMP surrogate to further study the regulation of expression of genes that contribute to MF iron metabolism.

Availability of human cell models to analyze *NRAMP1* regulation may be crucial given that mouse data indicate marked differences in transcriptional regulatory mechanisms. In fact, though CTCF locus insulation, myelo-monocytic specificity and timing of gene expression appear similar between human *NRAMP1* and its mouse ortholog, as well as involvement of master regulators such as Pu.1 and C/ebps, currently available data imply the spatio-temporal determinants that control expression differ between species. Future site-specific mutagenesis analyses targeting the 14 functional determinants predicted in *NRAMP1*, and the CTCF sites bordering the locus, should thus provide critical information to gain understanding of the regulatory mechanisms that control expression of a gene that has crucial roles in nutritional immunity and/or MF metal homeostasis.

## Figures and Tables

**Table 1:** Developmental activation of transcription at *NRAMP1* locus.

CHROMOSOMAL REGIONS (Presented in Section)		i (2.4.5.2)			ii (2.4.5.1)					iii (2.4.5.3)			iv (2.4.5.4)		v (2.4.5.5)			
DNAse1 FOOTPRINTS (described in section)	5′ CTCF	F6	F11	F4A	F12	F13	F5	F2	F4B	F7	F1	F8	F14	F9	F10	F3	3′ CTCF stretch	
	(2.3.3)	(2.3.1.1)	(2.3.2.1)	(2.3.1.2)	(2.2.3.1)	(2.2.3.2)	(2.2.3.3)	(2.2.3.3)	(2.3.2.2)	(2.2.3.5)	(2.3.1.3)	(2.2.3.6)	(2.3.1.4	(2.3.1.5)	(2.3.1.6)	(2.3.1.7)	(2.3.3)	(2.3.4)
DIFFERENTIATION STAGE																		
ESC HUVEC	+	−	−	−	(+)	+/−	−	−	−	−	−	−	−	+	−	−	+	+
	+/−	−	−	−	(+)	−	−	−	−	−	−	−	−	+/−	−	−	+	+
HSPC CD34^+^ cells EP (K562 cells)	+	(+)	−	−	++	(+)	(+)	−	−	+/−	+/−	−	−	+	(+)	(+)	++	++
	+	+/−	−	−	+	−	−	−	−	−	−	−	−	−	++	+/−	++	++
ITD FLT3 AML blasts	+/−	+	−	−	+	+/−	+/−	−	−	−	−	−	−	+/−	(+)	(+)	++	+
	+/−	(+)	−	−	+	+/−	+/−	−	−	+/−	(+)	−	−	−	+/−	+/−	++	(+)
**CMP (mCD34 cells)**	+	(+)	−	−	++	(+)	+	−	−	(+)	(+)	−	−	+	(+)	(+)	++	++
GMP (NB4 cells) **GMP (HL-60 cells)**	(+)	(+)	−	−	++	+	(+)	(+)	−	−	−	−	−	−	+	+/−	++	++
	(+)	+	−	+/−	+	+/−	++	+/−	−	+/−	++	+/−	−	+/−	+/−	+/−	++	++
**MNs** **PMNs**	+	+	(+)	+/−	++	(+)	++	(+)	(+)	+	++	(+)	+/−	(+)	+/−	+/−	++	++
	+	+	(+)	+/−	++	(+)	++	(+)	(+)	+	++	(+)	+/−	(+)	+/−	+/−	++	++
BOUND FACTORS																		
MN/Mac	CTCF				(CTCF)	(CTCF)											CTCF	CTCF
							C/EBPb			C/EBPb	(C/EBPb)							
		(PU.1)		PU.1			PU.1	(PU.1)		(PU.1)	PU.1						(PU.1)	(PU.1)
		EGR2			(EGR2)					(EGR2)	EGR2				EGR2		EGR2	
Mac (hypox., ^+/−^ IL-10)							(HIF1/2a)			HIF1/2a	(HIF1/2a)							
Mac (^+/−^ LPS, IFNg)		(STAT1)	(STAT1)	STAT1		STAT1				STAT1	STAT1						STAT1	(STAT1)
							IRF1			(IRF1)	(IRF1)							

+: denotes transcriptional activation. Bold: cell-type expressing or prone to express *NRAMP1*. ESC, embryonic stem cells; HUVEC, human umbilical vein endothelial cells; HSPC, hematopoietic stem progenitor cells; CMP, common myeloid progenitors; EP, erythroid progenitors; GMP, granulocyte-MF progenitors; MN, monocytes; Mac, MFs. Transcription factors that are underlined indicate that binding was induced by treatment with LPS (lipopolysaccharide) and/or interferon gamma (IFNg).

## Supplementary Materials

Supplementary materials are available online at www.mdpi.com/2079-7737/6/2/28/s1, including Supplemental text, Figures S1–S28 and Tables S1 and S2. Correlating CAGE signals and other marks of *NRAMP1* expression in AMLs. Figure S1: CTCF-dependent topological organization of *NRAMP1* locus. Figure S2: Regulatory element in *NRAMP1* intron 12 having negative impact on gene expression. Figure S3: Detail of CAGE at *NRAMP1* region ii spanning DNASe1 footprints (DHS) F12, F13, F5, F2 and F4B. Figure S4: Detail of CAGE at *NRAMP1* region iii spanning DNASe1 footprints (DHS) F7, F1 (TSS) and F8. Figure S5: Detail of CAGE at *NRAMP1* region i spanning DNASe1 footprints (DHS) F6, F11 and F4A. Figure S6: Detail of CAGE at *NRAMP1* region iv spanning DNASe1 footprints (DHS) F14 and F9. Figure S7: Detail of CAGE at *NRAMP1* region v spanning DNASe1 footprints (DHS) F10 and F3. Figure S8: ChIP-seq data for histone marks of de-activation: K27me3 (transcriptionally silent chromatin) and K9me3 (heterochromatin). Figure S9: UCSC browser display of ENCODE TF-specific ChIP-seq data for *NRAMP1* locus shows TF association with multiple DHS, including several CTCF sites. Figure S10: Association of TF mediating inflammatory responses with *NRAMP1* locus. Figure S11: Predicted *NRAMP1* super-enhancer (S-E) determinant. Figure S12: Comparison of ChIP-seq data at *NRAMP1* locus for histone marks of chromatin activation (K4me1, K27ac and K4me3) from select cell types (ESCs, ESC-derived cultured cells) and various tissues of mesodermal origin. Figure S13: Comparison of ChIP-seq data at *NRAMP1* locus for histone marks of chromatin de-activation (K27me3 and K9me3) from select cell types (ESCs, ESC-derived cultured cells) and various tissues of mesodermal origin. Figure S14: ENCODE CAGE and RNA-seq data corresponding to *NRAMP1* locus. Figure S15: Chromatin status at *NRAMP1* locus in hematopoietic cells. Figure S16: Chromatin status at *NRAMP1* locus in myeloid cells, HUVEC and ESC. Figure S17: Hypothetical CTCF-dependent topology of *NRAMP1* locus and hematopoietic determinants of gene expression. Figure S18: *NRAMP1* locus region i and ii represent candidate transcriptional regulatory determinants mediating innate memory in response to microbial stimuli from various origins (*Saccharomyces cerevisiae* β(1,3)D-glucan, BG, and *Escherichia coli* lipopolysaccharide, LPS). Figure S19: DHS and binding of RUNX1 at *NRAMP1* locus in FLT3-ITD AML patients. Figure S20: Effect of all *trans* retinoic acid ATRA on *NRAMP1* locus activity in AML (TSU1681MT) and APL (NB4). Figure S21: *NRAMP1* activity in Kasumi AML. Figure S22: TF-specific ChIP-seq data for ME-1 AML. Figure S23: mRNA levels induced by 28 stimulation conditions producing MF phenotypes distributing across M1-M2 spectrum and ranked according to *NRAMP1* mRNA level. Figure S24: RNA-seq analysis of gene expression during mouse embryogenesis and organogenesis. Figure S25: Epigenetic activity of *Nramp1* locus during primitive or definitive hematopoiesis. Figure S26: De novo activation of *Nramp1* promoter in the myelo-monocytic lineage during definitive hematopoiesis in vivo (bone marrow). Figure S27: In vitro and in vivo regulation of *Nramp1* gene expression. Figure S28: Regulation of *Nramp1* expression in vitro: association with TFs and response to infectious stimuli. Table S1: Expression of macrophage iron genes. Table S2: Macrophage iron gene mRNA expression levels.
