# Supplementary material for: Developmental Control of NRAMP1 (SLC11A1) Expression in Professional Phagocytes
Source: Biology (Basel). 2017 May 3;6(2):28. doi: 10.3390/biology6020028 (PMC5485475; doi:10.3390/biology6020028)
Supplement: Supplementary file 1 [file biology-06-00028-s001.pdf]

Supplementary Materials: Developmental Control of *NRAMP1* (*SLC11A1*) Expression in Professional Phagocytes

Mathieu F. M. Cellier

Correlating CAGE signals and other marks of *NRAMP1* expression in AMLs.

The area spanning *NRAMP1* exons XIII-XV that comprises DHS F10 and F3 [11] is heavily decorated with K9/27ac and K4me2/3 in K562 cells, whereas CD14$^+$MNs show strong marking with K4me1 only (Figure S2B). Histone modifications thus appear consistent with CAGE data (Figure S1B), suggesting that RNA Pol II activity at the 3' end of *NRAMP1* Orf may coincide with absence of full-length gene expression in myeloid cell types either divergent from the myelo-monocytic fate (such as K562 cells) or representing some transformed, aberrant progenitor stage (e.g., NB4 APL, Figure 1). In contrast, RNA Pol II activity at *NRAMP1* TSS characterizes cells either expressing or prone to express *NRAMP1*, such as CD14$^+$MNs and HL-60 cells, respectively.

Along these lines, the area of *NRAMP1* exon XV comprises DNAse footprint F3 found in both megakaryocytic progenitors and CMPs that matches a potential binding site for ELF1 revealed by ChIP-seq (see text section 2.3.1.7.). Given ELF1 involvement in gene regulation in both erythrocytes and megakaryocytes [80, 81, 91], its peak of expression in neutrophil precursors from human bone marrow [84] and its ability to either activate transcription [86,89] or mediate transcriptional repression [79], including in MCs [82], data suggest possible cooperation between F3 element overlapping *NRAMP1* exon XV and F10 candidate alternative TSSs located in intron 12. As a result, full-length transcription of *NRAMP1* may be prevented in myeloid cell types that either represent early progenitors of microbicidal phagocytes (i.e., MNs, PMNs and PM eosinophils) or diverge toward different lineages (e.g., erythrocytic/megakaryocytic) or various cell types such as DC and MC.

The suspected *NRAMP1* alternative TSS was also detected in larger CAGE datasets: it is the sole cluster of tags detected in ENCODE CAGE dataset (82 samples, including K562 cells but no other myeloid sample) [92] while it is apparently absent from two other sets that contain data from the monocytic lineage: (FANTOM3: 120 samples representing various human tissues, hence expected to contain resident populations of macrophages, including lung which is a major site of *NRAMP1* expression, [112]) [94] and (FANTOM4: THP1 CAGE, 36 samples including both untreated and phorblol-ester stimulated cells) [93]; and it was found in FANTOM5 (1829 sample-set that includes BloodCAGE data presented in Figure 1 and Figures S3-S7) [16,17].

Further examination of *NRAMP1* expression data in THP1 monocytic cells (FANTOM4) shows that the gene is transcribed at low level in untreated cells. Gene expression is significantly induced after 24h treatment with phorblol-ester, to a level that is maintained slightly increased after 96h. These data confirm previous results obtained with HL-60 cells showing that *NRAMP1* expression is not induced rapidly during macrophage-like differentiation stimulated with phorbol ester (maximal induction attained after 48h treatment; [74]). In addition, examination of *NRAMP1* transcript level after siRNA perturbation against 53 TFs showed an up-regulation trend (twofold or more) in three instances: CTCF, IRF7 and MYC, suggesting that these factors may interfere with *NRAMP1* expression [93]. However since transcript expression was probed using the last exon (XV) the nature of the TSS involved (5′ or "3 ′alternate") is not known.

Lastly, ChIP-seq data from untreated THP1 cells [93] associated the TFs SP1 and PU.1 with both the 5′ and 3′ parts of *NRAMP1* Orf, in agreement with other results implicating SP1 in *NRAMP1* expression in VitD-differentiated HL-60 cells [12] and demonstrating PU.1 binding at *NRAMP1* locus both in MNs and MDMs [13], whereas K9ac mark and RNA Pol II were found spread along *NRAMP1* locus including the 5′ enhancer determinants. The negative element suspected in *NRAMP1* intron 12 (F10) corresponds to a peak in all four

ChIP-seq analyses, which is more prominent in the case of SP1 compared to PU.1. In contrast, CAGE tags were few at this site while tag clustering at *NRAMP1* TSS was comparatively inferior to *CTDSP1* TSS, consistent with low level *NRAMP1* expression in untreated THP1 cells.

Legend to Figures S1-S28

Figure S1. CTCF-dependent topological organization of *NRAMP1* locus. **A.** View of a 700 kb chromosome 2 segment centered on *NRAMP1* (*SLC11A1*) gene [9, 10, 48, 78]. *From top to bottom*: Orientation of CTCF sites identified by ENCODE project, forward (F) and reverse (R), and labelled with numbers or letters; Chromosome 2 scale; CTCF ChIP-seq data from $CD14^+$ MNs, K562, HUVEC and ESC; CpG islands; UCSC gene descriptions; DHS mapped in $CD14^+$ MN, HL-60, NB4, G-CSF mobilized $CD34^+$ precursors (mCD34/CMP), CMK, K562, HUVEC and ESC; Histone marks of activation (K4me1 and K27ac) in $CD14^+$ MNs, K562, HUVEC and ESC; ChIP-seq data for EGR2 (MF/MAC), PU.1 and C/EBPb (MAC and MN/MO; [13]); Histone marks of transcriptional activity (K9ac, K4me2, K4me3) in $CD14^+$ MN, K562, HUVEC and ESC; RefSeq gene descriptions. A candidate super-domain encompassing *NRAMP1* locus is indicated by a thick black line. **B.** Close-up view of CTCF-dependent looping around *NRAMP1* locus. *From top to bottom*: CTCF-dependent loops revealed by ChIA-PET analyses in both K562 and MCF7 cells; Orientation of the corresponding CTCF sites, F or R, identified by their genomic location; Chromosome 2 scale; CTCF ChIP-seq data from $CD14^+$ MN, K562, HUVEC and ESC; CpG islands; UCSC gene descriptions; DHS mapped in $CD14^+$ MN, HL-60, NB4, mCD34, CMK, K562, HUVEC and ESC; Histone marks of activation (K4me1 and K27ac) in $CD14^+$ MN, K562, HUVEC and ESC; ChIP-seq data for EGR2 (MF), PU.1 and C/EBPb (MF and MN); Histone marks of transcriptional activity (K9ac, K4me2, K4me3) in $CD14^+$ MN, K562, HUVEC and ESC; RNA-seq data for both strands from GM12878 lymphoblasts, $CD20^+$ B lymphocytes, mononuclear cells from peripheral blood, $CD14^+$ MN, K562, mCD34, HUVEC and ESC; RefSeq gene descriptions; whole cell CAGE for both strands from GM12878 lymphoblasts, $CD20^+$ B lymphocytes, $CD14^+$ MN, K562, mCD34, HUVEC and ESC. Green highlighting underlines myelo-monocytic specific transcription that typifies *NRAMP1* locus; a small contiguous area (blue highlight) delineates hematopoietic-specific transcription signals. **C.** Sequence of CTCF sites. Top: CTCF consensus sequence from various sources: JASPAR TF database [63], Factorbook database [61] and Guo *et al*. study [58]; Bottom: sequence and orientation of CTCF sites identified by ENCODE around *NRAMP1*; the three most conserved positions are underlined; # refers to CTCF site labels in panel A. **D.** Both *NRAMP1* locus CTCF boundaries comprise predicted eQTLs [54]. ***a***. Immunpop server [54] display of 416 local eQTLs associated with *NRAMP1*, based on gene expression level variations measured in M2 MF/immature DC ($CD14^+$, $CD1a^+$, $CD83^{low}$, HLA-DR^{low}), either resting or infected for two hours with Listeria or Salmonella, from individuals of European and/or African ancestry. ***b***. Blow-up of *NRAMP1* locus area (~70kb) highlighting the location of two SNPs widely used for *NRAMP1* genotyping which were previously associated with susceptibility to infectious diseases, including tuberculosis, and/or autoimmune pathologies (e.g., inflammatory bowel disease, rheumatoid arthritis [111]). Horizontal green arrows indicate eQTL haplotypes that were co-detected in independent studies [54,50]; the corresponding individual eQTLs are pointed with green arrowheads. ***c***. UCSC genome browser display of the CTCF topologically associating domain (TAD) delineating *NRAMP1* locus. *From top to bottom*: Chromosome 2 scale; CTCF binding sites revealed by ChIP-seq assays in $CD14^+$ MNs and K562 cells; CpG islands; RefSeq genes; DHS in $CD14^+$ MN (x2); ENCODE TF binding sites detected by ChIP-seq assays; UCSC genes; Chromosome 2 regions (i-v) part of *NRAMP1* locus (CTCF TAD) carrying sets of transcriptional regulatory elements (F1-F14) predicted to control gene expression.

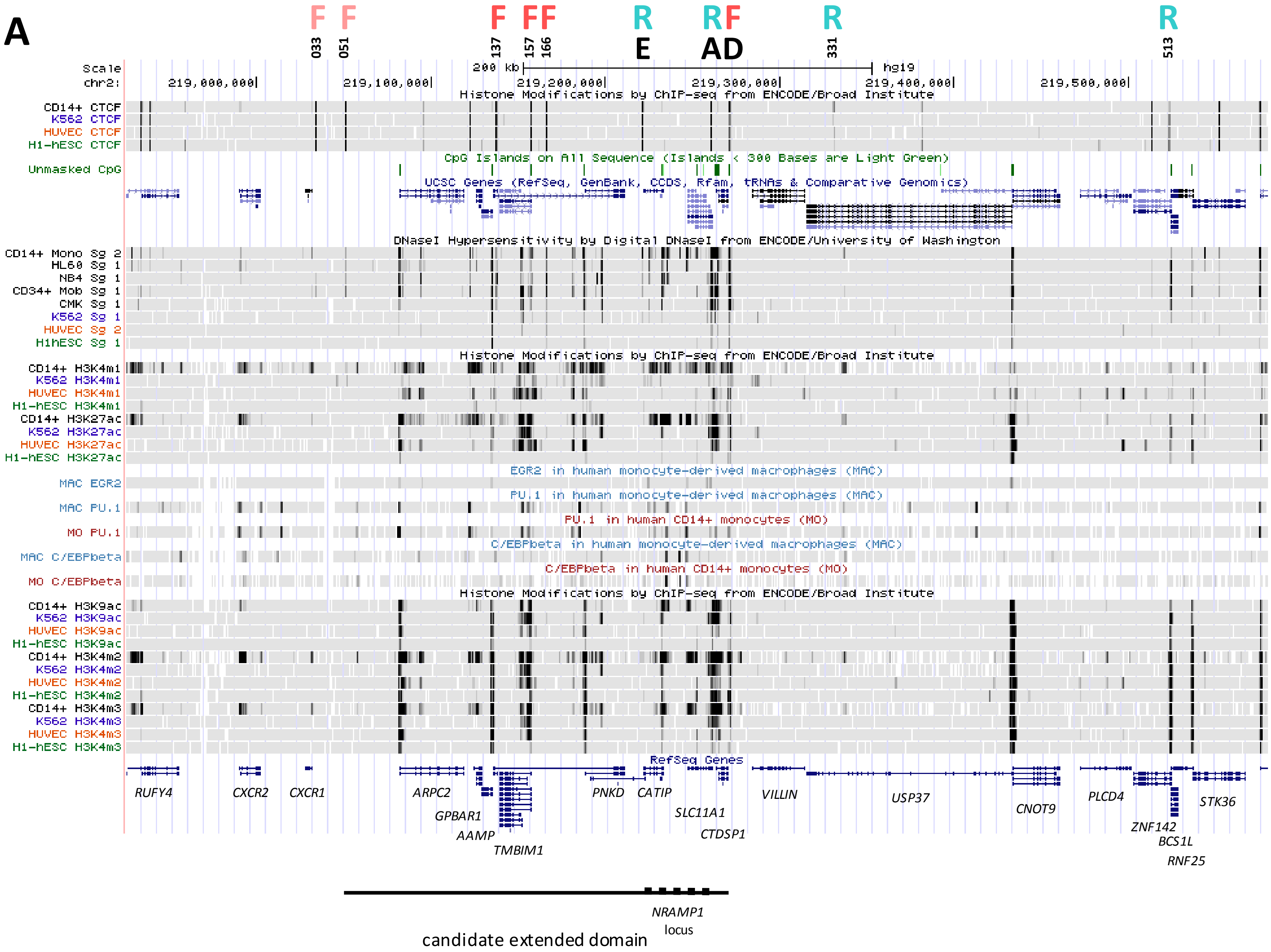
A
F F F FF R RF R R
033 051 137 157 166 331 513
E AD
Scale
chr2:
200 kb
hg19
219,000,000
219,100,000
219,200,000
219,300,000
219,400,000
219,500,000
Histone Modifications by ChIP-seq from ENCODE/Broad Institute
CD14+ CTCF
K562 CTCF
HUVEC CTCF
H1-hESC CTCF
CpG Islands on All Sequence (Islands < 300 Bases are Light Green)
Unmasked CpG
UCSC Genes (RefSeq, GenBank, CCDS, Rfam, tRNAs & Comparative Genomics)
DNaseI Hypersensitivity by Digital DNaseI from ENCODE/University of Washington
CD14+ Mono Sg 2
HL60 Sg 1
NB4 Sg 1
CD34+ Mob Sg 1
CMK Sg 1
K562 Sg 1
HUVEC Sg 2
H1hESC Sg 1
CD14+ H3K4m1
K562 H3K4m1
HUVEC H3K4m1
H1-hESC H3K4m1
CD14+ H3K27ac
K562 H3K27ac
HUVEC H3K27ac
H1-hESC H3K27ac
EGR2 in human monocyte-derived macrophages (MAC)
MAC EGR2
PU.1 in human monocyte-derived macrophages (MAC)
MAC PU.1
PU.1 in human CD14+ monocytes (MO)
MO PU.1
C/EBPbeta in human monocyte-derived macrophages (MAC)
MAC C/EBPbeta
C/EBPbeta in human CD14+ monocytes (MO)
MO C/EBPbeta
CD14+ H3K9ac
K562 H3K9ac
HUVEC H3K9ac
H1-hESC H3K9ac
CD14+ H3K4m2
K562 H3K4m2
HUVEC H3K4m2
H1-hESC H3K4m2
CD14+ H3K4m3
K562 H3K4m3
HUVEC H3K4m3
H1-hESC H3K4m3
RefSeq Genes
RUFY4
CXCR2
CXCR1
ARPC2
GPBAR1
AAMP
TMBIM1
PNKD
CATIP
SLC11A1
CTDSP1
VILLIN
USP37
CNOT9
PLCD4
ZNF142
BCS1L
RNF25
STK36
NRAMP1
locus
candidate extended domain

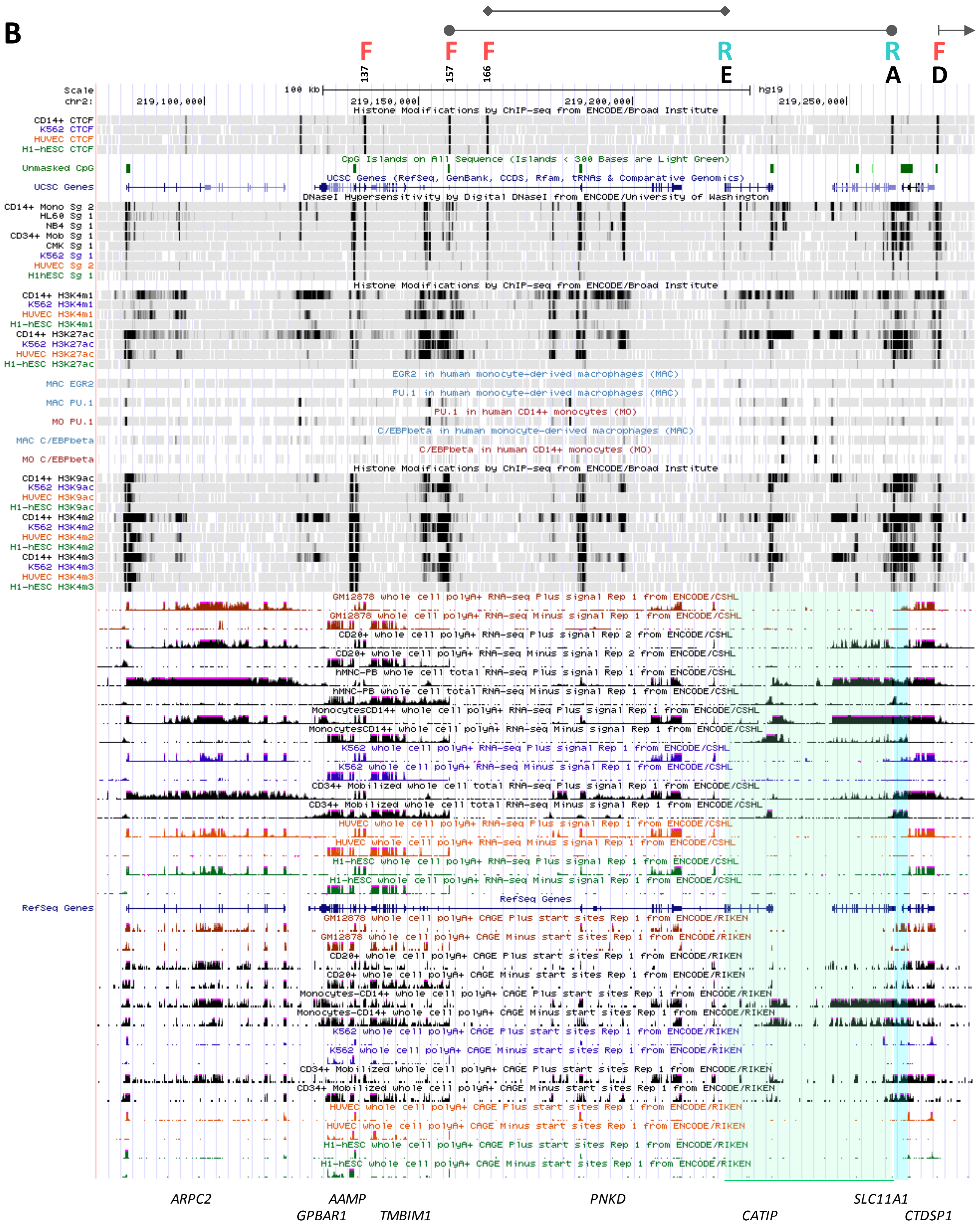
B
F
F
F
R
R
F
137
157
166
E
A
D
Scale
chr2:
100 kb
hg19
219,100,000
219,150,000
219,200,000
219,250,000
Histone Modifications by ChIP-seq from ENCODE/Broad Institute
CD14+ CTCF
K562 CTCF
HUVEC CTCF
H1-hESC CTCF
CpG Islands on All Sequence (Islands < 300 Bases are Light Green)
Unmasked CpG
UCSC Genes (RefSeq, GenBank, CCDS, Rfam, tRNAs & Comparative Genomics)
UCSC Genes
DNaseI Hypersensitivity by Digital DNaseI from ENCODE/University of Washington
CD14+ Mono Sg 2
HL60 Sg 1
NB4 Sg 1
CD34+ Mob Sg 1
CMK Sg 1
K562 Sg 1
HUVEC Sg 2
H1hESC Sg 1
Histone Modifications by ChIP-seq from ENCODE/Broad Institute
CD14+ H3K4m1
K562 H3K4m1
HUVEC H3K4m1
H1-hESC H3K4m1
CD14+ H3K27ac
K562 H3K27ac
HUVEC H3K27ac
H1-hESC H3K27ac
EGR2 in human monocyte-derived macrophages (MAC)
MAC EGR2
PU.1 in human monocyte-derived macrophages (MAC)
MAC PU.1
PU.1 in human CD14+ monocytes (MO)
MO PU.1
C/EBPbeta in human monocyte-derived macrophages (MAC)
MAC C/EBPbeta
C/EBPbeta in human CD14+ monocytes (MO)
MO C/EBPbeta
Histone Modifications by ChIP-seq from ENCODE/Broad Institute
CD14+ H3K9ac
K562 H3K9ac
HUVEC H3K9ac
H1-hESC H3K9ac
CD14+ H3K4m2
K562 H3K4m2
HUVEC H3K4m2
H1-hESC H3K4m2
CD14+ H3K4m3
K562 H3K4m3
HUVEC H3K4m3
H1-hESC H3K4m3
GM12878 whole cell polyA+ RNA-seq Plus signal Rep 1 from ENCODE/CSHL
GM12878 whole cell polyA+ RNA-seq Minus signal Rep 1 from ENCODE/CSHL
CD20+ whole cell polyA+ RNA-seq Plus signal Rep 2 from ENCODE/CSHL
CD20+ whole cell polyA+ RNA-seq Minus signal Rep 2 from ENCODE/CSHL
hMNC-PB whole cell total RNA-seq Plus signal Rep 1 from ENCODE/CSHL
hMNC-PB whole cell total RNA-seq Minus signal Rep 1 from ENCODE/CSHL
MonocytesCD14+ whole cell polyA+ RNA-seq Plus signal Rep 1 from ENCODE/CSHL
MonocytesCD14+ whole cell polyA+ RNA-seq Minus signal Rep 1 from ENCODE/CSHL
K562 whole cell polyA+ RNA-seq Plus signal Rep 1 from ENCODE/CSHL
K562 whole cell polyA+ RNA-seq Minus signal Rep 1 from ENCODE/CSHL
CD34+ Mobilized whole cell total RNA-seq Plus signal Rep 1 from ENCODE/CSHL
CD34+ Mobilized whole cell total RNA-seq Minus signal Rep 1 from ENCODE/CSHL
HUVEC whole cell polyA+ RNA-seq Plus signal Rep 1 from ENCODE/CSHL
HUVEC whole cell polyA+ RNA-seq Minus signal Rep 1 from ENCODE/CSHL
H1-hESC whole cell polyA+ RNA-seq Plus signal Rep 1 from ENCODE/CSHL
H1-hESC whole cell polyA+ RNA-seq Minus signal Rep 1 from ENCODE/CSHL
RefSeq Genes
RefSeq Genes
GM12878 whole cell polyA+ CAGE Plus start sites Rep 1 from ENCODE/RIKEN
GM12878 whole cell polyA+ CAGE Minus start sites Rep 1 from ENCODE/RIKEN
CD20+ whole cell polyA+ CAGE Plus start sites Rep 1 from ENCODE/RIKEN
CD20+ whole cell polyA+ CAGE Minus start sites Rep 1 from ENCODE/RIKEN
Monocytes-CD14+ whole cell polyA+ CAGE Plus start sites Rep 1 from ENCODE/RIKEN
Monocytes-CD14+ whole cell polyA+ CAGE Minus start sites Rep 1 from ENCODE/RIKEN
K562 whole cell polyA+ CAGE Plus start sites Rep 1 from ENCODE/RIKEN
K562 whole cell polyA+ CAGE Minus start sites Rep 1 from ENCODE/RIKEN
CD34+ Mobilized whole cell polyA+ CAGE Plus start sites Rep 1 from ENCODE/RIKEN
CD34+ Mobilized whole cell polyA+ CAGE Minus start sites Rep 1 from ENCODE/RIKEN
HUVEC whole cell polyA+ CAGE Plus start sites Rep 1 from ENCODE/RIKEN
HUVEC whole cell polyA+ CAGE Minus start sites Rep 1 from ENCODE/RIKEN
H1-hESC whole cell polyA+ CAGE Plus start sites Rep 1 from ENCODE/RIKEN
H1-hESC whole cell polyA+ CAGE Minus start sites Rep 1 from ENCODE/RIKEN
ARPC2
AAMP
GPBAR1
TMBIM1
PNKD
CATIP
SLC11A1
CTDSP1

C

CTCF consensus sites:

JASPAR (MA0139.1)

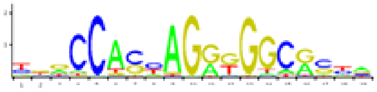

Mathelier, Fornes, Arenillas, Chen *et al*., 2016

(MA0531.1)

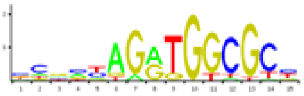

PCDHα alternate exons

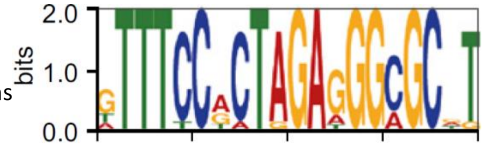

Guo, Monahan, Wu *et al*., 2012

Factorbook K562

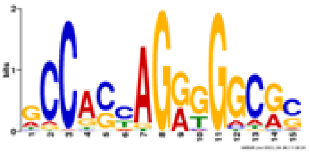

Wang, Zhuang, Iyer, Lin, Whitfield *et al*., 2012

HUVEC

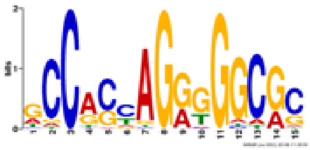

ESC

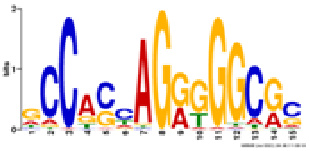

CTCF sites around *NRAMP1* locus:

	#	Strand	Orientation
GCCACAAGGGGGCAG	033	+	F
TCCAGGAGAGGGCAG	051	+	F
TCCACCAGAGGGCGC	137	+	F
GCCGCCAGGGGGCGC	157	+	F
ACCACTAGGGGGCAG	166	+	F
TCCAGCAGAGGGAGC	E	-	R
GCCCCTAGGGGGAGC	A	-	R
GCCGCTAGAGGCCTC	D	+	F
AACAGTAGGTGTCAC	331	-	R
CCCACAAGAGGGTGC	513	-	R

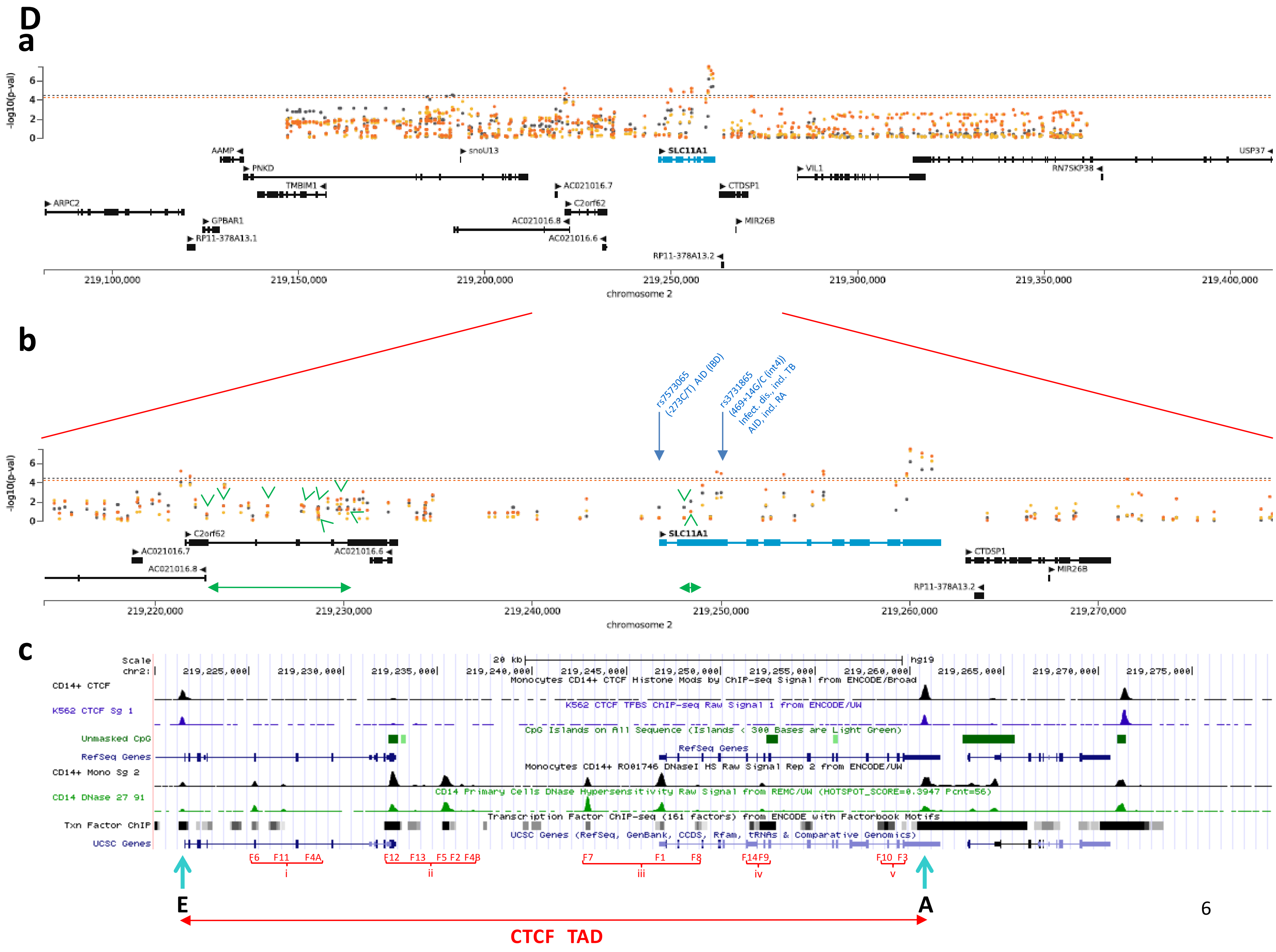

D
a
-log10(p-val)
AAMP
snoU13
SLC11A1
USP37
PNKD
VIL1
RN7SKP38
TMBIM1
AC021016.7
CTDSP1
ARPC2
C2orf62
GPBAR1
AC021016.8
MIR26B
RP11-378A13.1
AC021016.6
RP11-378A13.2
219,100,000
219,150,000
219,200,000
219,250,000
219,300,000
219,350,000
219,400,000
chromosome 2
b
rs7573065 (-273C/T) AID (IBD)
rs3731865 (469+14G/C (int4)) Infect. dis., incl. TB AID, incl. RA
219,220,000
219,230,000
219,240,000
219,250,000
219,260,000
219,270,000
chromosome 2
c
Scale
chr2:
20 kb
hg19
CD14+ CTCF
Monocytes CD14+ CTCF Histone Mods by ChIP-seq Signal from ENCODE/Broad
K562 CTCF Sg 1
K562 CTCF TFBS ChIP-seq Raw Signal 1 from ENCODE/UW
Unmasked CpG
CpG Islands on All Sequence (Islands < 300 Bases are Light Green)
RefSeq Genes
CD14+ Mono Sg 2
Monocytes CD14+ RO01746 DNaseI HS Raw Signal Rep 2 from ENCODE/UW
CD14 DNase 27 91
CD14 Primary Cells DNase Hypersensitivity Raw Signal from REMC/UW (HOTSPOT_SCORE=0.3947 Pcnt=56)
Txn Factor ChIP
Transcription Factor ChIP-seq (161 factors) from ENCODE with Factorbook Motifs
UCSC Genes
UCSC Genes (RefSeq, GenBank, CCDS, Rfam, tRNAs & Comparative Genomics)
F6 F11 F4A
i
F12 F13 F5 F2 F4B
ii
F7 F1 F8
iii
F14 F9
iv
F10 F3
v
E
A
CTCF TAD

Figure S2. Regulatory element in *NRAMP1* intron 12 having negative impact on gene expression. **A.** Cap analysis of gene expression (CAGE) at *NRAMP1* locus showing the transcription start sites (TSS) of *NRAMP1* and downstream gene *CDTSP1* whereas the upstream gene *CATIP* is not expressed in myeloid cells [68]. CAGE for representative examples of cell types that express *NRAMP1* at high level (neutrophils, CD14+ MNs), at moderate level similar to *CDTSP1* (CD34+ progenitors, dendritic cells) or that show negligible *NRAMP1* expression (acute myeloid leukemias, mast cells). The mean of tags per million (tpm) detected over the 50kb analyzed is given for each cell type to illustrate the predominance of *NRAMP1* expression in mature phagocytes. A vertical arrow indicates a putative *NRAMP1* internal TSS that is detected in cells producing little full-length transcript.

A

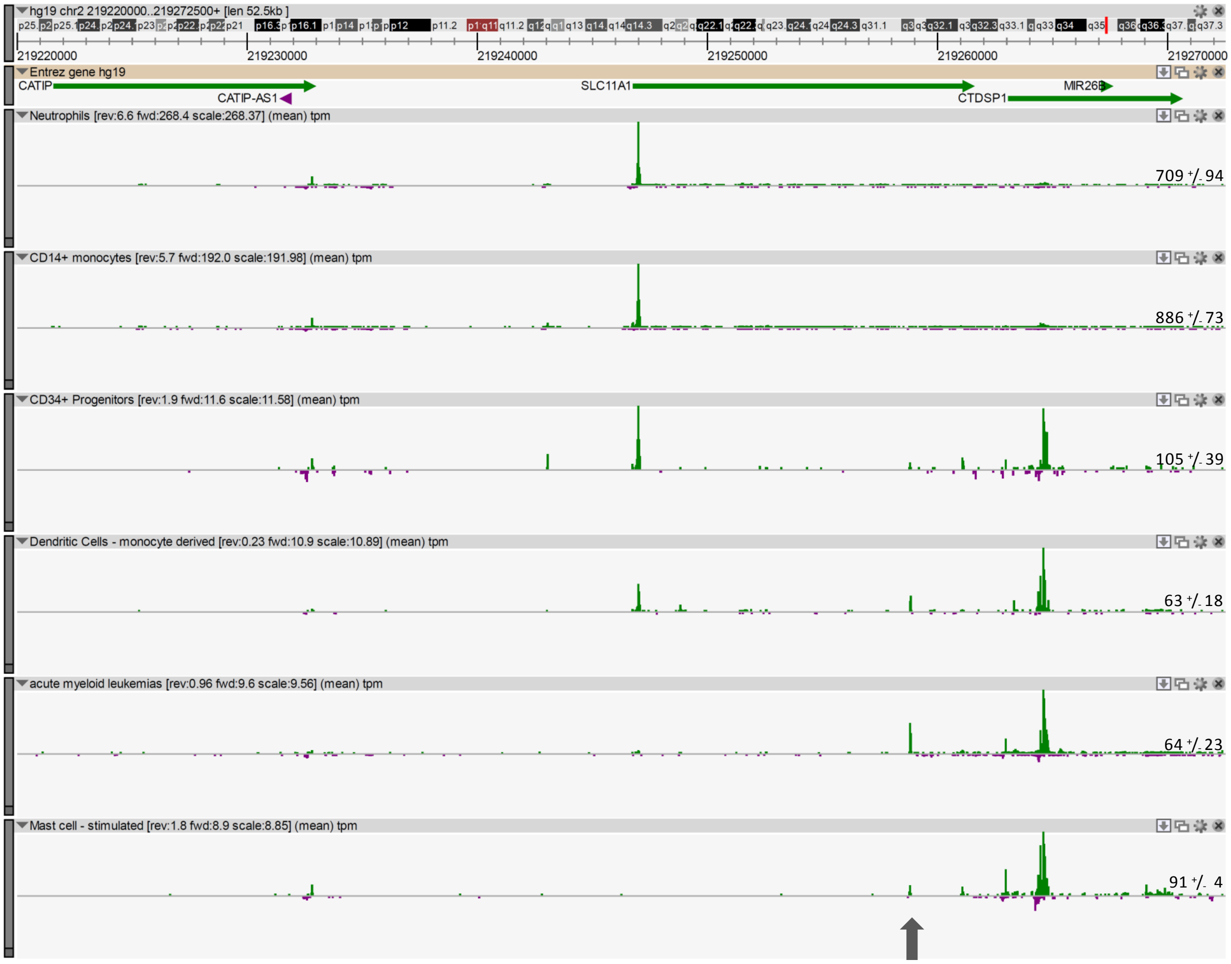

Figure S2B. (Epi)genetic analysis of the 3' end of *NRAMP1* gene (exons XIII-XV) including ChIP-seq data for specific TFs and select histone marks, and DNAse 1 footprints. *From top to bottom*: F10 and F3 denote DNAse 1 footprints previously described [11]; chromosome 2 scale; ENCODE ChIP-seq for CTCF (CD14+ MNs, K562 cells), UCSC browser description of *NRAMP1* structure; CpG islands; ENCODE ChIP-seq data for various TFs; footprints of areas sensitive to DNase1 digestion (CD14+ MNs, HL-60 and NB4 cells, mCD34, CMK and K562 cells); ChIP-seq data for select histone marks of regulatory element priming (K4me1, K27ac; CD14+ MNs, K562 cells); ChIP-seq data for the TFs EGR2, PU.1 and C/EBPb in MO and MAC [13]; ChIP-seq data for select histone marks of transcriptional activation (K9ac, K4me2, K4me3) in CD14+ MNs and K562 cells; RefSeq description of *NRAMP1* structure.

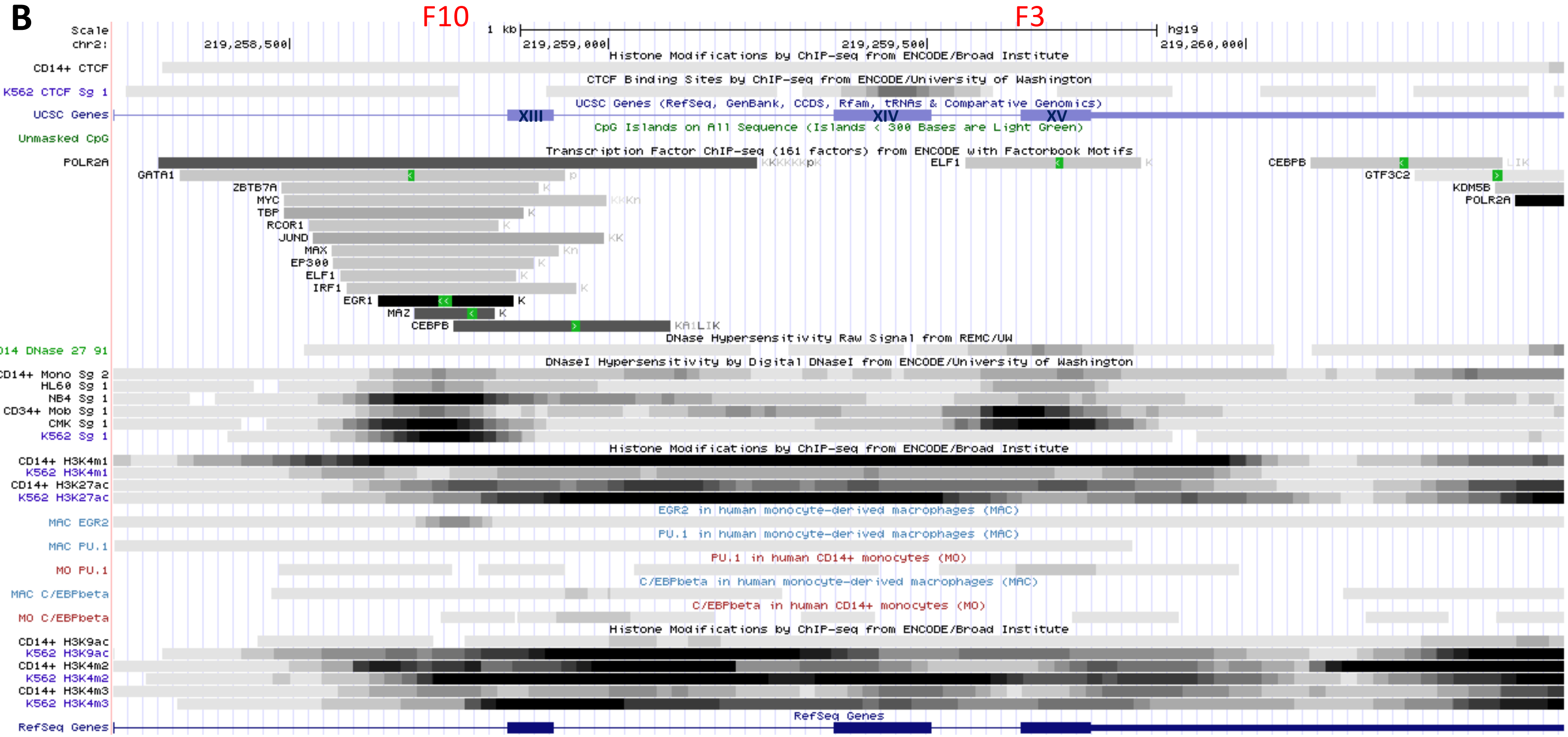

Figure S3. Detail of CAGE at *NRAMP1* region ii spanning DNASe1 footprints (DHS) F12, F13, F5, F2 and F4B. *From top to bottom*: Location of DNASe1 footprints (DHS, ENCODE); chromosome 2 scale; ENCODE CTCF ChIP-seq data for CD14[+] MNs, HL-60, NB4 and K562 cells, HUVEC and ESC; CpG islands; DNAse1 footprints in CD14[+] MNs; UCSC browser gene descriptions; RNA Pol II ChIP-seq data for HL-60, NB4, K562, HUVEC and ESC; RefSeq genes; CAGE data for 22 blood cell types [68].

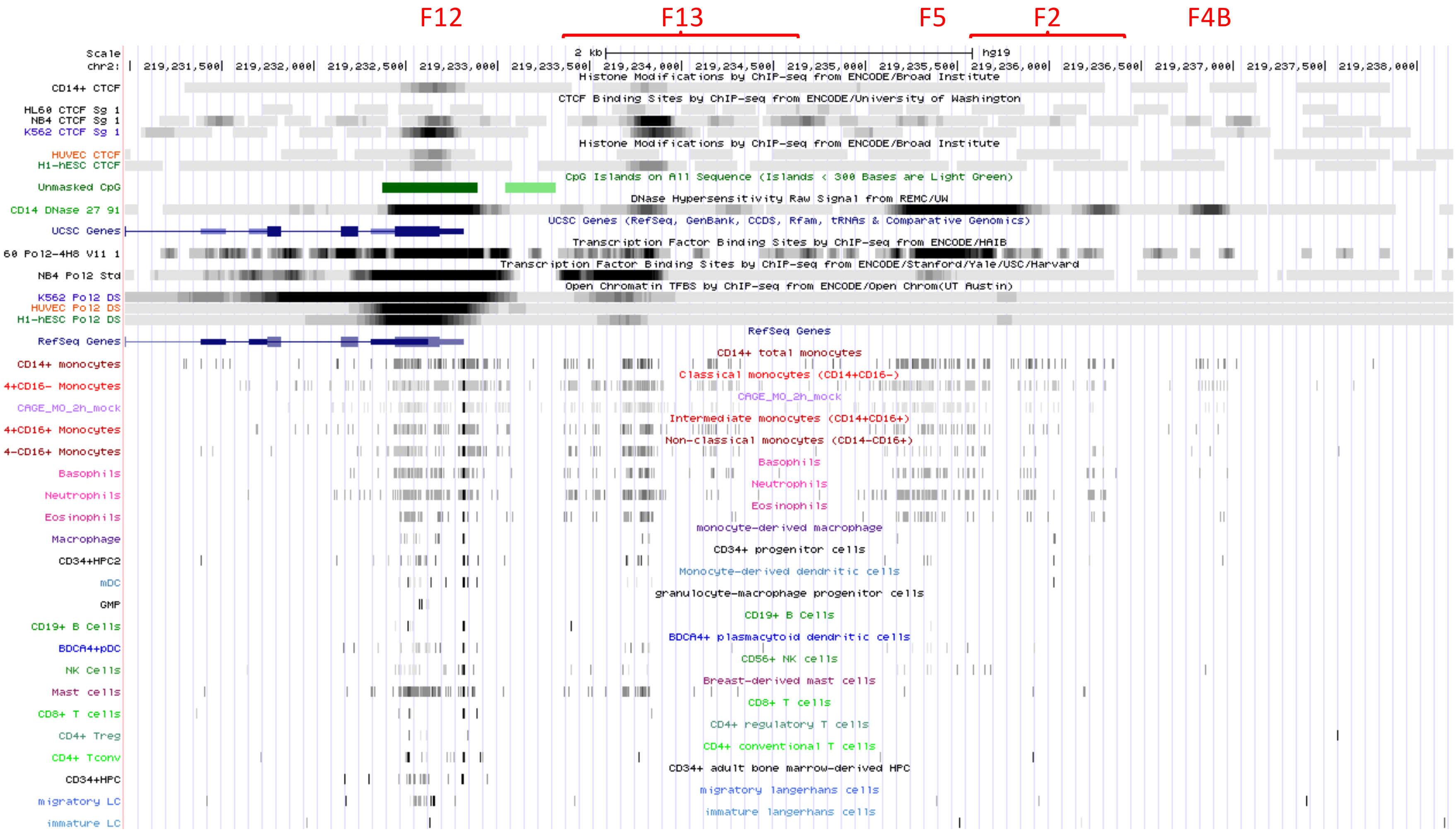

Figure S4. Detail of CAGE at *NRAMP1* region iii spanning DNASe1 footprints (DHS) F7, F1 (TSS) and F8. *From top to bottom*: same as Figure S2.

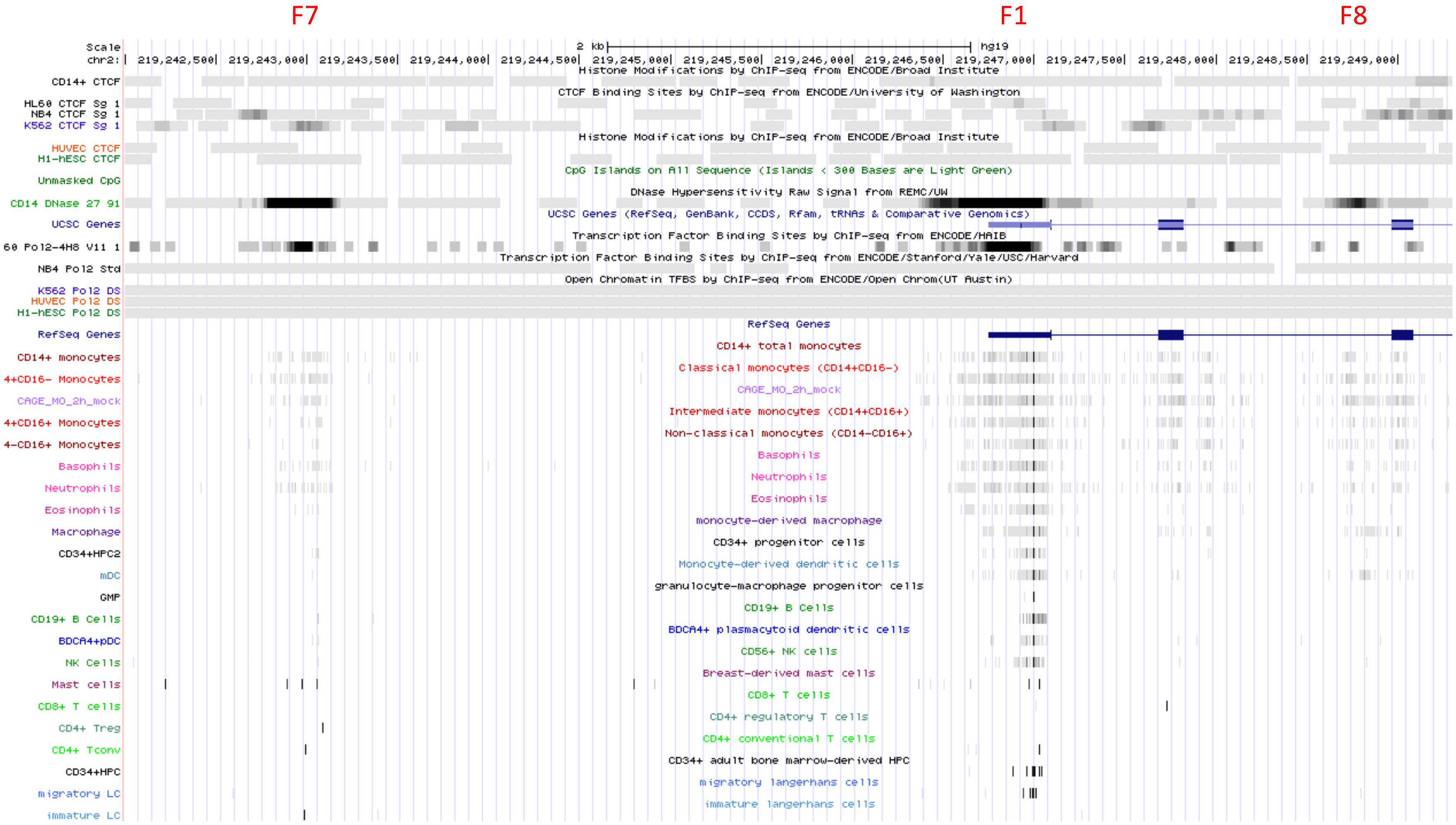

Figure S5. Detail of CAGE at *NRAMP1* region i spanning DNASe1 footprints (DHS) F6, F11 and F4A. *From top to bottom*: same as Figure S2.

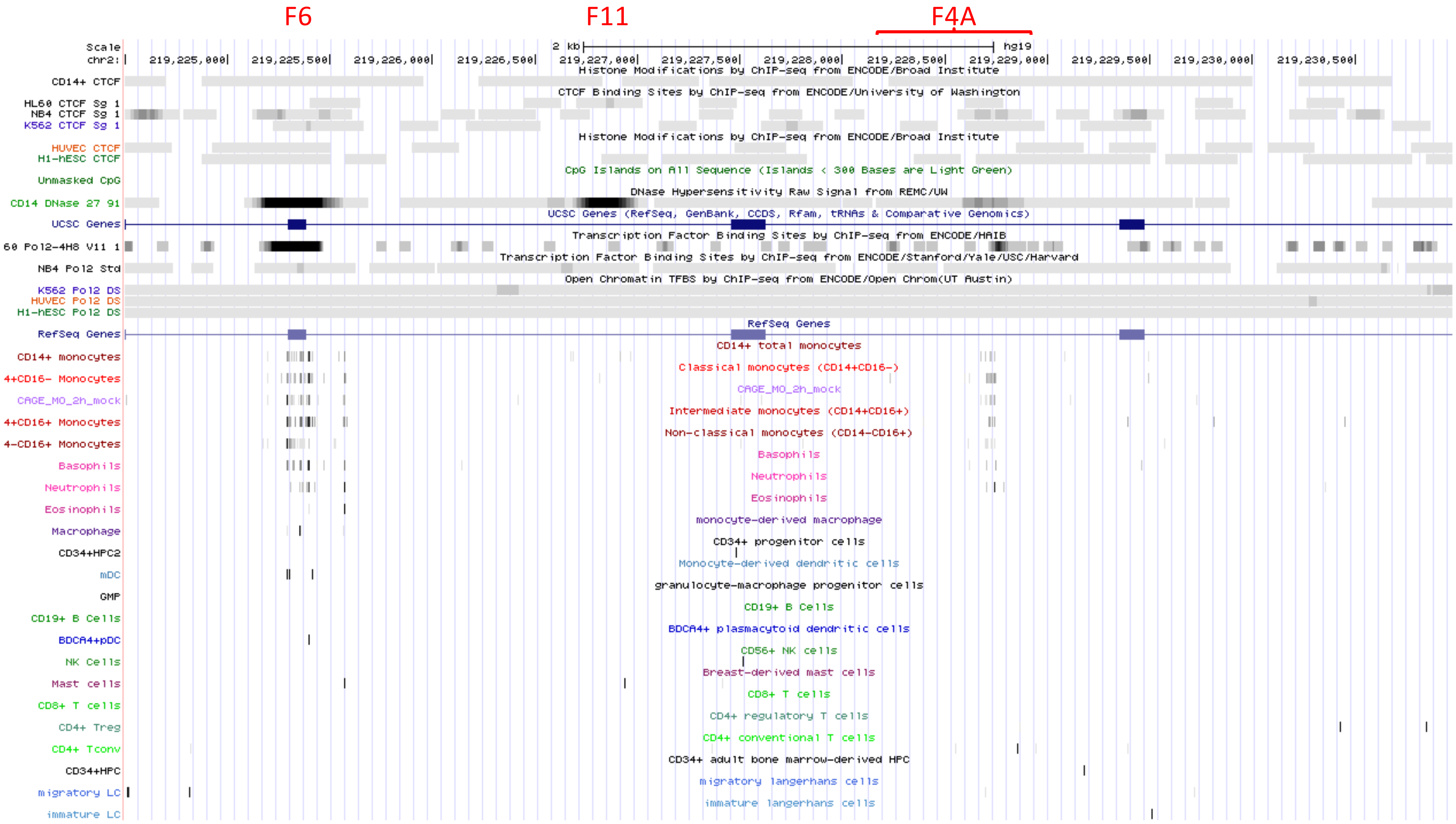

Figure S6. Detail of CAGE at *NRAMP1* region iv spanning DNASe1 footprints (DHS) F14 and F9. *From top to bottom*: same as Figure S2.

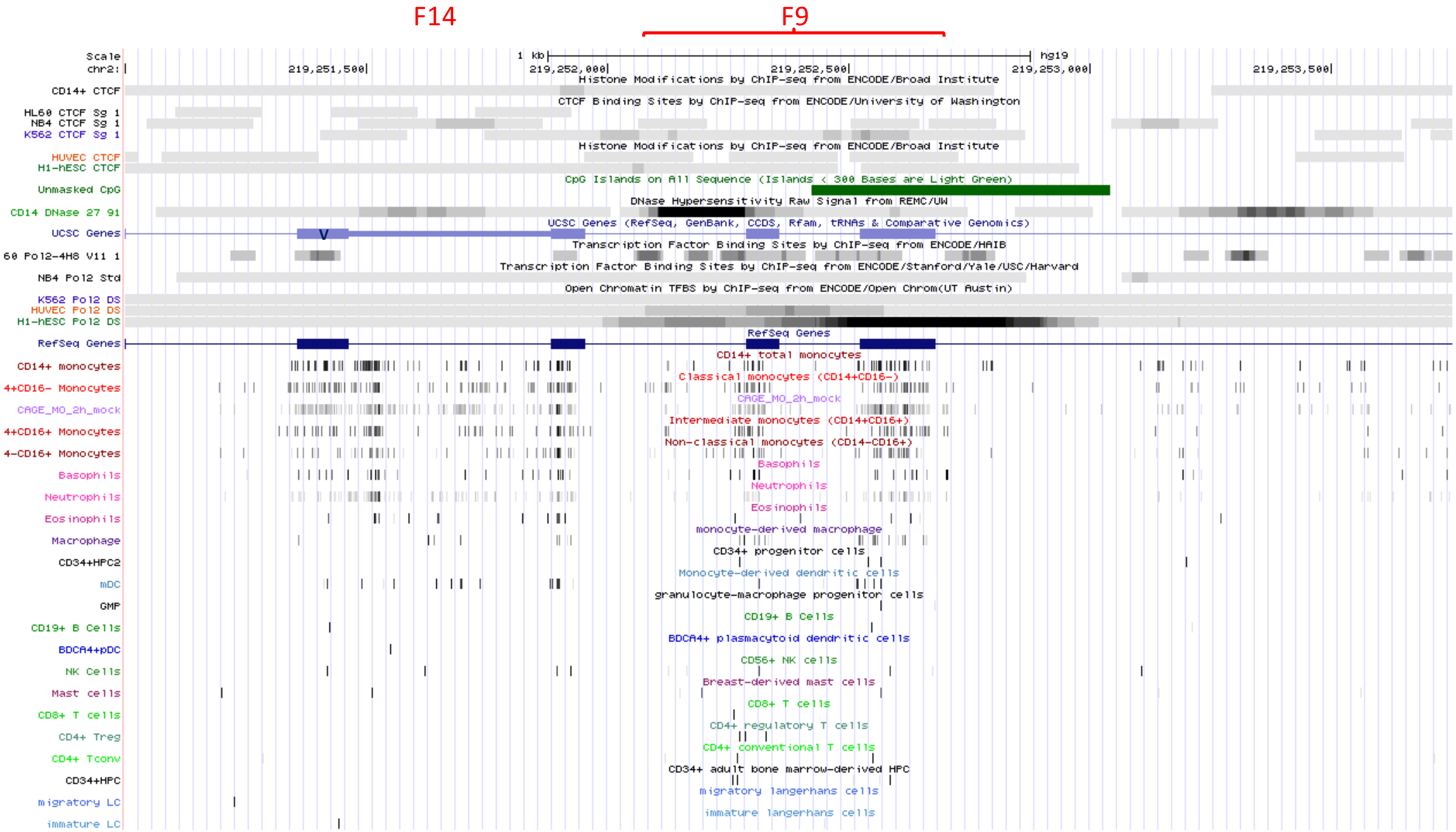

Figure S7. Detail of CAGE at *NRAMP1* region v spanning DNASe1 footprints (DHS) F10 and F3. *From top to bottom*: same as Figure S2.

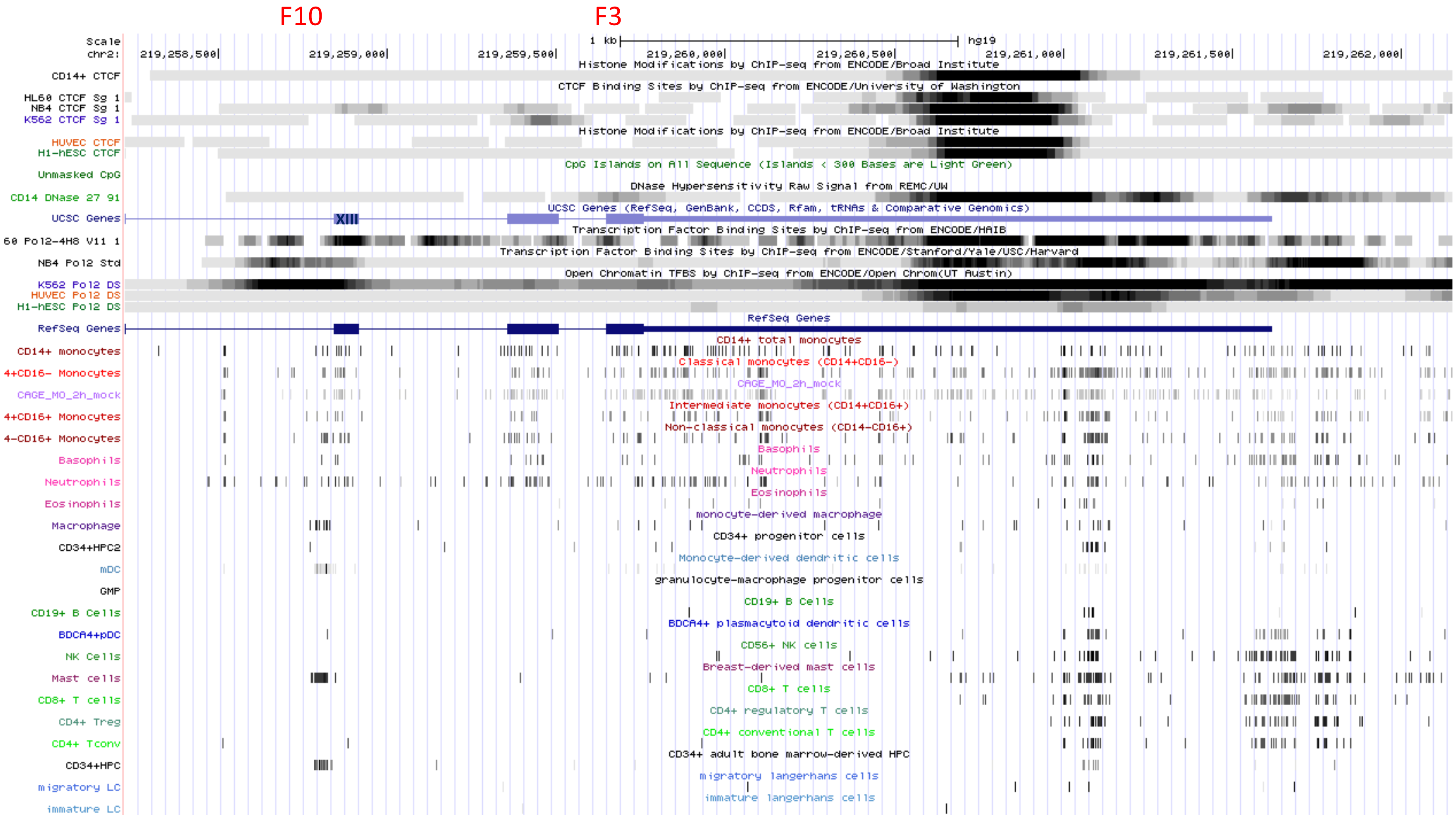

Figure S8. ChIP-seq data for histone marks of de-activation: K27me3 (transcriptionally silent chromatin) and K9me3 (heterochromatin). *NRAMP1* locus is examined at two different scales: **upper panel**, 6Mb, a vertical arrow localizes the gene; **lower panel**, 55kb, the gene is underlined. For each panel, *from top to bottom*: chromosome 2 banding pattern; local chromosome 2 scale; CpG islands; ENCODE CTCF ChIP-seq data for ESC, HUVEC, CD14+ MNs and K562 cells; UCSC gene descriptions; DNAse1 footprints for CD14+ MNs, HL-60 and NB4 cells, mCD34, CMK and K562 cells; ChIP-seq data for the TFs EGR2, PU.1 and C/EBPb in MN and MF [13]; ChIP-seq data for select histone marks of chromatin de-activation (K27me3 and K9me3; ESC, HUVEC, CD14+ MNs and K562 cells); RefSeq gene descriptions.

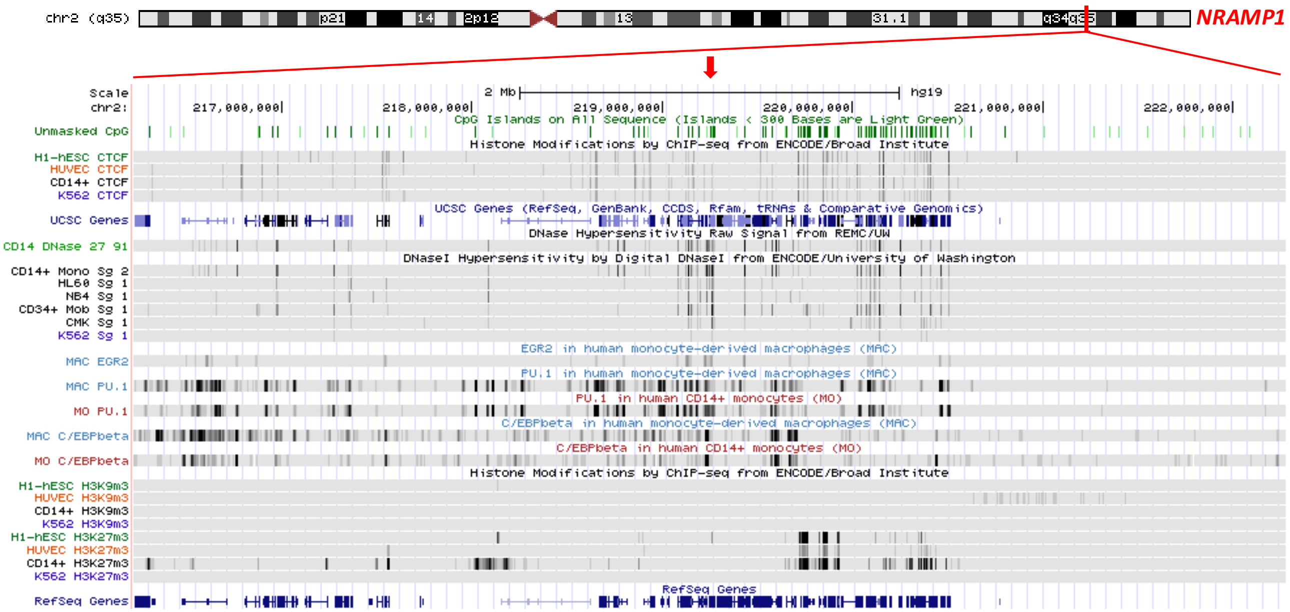

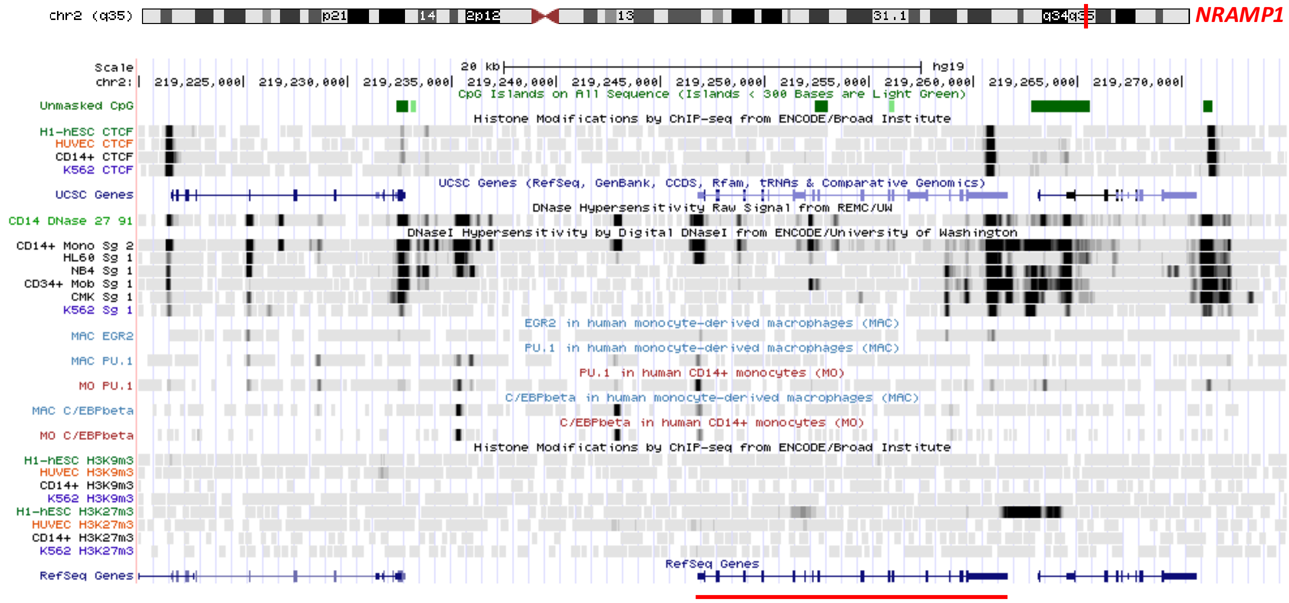

Figure S9. UCSC browser display of ENCODE TF-specific ChIP-seq data for *NRAMP1* locus shows TF association with multiple DHS, including several CTCF sites. *From top to bottom*: position and orientation of CTCF sites, indicated by colored letters: red (forward), blue (reverse) and grey (undetermined); chromosome 2 scale bar; $CD14^+$ MN DHS; CTCF-specific ChIP-seq (K562 cells); UCSC gene descriptions; CpG Islands; ENCODE TF-specific ChIP-seq data shows variable enrichment at CTCF sites; CTCF-specific ChIP-seq ($CD14^+$ MNs); $CD14^+$ MN DHS; Refseq gene descriptions.

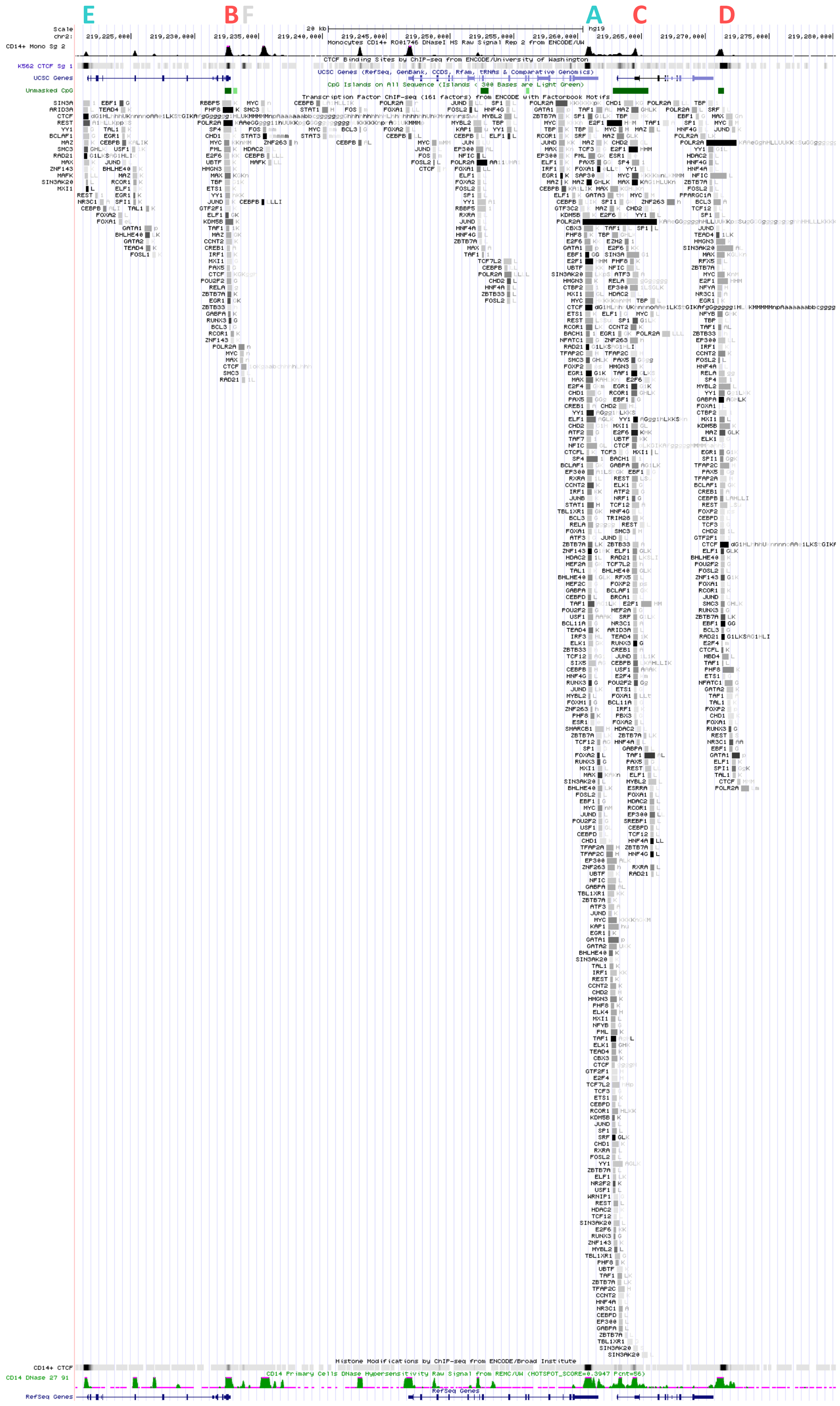
E
B
F
A
C
D
Scale
chr2:
CD14+ Mono Sg 2
K562 CTCF Sg 1
UCSC Genes
Unmasked CpG
CD14+ CTCF
CD14 DNase 27 91
RefSeq Genes

Figure S10. Association of TF mediating inflammatory responses with *NRAMP1* locus. *From top to bottom*: major CTCF binding sites E, A and D; gene names and position of *NRAMP1* TSS; chromosome 2 regions carrying predicted transcriptional regulatory elements (F1-F14); chromosome 2 scale; IRF1 ChIP-seq data for MDM (in presence of M-CSF), stimulated for 6h with or without LPS and with or without 24h IFN-g priming [99]; Other RefSeq gene descriptions; STAT1 ChIP-seq data for MDM stimulated for 6h with or without LPS and with or without 24h IFN-g priming [99]; UCSC gene descriptions; HIF1a and HIF2a association with *NRAMP1* locus in MDM (RPMI medium alone) exposed to hypoxia for 4h at 37°C after priming or not with IL-10 for 16h [103]; Basic gene depictions; ChIP-seq for EGR2 (MF), C/EBPb and PU.1 (MN and MF) [13]; Ensembl Genes; ENCODE DHS for $CD14^+$ MNs, HL-60, NB4, K562 and CMK cells, mCD34, $CD34^+$ progenitors; CTCF specific ChIP-seq in $CD14^+$ MNs; K27ac specific ChIP-seq data for MN and MF [13], $CD14^+$ $CD16^-$ MNs and $CD14^-$ $CD16^+$ MNs [152], $CD14^+$ MNs and $CD34^+$ progenitors (ENCODE); CTCF specific ChIP-seq in ESC; K4me1 specific ChIP-seq data for MO and MAC [13], $CD14^+$ MNs, $CD34^+$ progenitors and HSC (ENCODE); CpG islands; K9ac ChIP-seq data ($CD14^+$ MNs); CTCF specific ChIP-seq in K562 cells; K4me2 and me3 ChIP-seq ($CD14^+$ MNs); RefSeq genes; ChIP-seq in $CD14^+$ MNs (H4K20me1, K79me2, K36me3); CTCF specific ChIP-seq in HUVEC; CAGE data for $CD14^+$ $CD16^-$ MNs and $CD14^-$ $CD16^+$ MNs [152].

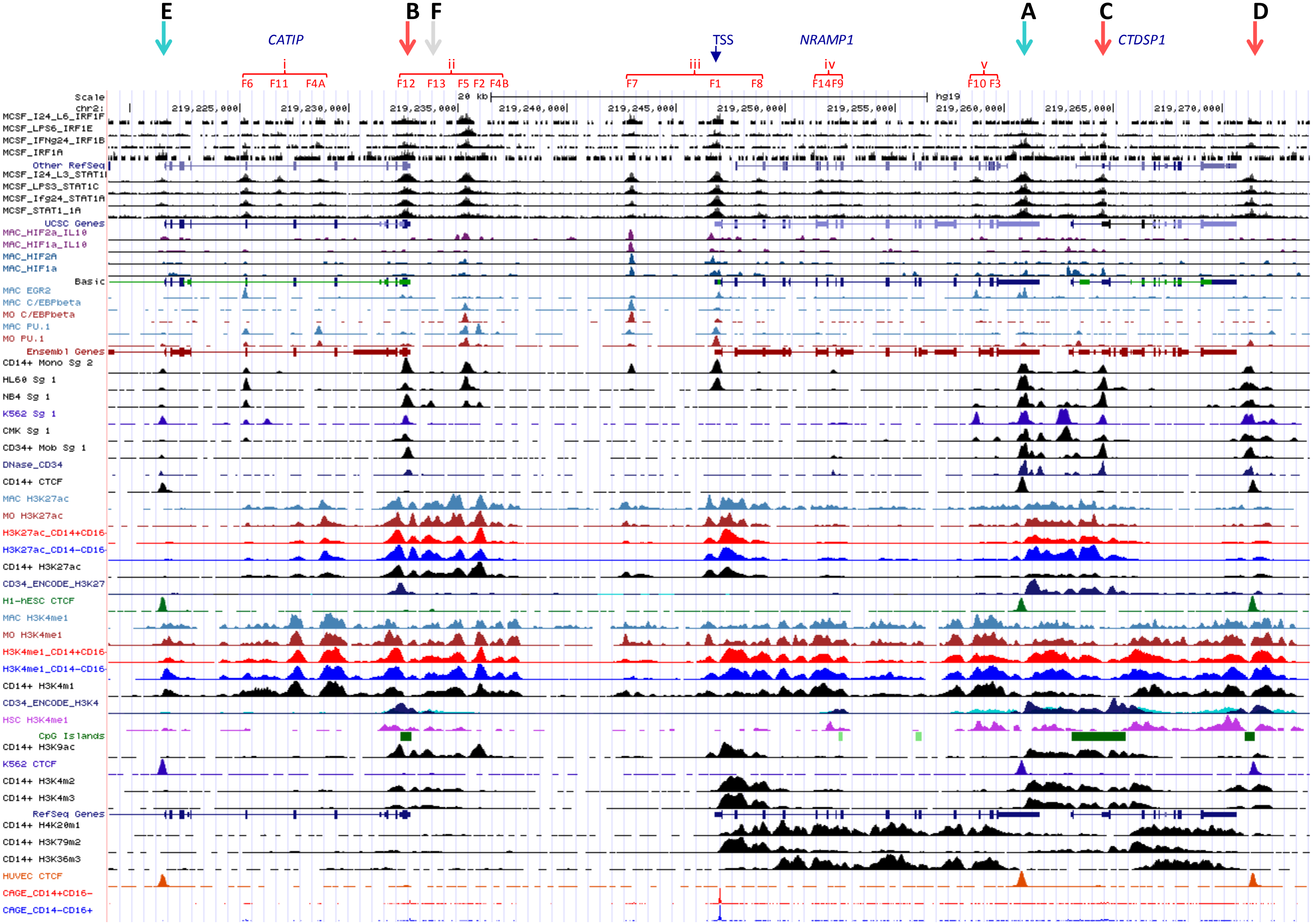

E
B F
A
C
D
CATIP
TSS
NRAMP1
CTDSP1
i
ii
iii
iv
v
F6 F11 F4A
F12 F13 F5 F2 F4B
F7 F1 F8
F14 F9
F10 F3
Scale
chr2:
20 kb
hg19
219,225,000
219,230,000
219,235,000
219,240,000
219,245,000
219,250,000
219,255,000
219,260,000
219,265,000
219,270,000
MCSF_I24_L6_IRF1F
MCSF_LPS6_IRF1E
MCSF_IFNg24_IRF1B
MCSF_IRF1A
Other RefSeq
MCSF_I24_L3_STAT1
MCSF_LPS3_STAT1C
MCSF_Ifg24_STAT1A
MCSF_STAT1_1A
UCSC Genes
MAC_HIF2a_IL10
MAC_HIF1a_IL10
MAC_HIF2A
MAC_HIF1a
Basic
MAC EGR2
MAC C/EBPbeta
MO C/EBPbeta
MAC PU.1
MO PU.1
Ensembl Genes
CD14+ Mono Sg 2
HL60 Sg 1
NB4 Sg 1
K562 Sg 1
CMK Sg 1
CD34+ Mob Sg 1
DNase_CD34
CD14+ CTCF
MAC H3K27ac
MO H3K27ac
H3K27ac_CD14+CD16-
H3K27ac_CD14-CD16+
CD14+ H3K27ac
CD34_ENCODE_H3K27
H1-hESC CTCF
MAC H3K4me1
MO H3K4me1
H3K4me1_CD14+CD16-
H3K4me1_CD14-CD16+
CD14+ H3K4m1
CD34_ENCODE_H3K4
HSC H3K4me1
CpG Islands
CD14+ H3K9ac
K562 CTCF
CD14+ H3K4m2
CD14+ H3K4m3
RefSeq Genes
CD14+ H4K20m1
CD14+ H3K79m2
CD14+ H3K36m3
HUVEC CTCF
CAGE_CD14+CD16-
CAGE_CD14-CD16+

Figure S11. Predicted *NRAMP1* super-enhancer (S-E) determinant. *From top to bottom*: Chromosome 2 scale; ChIP-seq for CTCF ($CD14^+$ MNs, K562 cells, ESC); CpG islands; UCSC gene description; DHS ($CD14^+$ MNs, HL-60 and NB4 cells, mCD34, CMK and K562 cells); ChIP-seq data (K27ac, K4me1 and the TFs PU.1 and C/EBPb in MN and MF [13]; polyA+ CAGE tags for $CD14^+$ MNs, mCD34, K562 cells, ESC [16,17]; RefSeq genes; dbSUPER prediction of S-E determinants in $CD14^+$ MNs, K562 cells and $CD34^+$ precursors [101]

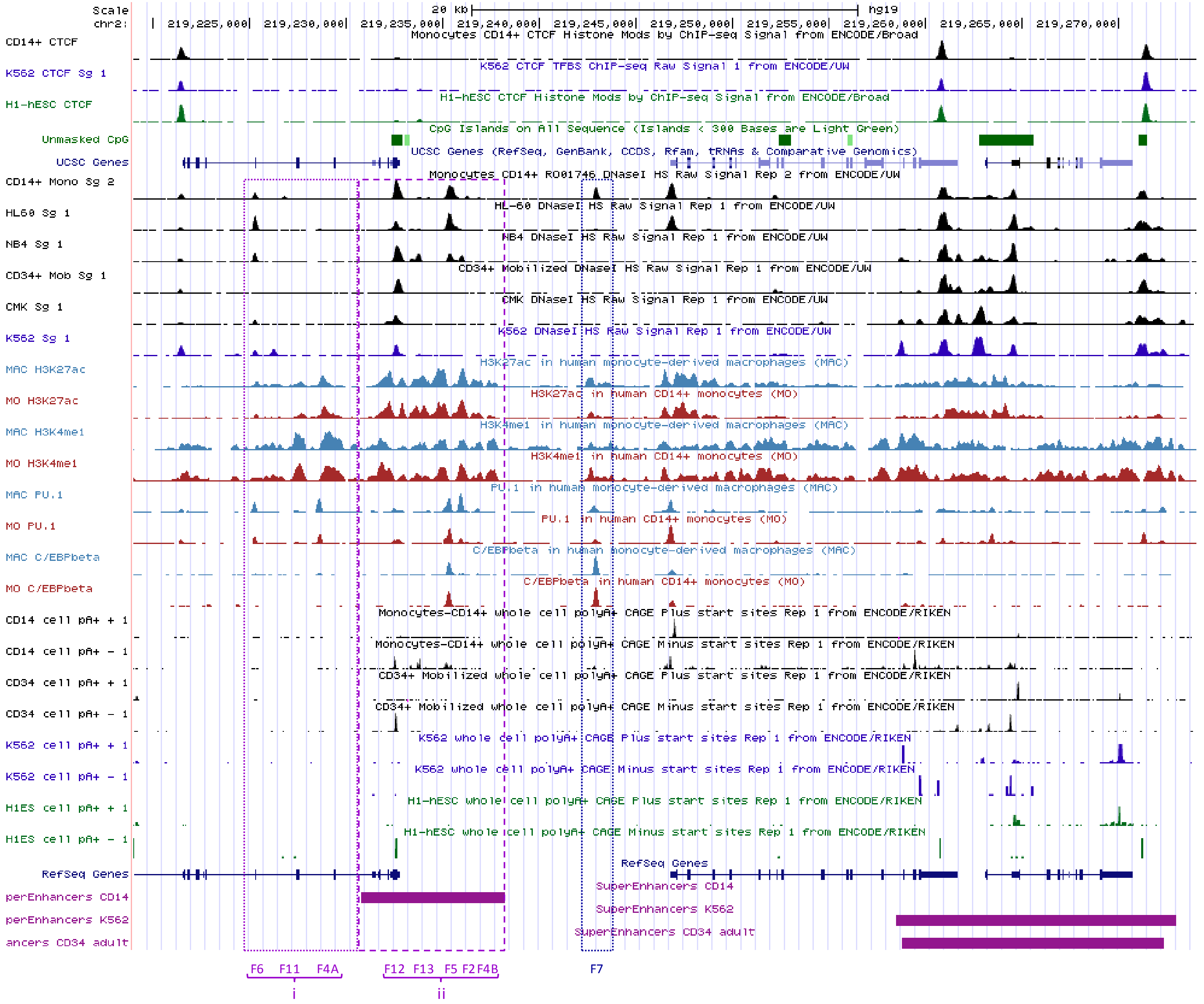

Scale
chr2:
20 kb
hg19
219,225,000| 219,230,000| 219,235,000| 219,240,000| 219,245,000| 219,250,000| 219,255,000| 219,260,000| 219,265,000| 219,270,000|
Monocytes CD14+ CTCF Histone Mods by ChIP-seq Signal from ENCODE/Broad
K562 CTCF TFBS ChIP-seq Raw Signal 1 from ENCODE/UW
H1-hESC CTCF Histone Mods by ChIP-seq Signal from ENCODE/Broad
CpG Islands on All Sequence (Islands < 300 Bases are Light Green)
UCSC Genes (RefSeq, GenBank, CCDS, Rfam, tRNAs & Comparative Genomics)
Monocytes CD14+ RO01746 DNaseI HS Raw Signal Rep 2 from ENCODE/UW
HL-60 DNaseI HS Raw Signal Rep 1 from ENCODE/UW
NB4 DNaseI HS Raw Signal Rep 1 from ENCODE/UW
CD34+ Mobilized DNaseI HS Raw Signal Rep 1 from ENCODE/UW
CMK DNaseI HS Raw Signal Rep 1 from ENCODE/UW
K562 DNaseI HS Raw Signal Rep 1 from ENCODE/UW
H3K27ac in human monocyte-derived macrophages (MAC)
H3K27ac in human CD14+ monocytes (MO)
H3K4me1 in human monocyte-derived macrophages (MAC)
H3K4me1 in human CD14+ monocytes (MO)
PU.1 in human monocyte-derived macrophages (MAC)
PU.1 in human CD14+ monocytes (MO)
C/EBPbeta in human monocyte-derived macrophages (MAC)
C/EBPbeta in human CD14+ monocytes (MO)
Monocytes-CD14+ whole cell polyA+ CAGE Plus start sites Rep 1 from ENCODE/RIKEN
Monocytes-CD14+ whole cell polyA+ CAGE Minus start sites Rep 1 from ENCODE/RIKEN
CD34+ Mobilized whole cell polyA+ CAGE Plus start sites Rep 1 from ENCODE/RIKEN
CD34+ Mobilized whole cell polyA+ CAGE Minus start sites Rep 1 from ENCODE/RIKEN
K562 whole cell polyA+ CAGE Plus start sites Rep 1 from ENCODE/RIKEN
K562 whole cell polyA+ CAGE Minus start sites Rep 1 from ENCODE/RIKEN
H1-hESC whole cell polyA+ CAGE Plus start sites Rep 1 from ENCODE/RIKEN
H1-hESC whole cell polyA+ CAGE Minus start sites Rep 1 from ENCODE/RIKEN
RefSeq Genes
SuperEnhancers CD14
SuperEnhancers K562
SuperEnhancers CD34 adult
CD14+ CTCF
K562 CTCF Sg 1
H1-hESC CTCF
Unmasked CpG
UCSC Genes
CD14+ Mono Sg 2
HL60 Sg 1
NB4 Sg 1
CD34+ Mob Sg 1
CMK Sg 1
K562 Sg 1
MAC H3K27ac
MO H3K27ac
MAC H3K4me1
MO H3K4me1
MAC PU.1
MO PU.1
MAC C/EBPbeta
MO C/EBPbeta
CD14 cell pA+ + 1
CD14 cell pA+ - 1
CD34 cell pA+ + 1
CD34 cell pA+ - 1
K562 cell pA+ + 1
K562 cell pA+ - 1
H1ES cell pA+ + 1
H1ES cell pA+ - 1
RefSeq Genes
perEnhancers CD14
perEnhancers K562
ancers CD34 adult
F6 F11 F4A
i
F12 F13 F5 F2 F4B
ii
F7

Figure S12. Comparison of ChIP-seq data at *NRAMP1* locus for histone marks of chromatin activation (K4me1, K27ac and K4me3) from select cell types (ESCs, ESC-derived cultured cells) and various tissues of mesodermal origin [14]. Each panel is depicted using a common Y-scale. *NRAMP1* predicted 5' and 3' regulatory hubs (RH) are highlighted (red and brown, respectively).

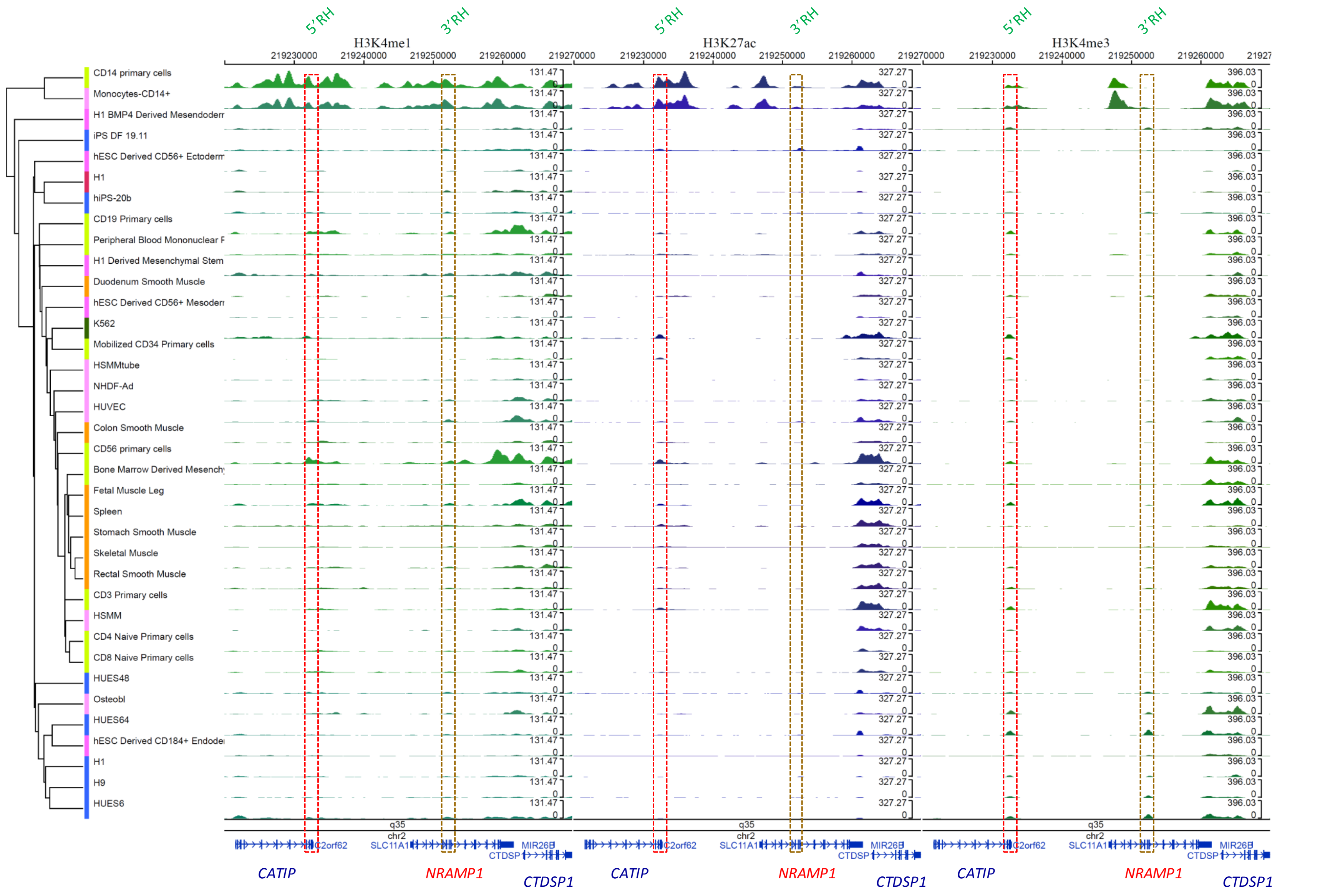

Figure S13. Comparison of ChIP-seq data at *NRAMP1* locus for histone marks of chromatin de-activation (K27me3 and K9me3) from select cell types (ESCs, ESC-derived cultured cells) and various tissues of mesodermal origin [14]. Each panel is depicted using a common Y-scale. *NRAMP1* predicted 5' and 3' regulatory hubs (RH) are highlighted (red and brown, respectively). K4me1 mark added for comparison with Figure S12.

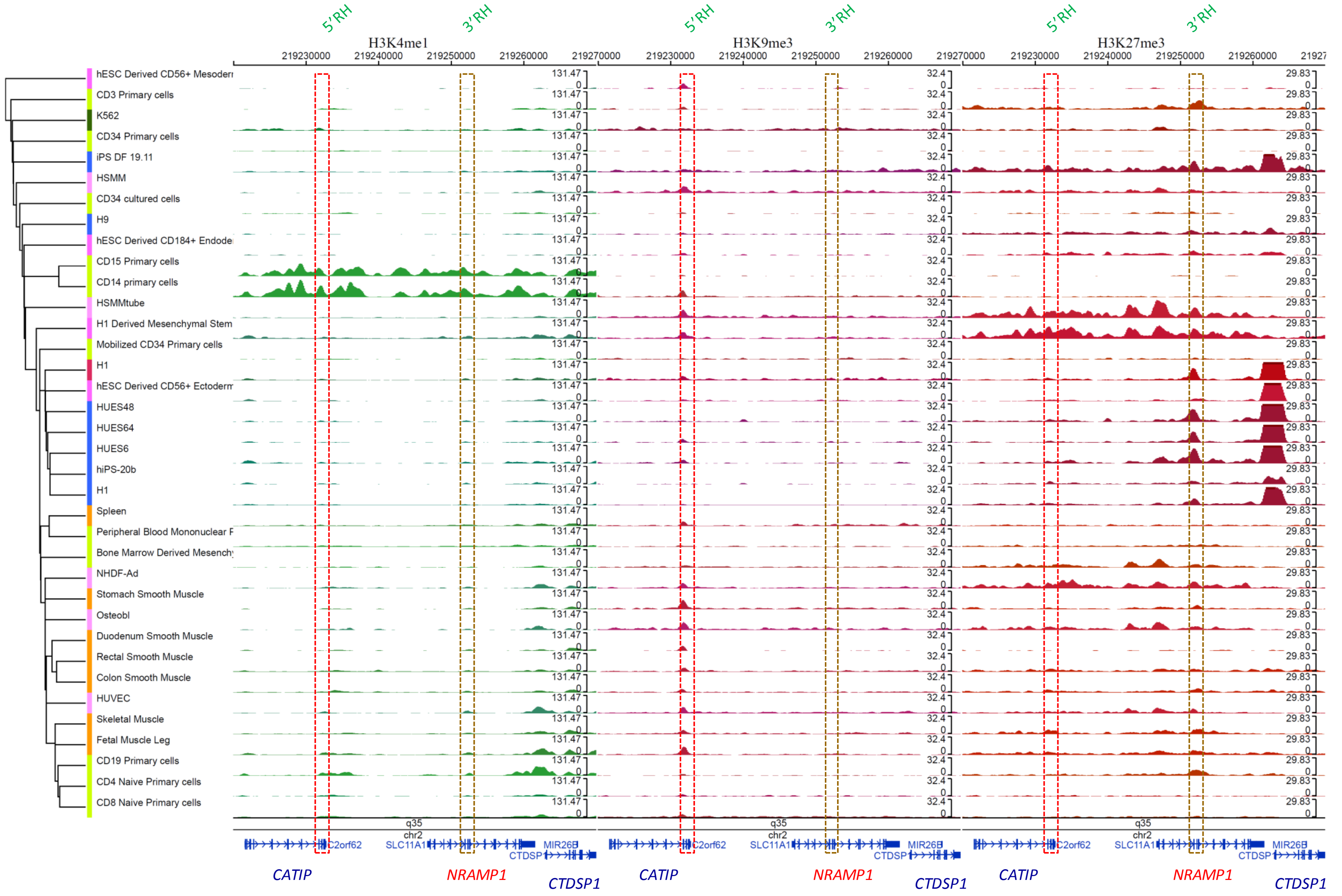

Figure S14. ENCODE CAGE and RNA-seq data corresponding to *NRAMP1* locus. Results are presented separately for five regions (i-v). *From top to bottom*: Location of DHSs; chromosome 2 scale; CTCF ChIP-seq for CD14$^+$ MNs, K562 cells, HUVEC and ESC; CpG islands; UCSC gene description; DHSs in CD14$^+$ MNs, HL-60, NB4, mCD34/CMP, CMK and K562 cells, HUVEC and ESC; ChIP-seq for histone marks of priming (K27ac, K4me1; CD14$^+$ MNs, K562 cells, HUVECs and ESCs); ChIP-seq for the TFs PU.1 and C/EBPb in MN and MF; ChIP-seq for histone marks of activation (K9ac, K4me2 and K4me3); RNA-seq data (CD14$^+$ MNs, K562 cells, mCD34, HUVEC and ESC); RefSeq gene description; CAGE data (CD14$^+$ MNs, K562 cells, mCD34, HUVEC and ESC).

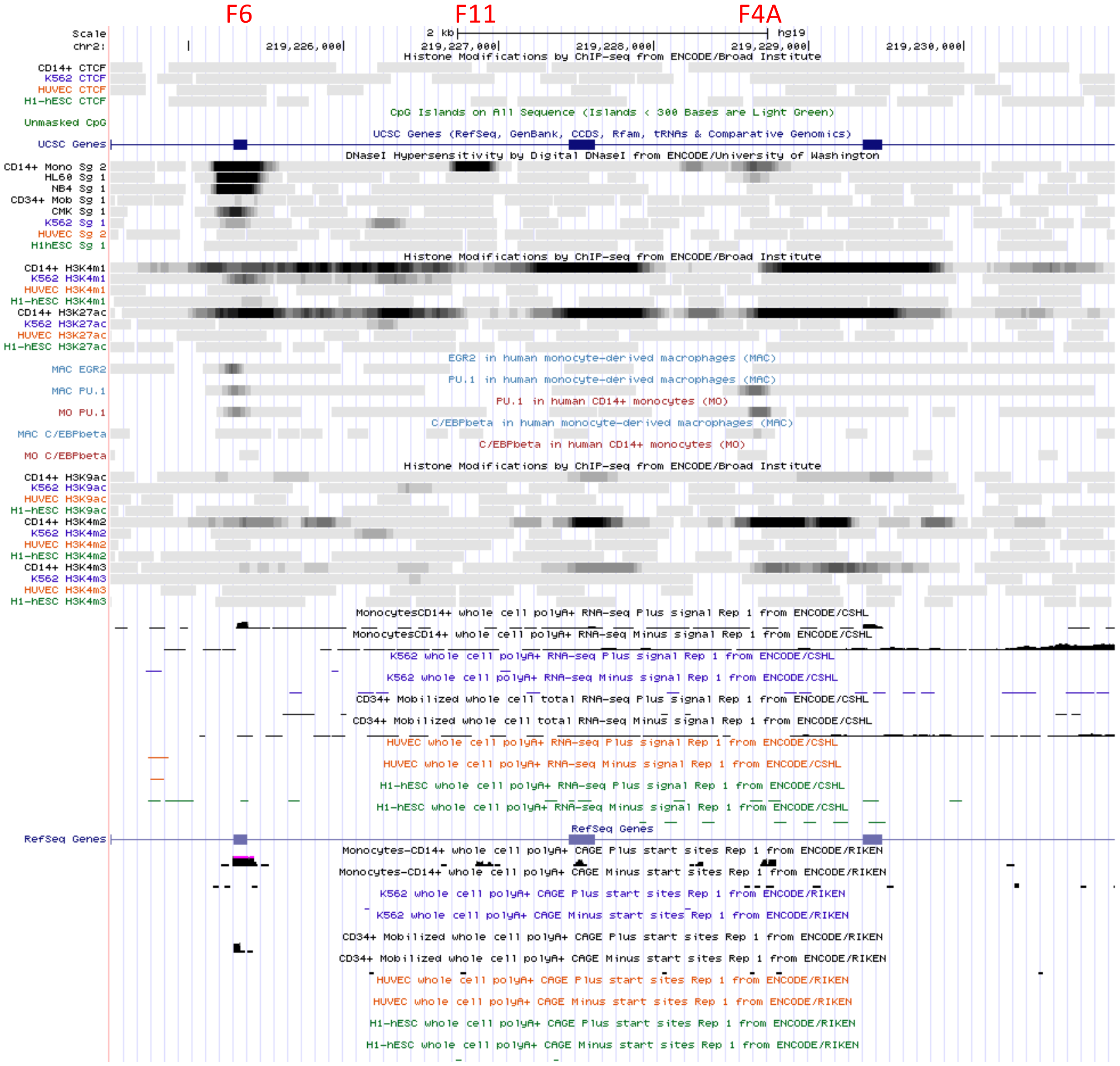
F6
F11
F4A
Scale
chr2:
2 kb
hg19
219,226,000
219,227,000
219,228,000
219,229,000
219,230,000
Histone Modifications by ChIP-seq from ENCODE/Broad Institute
CD14+ CTCF
K562 CTCF
HUVEC CTCF
H1-hESC CTCF
CpG Islands on All Sequence (Islands < 300 Bases are Light Green)
Unmasked CpG
UCSC Genes (RefSeq, GenBank, CCDS, Rfam, tRNAs & Comparative Genomics)
UCSC Genes
DNaseI Hypersensitivity by Digital DNaseI from ENCODE/University of Washington
CD14+ Mono Sg 2
HL60 Sg 1
NB4 Sg 1
CD34+ Mob Sg 1
CMK Sg 1
K562 Sg 1
HUVEC Sg 2
H1hESC Sg 1
Histone Modifications by ChIP-seq from ENCODE/Broad Institute
CD14+ H3K4m1
K562 H3K4m1
HUVEC H3K4m1
H1-hESC H3K4m1
CD14+ H3K27ac
K562 H3K27ac
HUVEC H3K27ac
H1-hESC H3K27ac
EGR2 in human monocyte-derived macrophages (MAC)
MAC EGR2
PU.1 in human monocyte-derived macrophages (MAC)
MAC PU.1
PU.1 in human CD14+ monocytes (MO)
MO PU.1
C/EBPbeta in human monocyte-derived macrophages (MAC)
MAC C/EBPbeta
C/EBPbeta in human CD14+ monocytes (MO)
MO C/EBPbeta
Histone Modifications by ChIP-seq from ENCODE/Broad Institute
CD14+ H3K9ac
K562 H3K9ac
HUVEC H3K9ac
H1-hESC H3K9ac
CD14+ H3K4m2
K562 H3K4m2
HUVEC H3K4m2
H1-hESC H3K4m2
CD14+ H3K4m3
K562 H3K4m3
HUVEC H3K4m3
H1-hESC H3K4m3
MonocytesCD14+ whole cell polyA+ RNA-seq Plus signal Rep 1 from ENCODE/CSHL
MonocytesCD14+ whole cell polyA+ RNA-seq Minus signal Rep 1 from ENCODE/CSHL
K562 whole cell polyA+ RNA-seq Plus signal Rep 1 from ENCODE/CSHL
K562 whole cell polyA+ RNA-seq Minus signal Rep 1 from ENCODE/CSHL
CD34+ Mobilized whole cell total RNA-seq Plus signal Rep 1 from ENCODE/CSHL
CD34+ Mobilized whole cell total RNA-seq Minus signal Rep 1 from ENCODE/CSHL
HUVEC whole cell polyA+ RNA-seq Plus signal Rep 1 from ENCODE/CSHL
HUVEC whole cell polyA+ RNA-seq Minus signal Rep 1 from ENCODE/CSHL
H1-hESC whole cell polyA+ RNA-seq Plus signal Rep 1 from ENCODE/CSHL
H1-hESC whole cell polyA+ RNA-seq Minus signal Rep 1 from ENCODE/CSHL
RefSeq Genes
RefSeq Genes
Monocytes-CD14+ whole cell polyA+ CAGE Plus start sites Rep 1 from ENCODE/RIKEN
Monocytes-CD14+ whole cell polyA+ CAGE Minus start sites Rep 1 from ENCODE/RIKEN
K562 whole cell polyA+ CAGE Plus start sites Rep 1 from ENCODE/RIKEN
K562 whole cell polyA+ CAGE Minus start sites Rep 1 from ENCODE/RIKEN
CD34+ Mobilized whole cell polyA+ CAGE Plus start sites Rep 1 from ENCODE/RIKEN
CD34+ Mobilized whole cell polyA+ CAGE Minus start sites Rep 1 from ENCODE/RIKEN
HUVEC whole cell polyA+ CAGE Plus start sites Rep 1 from ENCODE/RIKEN
HUVEC whole cell polyA+ CAGE Minus start sites Rep 1 from ENCODE/RIKEN
H1-hESC whole cell polyA+ CAGE Plus start sites Rep 1 from ENCODE/RIKEN
H1-hESC whole cell polyA+ CAGE Minus start sites Rep 1 from ENCODE/RIKEN

Region i

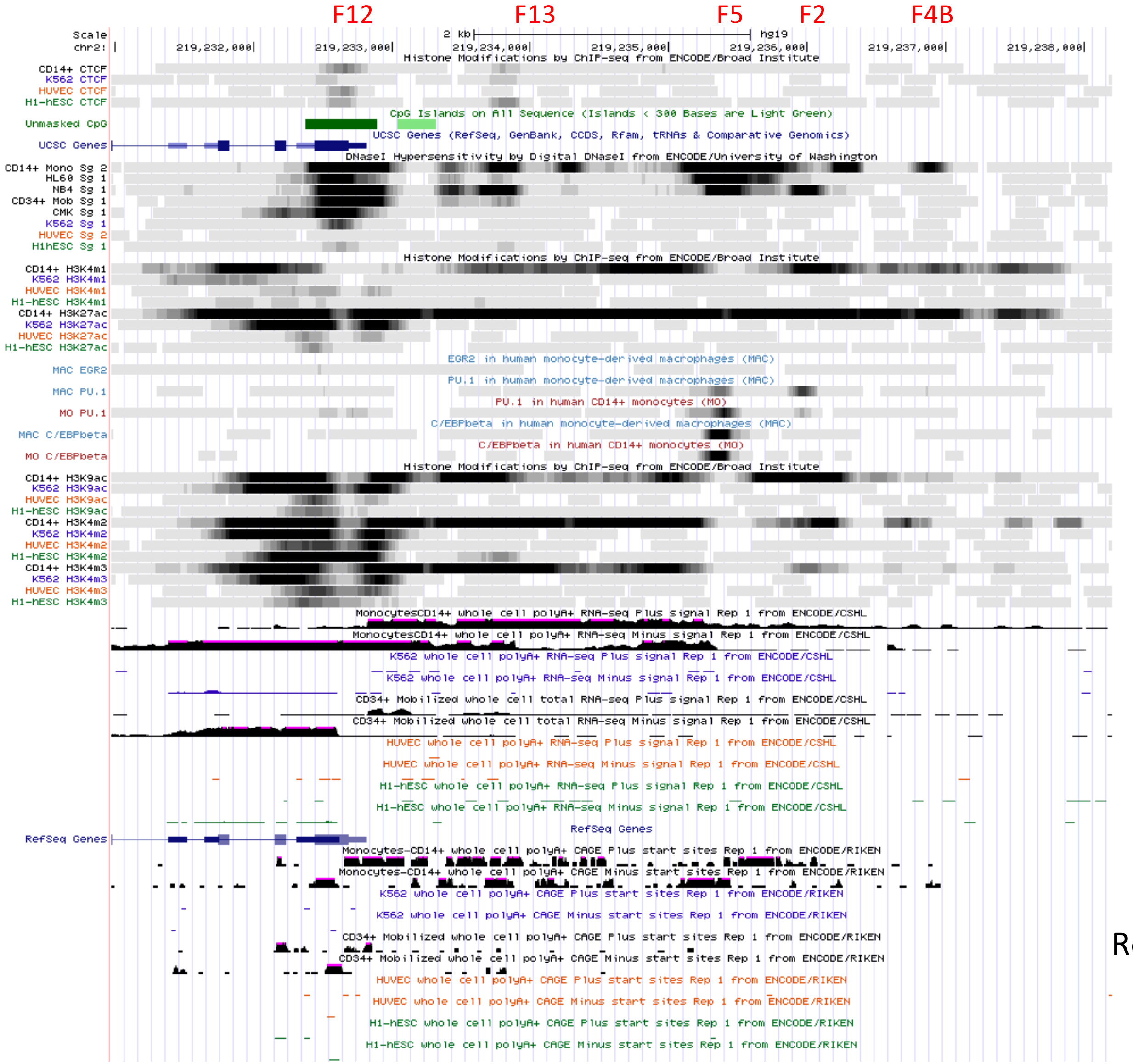
F12
F13
F5
F2
F4B
Scale
chr2:
2 kb
hg19
219,232,000|
219,233,000|
219,234,000|
219,235,000|
219,236,000|
219,237,000|
219,238,000|
Histone Modifications by ChIP-seq from ENCODE/Broad Institute
CD14+ CTCF
K562 CTCF
HUVEC CTCF
H1-hESC CTCF
CpG Islands on All Sequence (Islands < 300 Bases are Light Green)
Unmasked CpG
UCSC Genes (RefSeq, GenBank, CCDS, Rfam, tRNAs & Comparative Genomics)
UCSC Genes
DNaseI Hypersensitivity by Digital DNaseI from ENCODE/University of Washington
CD14+ Mono Sg 2
HL60 Sg 1
NB4 Sg 1
CD34+ Mob Sg 1
CMK Sg 1
K562 Sg 1
HUVEC Sg 2
H1hESC Sg 1
Histone Modifications by ChIP-seq from ENCODE/Broad Institute
CD14+ H3K4m1
K562 H3K4m1
HUVEC H3K4m1
H1-hESC H3K4m1
CD14+ H3K27ac
K562 H3K27ac
HUVEC H3K27ac
H1-hESC H3K27ac
EGR2 in human monocyte-derived macrophages (MAC)
MAC EGR2
PU.1 in human monocyte-derived macrophages (MAC)
MAC PU.1
PU.1 in human CD14+ monocytes (MO)
MO PU.1
C/EBPbeta in human monocyte-derived macrophages (MAC)
MAC C/EBPbeta
C/EBPbeta in human CD14+ monocytes (MO)
MO C/EBPbeta
Histone Modifications by ChIP-seq from ENCODE/Broad Institute
CD14+ H3K9ac
K562 H3K9ac
HUVEC H3K9ac
H1-hESC H3K9ac
CD14+ H3K4m2
K562 H3K4m2
HUVEC H3K4m2
H1-hESC H3K4m2
CD14+ H3K4m3
K562 H3K4m3
HUVEC H3K4m3
H1-hESC H3K4m3
MonocytesCD14+ whole cell polyA+ RNA-seq Plus signal Rep 1 from ENCODE/CSHL
MonocytesCD14+ whole cell polyA+ RNA-seq Minus signal Rep 1 from ENCODE/CSHL
K562 whole cell polyA+ RNA-seq Plus signal Rep 1 from ENCODE/CSHL
K562 whole cell polyA+ RNA-seq Minus signal Rep 1 from ENCODE/CSHL
CD34+ Mobilized whole cell total RNA-seq Plus signal Rep 1 from ENCODE/CSHL
CD34+ Mobilized whole cell total RNA-seq Minus signal Rep 1 from ENCODE/CSHL
HUVEC whole cell polyA+ RNA-seq Plus signal Rep 1 from ENCODE/CSHL
HUVEC whole cell polyA+ RNA-seq Minus signal Rep 1 from ENCODE/CSHL
H1-hESC whole cell polyA+ RNA-seq Plus signal Rep 1 from ENCODE/CSHL
H1-hESC whole cell polyA+ RNA-seq Minus signal Rep 1 from ENCODE/CSHL
RefSeq Genes
RefSeq Genes
Monocytes-CD14+ whole cell polyA+ CAGE Plus start sites Rep 1 from ENCODE/RIKEN
Monocytes-CD14+ whole cell polyA+ CAGE Minus start sites Rep 1 from ENCODE/RIKEN
K562 whole cell polyA+ CAGE Plus start sites Rep 1 from ENCODE/RIKEN
K562 whole cell polyA+ CAGE Minus start sites Rep 1 from ENCODE/RIKEN
CD34+ Mobilized whole cell polyA+ CAGE Plus start sites Rep 1 from ENCODE/RIKEN
CD34+ Mobilized whole cell polyA+ CAGE Minus start sites Rep 1 from ENCODE/RIKEN
HUVEC whole cell polyA+ CAGE Plus start sites Rep 1 from ENCODE/RIKEN
HUVEC whole cell polyA+ CAGE Minus start sites Rep 1 from ENCODE/RIKEN
H1-hESC whole cell polyA+ CAGE Plus start sites Rep 1 from ENCODE/RIKEN
H1-hESC whole cell polyA+ CAGE Minus start sites Rep 1 from ENCODE/RIKEN

Region ii

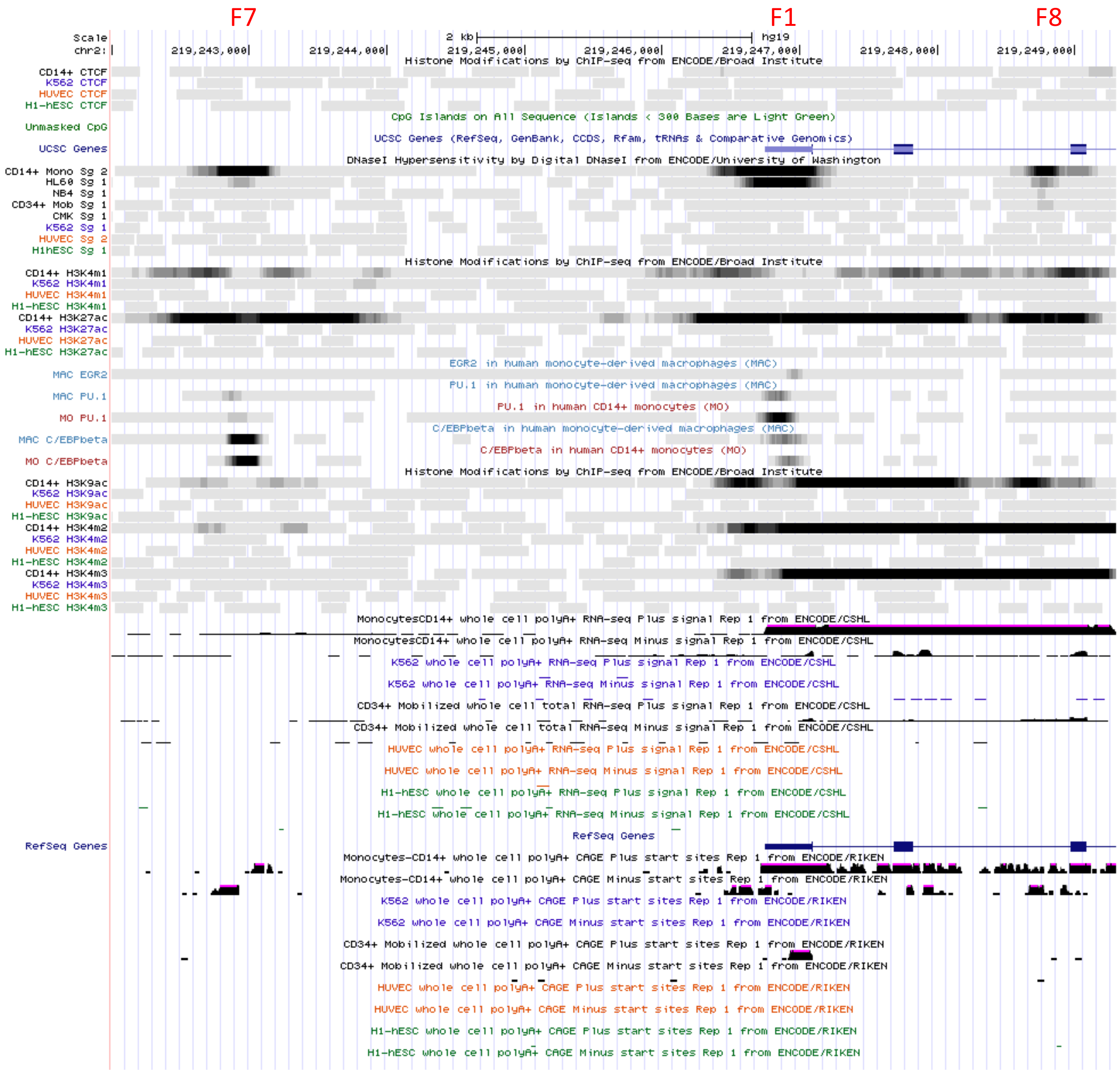
F7
F1
F8
Scale
chr2:
2 kb
hg19
219,243,000
219,244,000
219,245,000
219,246,000
219,247,000
219,248,000
219,249,000
Histone Modifications by ChIP-seq from ENCODE/Broad Institute
CD14+ CTCF
K562 CTCF
HUVEC CTCF
H1-hESC CTCF
CpG Islands on All Sequence (Islands < 300 Bases are Light Green)
Unmasked CpG
UCSC Genes (RefSeq, GenBank, CCDS, Rfam, tRNAs & Comparative Genomics)
UCSC Genes
DNaseI Hypersensitivity by Digital DNaseI from ENCODE/University of Washington
CD14+ Mono Sg 2
HL60 Sg 1
NB4 Sg 1
CD34+ Mob Sg 1
CMK Sg 1
K562 Sg 1
HUVEC Sg 2
H1hESC Sg 1
Histone Modifications by ChIP-seq from ENCODE/Broad Institute
CD14+ H3K4m1
K562 H3K4m1
HUVEC H3K4m1
H1-hESC H3K4m1
CD14+ H3K27ac
K562 H3K27ac
HUVEC H3K27ac
H1-hESC H3K27ac
EGR2 in human monocyte-derived macrophages (MAC)
MAC EGR2
PU.1 in human monocyte-derived macrophages (MAC)
MAC PU.1
PU.1 in human CD14+ monocytes (MO)
MO PU.1
C/EBPbeta in human monocyte-derived macrophages (MAC)
MAC C/EBPbeta
C/EBPbeta in human CD14+ monocytes (MO)
MO C/EBPbeta
Histone Modifications by ChIP-seq from ENCODE/Broad Institute
CD14+ H3K9ac
K562 H3K9ac
HUVEC H3K9ac
H1-hESC H3K9ac
CD14+ H3K4m2
K562 H3K4m2
HUVEC H3K4m2
H1-hESC H3K4m2
CD14+ H3K4m3
K562 H3K4m3
HUVEC H3K4m3
H1-hESC H3K4m3
MonocytesCD14+ whole cell polyA+ RNA-seq Plus signal Rep 1 from ENCODE/CSHL
MonocytesCD14+ whole cell polyA+ RNA-seq Minus signal Rep 1 from ENCODE/CSHL
K562 whole cell polyA+ RNA-seq Plus signal Rep 1 from ENCODE/CSHL
K562 whole cell polyA+ RNA-seq Minus signal Rep 1 from ENCODE/CSHL
CD34+ Mobilized whole cell total RNA-seq Plus signal Rep 1 from ENCODE/CSHL
CD34+ Mobilized whole cell total RNA-seq Minus signal Rep 1 from ENCODE/CSHL
HUVEC whole cell polyA+ RNA-seq Plus signal Rep 1 from ENCODE/CSHL
HUVEC whole cell polyA+ RNA-seq Minus signal Rep 1 from ENCODE/CSHL
H1-hESC whole cell polyA+ RNA-seq Plus signal Rep 1 from ENCODE/CSHL
H1-hESC whole cell polyA+ RNA-seq Minus signal Rep 1 from ENCODE/CSHL
RefSeq Genes
RefSeq Genes
Monocytes-CD14+ whole cell polyA+ CAGE Plus start sites Rep 1 from ENCODE/RIKEN
Monocytes-CD14+ whole cell polyA+ CAGE Minus start sites Rep 1 from ENCODE/RIKEN
K562 whole cell polyA+ CAGE Plus start sites Rep 1 from ENCODE/RIKEN
K562 whole cell polyA+ CAGE Minus start sites Rep 1 from ENCODE/RIKEN
CD34+ Mobilized whole cell polyA+ CAGE Plus start sites Rep 1 from ENCODE/RIKEN
CD34+ Mobilized whole cell polyA+ CAGE Minus start sites Rep 1 from ENCODE/RIKEN
HUVEC whole cell polyA+ CAGE Plus start sites Rep 1 from ENCODE/RIKEN
HUVEC whole cell polyA+ CAGE Minus start sites Rep 1 from ENCODE/RIKEN
H1-hESC whole cell polyA+ CAGE Plus start sites Rep 1 from ENCODE/RIKEN
H1-hESC whole cell polyA+ CAGE Minus start sites Rep 1 from ENCODE/RIKEN

Region iii

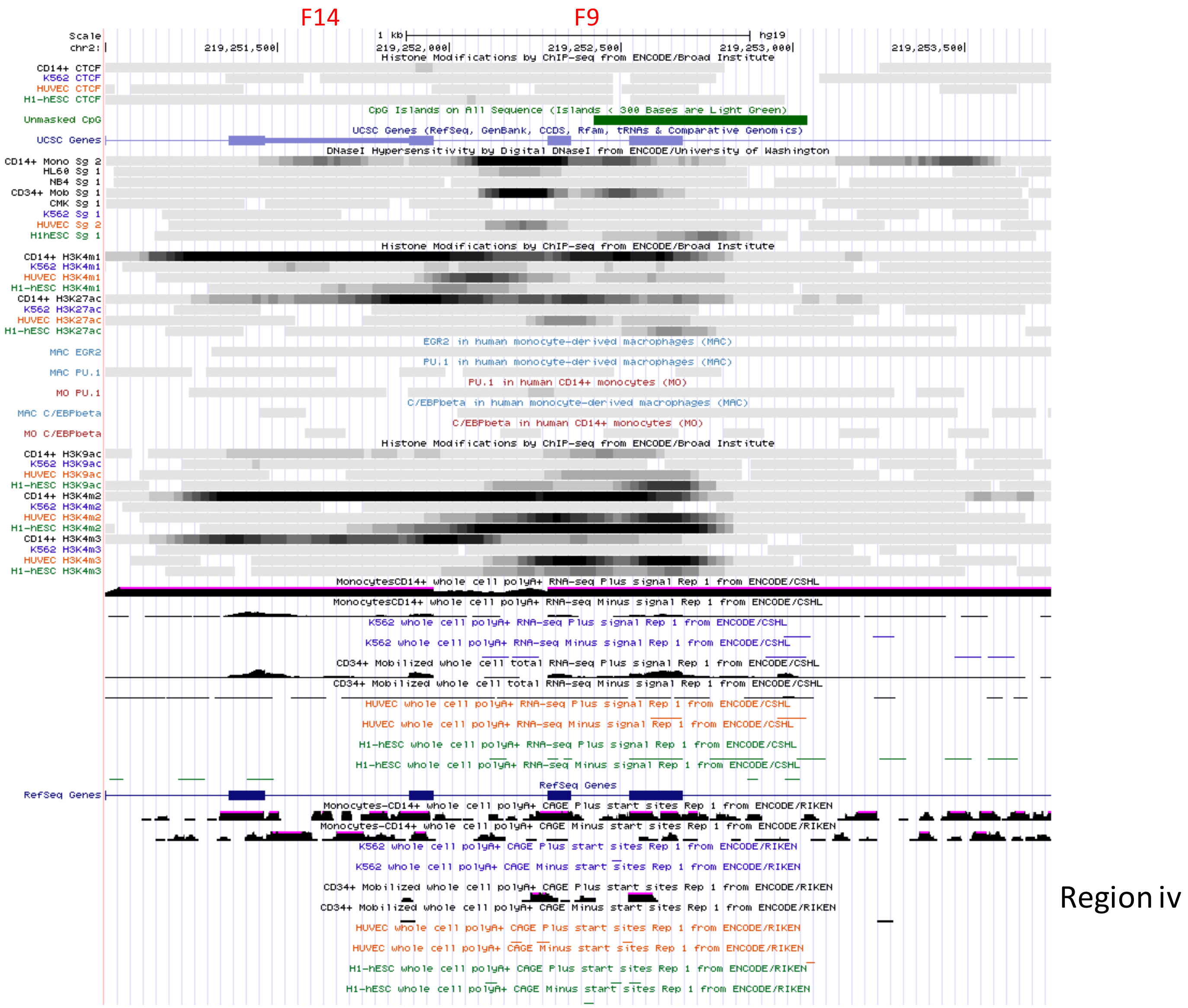
F14
F9
Scale
chr2:
1 kb
hg19
219,251,500
219,252,000
219,252,500
219,253,000
219,253,500
Histone Modifications by ChIP-seq from ENCODE/Broad Institute
CD14+ CTCF
K562 CTCF
HUVEC CTCF
H1-hESC CTCF
CpG Islands on All Sequence (Islands < 300 Bases are Light Green)
Unmasked CpG
UCSC Genes (RefSeq, GenBank, CCDS, Rfam, tRNAs & Comparative Genomics)
UCSC Genes
DNaseI Hypersensitivity by Digital DNaseI from ENCODE/University of Washington
CD14+ Mono Sg 2
HL60 Sg 1
NB4 Sg 1
CD34+ Mob Sg 1
CMK Sg 1
K562 Sg 1
HUVEC Sg 2
H1hESC Sg 1
CD14+ H3K4m1
K562 H3K4m1
HUVEC H3K4m1
H1-hESC H3K4m1
CD14+ H3K27ac
K562 H3K27ac
HUVEC H3K27ac
H1-hESC H3K27ac
EGR2 in human monocyte-derived macrophages (MAC)
MAC EGR2
PU.1 in human monocyte-derived macrophages (MAC)
MAC PU.1
PU.1 in human CD14+ monocytes (MO)
MO PU.1
C/EBPbeta in human monocyte-derived macrophages (MAC)
MAC C/EBPbeta
C/EBPbeta in human CD14+ monocytes (MO)
MO C/EBPbeta
CD14+ H3K9ac
K562 H3K9ac
HUVEC H3K9ac
H1-hESC H3K9ac
CD14+ H3K4m2
K562 H3K4m2
HUVEC H3K4m2
H1-hESC H3K4m2
CD14+ H3K4m3
K562 H3K4m3
HUVEC H3K4m3
H1-hESC H3K4m3
MonocytesCD14+ whole cell polyA+ RNA-seq Plus signal Rep 1 from ENCODE/CSHL
MonocytesCD14+ whole cell polyA+ RNA-seq Minus signal Rep 1 from ENCODE/CSHL
K562 whole cell polyA+ RNA-seq Plus signal Rep 1 from ENCODE/CSHL
K562 whole cell polyA+ RNA-seq Minus signal Rep 1 from ENCODE/CSHL
CD34+ Mobilized whole cell total RNA-seq Plus signal Rep 1 from ENCODE/CSHL
CD34+ Mobilized whole cell total RNA-seq Minus signal Rep 1 from ENCODE/CSHL
HUVEC whole cell polyA+ RNA-seq Plus signal Rep 1 from ENCODE/CSHL
HUVEC whole cell polyA+ RNA-seq Minus signal Rep 1 from ENCODE/CSHL
H1-hESC whole cell polyA+ RNA-seq Plus signal Rep 1 from ENCODE/CSHL
H1-hESC whole cell polyA+ RNA-seq Minus signal Rep 1 from ENCODE/CSHL
RefSeq Genes
RefSeq Genes
Monocytes-CD14+ whole cell polyA+ CAGE Plus start sites Rep 1 from ENCODE/RIKEN
Monocytes-CD14+ whole cell polyA+ CAGE Minus start sites Rep 1 from ENCODE/RIKEN
K562 whole cell polyA+ CAGE Plus start sites Rep 1 from ENCODE/RIKEN
K562 whole cell polyA+ CAGE Minus start sites Rep 1 from ENCODE/RIKEN
CD34+ Mobilized whole cell polyA+ CAGE Plus start sites Rep 1 from ENCODE/RIKEN
CD34+ Mobilized whole cell polyA+ CAGE Minus start sites Rep 1 from ENCODE/RIKEN
HUVEC whole cell polyA+ CAGE Plus start sites Rep 1 from ENCODE/RIKEN
HUVEC whole cell polyA+ CAGE Minus start sites Rep 1 from ENCODE/RIKEN
H1-hESC whole cell polyA+ CAGE Plus start sites Rep 1 from ENCODE/RIKEN
H1-hESC whole cell polyA+ CAGE Minus start sites Rep 1 from ENCODE/RIKEN
Region iv

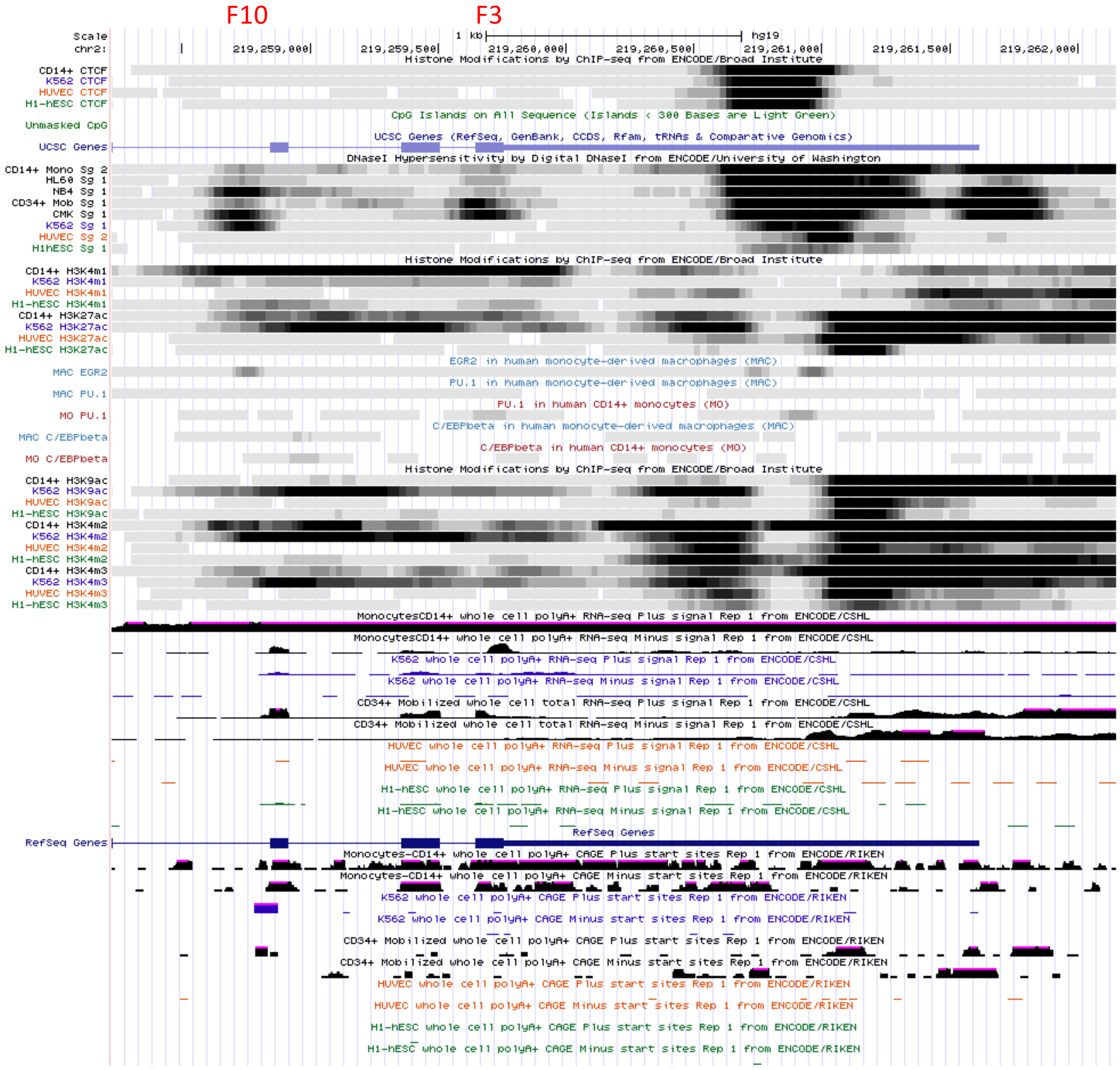
F10
F3
Scale
chr2:
1 kb
hg19
219,259,000
219,259,500
219,260,000
219,260,500
219,261,000
219,261,500
219,262,000
Histone Modifications by ChIP-seq from ENCODE/Broad Institute
CD14+ CTCF
K562 CTCF
HUVEC CTCF
H1-hESC CTCF
CpG Islands on All Sequence (Islands < 300 Bases are Light Green)
Unmasked CpG
UCSC Genes (RefSeq, GenBank, CCDS, Rfam, tRNAs & Comparative Genomics)
UCSC Genes
DNaseI Hypersensitivity by Digital DNaseI from ENCODE/University of Washington
CD14+ Mono Sg 2
HL60 Sg 1
NB4 Sg 1
CD34+ Mob Sg 1
CMK Sg 1
K562 Sg 1
HUVEC Sg 2
H1hESC Sg 1
Histone Modifications by ChIP-seq from ENCODE/Broad Institute
CD14+ H3K4m1
K562 H3K4m1
HUVEC H3K4m1
H1-hESC H3K4m1
CD14+ H3K27ac
K562 H3K27ac
HUVEC H3K27ac
H1-hESC H3K27ac
EGR2 in human monocyte-derived macrophages (MAC)
MAC EGR2
PU.1 in human monocyte-derived macrophages (MAC)
MAC PU.1
PU.1 in human CD14+ monocytes (MO)
MO PU.1
C/EBPbeta in human monocyte-derived macrophages (MAC)
MAC C/EBPbeta
C/EBPbeta in human CD14+ monocytes (MO)
MO C/EBPbeta
Histone Modifications by ChIP-seq from ENCODE/Broad Institute
CD14+ H3K9ac
K562 H3K9ac
HUVEC H3K9ac
H1-hESC H3K9ac
CD14+ H3K4m2
K562 H3K4m2
HUVEC H3K4m2
H1-hESC H3K4m2
CD14+ H3K4m3
K562 H3K4m3
HUVEC H3K4m3
H1-hESC H3K4m3
MonocytesCD14+ whole cell polyA+ RNA-seq Plus signal Rep 1 from ENCODE/CSHL
MonocytesCD14+ whole cell polyA+ RNA-seq Minus signal Rep 1 from ENCODE/CSHL
K562 whole cell polyA+ RNA-seq Plus signal Rep 1 from ENCODE/CSHL
K562 whole cell polyA+ RNA-seq Minus signal Rep 1 from ENCODE/CSHL
CD34+ Mobilized whole cell total RNA-seq Plus signal Rep 1 from ENCODE/CSHL
CD34+ Mobilized whole cell total RNA-seq Minus signal Rep 1 from ENCODE/CSHL
HUVEC whole cell polyA+ RNA-seq Plus signal Rep 1 from ENCODE/CSHL
HUVEC whole cell polyA+ RNA-seq Minus signal Rep 1 from ENCODE/CSHL
H1-hESC whole cell polyA+ RNA-seq Plus signal Rep 1 from ENCODE/CSHL
H1-hESC whole cell polyA+ RNA-seq Minus signal Rep 1 from ENCODE/CSHL
RefSeq Genes
RefSeq Genes
Monocytes-CD14+ whole cell polyA+ CAGE Plus start sites Rep 1 from ENCODE/RIKEN
Monocytes-CD14+ whole cell polyA+ CAGE Minus start sites Rep 1 from ENCODE/RIKEN
K562 whole cell polyA+ CAGE Plus start sites Rep 1 from ENCODE/RIKEN
K562 whole cell polyA+ CAGE Minus start sites Rep 1 from ENCODE/RIKEN
CD34+ Mobilized whole cell polyA+ CAGE Plus start sites Rep 1 from ENCODE/RIKEN
CD34+ Mobilized whole cell polyA+ CAGE Minus start sites Rep 1 from ENCODE/RIKEN
HUVEC whole cell polyA+ CAGE Plus start sites Rep 1 from ENCODE/RIKEN
HUVEC whole cell polyA+ CAGE Minus start sites Rep 1 from ENCODE/RIKEN
H1-hESC whole cell polyA+ CAGE Plus start sites Rep 1 from ENCODE/RIKEN
H1-hESC whole cell polyA+ CAGE Minus start sites Rep 1 from ENCODE/RIKEN

Region v

Figure S15. Chromatin status at *NRAMP1* locus in hematopoietic cells. *From top to bottom*: position and orientation of CTCF sites indicated by arrows, red (forward), blue (reverse) and grey (undetermined); chromosome 2 regions carrying predicted transcriptional regulatory elements (F1-F14); chromosome 2 scale bar; ENCODE CTCF-specific ChIP-seq ($CD14^+$ MNs, K562 cells); UCSC gene descriptions; CpG Islands; ENCODE TF-specific ChIP-seq data; ENCODE DHS for $CD14^+$ MNs (x2), HL-60, NB4, mCD34, CMK and K562 cells; ENCODE ChIP-seq data for select histone marks (K4me1 and K27ac) in $CD14^+$ MNs and K562 cells; ChIP-seq data for the TFs EGR2, PU.1 and C/EBPb in MO and MAC [13]; ENCODE ChIP-seq data for histone marks K9ac, K4me2 and K4me3 in $CD14^+$ MNs and K562 cells; RefSeq gene descriptions; NIH Roadmap epigenomic data for hematopoietic cells: DHS and ChIP-seq data for histone marks of priming, K4me1 and K27ac (panel **A**); ChIP-seq data for histone marks of transcriptional activity, K9ac, K4me3 and K36me3 (panel **B**); RNA-seq profile and ChIP-seq data for histone marks of repression, K9me3 and K27me3 (panel **C**)

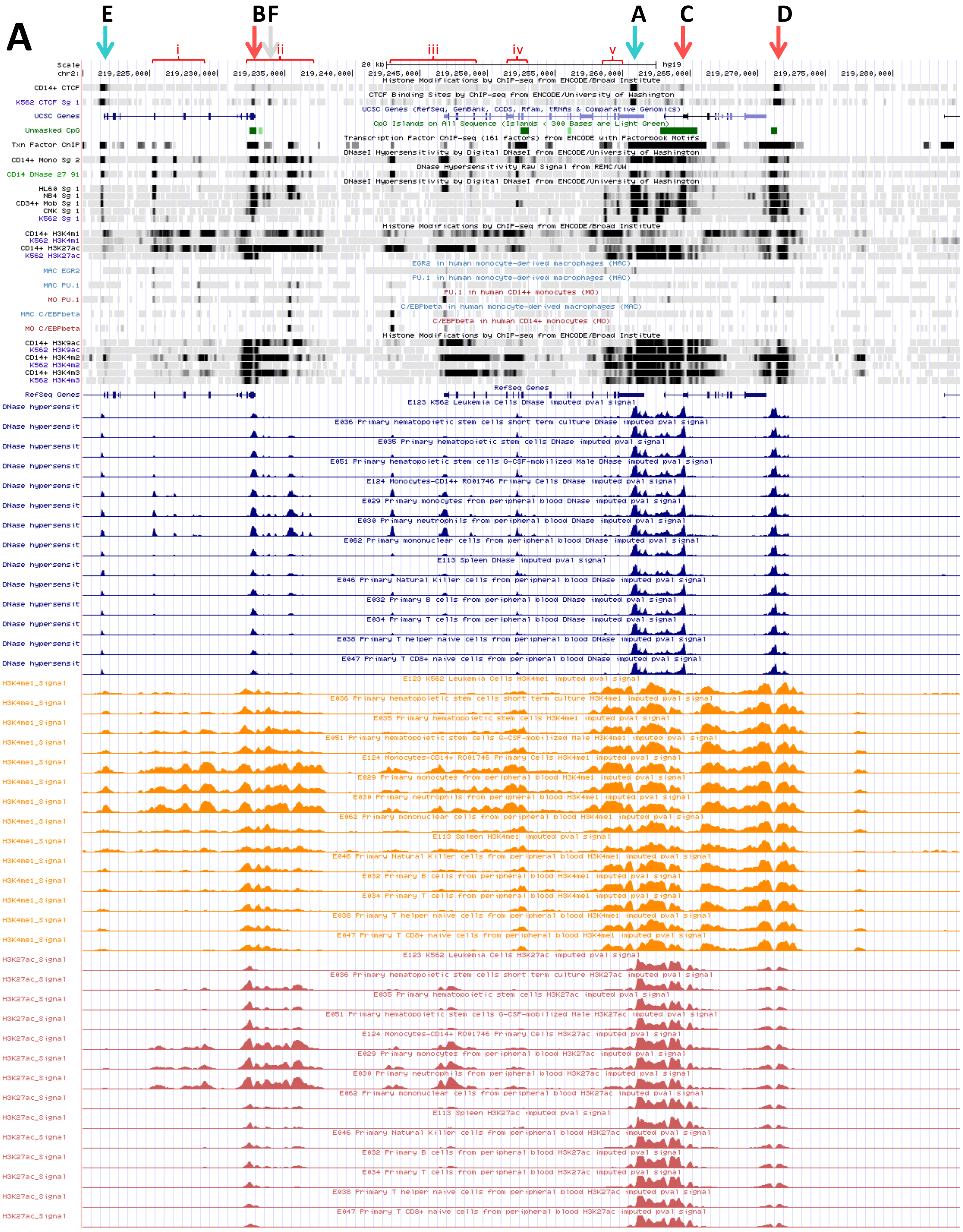
A
E
BF
A
C
D
i
ii
iii
iv
v

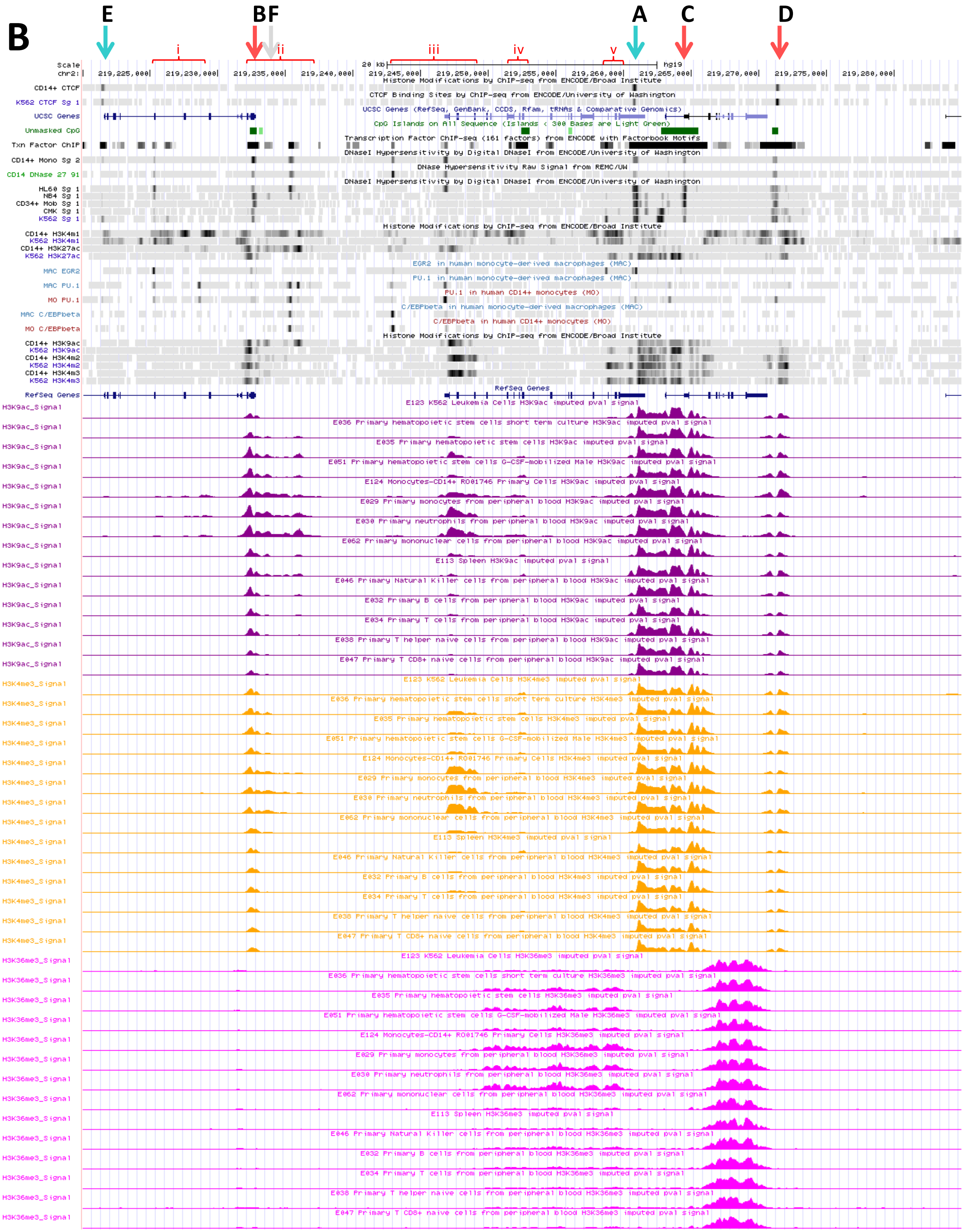
B
E
BF
A
C
D
i
ii
iii
iv
v
Scale
chr2:
20 kb
hg19
CD14+ CTCF
K562 CTCF Sg 1
UCSC Genes
Unmasked CpG
Txn Factor ChIP
CD14+ Mono Sg 2
CD14 DNase 27 91
HL60 Sg 1
NB4 Sg 1
CD34+ Mob Sg 1
CMK Sg 1
K562 Sg 1
CD14+ H3K4m1
K562 H3K4m1
CD14+ H3K27ac
K562 H3K27ac
MAC EGR2
MAC PU.1
MO PU.1
MAC C/EBPbeta
MO C/EBPbeta
CD14+ H3K9ac
K562 H3K9ac
CD14+ H3K4m2
K562 H3K4m2
CD14+ H3K4m3
K562 H3K4m3
RefSeq Genes
H3K9ac_Signal
H3K4me3_Signal
H3K36me3_Signal

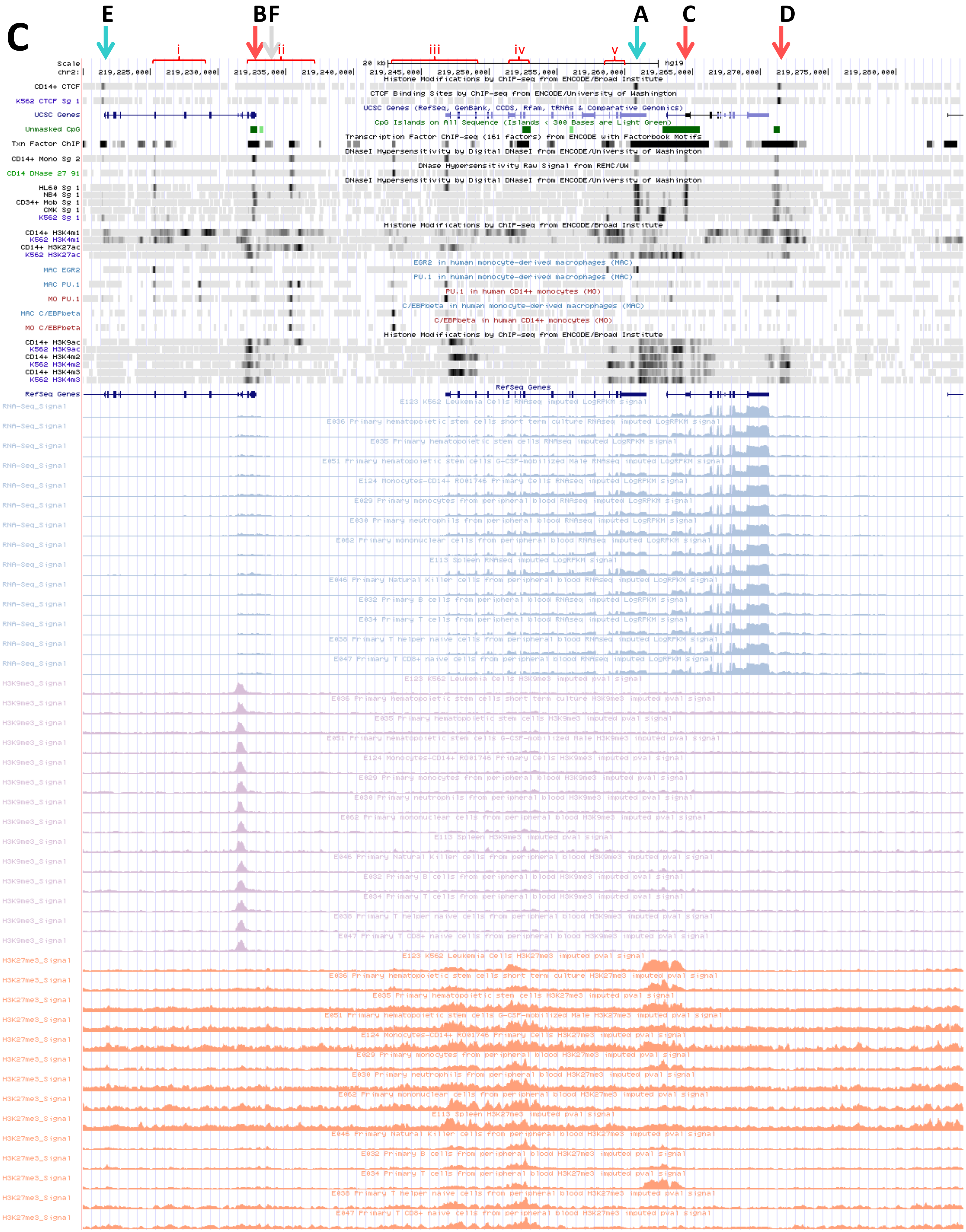
C
E
BF
A
C
D
i
ii
iii
iv
v
Scale
chr2:
20 kb
hg19
219,225,000
219,230,000
219,235,000
219,240,000
219,245,000
219,250,000
219,255,000
219,260,000
219,265,000
219,270,000
219,275,000
219,280,000
Histone Modifications by ChIP-seq from ENCODE/Broad Institute
CD14+ CTCF
CTCF Binding Sites by ChIP-seq from ENCODE/University of Washington
K562 CTCF Sg 1
UCSC Genes (RefSeq, GenBank, CCDS, Rfam, tRNAs & Comparative Genomics)
UCSC Genes
CpG Islands on All Sequence (Islands < 300 Bases are Light Green)
Unmasked CpG
Transcription Factor ChIP-seq (161 factors) from ENCODE with Factorbook Motifs
Txn Factor ChIP
DNaseI Hypersensitivity by Digital DNaseI from ENCODE/University of Washington
CD14+ Mono Sg 2
DNase Hypersensitivity Raw Signal from REMC/UW
CD14 DNase 27 91
DNaseI Hypersensitivity by Digital DNaseI from ENCODE/University of Washington
HL60 Sg 1
NB4 Sg 1
CD34+ Mob Sg 1
CMK Sg 1
K562 Sg 1
Histone Modifications by ChIP-seq from ENCODE/Broad Institute
CD14+ H3K4m1
K562 H3K4m1
CD14+ H3K27ac
K562 H3K27ac
EGR2 in human monocyte-derived macrophages (MAC)
MAC EGR2
PU.1 in human monocyte-derived macrophages (MAC)
MAC PU.1
PU.1 in human CD14+ monocytes (MO)
MO PU.1
C/EBPbeta in human monocyte-derived macrophages (MAC)
MAC C/EBPbeta
C/EBPbeta in human CD14+ monocytes (MO)
MO C/EBPbeta
Histone Modifications by ChIP-seq from ENCODE/Broad Institute
CD14+ H3K9ac
K562 H3K9ac
CD14+ H3K4m2
K562 H3K4m2
CD14+ H3K4m3
K562 H3K4m3
RefSeq Genes
RefSeq Genes
RNA-Seq_Signal
E123 K562 Leukemia Cells RNAseq imputed LogRPKM signal
E036 Primary hematopoietic stem cells short term culture RNAseq imputed LogRPKM signal
E035 Primary hematopoietic stem cells RNAseq imputed LogRPKM signal
E051 Primary hematopoietic stem cells G-CSF-mobilized Male RNAseq imputed LogRPKM signal
E124 Monocytes-CD14+ RO01746 Primary Cells RNAseq imputed LogRPKM signal
E029 Primary monocytes from peripheral blood RNAseq imputed LogRPKM signal
E030 Primary neutrophils from peripheral blood RNAseq imputed LogRPKM signal
E062 Primary mononuclear cells from peripheral blood RNAseq imputed LogRPKM signal
E113 Spleen RNAseq imputed LogRPKM signal
E046 Primary Natural Killer cells from peripheral blood RNAseq imputed LogRPKM signal
E032 Primary B cells from peripheral blood RNAseq imputed LogRPKM signal
E034 Primary T cells from peripheral blood RNAseq imputed LogRPKM signal
E038 Primary T helper naive cells from peripheral blood RNAseq imputed LogRPKM signal
E047 Primary T CD8+ naive cells from peripheral blood RNAseq imputed LogRPKM signal
H3K9me3_Signal
E123 K562 Leukemia Cells H3K9me3 imputed pval signal
E036 Primary hematopoietic stem cells short term culture H3K9me3 imputed pval signal
E035 Primary hematopoietic stem cells H3K9me3 imputed pval signal
E051 Primary hematopoietic stem cells G-CSF-mobilized Male H3K9me3 imputed pval signal
E124 Monocytes-CD14+ RO01746 Primary Cells H3K9me3 imputed pval signal
E029 Primary monocytes from peripheral blood H3K9me3 imputed pval signal
E030 Primary neutrophils from peripheral blood H3K9me3 imputed pval signal
E062 Primary mononuclear cells from peripheral blood H3K9me3 imputed pval signal
E113 Spleen H3K9me3 imputed pval signal
E046 Primary Natural Killer cells from peripheral blood H3K9me3 imputed pval signal
E032 Primary B cells from peripheral blood H3K9me3 imputed pval signal
E034 Primary T cells from peripheral blood H3K9me3 imputed pval signal
E038 Primary T helper naive cells from peripheral blood H3K9me3 imputed pval signal
E047 Primary T CD8+ naive cells from peripheral blood H3K9me3 imputed pval signal
H3K27me3_Signal
E123 K562 Leukemia Cells H3K27me3 imputed pval signal
E036 Primary hematopoietic stem cells short term culture H3K27me3 imputed pval signal
E035 Primary hematopoietic stem cells H3K27me3 imputed pval signal
E051 Primary hematopoietic stem cells G-CSF-mobilized Male H3K27me3 imputed pval signal
E124 Monocytes-CD14+ RO01746 Primary Cells H3K27me3 imputed pval signal
E029 Primary monocytes from peripheral blood H3K27me3 imputed pval signal
E030 Primary neutrophils from peripheral blood H3K27me3 imputed pval signal
E062 Primary mononuclear cells from peripheral blood H3K27me3 imputed pval signal
E113 Spleen H3K27me3 imputed pval signal
E046 Primary Natural Killer cells from peripheral blood H3K27me3 imputed pval signal
E032 Primary B cells from peripheral blood H3K27me3 imputed pval signal
E034 Primary T cells from peripheral blood H3K27me3 imputed pval signal
E038 Primary T helper naive cells from peripheral blood H3K27me3 imputed pval signal
E047 Primary T CD8+ naive cells from peripheral blood H3K27me3 imputed pval signal

Figure S16. Chromatin status at *NRAMP1* locus in myeloid cells, HUVEC and ESC. *From top to bottom*: position and orientation of CTCF sites indicated by arrows, red (forward), blue (reverse) and grey (undetermined); chromosome 2 regions carrying predicted transcriptional regulatory elements (F1-F14); chromosome 2 scale bar; ENCODE CTCF-specific ChIP-seq ($CD14^+$ MNs, K562 cells); UCSC gene descriptions; CpG Islands; ENCODE TF-specific ChIP-seq data; ENCODE DHS for $CD14^+$ MNs (x2), HL-60, NB4, mCD34, CMK and K562 cells; ENCODE ChIP-seq data for select histone marks (K4me1 and K27ac) in $CD14^+$ MNs and K562 cells; ChIP-seq data for the TFs EGR2, PU.1 and C/EBPb in MO and MAC [13]; ENCODE ChIP-seq data for histone marks K9ac, K4me2 and K4me3 in $CD14^+$ MNs and K562 cells; RefSeq gene descriptions; NIH Roadmap epigenomic data for mesodermal cell types and ESCs: DHS and ChIP-seq data for histone marks of priming, K4me1 and K27ac (panel **A**); ChIP-seq data for histone marks of transcriptional activity, K9ac, K4me3 and K36me3 (panel **B**); RNA-seq profile and ChIP-seq data for histone marks of repression, K9me3 and K27me3 (panel **C**).

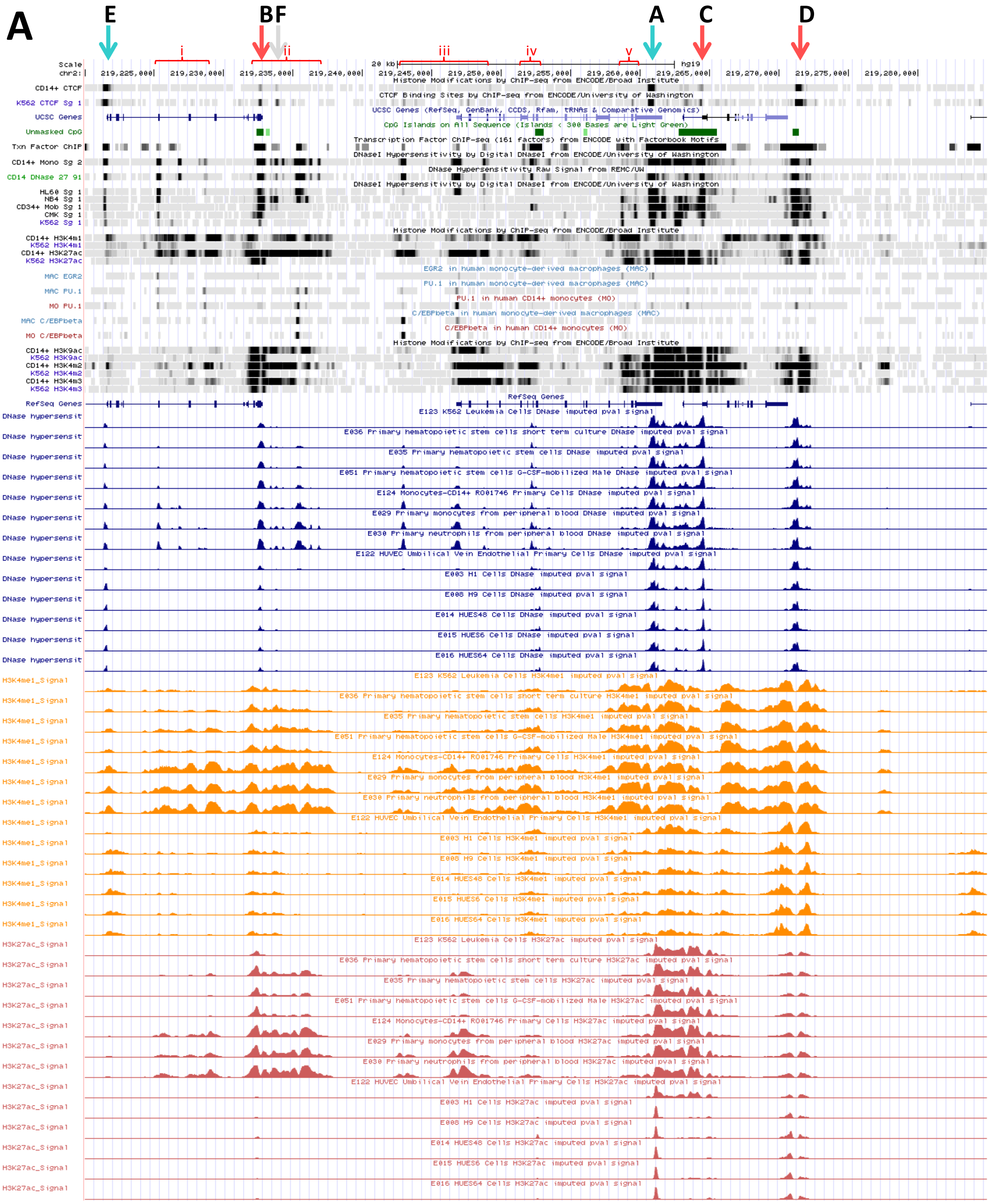
A
E
BF
A
C
D
i
ii
iii
iv
v
Scale
chr2:
20 kb
hg19
219,225,000
219,230,000
219,235,000
219,240,000
219,245,000
219,250,000
219,255,000
219,260,000
219,265,000
219,270,000
219,275,000
219,280,000
Histone Modifications by ChIP-seq from ENCODE/Broad Institute
CD14+ CTCF
CTCF Binding Sites by ChIP-seq from ENCODE/University of Washington
K562 CTCF Sg 1
UCSC Genes (RefSeq, GenBank, CCDS, Rfam, tRNAs & Comparative Genomics)
UCSC Genes
CpG Islands on All Sequence (Islands < 300 Bases are Light Green)
Unmasked CpG
Transcription Factor ChIP-seq (161 factors) from ENCODE with Factorbook Motifs
Txn Factor ChIP
DNaseI Hypersensitivity by Digital DNaseI from ENCODE/University of Washington
CD14+ Mono Sg 2
DNase Hypersensitivity Raw Signal from REMC/UW
CD14 DNase 27 91
DNaseI Hypersensitivity by Digital DNaseI from ENCODE/University of Washington
HL60 Sg 1
NB4 Sg 1
CD34+ Mob Sg 1
CMK Sg 1
K562 Sg 1
Histone Modifications by ChIP-seq from ENCODE/Broad Institute
CD14+ H3K4m1
K562 H3K4m1
CD14+ H3K27ac
K562 H3K27ac
EGR2 in human monocyte-derived macrophages (MAC)
MAC EGR2
PU.1 in human monocyte-derived macrophages (MAC)
MAC PU.1
PU.1 in human CD14+ monocytes (MO)
MO PU.1
C/EBPbeta in human monocyte-derived macrophages (MAC)
MAC C/EBPbeta
C/EBPbeta in human CD14+ monocytes (MO)
MO C/EBPbeta
Histone Modifications by ChIP-seq from ENCODE/Broad Institute
CD14+ H3K9ac
K562 H3K9ac
CD14+ H3K4m2
K562 H3K4m2
CD14+ H3K4m3
K562 H3K4m3
RefSeq Genes
RefSeq Genes
DNase hypersensit
E123 K562 Leukemia Cells DNase imputed pval signal
E036 Primary hematopoietic stem cells short term culture DNase imputed pval signal
E035 Primary hematopoietic stem cells DNase imputed pval signal
E051 Primary hematopoietic stem cells G-CSF-mobilized Male DNase imputed pval signal
E124 Monocytes-CD14+ RO01746 Primary Cells DNase imputed pval signal
E029 Primary monocytes from peripheral blood DNase imputed pval signal
E030 Primary neutrophils from peripheral blood DNase imputed pval signal
E122 HUVEC Umbilical Vein Endothelial Primary Cells DNase imputed pval signal
E003 H1 Cells DNase imputed pval signal
E008 H9 Cells DNase imputed pval signal
E014 HUES48 Cells DNase imputed pval signal
E015 HUES6 Cells DNase imputed pval signal
E016 HUES64 Cells DNase imputed pval signal
H3K4me1_Signal
E123 K562 Leukemia Cells H3K4me1 imputed pval signal
E036 Primary hematopoietic stem cells short term culture H3K4me1 imputed pval signal
E035 Primary hematopoietic stem cells H3K4me1 imputed pval signal
E051 Primary hematopoietic stem cells G-CSF-mobilized Male H3K4me1 imputed pval signal
E124 Monocytes-CD14+ RO01746 Primary Cells H3K4me1 imputed pval signal
E029 Primary monocytes from peripheral blood H3K4me1 imputed pval signal
E030 Primary neutrophils from peripheral blood H3K4me1 imputed pval signal
E122 HUVEC Umbilical Vein Endothelial Primary Cells H3K4me1 imputed pval signal
E003 H1 Cells H3K4me1 imputed pval signal
E008 H9 Cells H3K4me1 imputed pval signal
E014 HUES48 Cells H3K4me1 imputed pval signal
E015 HUES6 Cells H3K4me1 imputed pval signal
E016 HUES64 Cells H3K4me1 imputed pval signal
H3K27ac_Signal
E123 K562 Leukemia Cells H3K27ac imputed pval signal
E036 Primary hematopoietic stem cells short term culture H3K27ac imputed pval signal
E035 Primary hematopoietic stem cells H3K27ac imputed pval signal
E051 Primary hematopoietic stem cells G-CSF-mobilized Male H3K27ac imputed pval signal
E124 Monocytes-CD14+ RO01746 Primary Cells H3K27ac imputed pval signal
E029 Primary monocytes from peripheral blood H3K27ac imputed pval signal
E030 Primary neutrophils from peripheral blood H3K27ac imputed pval signal
E122 HUVEC Umbilical Vein Endothelial Primary Cells H3K27ac imputed pval signal
E003 H1 Cells H3K27ac imputed pval signal
E008 H9 Cells H3K27ac imputed pval signal
E014 HUES48 Cells H3K27ac imputed pval signal
E015 HUES6 Cells H3K27ac imputed pval signal
E016 HUES64 Cells H3K27ac imputed pval signal

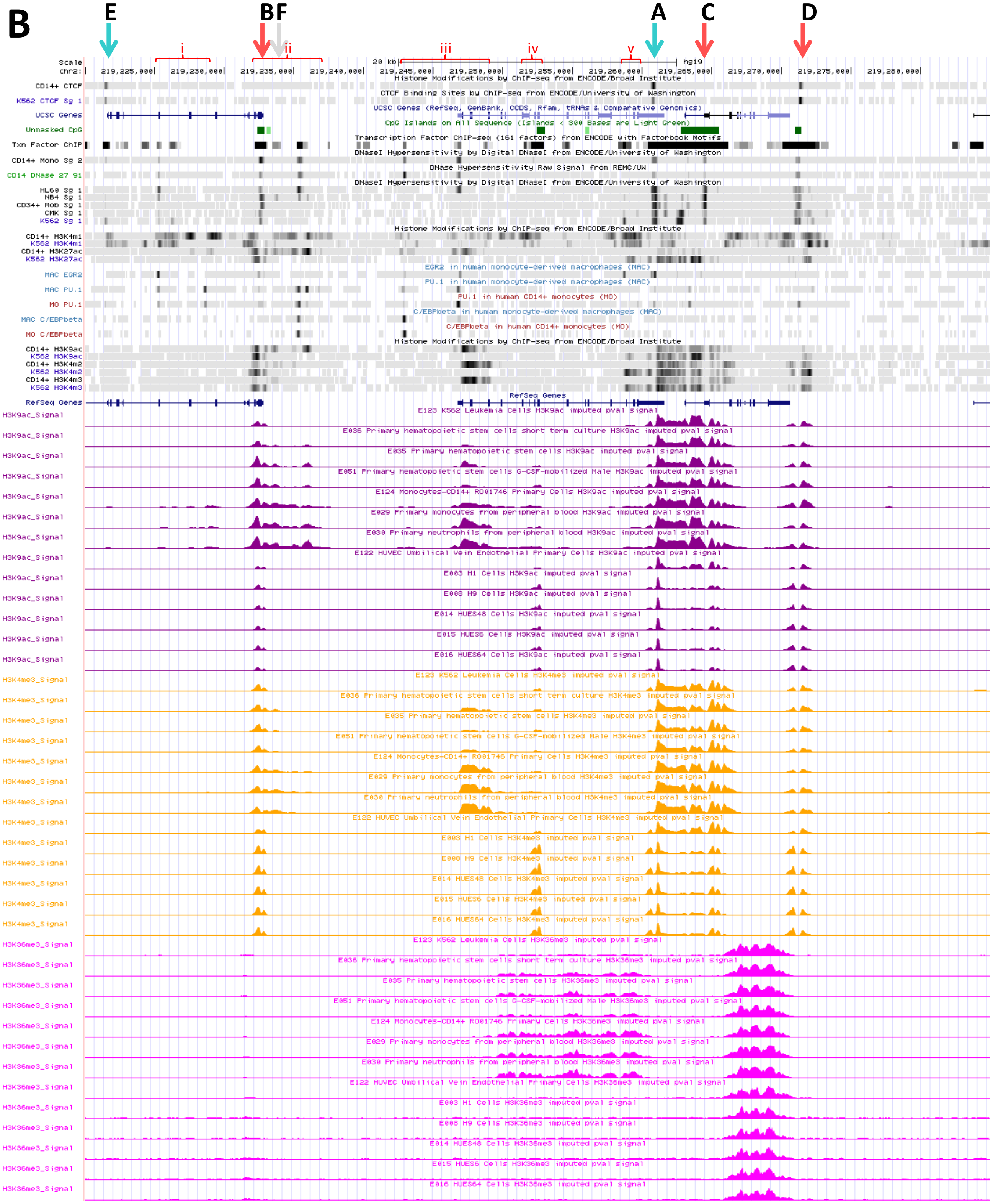
B
E
BF
A
C
D
i
ii
iii
iv
v
Scale
chr2:
20 kb
hg19
219,225,000| 219,230,000| 219,235,000| 219,240,000| 219,245,000| 219,250,000| 219,255,000| 219,260,000| 219,265,000| 219,270,000| 219,275,000| 219,280,000|
Histone Modifications by ChIP-seq from ENCODE/Broad Institute
CD14+ CTCF
CTCF Binding Sites by ChIP-seq from ENCODE/University of Washington
K562 CTCF Sg 1
UCSC Genes (RefSeq, GenBank, CCDS, Rfam, tRNAs & Comparative Genomics)
UCSC Genes
CpG Islands on All Sequence (Islands < 300 Bases are Light Green)
Unmasked CpG
Transcription Factor ChIP-seq (161 factors) from ENCODE with Factorbook Motifs
Txn Factor ChIP
DNaseI Hypersensitivity by Digital DNaseI from ENCODE/University of Washington
CD14+ Mono Sg 2
DNase Hypersensitivity Raw Signal from REMC/UW
CD14 DNase 27 91
DNaseI Hypersensitivity by Digital DNaseI from ENCODE/University of Washington
HL60 Sg 1
NB4 Sg 1
CD34+ Mob Sg 1
CMK Sg 1
K562 Sg 1
Histone Modifications by ChIP-seq from ENCODE/Broad Institute
CD14+ H3K4m1
K562 H3K4m1
CD14+ H3K27ac
K562 H3K27ac
EGR2 in human monocyte-derived macrophages (MAC)
MAC EGR2
PU.1 in human monocyte-derived macrophages (MAC)
MAC PU.1
PU.1 in human CD14+ monocytes (MO)
MO PU.1
C/EBPbeta in human monocyte-derived macrophages (MAC)
MAC C/EBPbeta
C/EBPbeta in human CD14+ monocytes (MO)
MO C/EBPbeta
Histone Modifications by ChIP-seq from ENCODE/Broad Institute
CD14+ H3K9ac
K562 H3K9ac
CD14+ H3K4m2
K562 H3K4m2
CD14+ H3K4m3
K562 H3K4m3
RefSeq Genes
RefSeq Genes
H3K9ac_Signal
E123 K562 Leukemia Cells H3K9ac imputed pval signal
E036 Primary hematopoietic stem cells short term culture H3K9ac imputed pval signal
E035 Primary hematopoietic stem cells H3K9ac imputed pval signal
E051 Primary hematopoietic stem cells G-CSF-mobilized Male H3K9ac imputed pval signal
E124 Monocytes-CD14+ RO01746 Primary Cells H3K9ac imputed pval signal
E029 Primary monocytes from peripheral blood H3K9ac imputed pval signal
E030 Primary neutrophils from peripheral blood H3K9ac imputed pval signal
E122 HUVEC Umbilical Vein Endothelial Primary Cells H3K9ac imputed pval signal
E003 H1 Cells H3K9ac imputed pval signal
E008 H9 Cells H3K9ac imputed pval signal
E014 HUES48 Cells H3K9ac imputed pval signal
E015 HUES6 Cells H3K9ac imputed pval signal
E016 HUES64 Cells H3K9ac imputed pval signal
H3K4me3_Signal
E123 K562 Leukemia Cells H3K4me3 imputed pval signal
E036 Primary hematopoietic stem cells short term culture H3K4me3 imputed pval signal
E035 Primary hematopoietic stem cells H3K4me3 imputed pval signal
E051 Primary hematopoietic stem cells G-CSF-mobilized Male H3K4me3 imputed pval signal
E124 Monocytes-CD14+ RO01746 Primary Cells H3K4me3 imputed pval signal
E029 Primary monocytes from peripheral blood H3K4me3 imputed pval signal
E030 Primary neutrophils from peripheral blood H3K4me3 imputed pval signal
E122 HUVEC Umbilical Vein Endothelial Primary Cells H3K4me3 imputed pval signal
E003 H1 Cells H3K4me3 imputed pval signal
E008 H9 Cells H3K4me3 imputed pval signal
E014 HUES48 Cells H3K4me3 imputed pval signal
E015 HUES6 Cells H3K4me3 imputed pval signal
E016 HUES64 Cells H3K4me3 imputed pval signal
H3K36me3_Signal
E123 K562 Leukemia Cells H3K36me3 imputed pval signal
E036 Primary hematopoietic stem cells short term culture H3K36me3 imputed pval signal
E035 Primary hematopoietic stem cells H3K36me3 imputed pval signal
E051 Primary hematopoietic stem cells G-CSF-mobilized Male H3K36me3 imputed pval signal
E124 Monocytes-CD14+ RO01746 Primary Cells H3K36me3 imputed pval signal
E029 Primary monocytes from peripheral blood H3K36me3 imputed pval signal
E030 Primary neutrophils from peripheral blood H3K36me3 imputed pval signal
E122 HUVEC Umbilical Vein Endothelial Primary Cells H3K36me3 imputed pval signal
E003 H1 Cells H3K36me3 imputed pval signal
E008 H9 Cells H3K36me3 imputed pval signal
E014 HUES48 Cells H3K36me3 imputed pval signal
E015 HUES6 Cells H3K36me3 imputed pval signal
E016 HUES64 Cells H3K36me3 imputed pval signal

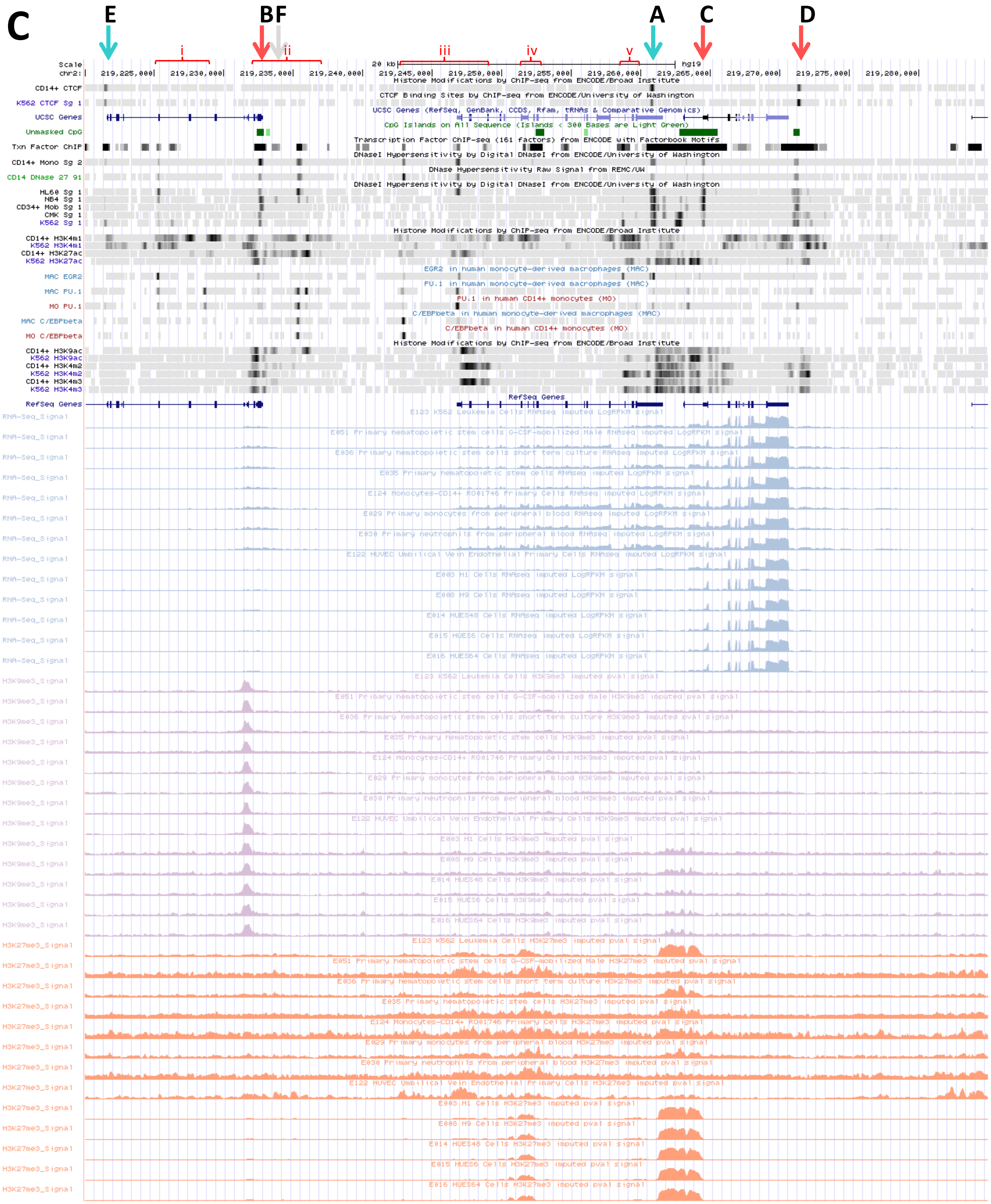
C
E
BF
A
C
D
i
ii
iii
iv
v
Scale
chr2:
20 kb
hg19
219,225,000
219,230,000
219,235,000
219,240,000
219,245,000
219,250,000
219,255,000
219,260,000
219,265,000
219,270,000
219,275,000
219,280,000
Histone Modifications by ChIP-seq from ENCODE/Broad Institute
CD14+ CTCF
CTCF Binding Sites by ChIP-seq from ENCODE/University of Washington
K562 CTCF Sg 1
UCSC Genes (RefSeq, GenBank, CCDS, Rfam, tRNAs & Comparative Genomics)
UCSC Genes
CpG Islands on All Sequence (Islands < 300 Bases are Light Green)
Unmasked CpG
Transcription Factor ChIP-seq (161 factors) from ENCODE with Factorbook Motifs
Txn Factor ChIP
DNaseI Hypersensitivity by Digital DNaseI from ENCODE/University of Washington
CD14+ Mono Sg 2
DNase Hypersensitivity Raw Signal from REMC/UW
CD14 DNase 27 91
HL60 Sg 1
NB4 Sg 1
CD34+ Mob Sg 1
CMK Sg 1
K562 Sg 1
CD14+ H3K4m1
K562 H3K4m1
CD14+ H3K27ac
K562 H3K27ac
EGR2 in human monocyte-derived macrophages (MAC)
MAC EGR2
PU.1 in human monocyte-derived macrophages (MAC)
MAC PU.1
PU.1 in human CD14+ monocytes (MO)
MO PU.1
C/EBPbeta in human monocyte-derived macrophages (MAC)
MAC C/EBPbeta
C/EBPbeta in human CD14+ monocytes (MO)
MO C/EBPbeta
CD14+ H3K9ac
K562 H3K9ac
CD14+ H3K4m2
K562 H3K4m2
CD14+ H3K4m3
K562 H3K4m3
RefSeq Genes
RNA-Seq_Signal
H3K9me3_Signal
H3K27me3_Signal
E123 K562 Leukemia Cells RNAseq imputed LogRPKM signal
E051 Primary hematopoietic stem cells G-CSF-mobilized Male RNAseq imputed LogRPKM signal
E036 Primary hematopoietic stem cells short term culture RNAseq imputed LogRPKM signal
E035 Primary hematopoietic stem cells RNAseq imputed LogRPKM signal
E124 Monocytes-CD14+ RO01746 Primary Cells RNAseq imputed LogRPKM signal
E029 Primary monocytes from peripheral blood RNAseq imputed LogRPKM signal
E030 Primary neutrophils from peripheral blood RNAseq imputed LogRPKM signal
E122 HUVEC Umbilical Vein Endothelial Primary Cells RNAseq imputed LogRPKM signal
E003 H1 Cells RNAseq imputed LogRPKM signal
E008 H9 Cells RNAseq imputed LogRPKM signal
E014 HUES48 Cells RNAseq imputed LogRPKM signal
E015 HUES6 Cells RNAseq imputed LogRPKM signal
E016 HUES64 Cells RNAseq imputed LogRPKM signal
E123 K562 Leukemia Cells H3K9me3 imputed pval signal
E051 Primary hematopoietic stem cells G-CSF-mobilized Male H3K9me3 imputed pval signal
E036 Primary hematopoietic stem cells short term culture H3K9me3 imputed pval signal
E035 Primary hematopoietic stem cells H3K9me3 imputed pval signal
E124 Monocytes-CD14+ RO01746 Primary Cells H3K9me3 imputed pval signal
E029 Primary monocytes from peripheral blood H3K9me3 imputed pval signal
E030 Primary neutrophils from peripheral blood H3K9me3 imputed pval signal
E122 HUVEC Umbilical Vein Endothelial Primary Cells H3K9me3 imputed pval signal
E003 H1 Cells H3K9me3 imputed pval signal
E008 H9 Cells H3K9me3 imputed pval signal
E014 HUES48 Cells H3K9me3 imputed pval signal
E015 HUES6 Cells H3K9me3 imputed pval signal
E016 HUES64 Cells H3K9me3 imputed pval signal
E123 K562 Leukemia Cells H3K27me3 imputed pval signal
E051 Primary hematopoietic stem cells G-CSF-mobilized Male H3K27me3 imputed pval signal
E036 Primary hematopoietic stem cells short term culture H3K27me3 imputed pval signal
E035 Primary hematopoietic stem cells H3K27me3 imputed pval signal
E124 Monocytes-CD14+ RO01746 Primary Cells H3K27me3 imputed pval signal
E029 Primary monocytes from peripheral blood H3K27me3 imputed pval signal
E030 Primary neutrophils from peripheral blood H3K27me3 imputed pval signal
E122 HUVEC Umbilical Vein Endothelial Primary Cells H3K27me3 imputed pval signal
E003 H1 Cells H3K27me3 imputed pval signal
E008 H9 Cells H3K27me3 imputed pval signal
E014 HUES48 Cells H3K27me3 imputed pval signal
E015 HUES6 Cells H3K27me3 imputed pval signal
E016 HUES64 Cells H3K27me3 imputed pval signal

Figure S17. Hypothetical CTCF-dependent topology of *NRAMP1* locus and hematopoietic determinants of gene expression (see text for details, sections 2.3.3. and 2.3.4.). *NRAMP1* locus boundaries (CTCF sites A and E, "R" orientation, shown in black) are represented interacting with compatible and preferred upstream CTCF sites ("F" orientation, indicated as 157 and 166, respectively). Other CTCF sites are indicated in grey ("F" or undetermined orientation: B-D and F, respectively). DHS F1-F14 are colored depending on temporal order of mobilization: purple, light purple, pink and red. It is proposed that *NRAMP1* (*SLC11A1*) locus activation results from successive steps: i) F12/5'RH (CTCF_B) and F9/3'RH are partially mobilized in non-hematopoietic cell types; ii) initiation of hematopoiesis may erase negative histone marks leading to increased *CTDSP1* transcription -a process that may result from formation of a CTCF-dependent loop involving sites F and C (orange dotted arrow); iii) myeloid-specific TF-dependent mobilization of F12/5' RH, F13 and co-activation of sites F6, F10-F3 may prime the locus while preventing gene expression; iv) as differentiation progresses, specific myelo-monocytic TFs activate elements F5, F7 and F9 leading to disruption of CTCF_F-C loop and allowing contacts between distal regulatory elements and NR1 TSS (F1), through Mediator based interactions for instance, which activate *NRAMP1* transcription (green dotted arrow); and v) terminal differentiation into mature phagocytes leads to extensive priming/activation of the locus, including elements F11, F4A, F2, F4B, F8 and F14, and further modulation by signal-dependent TFs (e.g., tissue microenvironment, infections...). According to this hypothesis, CTCF determinants define *NRAMP1* locus (TAD); hematopoietic TFs are required for transcriptional activation of the locus 3' end; myeloid TFs allow *NRAMP1* locus priming for subsequent activation while myelo-monocytic TFs mediate *NRAMP1* expression and/or signal-dependent regulation.

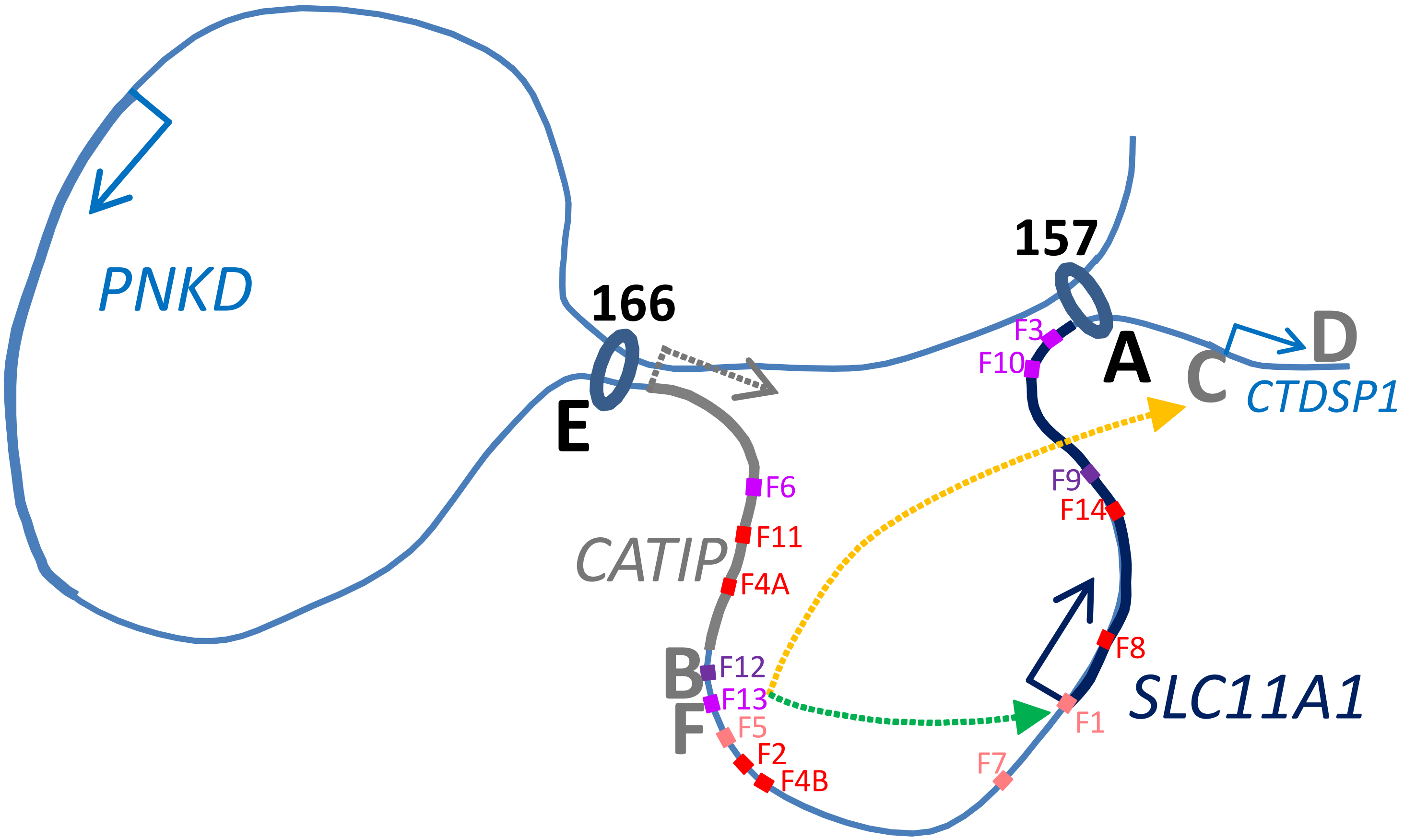

PNKD
166
E
157
F3
F10
A
C
D
CTDSP1
F6
F11
CATIP
F4A
F9
F14
B
F12
F13
F
F5
F2
F4B
F8
SLC11A1
F1
F7

Figure S18. *NRAMP1* locus region i and ii represent candidate transcriptional regulatory determinants mediating innate memory in response to microbial stimuli from various origins (*Saccharomyces cerevisiae* β(1,3)D-glucan, BG, and *Escherichia coli* lipopolysaccharide, LPS) [128]. *From top to bottom*: Major CTCF sites E, A and D (blue and red arrows correspond to reverse and forward orientations, respectively); gene names and position of *NRAMP1* TSS; candidate *NRAMP1* regulatory regions i-v, including DHS F1-F14; chromosome 2 scale; CpG islands; ENCODE CTCF-specific ChIP-seq data in CD14+ MNs; histone modification-specific ChIP-seq (K4me1, K27ac, K4me3) and Tn5 transposase accessibility (ATAC-seq) data for MNs stimulated with: a) LPS, for 24h and analyzed five days after (LPS_D6), b) BG, for 24h and analyzed five days after (BG_D6, c) no additive, for 24h and analyzed five days after (RPMI_D6), d) LPS, for 24h (LPS_D1), e) BG, for 24h (BG_D1) and f) no additive, for 24h (RPMI_D1), as well as for freshly explanted MNs (RPMI_0); ENCODE DHS (x2) and ChIP-seq analyses for similar histone modifications in CD14+ MNs (K4me1, K27ac, K4me3); UCSC genes; ChIP-seq data for the TFs EGR2, PU.1 and C/EBPb in MO and MAC [13]; ENCODE CTCF ChIP-seq data in K562 cells; ENCODE TF-specific ChIP-seq data; delineation of *NRAMP1* regions i-v.

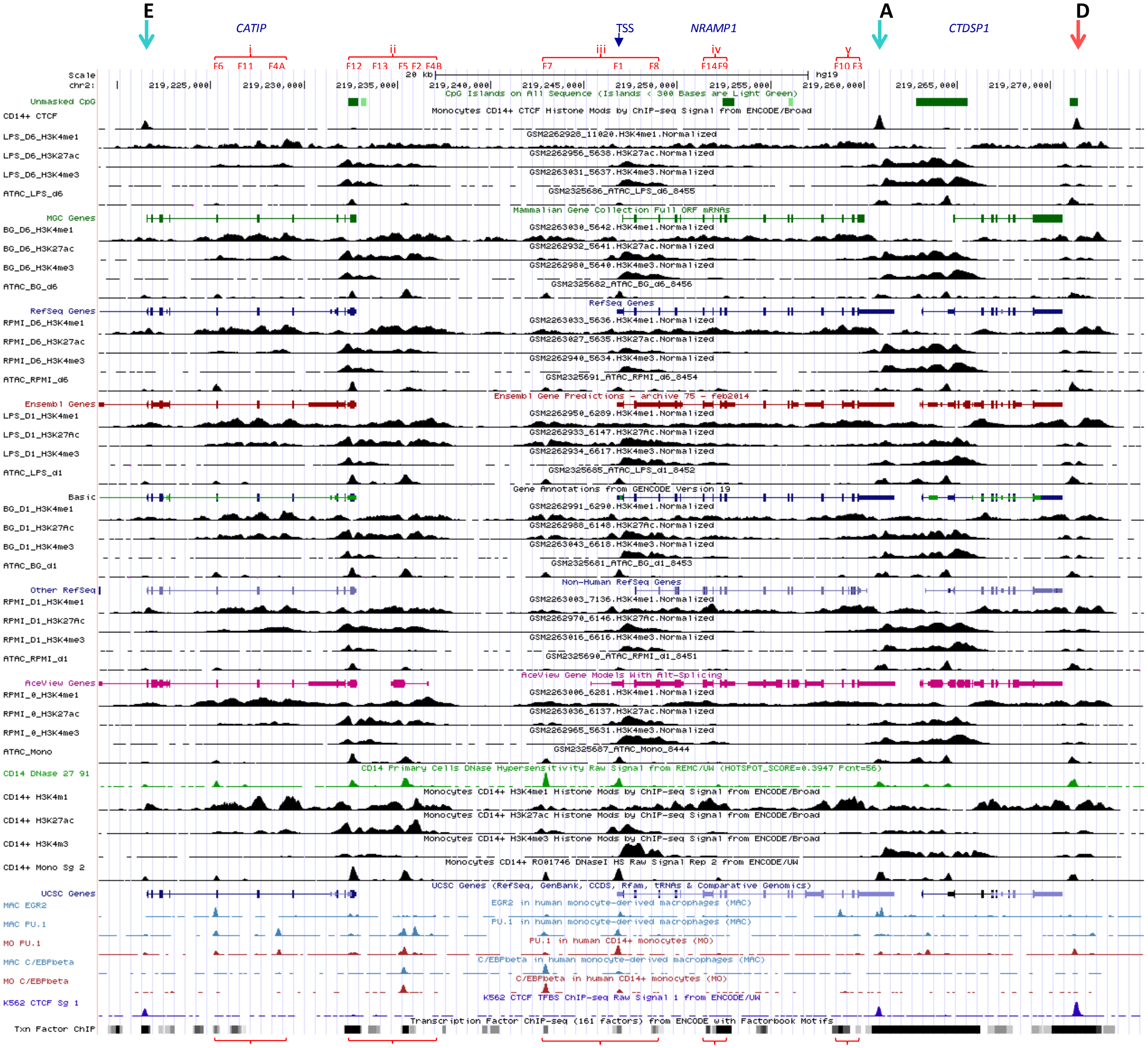

E
CATIP
TSS
NRAMP1
A
CTDSP1
D
i
ii
iii
iv
v
F6 F11 F4A
F12 F13 F5 F2 F4B
F7 F1 F8
F14F9
F10 F3
Scale
chr2:
20 kb
hg19
219,225,000
219,230,000
219,235,000
219,240,000
219,245,000
219,250,000
219,255,000
219,260,000
219,265,000
219,270,000
Unmasked CpG
CpG Islands on All Sequence (Islands < 300 Bases are Light Green)
CD14+ CTCF
Monocytes CD14+ CTCF Histone Mods by ChIP-seq Signal from ENCODE/Broad
LPS_D6_H3K4me1
LPS_D6_H3K27ac
LPS_D6_H3K4me3
ATAC_LPS_d6
MGC Genes
Mammalian Gene Collection Full ORF mRNAs
BG_D6_H3K4me1
BG_D6_H3K27ac
BG_D6_H3K4me3
ATAC_BG_d6
RefSeq Genes
RPMI_D6_H3K4me1
RPMI_D6_H3K27ac
RPMI_D6_H3K4me3
ATAC_RPMI_d6
Ensembl Genes
Ensembl Gene Predictions - archive 75 - feb2014
LPS_D1_H3K4me1
LPS_D1_H3K27Ac
LPS_D1_H3K4me3
ATAC_LPS_d1
Basic
Gene Annotations from GENCODE Version 19
BG_D1_H3K4me1
BG_D1_H3K27Ac
BG_D1_H3K4me3
ATAC_BG_d1
Other RefSeq
Non-Human RefSeq Genes
RPMI_D1_H3K4me1
RPMI_D1_H3K27Ac
RPMI_D1_H3K4me3
ATAC_RPMI_d1
AceView Genes
AceView Gene Models With Alt-Splicing
RPMI_0_H3K4me1
RPMI_0_H3K27ac
RPMI_0_H3K4me3
ATAC_Mono
CD14 DNase 27 91
CD14 Primary Cells DNase Hypersensitivity Raw Signal from REMC/UW (HOTSPOT_SCORE=0.3947 Pcnt=56)
CD14+ H3K4m1
Monocytes CD14+ H3K4me1 Histone Mods by ChIP-seq Signal from ENCODE/Broad
CD14+ H3K27ac
Monocytes CD14+ H3K27ac Histone Mods by ChIP-seq Signal from ENCODE/Broad
CD14+ H3K4m3
Monocytes CD14+ H3K4me3 Histone Mods by ChIP-seq Signal from ENCODE/Broad
CD14+ Mono Sg 2
Monocytes CD14+ RO01746 DNaseI HS Raw Signal Rep 2 from ENCODE/UW
UCSC Genes
UCSC Genes (RefSeq, GenBank, CCDS, Rfam, tRNAs & Comparative Genomics)
MAC EGR2
EGR2 in human monocyte-derived macrophages (MAC)
MAC PU.1
PU.1 in human monocyte-derived macrophages (MAC)
MO PU.1
PU.1 in human CD14+ monocytes (MO)
MAC C/EBPbeta
C/EBPbeta in human monocyte-derived macrophages (MAC)
MO C/EBPbeta
C/EBPbeta in human CD14+ monocytes (MO)
K562 CTCF Sg 1
K562 CTCF TFBS ChIP-seq Raw Signal 1 from ENCODE/UW
Txn Factor ChIP
Transcription Factor ChIP-seq (161 factors) from ENCODE with Factorbook Motifs

Figure S19. DHS and binding of RUNX1 at *NRAMP1* locus in FLT3-ITD AML patients [139]. *From top to bottom*: chromosome 2 scale; RefSeq gene descriptions; DHS in CD14⁺ MNs, CD34⁺ peripheral blood stem cells (PBSC), FLT3-ITD AMLs (x3) and MNs; RUNX1-specific ChIP-seq in CD34⁺ cells and FLT3-ITD AMLs (x2); DHS in HL-60 cells; GENCODE gene descriptions; CpG islands.

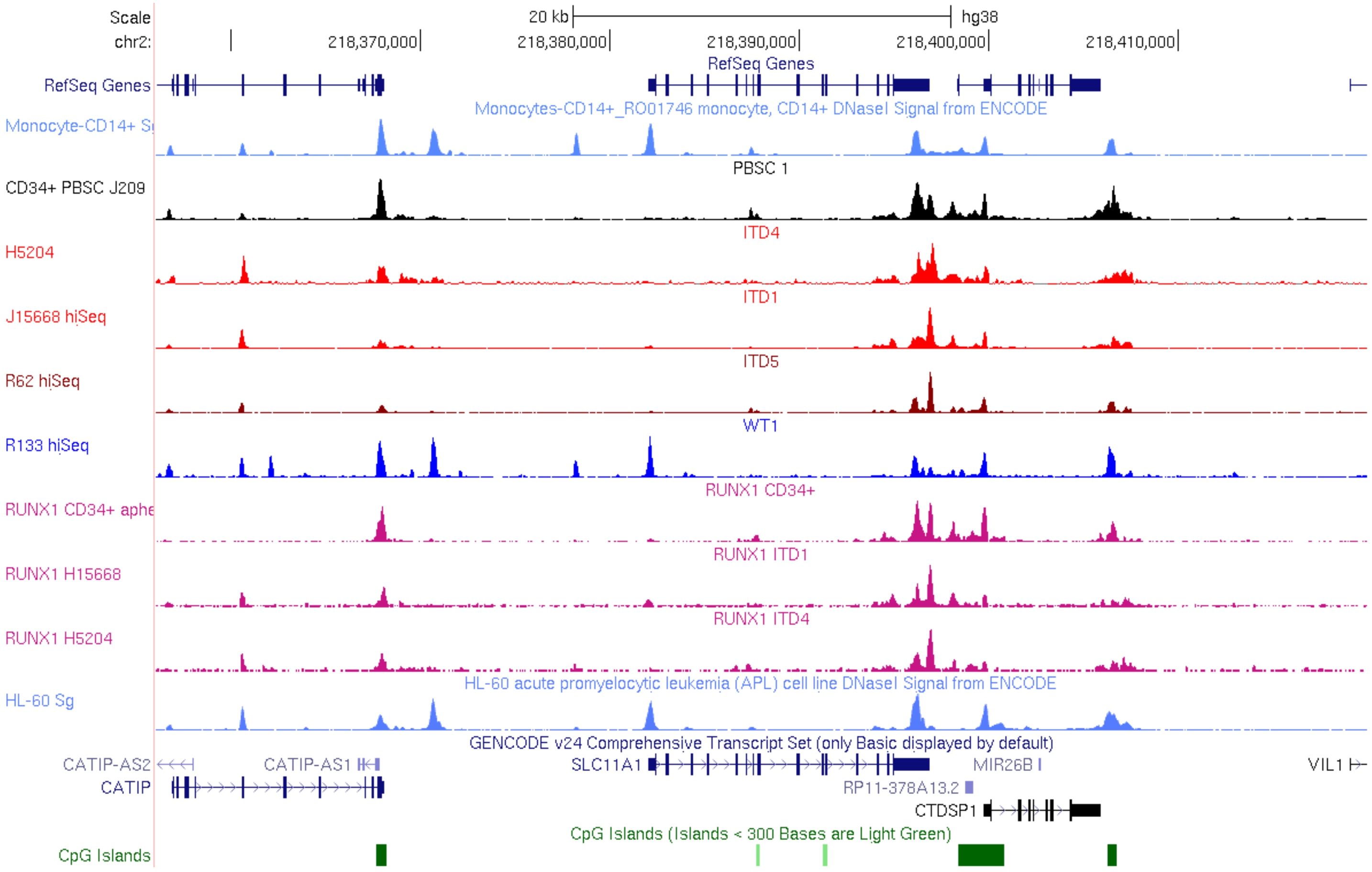

Figure S20. Effect of all *trans* retinoic acid ATRA on *NRAMP1* locus activity in AML (TSU1681MT) and APL (NB4). **A**. Gene expression and TF-specific ChIP-seq data for TSU-1621-MT AML [141]. *From top to bottom*: Chromosome 2 scale; DHS for HL-60 (M2 AML) and NB4 (M3 AML/APL); CpG islands; RNA-seq data in cells expressing ERG or not; ChIP-seq data for FLi1, RUNX1/AML1, SPI1/PU.1; FUS, ERG and RNA Pol II in cells treated with ATRA or not; UCSC gene descriptions. **B**. *NRAMP1* activity in NB4 cells ATRA-treated or not [146]. *From top to bottom*: ChIP-seq for RNA Pol II; RNA-seq data; ChIP-seq for K9me3; nuclease accessibility (*Nla* III; *Hpa* II, sensitive to DNA methylation); DNA methylation; ChIP-seq for K9/14ac; ChIP-seq data in untreated NB4 cells: p300 (HAT), H2A.Zac and K4me3. DHS in NB4 and HL-60 cells; ENCODE TF-ChIP-seq data; RefSeq gene depictions.

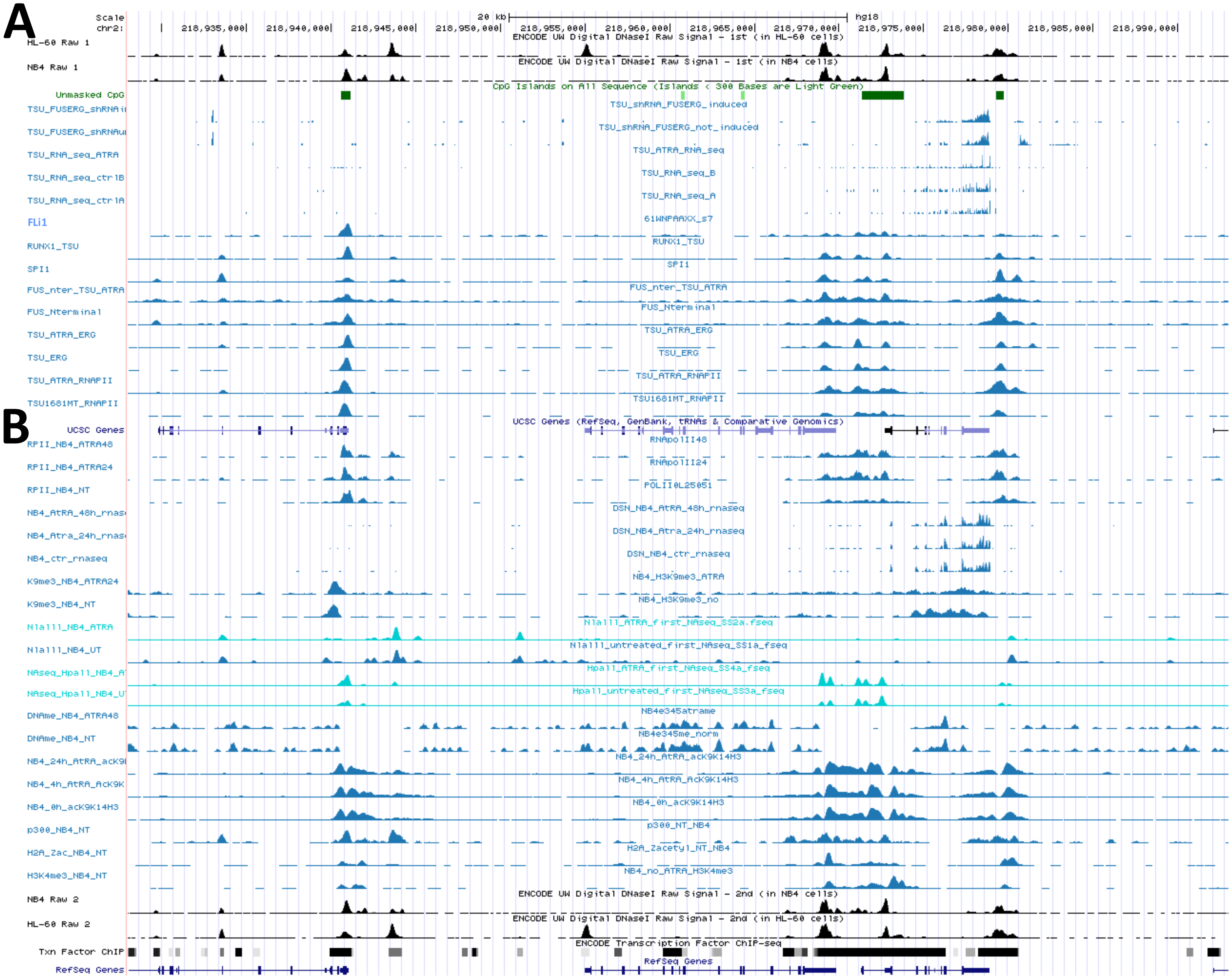
A
B
Scale
chr2:
20 kb
hg18
ENCODE UW Digital DNaseI Raw Signal - 1st (in HL-60 cells)
HL-60 Raw 1
ENCODE UW Digital DNaseI Raw Signal - 1st (in NB4 cells)
NB4 Raw 1
CpG Islands on All Sequence (Islands < 300 Bases are Light Green)
Unmasked CpG
TSU_shRNA_FUSERG_induced
TSU_shRNA_FUSERG_not_induced
TSU_ATRA_RNA_seq
TSU_RNA_seq_ATRA
TSU_RNA_seq_B
TSU_RNA_seq_A
FLi1
RUNX1_TSU
SPI1
FUS_nter_TSU_ATRA
FUS_Nterminal
TSU_ATRA_ERG
TSU_ERG
TSU_ATRA_RNAPII
TSU1681MT_RNAPII
UCSC Genes (RefSeq, GenBank, tRNAs & Comparative Genomics)
UCSC Genes
RNApolII48
RNApolII24
K9me3_NB4_ATRA24
K9me3_NB4_NT
NB4_H3K9me3_ATRA
NB4_H3K9me3_no
DNAme_NB4_ATRA48
DNAme_NB4_NT
NB4_24h_AtRA_acK9K14H3
NB4_4h_AtRA_AcK9K14H3
NB4_0h_acK9K14H3
p300_NB4_NT
H2A_Zac_NB4_NT
H3K4me3_NB4_NT
NB4_no_ATRA_H3K4me3
ENCODE UW Digital DNaseI Raw Signal - 2nd (in NB4 cells)
NB4 Raw 2
ENCODE UW Digital DNaseI Raw Signal - 2nd (in HL-60 cells)
HL-60 Raw 2
ENCODE Transcription Factor ChIP-seq
Txn Factor ChIP
RefSeq Genes

Figure S21. *NRAMP1* activity in Kasumi AML [142–44]. *From top to bottom*: chromosome 2 scale; CTCF-specific ChIP-seq ($CD14^+$ MNs); RNA-seq for Kasumi cells expressing ERG or not; UCSC gene descriptions; acetylated histone-specific ChIP-seq data: K9/14ac in cells treated with MS275 (HDAC inhibitor; for 24, 4, or 0h); H4ac (untreated cells); ENCODE TF-specific ChIP-seq data; ChIP-seq for HDAC1 and HDAC2, N-CoR, HAT p300 (x2), GATA, LYL1, FLi1, TAL1, RUNX1/AML1 (AML blasts, Kasumi (x2) and NB4 cells; CpG islands; ChIP-seq for AML1-ETO fusion (x5); ENCODE DHS in $CD14^+$ MNs (x2), HL-60 and NB4 AMLs, AML patients (2), mCD34, normal $CD34^+$ HSPCs, CMK and K562 cells; RefSeq genes; CTCF-specific ChIP-seq (K562 cells); K4me1 and K27ac ChIP-seq data ($CD14^+$ MNs and K562 cells); ChIP-seq for the TFs EGR2, PU.1 and C/EBPb in MN and MF [13]; K9ac, K4me2 and K4me3 ChIP-seq data ($CD14^+$ MNs and K562 cells).

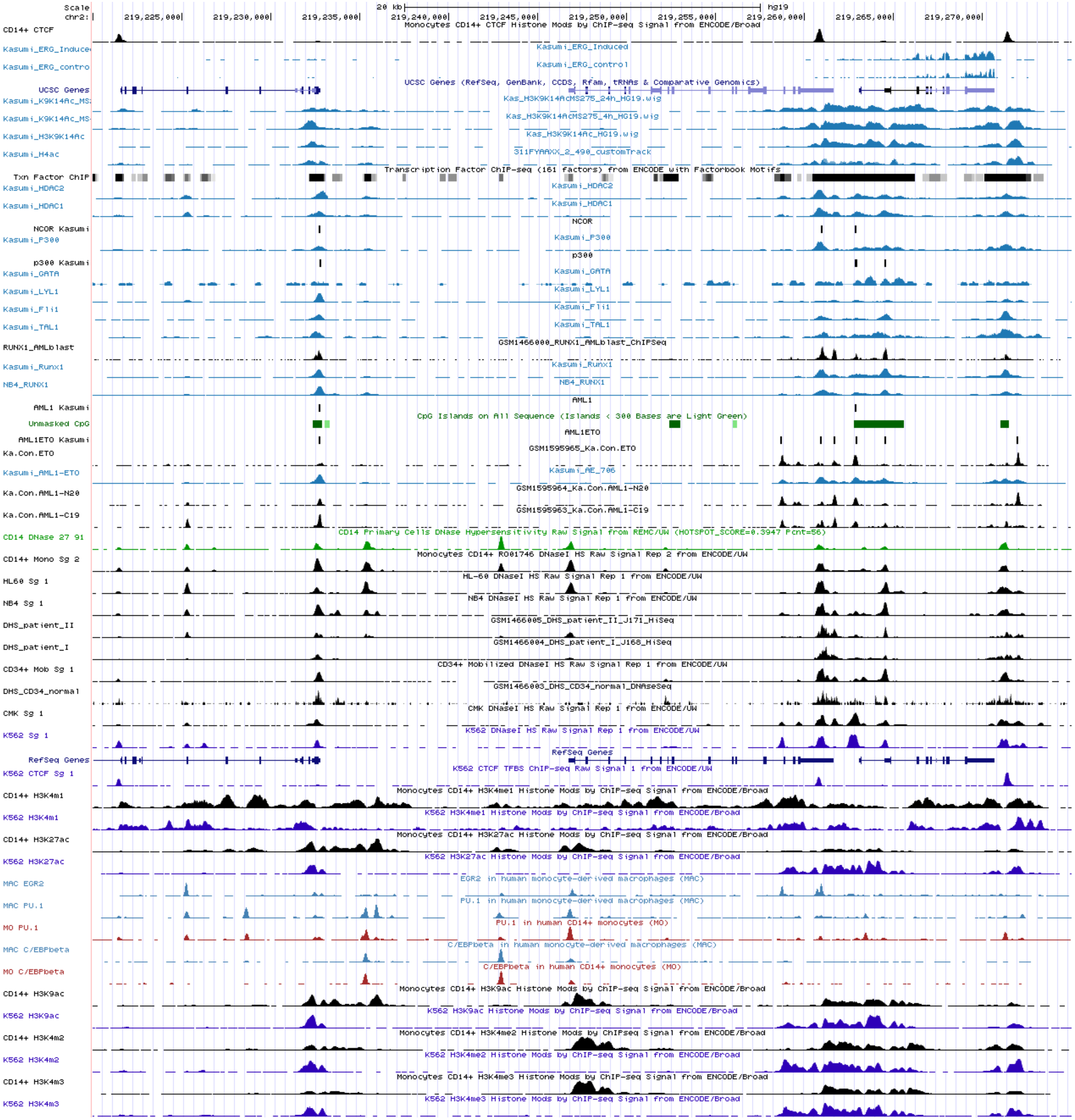
Scale
chr2:
20 kb
hg19
219,225,000
219,230,000
219,235,000
219,240,000
219,245,000
219,250,000
219,255,000
219,260,000
219,265,000
219,270,000
CD14+ CTCF
Monocytes CD14+ CTCF Histone Mods by ChIP-seq Signal from ENCODE/Broad
Kasumi_ERG_Induced
Kasumi_ERG_control
UCSC Genes
UCSC Genes (RefSeq, GenBank, CCDS, Rfam, tRNAs & Comparative Genomics)
Kas_H3K9K14AcMS275_24h_HG19.wig
Kas_H3K9K14AcMS275_4h_HG19.wig
Kasumi_H3K9K14Ac
Kas_H3K9K14Ac_HG19.wig
Kasumi_H4ac
311FYAAXX_2_490_customTrack
Txn Factor ChIP
Transcription Factor ChIP-seq (161 factors) from ENCODE with Factorbook Motifs
Kasumi_HDAC2
Kasumi_HDAC1
NCOR Kasumi
NCOR
Kasumi_P300
p300 Kasumi
p300
Kasumi_GATA
Kasumi_LYL1
Kasumi_Fli1
Kasumi_TAL1
RUNX1_AMLblast
GSM1466000_RUNX1_AMLblast_ChIPSeq
Kasumi_Runx1
NB4_RUNX1
AML1 Kasumi
AML1
Unmasked CpG
CpG Islands on All Sequence (Islands < 300 Bases are Light Green)
AML1ETO Kasumi
AML1ETO
Ka.Con.ETO
GSM1595965_Ka.Con.ETO
Kasumi_AML1-ETO
Kasumi_AE_706
Ka.Con.AML1-N20
GSM1595964_Ka.Con.AML1-N20
Ka.Con.AML1-C19
GSM1595963_Ka.Con.AML1-C19
CD14 DNase 27 91
CD14 Primary Cells DNase Hypersensitivity Raw Signal from REMC/UW (HOTSPOT_SCORE=0.3947 Pcnt=56)
CD14+ Mono Sg 2
Monocytes CD14+ RO01746 DNaseI HS Raw Signal Rep 2 from ENCODE/UW
HL60 Sg 1
HL-60 DNaseI HS Raw Signal Rep 1 from ENCODE/UW
NB4 Sg 1
NB4 DNaseI HS Raw Signal Rep 1 from ENCODE/UW
DHS_patient_II
GSM1466005_DHS_patient_II_J171_HiSeq
DHS_patient_I
GSM1466004_DHS_patient_I_J168_HiSeq
CD34+ Mob Sg 1
CD34+ Mobilized DNaseI HS Raw Signal Rep 1 from ENCODE/UW
DHS_CD34_normal
GSM1466003_DHS_CD34_normal_DNAseSeq
CMK Sg 1
CMK DNaseI HS Raw Signal Rep 1 from ENCODE/UW
K562 Sg 1
K562 DNaseI HS Raw Signal Rep 1 from ENCODE/UW
RefSeq Genes
K562 CTCF Sg 1
K562 CTCF TFBS ChIP-seq Raw Signal 1 from ENCODE/UW
CD14+ H3K4m1
Monocytes CD14+ H3K4me1 Histone Mods by ChIP-seq Signal from ENCODE/Broad
K562 H3K4m1
K562 H3K4me1 Histone Mods by ChIP-seq Signal from ENCODE/Broad
CD14+ H3K27ac
Monocytes CD14+ H3K27ac Histone Mods by ChIP-seq Signal from ENCODE/Broad
K562 H3K27ac
K562 H3K27ac Histone Mods by ChIP-seq Signal from ENCODE/Broad
MAC EGR2
EGR2 in human monocyte-derived macrophages (MAC)
MAC PU.1
PU.1 in human monocyte-derived macrophages (MAC)
MO PU.1
PU.1 in human CD14+ monocytes (MO)
MAC C/EBPbeta
C/EBPbeta in human monocyte-derived macrophages (MAC)
MO C/EBPbeta
C/EBPbeta in human CD14+ monocytes (MO)
CD14+ H3K9ac
Monocytes CD14+ H3K9ac Histone Mods by ChIP-seq Signal from ENCODE/Broad
K562 H3K9ac
K562 H3K9ac Histone Mods by ChIP-seq Signal from ENCODE/Broad
CD14+ H3K4m2
Monocytes CD14+ H3K4me2 Histone Mods by ChIPseq Signal from ENCODE/Broad
K562 H3K4m2
K562 H3K4me2 Histone Mods by ChIP-seq Signal from ENCODE/Broad
CD14+ H3K4m3
Monocytes CD14+ H3K4me3 Histone Mods by ChIP-seq Signal from ENCODE/Broad
K562 H3K4m3
K562 H3K4me3 Histone Mods by ChIP-seq Signal from ENCODE/Broad

Figure S22. TF-specific ChIP-seq data for ME-1 AML [144]. *From top to bottom*: chromosome 2 scale; RefSeq genes; ENCODE TF-specific ChIP-seq data; DHS for HL-60 and NB4 APLs; ChIP-seq data specific for ERG, FLi1, TAL1, HEB, ELF1, PU.1, HDAC1, K9K14ac, HAT p300, TBP, RNA Pol II; DHS for NB4 and HL-60 APLs; CpG islands; RefSeq gene depictions.

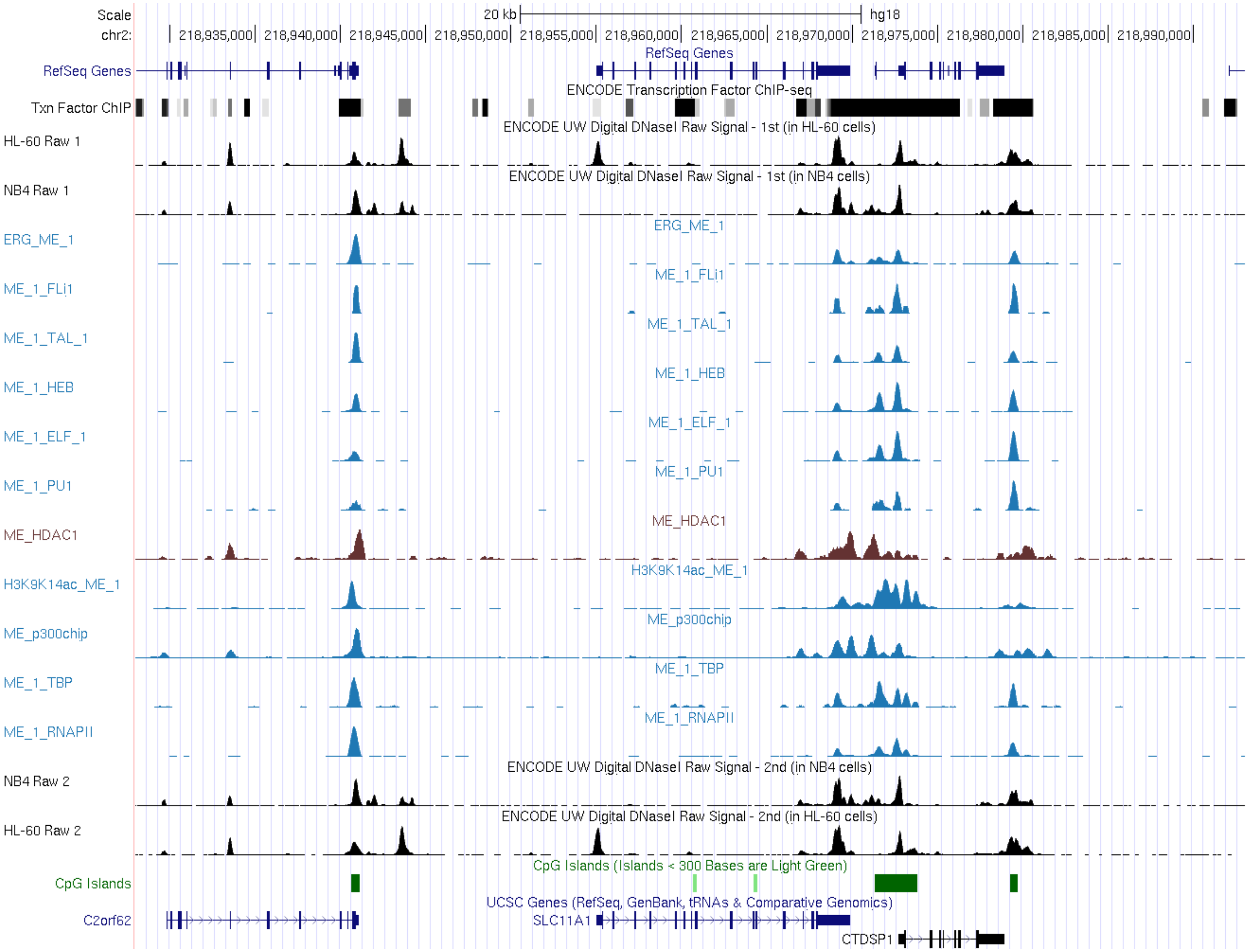

Figure S23. mRNA levels induced by 28 stimulation conditions producing MF phenotypes distributing across M1-M2 spectrum and ranked according to *NRAMP1* mRNA level (log2 transformed values, blue), decreasing from left to right, except for basal conditions (M-CSF or GM-CSF), at most left. **A**. Expression values are represented for *NRAMP1* and two other genes: *FTH1* (red) and *NCOA4* (green). **B**. Superimposition on *NRAMP1* profile of expression values for *S100A8* (red) and *S100A9* (green). **C**. Comparison of multiple profiles: *NRAMP1* (blue), *CD163* (purple), *MCOLN1* (red), *HMOX1* (cyan), *CYBRD1* (orange), *SLC40A1* (green) [132].

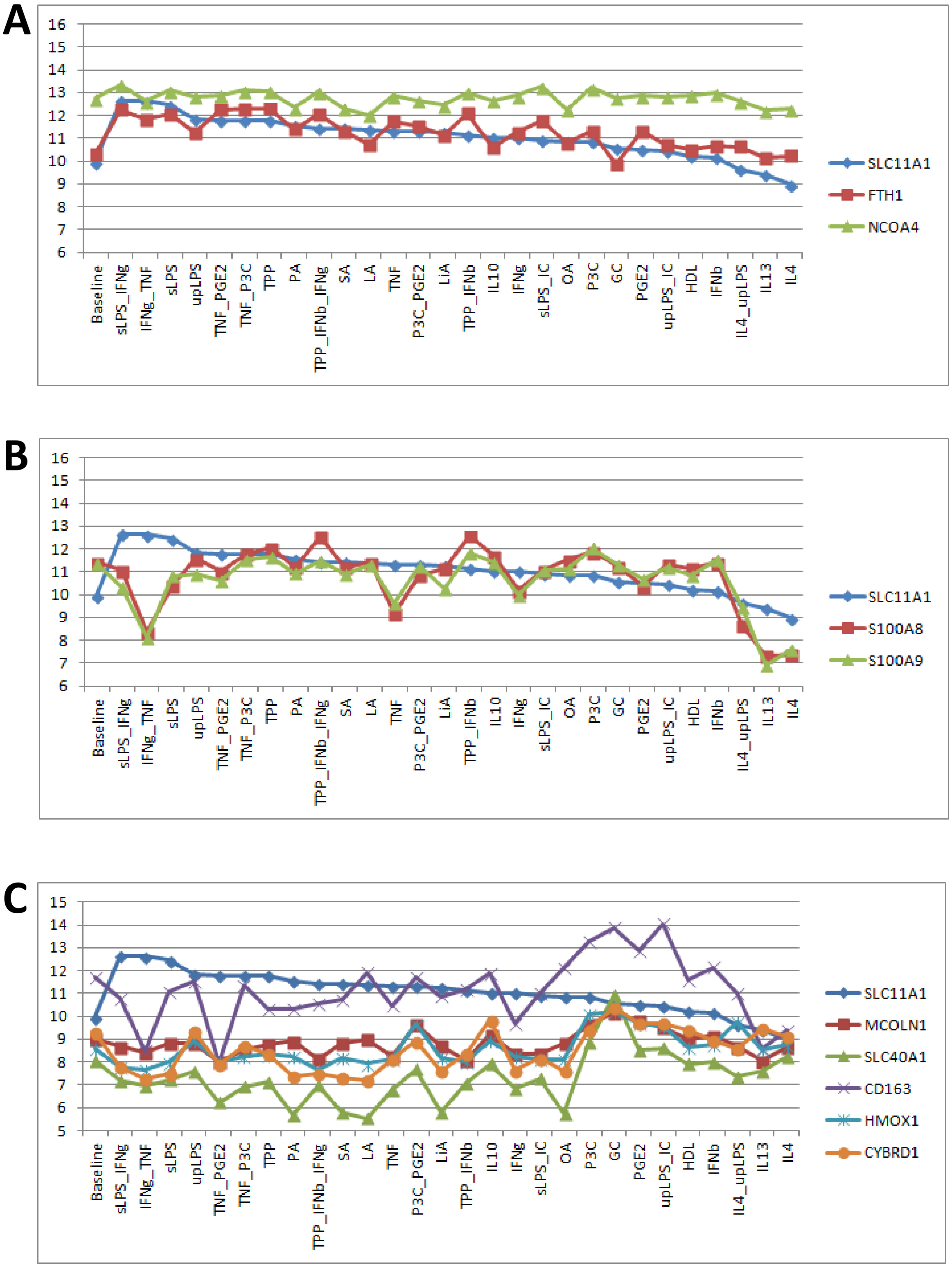

(next three pages)
Figure S24. RNA-seq analysis of gene expression during mouse embryogenesis and organogenesis: *Nramp1* (**A**), *Emr1* (*F4/80*; **B**) and *Cx3cr1* (**C**) [118]. Transcript levels for the genes *Catip*, *Nramp1*/*Slc11a1* and *Ctdsp1*, across several developmental steps, from embryo to adult, including i) tissue MFs (Mac E10.25 and E10.5 (YS, HB, LIV, SKI); Mac E12.5 (HB, LIV, SKI); Mac E14.5 and E18.5 (HB, LIV, SKI, KID, LUN); Mac P2, P8 and P21 (HB, LIV, SKI, KID, LUN)) and ii) their precursors (YS EMP E9 and E10.25; LIV EMP 10.25; pMac E9.5 (YS, HB, SKI); pMac E10.25 (YS, HB, LIV, SKI)). *Cx3cr1* expression is the most precocious (starting in EMP), followed by *Nramp1* (beginning in pMac) and *Emr1* (*F4/80*; first detected in tissue Mac). Also, *Nramp1* expression is specifically down regulated in MFs of adult skin and lung (P8, P21 and P2, P8 and P21, respectively).

A

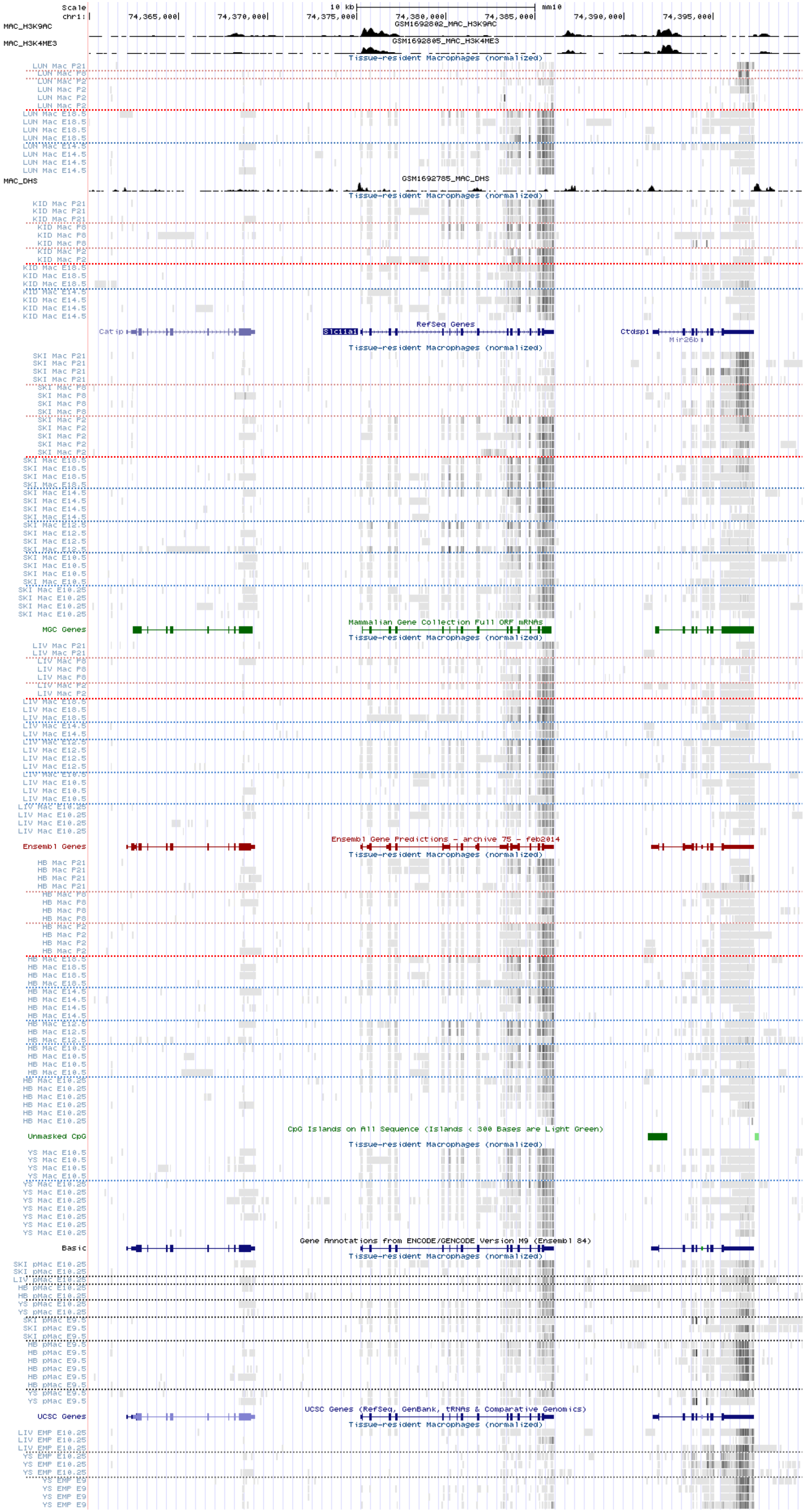

B

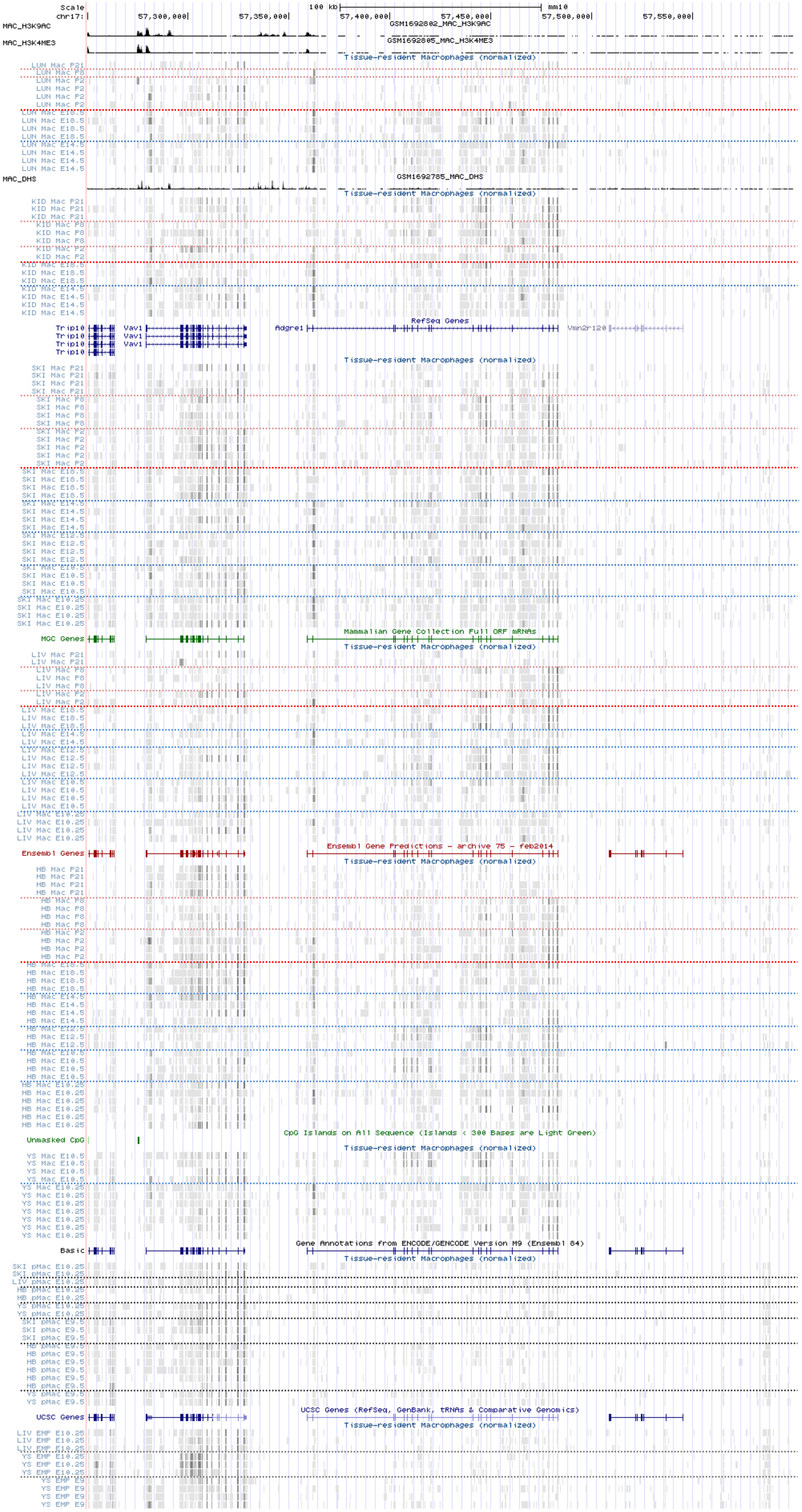

C

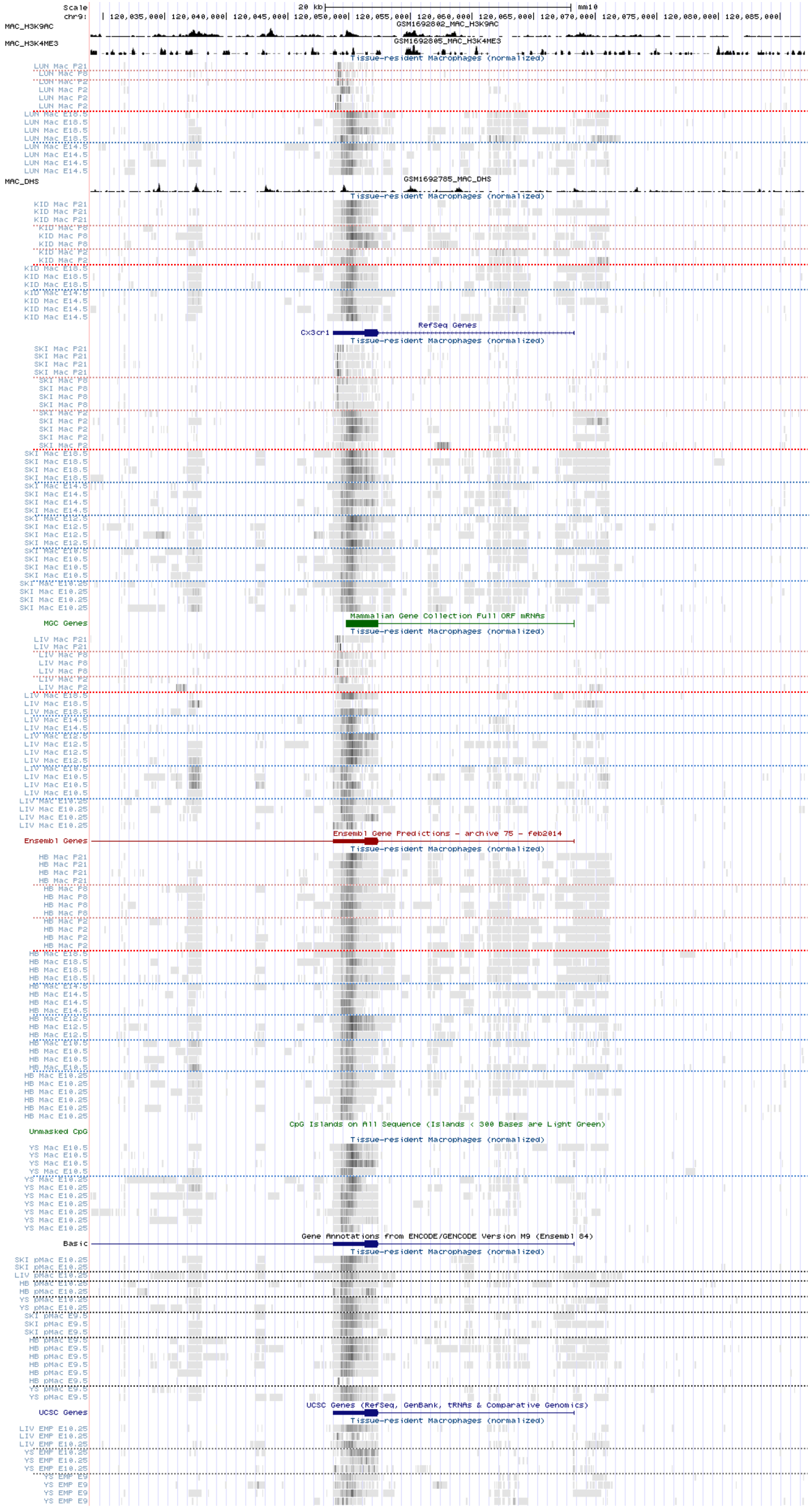
Scale
chr9:
20 kb
mm10
MAC_H3K9AC
GSM1692802_MAC_H3K9AC
MAC_H3K4ME3
GSM1692805_MAC_H3K4ME3
Tissue-resident Macrophages (normalized)
MAC_DHS
GSM1692785_MAC_DHS
RefSeq Genes
Cx3cr1
MGC Genes
Mammalian Gene Collection Full ORF mRNAs
Ensembl Genes
Ensembl Gene Predictions - archive 75 - feb2014
Unmasked CpG
CpG Islands on All Sequence (Islands < 300 Bases are Light Green)
Basic
Gene Annotations from ENCODE/GENCODE Version M9 (Ensembl 84)
UCSC Genes
UCSC Genes (RefSeq, GenBank, tRNAs & Comparative Genomics)

(next page)
Figure S25. Epigenetic activity of *Nramp1* locus during primitive or definitive hematopoiesis. **A.** In vitro embryonic blood cell development: hematopoietic specification and differentiation starting from ESC [172]. Five successive stages of in vitro cell differentiation of ESC-derived embryoid bodies include: mesoderm cell (MES, day 1 of culture), hemangioblast (HB, day 2-3; smooth muscle, endothelial and hematopoietic potential), hemogenic endothelium (HE, day 4-5; endothelial and hematopoietic potential), $CD41^+$ hematogenic precursor (HP, day 7) and $Cd11b^+$ MFs (MAC). Reported epigenetic modifications comprise ChIP-seq analyses directed either at histone modifications or transcription factors, and DNAse 1 hypersensitive sites (DHS), which together inform on chromatin activity. *From top to bottom*: candidate *Nramp1* regulatory elements, labeled by analogy with *NRAMP1* locus (cf Figure 7); mouse chromosome 1 scale; K27me3 (silent chromatin); UCSC genes; DHS (accessible chromatin); RefSeq genes; K27ac (activated chromatin); Ensembl genes; K9ac (transcriptionally active chromatin); Other RefSeq genes; K4me3 (transcriptionally active chromatin); CpG islands; association with TFs (Lmo2, Fli1, Tal1, Runx1, C/ebpb and Pu.1 (Spi1)). Note the presence of a cell-type-specific DHS spanning *Nramp1* TSS in MAC, which correlates with significant binding of few TFs in these cells only, including Pu.1, C/ebpb, Runx1, Tal1 but not Fli1 nor Lmo2 (Pu.1 binding was also detected in HP, with low intensity, data not shown). *Nramp1* TSS and downstream area is also prominently decorated with K27ac, K9ac and K4me3 marks, which indicate gene transcription in MAC. *Nramp1* TSS is labeled F1-like by analogy with human *NRAMP1* regulation; other predicted regulatory areas that appear similarly positioned to human cis-elements are labeled accordingly (F12-like, F5-like, F7-like and F9-like) as well as Ctcf sites that either delimitate (E- and A-like) or flank *Nramp1* locus in downstream (C- and D-like). See text for details. DHS and histone marks also suggest limited activity at F12-, F7- and F9-like predicted determinants in MAC (and HB, F12-like). Epigenetic marks at *Ctdsp1* appear unrelated or inversely related to *Nramp1* activity. The above data indicate, as observed with *NRAMP1*, that *Nramp1* expression is restricted to late stages of myelo-monocytic differentiation and controlled by Pu.1 and C/ebpb TFs, consistent with the myeloid lineage determining roles of these factors. However, *Nramp1* TF binding profiles differ from what was observed with *NRAMP1* (e.g., Pu.1, C/ebpb, Runx1 and Tal1, compared to Figure 7 and Figures S19-22), implying regulatory divergence between mouse and human orthologous loci. **B.** *Nramp1* gene activity in vivo in bone marrow monocyte and progenitor subsets [150]. Cells were sorted using surface antigens ($Cd155^{hi}$, $Cd117^{hi}$ $Cd135^+$ and $Ly6C^{hi}$ or $Ly6C^{lo}$ for cMOP and MDP, respectively; $Cd115^{hi}$, $Cd117^{lo}$, $Cd11c^+$ and $Ly6C^{hi}$ or $Ly6C^{lo}$ for $Ly6C^{hi}$ and $Ly6C^{lo}$ MNs, respectively). ChIP-seq for Pu.1 ($Ly6C^{hi}$ and $Ly6C^{lo}$ MNs) and K27ac (MDP, cMOP, $Ly6C^{hi}$ and $Ly6C^{lo}$ MNs).

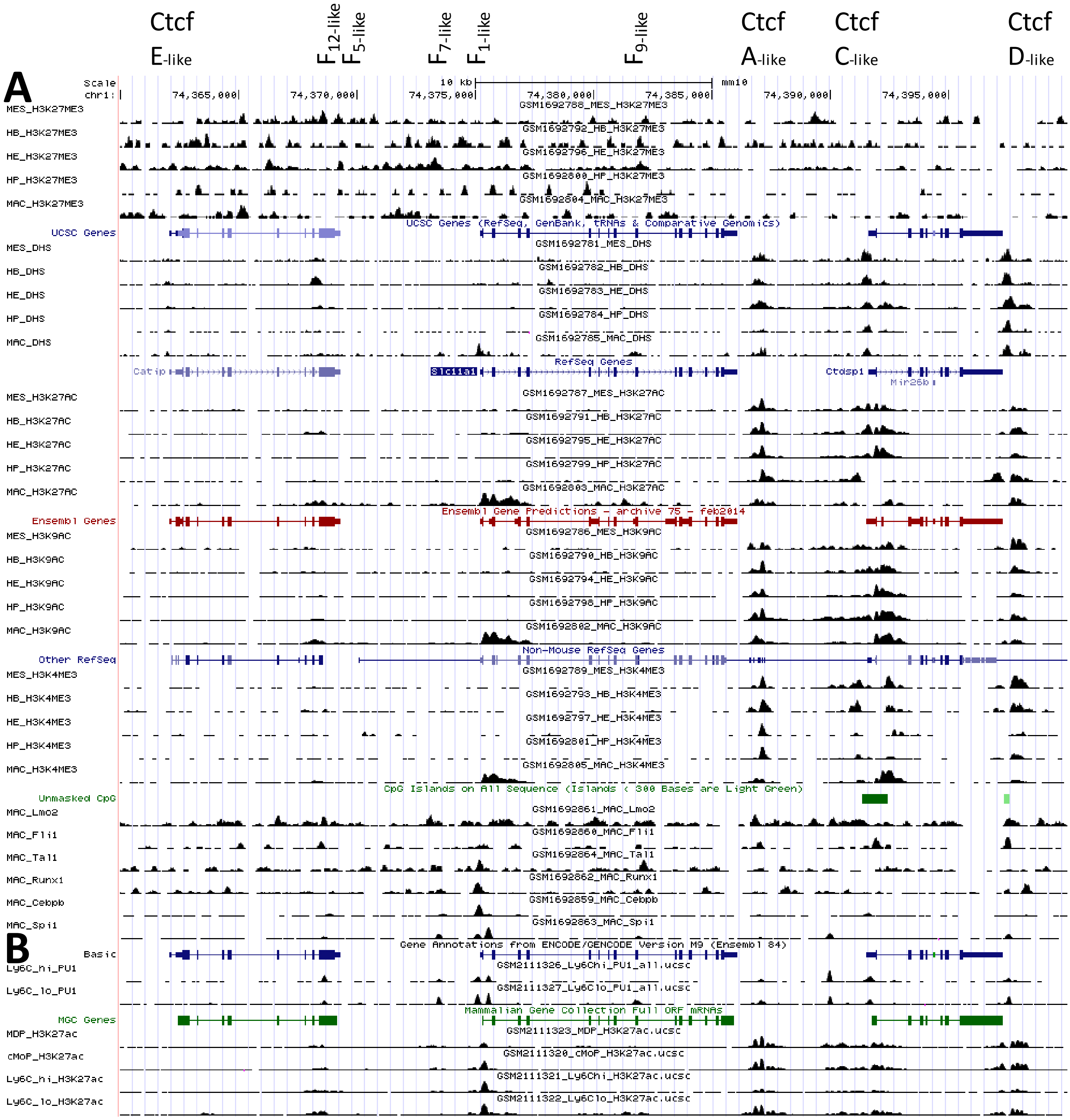
Ctcf
E-like
F12-like
F5-like
F7-like
F1-like
F9-like
Ctcf
A-like
Ctcf
C-like
Ctcf
D-like
A
Scale
chr1:
10 kb
mm10
74,365,000
74,370,000
74,375,000
74,380,000
74,385,000
74,390,000
74,395,000
MES_H3K27ME3
HB_H3K27ME3
HE_H3K27ME3
HP_H3K27ME3
MAC_H3K27ME3
UCSC Genes
UCSC Genes (RefSeq, GenBank, tRNAs & Comparative Genomics)
MES_DHS
HB_DHS
HE_DHS
HP_DHS
MAC_DHS
RefSeq Genes
Catip
Slc11a1
Ctdsp1
Mir26b
MES_H3K27AC
HB_H3K27AC
HE_H3K27AC
HP_H3K27AC
MAC_H3K27AC
Ensembl Genes
Ensembl Gene Predictions - archive 75 - feb2014
MES_H3K9AC
HB_H3K9AC
HE_H3K9AC
HP_H3K9AC
MAC_H3K9AC
Other RefSeq
Non-Mouse RefSeq Genes
MES_H3K4ME3
HB_H3K4ME3
HE_H3K4ME3
HP_H3K4ME3
MAC_H3K4ME3
Unmasked CpG
CpG Islands on All Sequence (Islands < 300 Bases are Light Green)
MAC_Lmo2
MAC_Fli1
MAC_Tal1
MAC_Runx1
MAC_Cebpb
MAC_Spi1
B
Basic
Gene Annotations from ENCODE/GENCODE Version M9 (Ensembl 84)
Ly6C_hi_PU1
Ly6C_lo_PU1
MGC Genes
Mammalian Gene Collection Full ORF mRNAs
MDP_H3K27ac
cMoP_H3K27ac
Ly6C_hi_H3K27ac
Ly6C_lo_H3K27ac

(next two pages)

Figure S26. De novo activation of *Nramp1* promoter in the myelo-monocytic lineage during definitive hematopoiesis in vivo (bone marrow) [25,173,177]. **A**. Myelopoiesis (long term HSC, LT-HSC; short-term HSC, ST-HSC; multipotent progenitor, MPP; CMP; GMP; GN; MN; MF). *From top to bottom*: candidate *Nramp1* regulatory elements labeled by analogy with *NRAMP1* locus (cf Figure 7); mouse chromosome 1 scale; Ctcf sites (murine erythroleukemia cells); UCSC genes; ChIP-seq for K27ac in B6, BXH2 (Irf8 R294C) and *Irf1*$^{-/-}$ BMDM, either naive of treated with Ifn-g; RefSeq genes; K27ac mark along myelopoiesis and in naive BMDM; other RefSeq genes; K4me1 mark along myelopoiesis and in naive BMDM (x2); super-enhancer tracks (MFs and pro-B cells); K4me2 mark along myelopoiesis; Ensembl genes; K4me3 mark along myelopoiesis and in naive BMDM; CpG islands; transposase accessible chromatin subjected to sequencing (ATAC-seq; Lsk (Lin^{-}, Csa1^{hi}, Kit^{hi}, representing HSC), CMP, GMP, MN, GN); Pfam domains in UCSC genes. **B**. Erythropoiesis (MEPs; erythrocytic progenitors A, EryA (Ter119^{+}, CD71^{+}, high FSC); EryB (Ter119^{+}, CD71^{+}, low FSC) and lymphopoiesis (common lymphocytic progenitors, CLP; T lymphocytes Cd8^{+}, CD8 and Cd4^{+}, CD4; B lymphocytes, B; NK cells, NK). *From top to bottom*: candidate *Nramp1* regulatory elements labeled by analogy with *NRAMP1* locus (cf Figure 7); mouse chromosome 1 scale; Ctcf sites (murine erythroleukemia cells); RefSeq genes; naive BMDM RNA-seq data; ChIP-seq for K27ac in B6, BXH2 (Irf8 R294C) and *Irf1*$^{-/-}$ BMDM, either naive of treated with Ifn-g; Other RefSeq genes; K27ac mark in naive BMDM and along erythropoiesis and lymphopoiesis; UCSC genes; K4me1 mark in naive BMDM (2) and along erythropoiesis and lymphopoiesis; super-enhancer (macrophage track); K4me2 mark along erythropoiesis and lymphopoiesis; super-enhancer (pro-B cell track); Ensembl genes; K4me3 mark in naive BMDM and along erythropoiesis and lymphopoiesis; CpG islands; ATAC-seq (MEP, CD4, CD8, B, NK); Pfam domains in UCSC genes. Note *Nramp1* displays the stereotypical behavior of a de novo myelo-monocytic gene (similar to *S100A8* and *S100A9*, for instance) with stepwise acquisition of histone marks during differentiation: *Nramp1* promoter becomes marked at the root of the myeloid commitment point (during the MPP to CMP transition; K4me1 and K4me2 marks), then primed in GMP (K27ac) and activated in mature phagocytes (K4me3; GN, Mono and MF). ATAC-seq areas were detected at F7- and F1-like elements, both starting in CMP and either maintained in mature phagocytes (F1-like) or reduced after GMP (F7-like), suggesting that co-activation of F7- and F1-like elements represent an intermediate state. Absence of epigenetic signal along erythropoiesis and lymphopoiesis demonstrates the myelo-monocytic specificity of *Nramp1* expression.

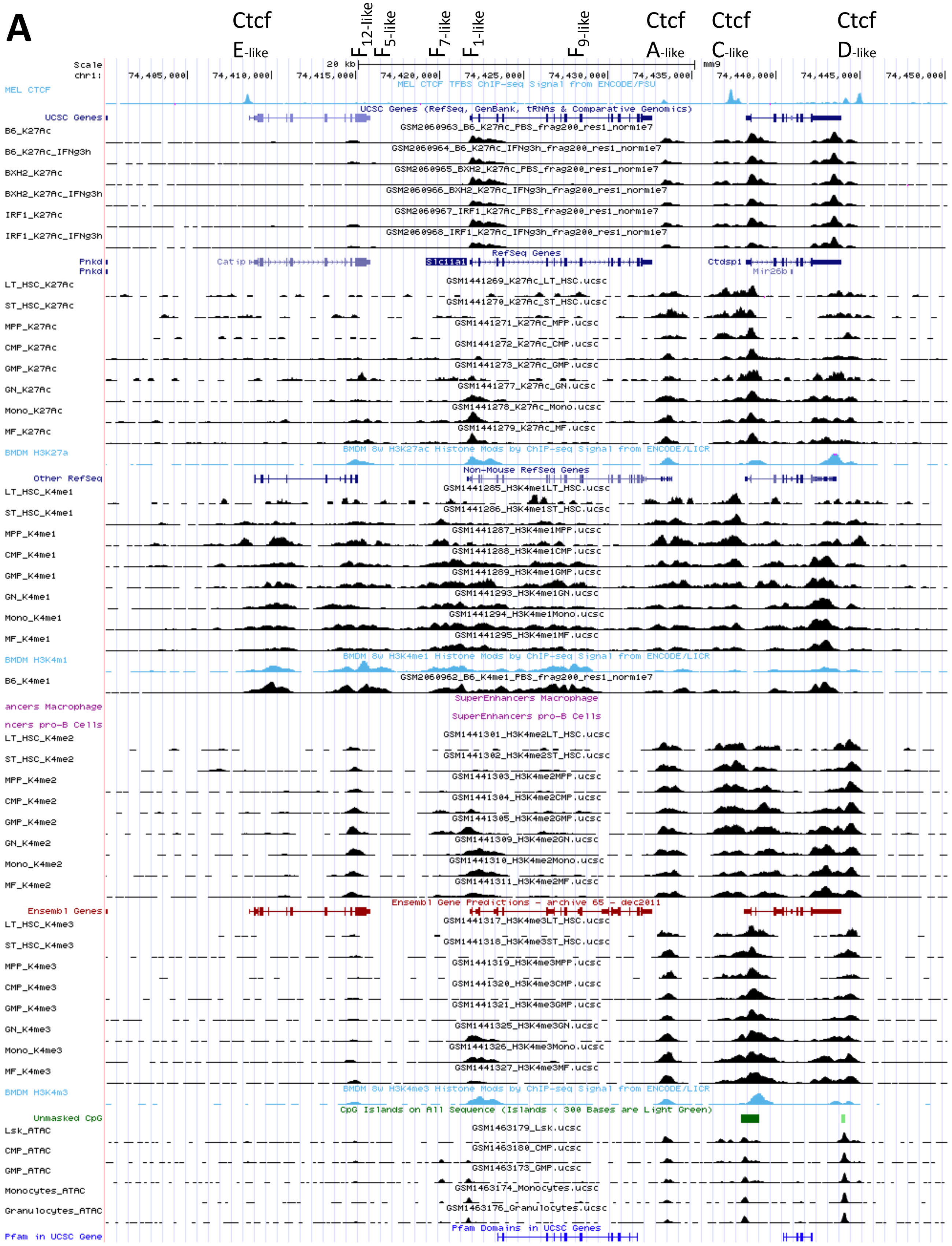
A
Ctcf E-like
F12-like
F5-like
F7-like
F1-like
F9-like
Ctcf A-like
Ctcf C-like
Ctcf D-like
Scale
chr1:
20 kb
mm9
MEL CTCF
UCSC Genes
B6_K27Ac
B6_K27Ac_IFNg3h
BXH2_K27Ac
BXH2_K27Ac_IFNg3h
IRF1_K27Ac
IRF1_K27Ac_IFNg3h
Pnkd
LT_HSC_K27Ac
ST_HSC_K27Ac
MPP_K27Ac
CMP_K27Ac
GMP_K27Ac
GN_K27Ac
Mono_K27Ac
MF_K27Ac
BMDM H3K27a
Other RefSeq
LT_HSC_K4me1
ST_HSC_K4me1
MPP_K4me1
CMP_K4me1
GMP_K4me1
GN_K4me1
Mono_K4me1
MF_K4me1
BMDM H3K4m1
B6_K4me1
ancers Macrophage
ncers pro-B Cells
LT_HSC_K4me2
ST_HSC_K4me2
MPP_K4me2
CMP_K4me2
GMP_K4me2
GN_K4me2
Mono_K4me2
MF_K4me2
Ensembl Genes
LT_HSC_K4me3
ST_HSC_K4me3
MPP_K4me3
CMP_K4me3
GMP_K4me3
GN_K4me3
Mono_K4me3
MF_K4me3
BMDM H3K4m3
Unmasked CpG
Lsk_ATAC
CMP_ATAC
GMP_ATAC
Monocytes_ATAC
Granulocytes_ATAC
Pfam in UCSC Gene

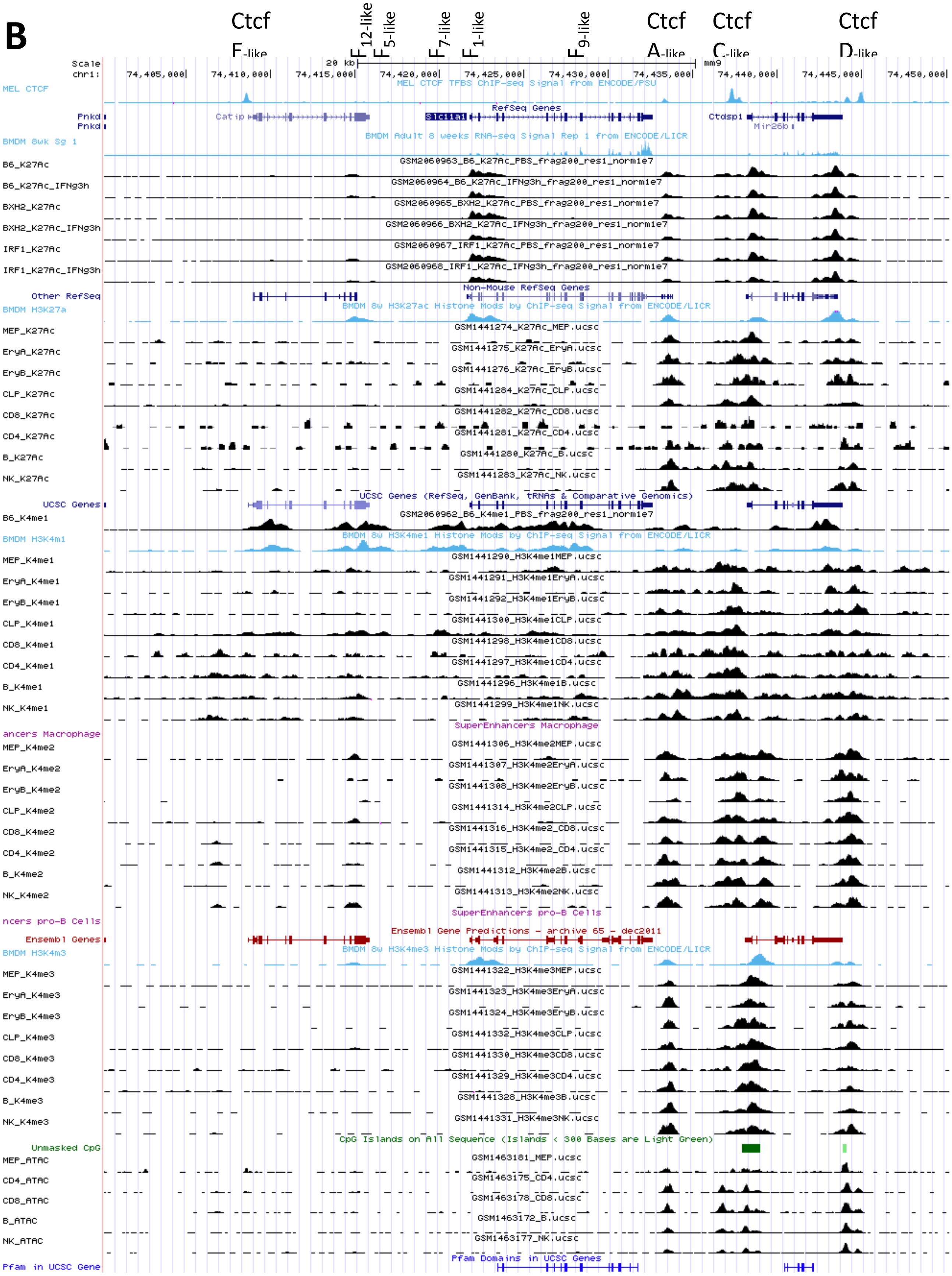
B
Ctcf E-like
F12-like
F5-like
F7-like
F1-like
F9-like
Ctcf A-like
Ctcf C-like
Ctcf D-like
MEL CTCF TFBS ChIP-seq Signal from ENCODE/PSU
RefSeq Genes
BMDM Adult 8 weeks RNA-seq Signal Rep 1 from ENCODE/LICR
Non-Mouse RefSeq Genes
BMDM 8w H3K27ac Histone Mods by ChIP-seq Signal from ENCODE/LICR
UCSC Genes (RefSeq, GenBank, tRNAs & Comparative Genomics)
BMDM 8w H3K4me1 Histone Mods by ChIP-seq Signal from ENCODE/LICR
SuperEnhancers Macrophage
SuperEnhancers pro-B Cells
Ensembl Gene Predictions - archive 65 - dec2011
BMDM 8w H3K4me3 Histone Mods by ChIP-seq Signal from ENCODE/LICR
CpG Islands on All Sequence (Islands < 300 Bases are Light Green)
Pfam Domains in UCSC Genes

(next page)

Figure S27. In vitro and in vivo regulation of *Nramp1* gene expression [120,174–176,178,181]. *From top to bottom*: candidate *Nramp1* regulatory elements labeled by analogy with *NRAMP1* locus (cf Figure 7); mouse chromosome 1 scale; Ctcf sites (murine erythroleukemia cells); H2BK20ac ChiP-seq analyses of microglial BV2 cells (naive or stimulated with LPS with or without Tgfb knockdown) and untreated ESC; super-enhancer track (macrophage); K27ac mark in microglial BV2 cells (naive or stimulated with LPS with or without Tgfb knockdown) and untreated ESC; naive BMDM RNA-seq data; RefSeq genes; K27ac mark in naive BMDM, tissue-resident MFs (peritoneum, small intestine) and blood MNs; Other RefSeq genes; naive BMDM K4me1 mark; CpG islands; ATAC-seq analyses of developmental stages of microglia (yolk sac EMP, early microglia (until E14), pre-microglia (few weeks post-partum); adult mciroglia); UCSC genes; K4me2 mark in developmental stages of microglia (yolk sac EMP, early microglia, pre- and adult microglia); super-enhancer track (pro-B cells); K4me2 mark in bone marrow CMP subtypes ($CD41^+$; $CD41^-$; $Flt3^+$ $Csf1r^+$; $Irf8^-$ GFP^+ $MHCII^+$; $Flt3^-$ $Csf1r^-$ $Irf8^-$ GFP^- $MHCII^-$); Ensembl genes; K4me2 mark along in vitro definitive myelopoiesis (fetal liver HSC; MEP, CMP, GMP); DHS and Pu.1-specific ChiP-seq in GNs; Pfam domains in UCSC genes; K4me2 mark in tissue-resident MFs (peritoneum, small intestine) and blood MNs; K4me3 mark from naive BMDM and BMDM stimulated with LPS (0, 4,12 and 24h). Note MF populations studied in vitro display limited activation of F12-like area (e.g., K27ac in BMDM and BN2 cells vs tissue-resident MFs and blood MNs). In contrast, tissue-resident MFs, including microglia, display high level of K27ac mark at F12-like element similar to TSS/F1-like; in addition, bone marrow myeloid progenitors show predominant K4me2 mark at F12-like element (four out of five CMP subtypes; HSC, MEP and CMP populations).

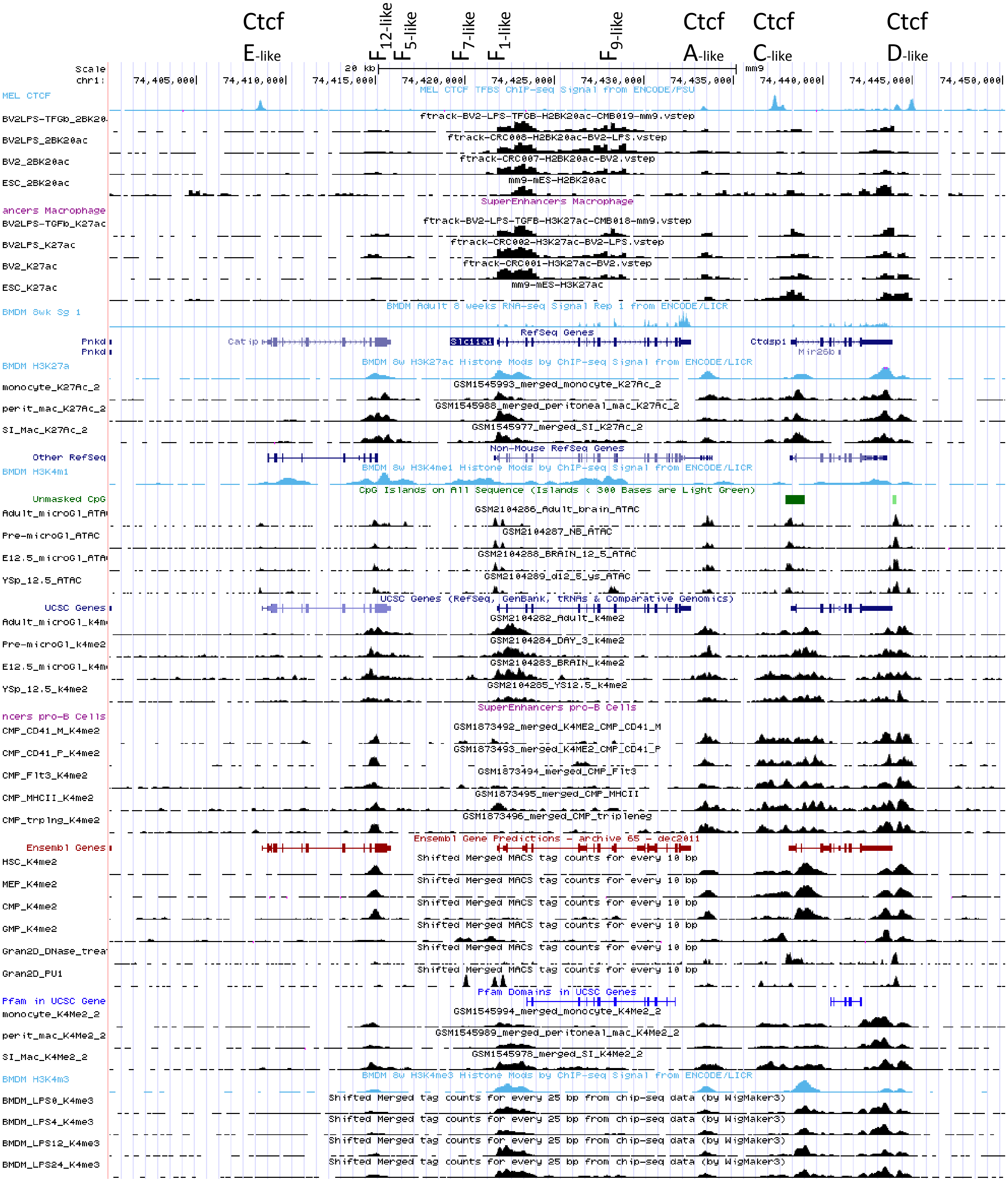
Ctcf
E-like
F12-like
F5-like
F7-like
F1-like
F9-like
Ctcf
A-like
Ctcf
C-like
Ctcf
D-like

(next page)

Figure S28. Regulation of *Nramp1* expression in vitro: association with TFs and response to infectious stimuli [126,180,182,184,185,187,188]. *From top to bottom*: candidate *Nramp1* regulatory elements labeled by analogy with *NRAMP1* locus (cf Figure 7); mouse chromosome 1 scale; ATAC-seq analyses of BMDM either naive or stimulated with lipid A; RefSeq genes; ChIP-seq analyses of Irf3 and RelA in BMDM stimulated with lipid A; Ctcf sites (murine erythroleukemia cells); RNA-seq of naive BMDC; co-ChIP-seq analyses in BMDC (Ctcf or C/ebpb or Pu.1 with H3, K27ac, K4me2 and K4me3); UCSC genes; ChIP-seq analyses of Irf8 and Pu.1 in $Irf8^{+}$ Tot2 cells vs input; CpG islands; ChIP-seq analyses of Irf8 in BMDC (WT, *Batf3*$^{-/-}$ and *Irf8*$^{-/-}$); Other RefSeq genes; RNA-seq of naive BMDM; ChIP-seq analyses of C/ebpa in macrophages, GMP and LSK (Lyn^{-}, $Sca1^{+}$, c-Kit^{-} HSC population [186]); Ensembl genes; H4K8ac and K27ac activation marks in RN2 cells (MLL-AF9) and K27ac mark in BMDM; ChIP-seq analyses in RN2 cells (MLL-AF9) of C/ebpa, C/ebpb, Pu.1, Erg, Myb, Fli1 and p300. Note i) increased accessibility of F9-like element in response to lipid A treatment, which corresponds to time-dependent chromatin association with both Irf3 and RelA; ii) association of F7-like element with C/ebpa and C/ebpb in cell types that do not express *Nramp1* (RN2 AML) or that express *Nramp1* at low level (BMDC, compare RNA-seq data between BMDC and BMDM, *Nramp1* transcript abundance relative to *Ctdsp1* mRNA levels) vs cells that express *Nramp1* at high level (e.g., C/ebpa in Mac; C/ebpb in ESCDM, Figure S25A); and iii) altered pattern of chromatin association of Irf8 in *Batf3*$^{-/-}$ BMDC.

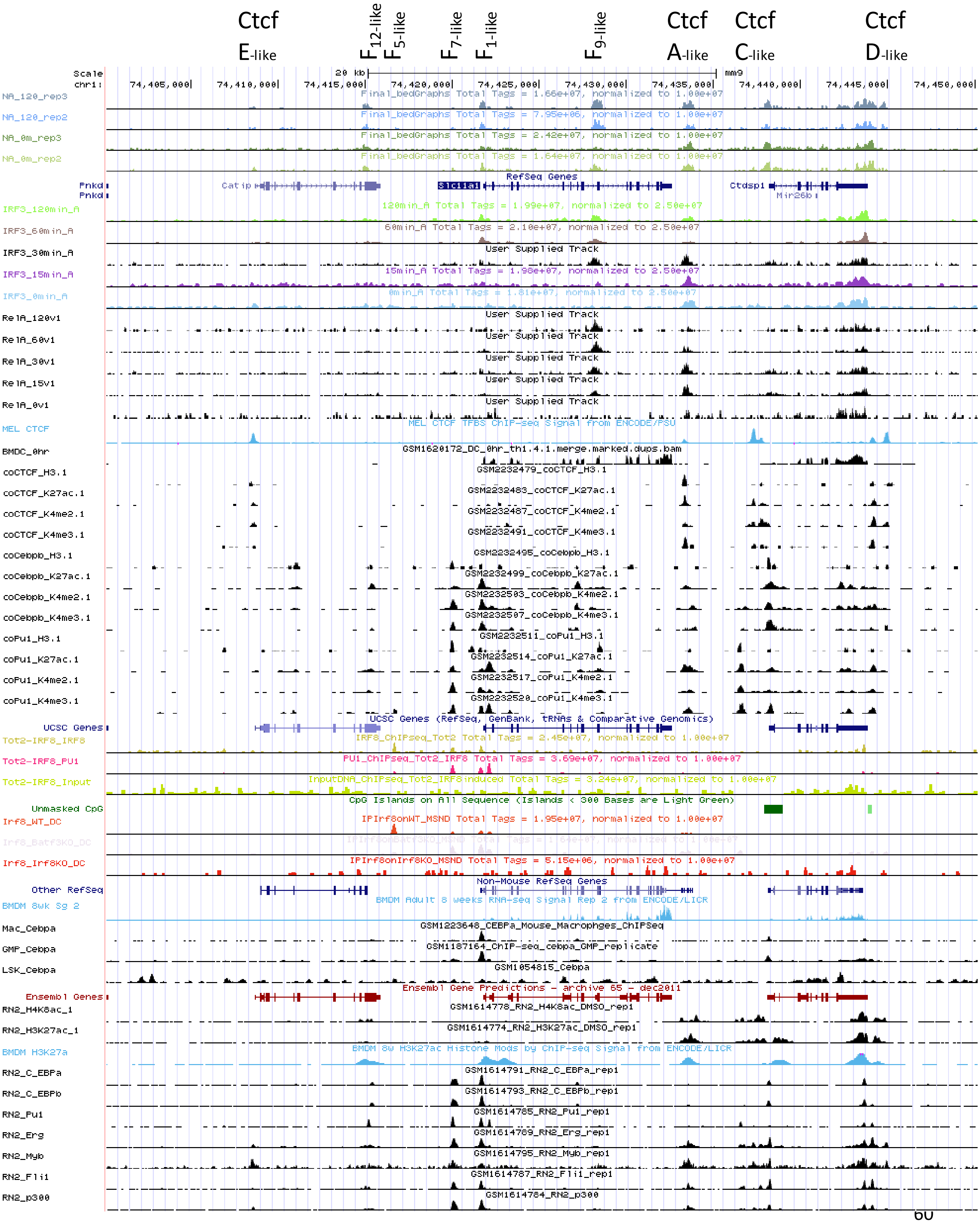
Ctcf E-like
F12-like
F5-like
F7-like
F1-like
F9-like
Ctcf A-like
Ctcf C-like
Ctcf D-like
Scale
chr1:
20 kb
mm9
74,405,000
74,410,000
74,415,000
74,420,000
74,425,000
74,430,000
74,435,000
74,440,000
74,445,000
74,450,000
NA_120_rep3
NA_120_rep2
NA_0m_rep3
NA_0m_rep2
Pnkd
Catip
Slc11a1
Ctdsp1
Mir26b
RefSeq Genes
IRF3_120min_A
IRF3_60min_A
IRF3_30min_A
IRF3_15min_A
IRF3_0min_A
RelA_120v1
RelA_60v1
RelA_30v1
RelA_15v1
RelA_0v1
User Supplied Track
MEL CTCF
MEL CTCF TFBS ChIP-seq Signal from ENCODE/PSU
BMDC_0hr
GSM1620172_DC_0hr_th1.4.1.merge.marked.dups.bam
coCTCF_H3.1
coCTCF_K27ac.1
coCTCF_K4me2.1
coCTCF_K4me3.1
coCebpb_H3.1
coCebpb_K27ac.1
coCebpb_K4me2.1
coCebpb_K4me3.1
coPu1_H3.1
coPu1_K27ac.1
coPu1_K4me2.1
coPu1_K4me3.1
UCSC Genes
UCSC Genes (RefSeq, GenBank, tRNAs & Comparative Genomics)
Tot2-IRF8_IRF8
Tot2-IRF8_PU1
Tot2-IRF8_Input
Unmasked CpG
CpG Islands on All Sequence (Islands < 300 Bases are Light Green)
Irf8_WT_DC
Irf8_Batf3KO_DC
Irf8_Irf8KO_DC
Other RefSeq
Non-Mouse RefSeq Genes
BMDM 8wk Sg 2
BMDM Adult 8 weeks RNA-seq Signal Rep 2 from ENCODE/LICR
Mac_Cebpa
GMP_Cebpa
LSK_Cebpa
Ensembl Genes
Ensembl Gene Predictions - archive 65 - dec2011
RN2_H4K8ac_1
RN2_H3K27ac_1
BMDM H3K27a
BMDM 8w H3K27ac Histone Mods by ChIP-seq Signal from ENCODE/LICR
RN2_C_EBPa
RN2_C_EBPb
RN2_Pu1
RN2_Erg
RN2_Myb
RN2_Fli1
RN2_p300

Table S1. Expression of macrophage iron genes

	Blood	Lung	Spleen	Mac[#] (max)	Ubq	TSS	Specificity[¥]	cS-E[§]
**MN and PMN:**								
SLC11A1	++	+	+	M1		no CpG no RPII NB4	MM	MN
SLC40A1	(+)	+	+	M2		CpG RPII NB4	(MM)	
FTH1	++	++	++	M1	+	CpG RPII NB4	(MM)	MN, Spl, Lng
NCOA4	++	++	++	++	+	CpG RPII NB4	(MM)	MN
FTL	++	++	++	++	+	CpG RPII NB4	(MM)	Spl
S100A8	++	++	++	M1		no CpG no RPII NB4	MM	
S100A9	++	++	++	+		no CpG no RPII NB4	MM	MN, Spl
MCOLN1	+	+	+	(M2)	low	CpG RPII NB4	(MM)	Spl
SLC11A2	+/-	(+)	(+)	+	low	CpG RPII NB4	(MM)	
TFRC	+/-	+	(+)	++	low	CpG RPII NB4	(MM)	
SLC49A1	+/-	+/-	+/-	(+/-)	vlo	CpG RPII NB4		
PCBP1	++	+	++	+	+	CpG RPII NB4	MM	MN, Spl, Lng
PCBP2	+	+	+	+	+	CpG RPII NB4		
CYBRD1	+/-	+	(+)	(M2)	+	CpG RPII NB4	(MM)	
GAPDH	++	++	++	(M1)	+	CpG RPII NB4	(MM)	MN, Spl, Lng
SLC48A1	+/-	+/-	+/-		vlo	CpG RPII NB4	(MM)	
**MN and NOT in PMN:**								
CD91	+/-	+	+	(+/-)	+	CpG w/o RPII NB4	(MM)	
HMOX1	+	+	++	(M2)		CpG w/o RPII NB4	MM	
CD163	+	+	+	M2	~lo	no CpG no RPII NB4	MM	
SLC49A2	+/-	(+)	(+)	(M2)	vlo	CpG RPII NB4 (wk)	MM	
HAMP	+/-	+/-	+/-	(M2)		no CpG no RPII NB4	MM	
STEAP3	+/-	(+)	+/-	+	vlo	CpG RPII NB4	(MM)	
ITLN1	+/-	+/-	+/-	(M2)		no CpG no RPII NB4	MM	
SLC46A1	0	+/-	(+)	+/-	vlo	CpG w/o RPII NB4		
**PMN and NOT in MN:**								
LTF	+	+/-	(+)	+/-		CpG (wk) w/o RPII NB4	MM	
LCN2	+	(+)	(+)	+/-		no CpG no RPII NB4		
**NOT in PMN and MN:**								
SLC39A14	0	(+)	+/-	+		CpG RPII NB4		
TF	0	0	0	+/-		CpG w/o RPII NB4		
TFR2	+/-	0	0	+/-		CpG RPII NB4	(MM)	
LRP2	0	+/-	0			CpG w/o RPII NB4		
SCARA5	0	+/-	0	+/-		no CpG no RPII NB4		
CP	0	+/-	(+)			no CpG w/ RPII NB4		
HEPH	+/-	(+)	(+)	+/-	vlo	no CpG no RPII NB4		
PCBP3	+/-	+/-	+/-	+/-	vlo	no CpG no RPII NB4	(MM)	
PCBP4	+/-	(+)	(+)	(+/-)	low	CpG w/o RPII NB4	MM	

[#]: compared to *SLC11A1* level; grey, no absolute values available
[§]: candidate super-enhancer domain, MN: monocytes; Spl: spleen; Lng: lung
GTEx expression levels (rpkm) [50]:
++ >200>+>20>(+)>5> +/- ;
Ubq +>20>Ubq low>5>Ubq vlo
[¥] MM: myelo-monocytic

Table S2. Macrophage iron gene mRNA expression levels [72].

Gene symbol	H1hESC	CD34	CD14	CD56	CD3	CD19
SLC11A1	1.47	47.82	7289.39	185.89	28.10	154.52
SLC40A1	1.70	7689.32	1681.30	1489.35	1621.83	709.28
FTH1	12478.75	52104.55	94947.65	27761.04	28520.66	14237.49
NCOA4	1944.77	15079.85	26725.16	8013.53	6585.10	6150.27
FTL	30226.82	36754.15	188509.89	32390.51	36014.26	36754.15
S100A8	0	3506.07	261343.64	2098.11	1047.96	3850.38
S100A9	0.60	4317.45	704402.40	3488.48	1958.10	4690.53
MCOLN1	1059.94	239.96	387.08	51.90	72.54	50.70
SLC11A2	1445.00	574.77	480.13	365.51	433.87	250.28
TFRC	2706.30	5399.45	752.67	967.00	1022.10	1378.73
SLC49A1	2125.13	230.89	328.92	286.20	298.91	321.54
PCBP1						
PCBP2	5600.73	17121.23	1967.30	3578.20	9041.77	2306.60
CYBRD1	466.81	1531.89	2657.30	711.21	73.03	138.75
GAPDH	630762.48	110615.15	61495.75	20351.54	25714.78	19192.00
SLC48A1	331.28	739.00	416.27	136.41	260.99	253.37
CD91	1814.69	49.40	2222.43	131.08	100.52	80.72
HMOX1	1690.77	43.28	8200.97	180.38	39.58	417.62
CD163	0.42	48.14	2233.87	91.48	12.53	35.68
SLC49A 2	471.22	30.48	884.00	38.14	52.87	31.47
HAMP	5.74	0	21.08	17.14	0	7.33
STEAP3	1866.10	228.60	193.95	46.93	1.76	45.01
ITLN1	0.41	6.06	139.99	5.36	0	1.74
SLC46A1	245.89	189.16	310.01	160.21	275.61	132.14
LTF	5.82	7.27	4.35	13.93	0	2.95
LCN2	11.24	22.64	5.11	88.17	0	40.74
SLC39A14	4531.96	212.85	46.63	130.75	224.29	148.33
TF	0.33	10.64	8.46	14.86	10.02	3.50
TFR2	75.49	1065.89	17.02	41.36	72.79	26.66
LRP2	82.61	3.59	1.23	0	1.01	0
SCARA5	0.63	0	8.06	17.55	0	30.25
CP	6.48	508.59	29.65	50.96	31.17	36.96
HEPHL1	9.82	6.35	17.26	59.32	6.05	40.86
PCBP3	34.13	92.03	0	18.24	20.22	15.29
PCBP4	893.95	132.47	29.58	195.63	299.66	264.89
